# HighRes_Builder: improved access and modeling of noncanonical residues for protein structure prediction

**DOI:** 10.1093/bib/bbag272

**Published:** 2026-05-31

**Authors:** Yanchao Han, Jianfeng Mei, Gaoshuai Li, Enkang Dai, Hanlei Lu, Chengyun Zhang, Yanlu Zhang, Chenshui Lin, Chuanlong Zeng, Hongliang Duan, Xudong Wang

**Affiliations:** College of Pharmaceutical Sciences, Zhejiang University of Technology, No. 18 Chaowang Road, Gongshu District, Hangzhou, Zhejiang 310014, China; College of Pharmaceutical Sciences, Zhejiang University of Technology, No. 18 Chaowang Road, Gongshu District, Hangzhou, Zhejiang 310014, China; College of Pharmaceutical Sciences, Zhejiang University of Technology, No. 18 Chaowang Road, Gongshu District, Hangzhou, Zhejiang 310014, China; College of Pharmaceutical Sciences, Zhejiang University of Technology, No. 18 Chaowang Road, Gongshu District, Hangzhou, Zhejiang 310014, China; College of Pharmaceutical Sciences, Zhejiang University of Technology, No. 18 Chaowang Road, Gongshu District, Hangzhou, Zhejiang 310014, China; Faculty of Applied Sciences, Macao Polytechnic University, R. de Luís Gonzaga Gomes, Macao 999078, China; College of Pharmaceutical Sciences, Zhejiang University of Technology, No. 18 Chaowang Road, Gongshu District, Hangzhou, Zhejiang 310014, China; College of Pharmaceutical Sciences, Zhejiang University of Technology, No. 18 Chaowang Road, Gongshu District, Hangzhou, Zhejiang 310014, China; University of Chinese Academy of Sciences, No. 19A Yuquan Road, Shijingshan District, Beijing 100049, China; Faculty of Applied Sciences, Macao Polytechnic University, R. de Luís Gonzaga Gomes, Macao 999078, China; College of Pharmaceutical Sciences, Zhejiang University of Technology, No. 18 Chaowang Road, Gongshu District, Hangzhou, Zhejiang 310014, China

**Keywords:** peptide drug, noncanonical amino acids, structure prediction

## Abstract

The growing support for noncanonical amino acids in structure prediction tools such as AlphaFold3 has been largely facilitated by the Chemical Component Dictionary (CCD). However, the limited coverage of modified residues in CCD continues to restrict the application of these models to many biologically and therapeutically relevant peptides. To address this gap, we present HighRes_Builder, a computational method for efficient residue search and automated construction of noncanonical amino acids not currently archived in CCD. We demonstrate the utility of our approach by predicting structures for 3179 noncanonical residues beyond the CCD using AlphaFold3. AlphaFold3 achieved 100% acceptance for both the noncanonical residue monomers and their corresponding ‘GGXGG’ motifs (where *X* denotes the noncanonical residue). Of these, 72.44% of the predicted residue monomer structures concurrently satisfy all five geometric criteria (d_N_C1, d_Ck_Ccarb, d_Ccarb_O_mean, ang_Ck_Ccarb_O_mean, and ang_O1_Ccarb_O2). Furthermore, among the generated motif structures, 85.78% exhibited favorable ω values for the embedded noncanonical residues. Furthermore, by integrating HighRes_Builder with structure prediction systems, AlphaFold3 for linear peptides and HighFold3 for cyclic peptides, we successfully model the conformation of the linear peptide drug Relamorelin and the cyclic therapeutic peptides LUNA18 and JNJ-77242113 in complex with their target proteins, elucidating structural determinants of their mechanism of action. This work establishes a scalable and accurate framework for structure prediction of diverse nonstandard peptides, highlighting its potential to accelerate rational design of peptide-based therapeutics.

## Introduction

The recent release of AlphaFold3 marks a potential breakthrough in structural biology through its innovative integration of residue- and atom-level feature engineering, enabling accurate prediction of modified peptides and small molecules [1, 2, 3]. The application of AlphaFold3 is expected to greatly accelerate the discovery and design of bioactive peptides, particularly in areas such as anticancer peptide research [4]. By improving the accuracy of complex molecular interaction modeling, AlphaFold3 offers new opportunities for rational drug design and the exploration of peptide-based therapeutics. However, despite its technical advancements, the AlphaFold3 model has not been fully open-sourced due to commercial considerations. Nonetheless, it provides valuable methodological insights, particularly in modeling non-natural amino acids and diverse molecular entities. Inspired by its architectural innovations, several derivative models such as Protenix [5], Chai1 [6], and the Boltz [7] series have emerged, accelerating progress in related computational fields. Central to these efforts is the use of the Chemical Component Dictionary (CCD) [8], which serves as a foundational resource for representing noncanonical amino acids. By assigning an appropriate CCD identifier, these models can process nonstandard residues and generate accurate structural conformations, thereby broadening the scope of predictable molecular designs.

However, identifying the appropriate CCD identifier for a specific noncanonical residue or modified compound remains a considerable challenge, particularly for researchers without specialized knowledge in chemistry or structural bioinformatics. Although the CCD database supports querying via chemical composition, simplified molecular-input line-entry system (SMILES) notation [9], or partial structural features, it lacks an efficient mechanism for exact structure-based retrieval. Consequently, users are often required to browse through chemically similar entries and manually compare structural details, a process that is not only time-consuming but also highly prone to error, especially when dealing with subtle atomic- or bond-level variations.

Moreover, the coverage of noncanonical and custom-designed residues in the CCD is inherently limited. It is not uncommon for researchers to find that the specific molecule they intend to model is absent from the database. This limitation highlights a critical gap in current structure prediction pipelines, especially as the field increasingly focuses on designed biomolecules such as synthetic peptides, noncanonical amino acids, and other chemically modified compounds. There is a growing need for more sophisticated and user-friendly tools that can support custom molecular parametrization or automatically generate consistent representations for unseen residues.

**Figure 1:**
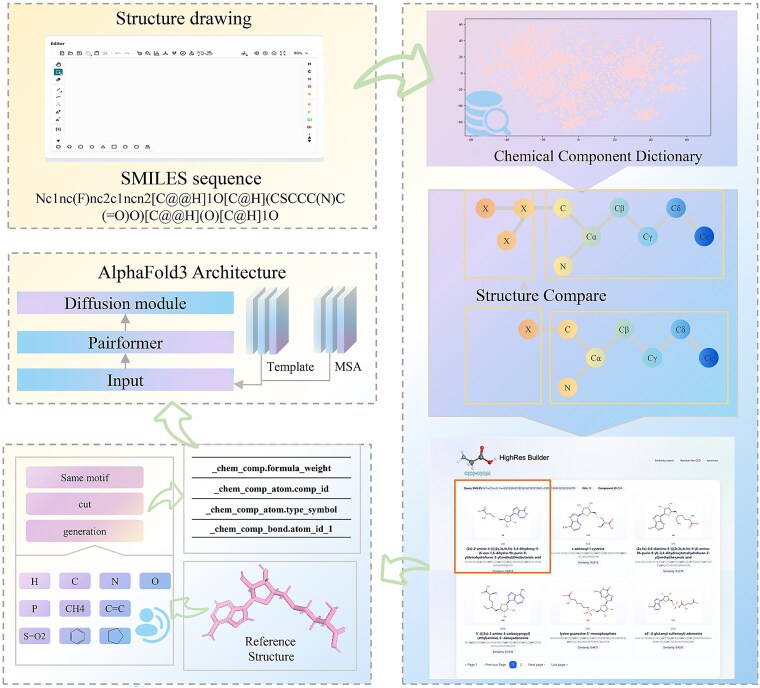
Workflow of HighRes_Builder for noncanonical residue information generation. Users input a SMILES string or interactively draw the chemical structure of a noncanonical amino acid residue. The system queries the CCD and returns candidate references. Upon selection of a suitable topology template, the platform automatically generates formatted residue information in CSV format for direct use with AlphaFold3. A corresponding JSON configuration file is also provided to facilitate model input.

In this study, we aim to develop a user-friendly web platform that enables efficient research and automated generation of specific noncanonical residue representations compatible with the three-letter coding like the CCD identifier, for direct use in prediction models such as AlphaFold3. Using AlphaFold3 as a proof-of-concept framework, we demonstrate the efficiency and practical utility of our approach (see Fig. 1). By streamlining the preparation of nonstandard residue inputs, the platform is designed to make advanced artificial intelligence (AI) modeling accessible to researchers without specialized expertise in biochemistry or computational chemistry, eliminating the need for time-consuming manual input preparation. We anticipate that this method will accelerate progress in related fields by broadening access to state-of-the-art structure prediction tools, facilitating wider evaluation of model performance, and supporting the discovery of novel therapeutic compounds.

## Material and methods

### Dataset

To establish a reference dataset compatible with structure prediction tools, we compiled residue templates from the CCD. These templates serve as reference conformations in our modeling pipeline. To systematically evaluate the performance of HighRes_Builder, we collected residue-like molecules from the ChEMBL_36 database [10, 11]. Here, peptide-embeddable noncanonical residue monomers (alpha/beta/gamma residues) refer to residue-like monomers that can be represented as a single-residue definition for AlphaFold3 and embedded into a peptide/protein chain via canonical peptide-bond formation. Operationally, we require (i) a free amine nitrogen (a nonamide N with at least one H), (ii) an aliphatic backbone path connecting that amine to a terminal carboxyl group (−C(=O)O(H/−)), and (iii) a shortest-path length consistent with alpha/beta/gamma scaffolds (two/three/four bonds between the amino nitrogen and the carboxyl carbon), while δ + cases are excluded. Residue candidates were derived from the ChEMBL chemrep table, which provides canonical SMILES strings for all registered compounds. A custom RDKit-based [12] pipeline was implemented to identify alpha, beta, and gamma type residues molecules

For each ChEMBL SMILES, the molecule is parsed, salts are removed, and explicit hydrogens are suppressed for topology analysis. The procedure first detects carboxylate groups by locating sp^2^ carbon atoms that are bonded to one doubly bonded oxygen and one singly bonded oxygen, corresponding to the carbonyl carbon of a carboxylic acid or carboxylate. Candidate amino nitrogens are collected among all nitrogen atoms after excluding those in nitro and nitrile groups. Optional flags control whether amide nitrogens and quaternary ammonium centers are accepted; in the default setting, amide nitrogens are discarded, and nitrogen centers with a degree greater than three are excluded.

For each candidate molecule, the shortest path between every carboxylate carbon and every candidate nitrogen is computed as the number of bonds along the path. The minimum path length determines the classification. Molecules in which the carboxylate carbon and the amino nitrogen are separated by two, three, or four bonds are labeled alpha, beta, and gamma type residues, respectively, whereas molecules with longer separations (δ or higher) are discarded.

For every molecule that passes these rules, a robust InChIKey [13] is generated. Before InChIKey calculation, salts are stripped and, when enabled, standardization routines are applied to clean up valence states and to canonicalize tautomeric forms. The resulting InChIKey is then used to map ChEMBL compounds to CCD entries. CCD structures are read from the public components-pub.sdf.gz archive. For each CCD record, an identifier and an InChIKey are obtained either from stored properties or by recomputing the InChIKey after light sanitization. Mapping is performed in two steps. First, an exact match on the full InChIKey is attempted. When this fails, a second pass considers only the connectivity layer, i.e. the first fourteen characters of the InChIKey, which allows for differences in protonation state or tautomerism while preserving the underlying graph. If several CCD components share the same key, either all candidates are retained or the first one is selected, depending on the configuration. In practice, template selection is most reliable when the query is structurally identical to a CCD component. Accordingly, we treat matches as high confidence only under strict identity conditions. Namely, when there is an exact full InChIKey match or when fingerprint similarity yields Tanimoto = 1.0, indicating graph-level equivalence between the query and the CCD entry. Outside these identity cases, ambiguity can arise because multiple CCD components may plausibly correspond to the same query, particularly under relaxed matching (e.g. connectivity-layer matching that tolerates protonation/tautomer differences). In such situations, we do not interpret a single automatically selected template as uniquely correct; instead, we recommend that users inspect the returned candidates and, where appropriate, retain multiple CCD candidates for downstream evaluation (e.g. running alternative inputs and comparing geometry/AlphaFold3 outcomes). This guidance makes clear when users can trust a selected template with high confidence and how to proceed when the mapping is intrinsically ambiguous.

Additional manual curation removed molecules with metal centers, highly charged coordination complexes, or other chemotypes that are not robustly handled by RDKit-based conformer generation. Based on this work, we have curated 3179 residue molecules not included in the CCD and encompassing alpha, beta, and gamma type residues. The detailed information about the dataset is displayed in Table S1.

### Chemical Component Dictionary reference library and similarity search

The curated ChEMBL-derived CCD assignments form the basis of the reference library used for similarity search in HighRes_Builder. For each library component, a circular Morgan fingerprint with radius 2 and length 2048 bits is computed with RDKit [14]. On these fingerprints, HighRes_Builder supports four similarity coefficients: Tanimoto, Dice, cosine, and Kulczynski. Tanimoto similarity is used as the default ranking measure in all experiments, while the other coefficients can be selected through the interface for sensitivity analyses or to accommodate user preferences.

When a user submits a residue molecule, either by pasting a SMILES string or by drawing a structure in the embedded Ketcher editor and converting it to SMILES, the query structure is sanitized and canonicalized, and a Morgan fingerprint is generated using the same parameters as for the library. Similarity scores between the query and all library components are then computed under the chosen coefficient. CCD entries with a Tanimoto similarity of 1.0 are treated as exact structural matches. If no exact match is found, HighRes_Builder returns the top-k most similar components, with k equal to 10 by default and user configurable. For each candidate, the platform reports the CCD identifier, the three-letter code, similarity scores under all four coefficients, and a two-dimensional depiction generated by RDKit. Users can either accept the highest-scoring template or manually choose any of the returned candidates as the basis for topology generation. It should be noted that the generated 3D coordinate information originates entirely from RDKit rather than from CCD templates, while the CCD templates are used solely to provide topology information.

### Conversion to residue-compatible atom naming

Because RDKit uses numeric atom indices while protein structure prediction models expect residue-style atom names that follow CCD conventions, we implemented a rule-based atom-renaming procedure. The amino-acid-like scaffold is recognized using SMARTS pattern matching [15]. For alpha residues, the backbone nitrogen, alpha carbon, carbonyl carbon, and carbonyl oxygen are identified and assigned the conventional labels N, CA, C, and O. For beta and gamma residues, the additional backbone carbons are detected and annotated as additional backbone carbons (for example, CB and CG or analogous labels, depending on the backbone topology). Side-chain attachment points are located as the first heavy atom branching from the terminal backbone carbon, and side-chain atoms are named following CCD-style patterns such as CB, CG, CD, CE, and CZ for carbon chains, with analogous patterns for heteroatoms. Hydrogen atoms are named according to the heavy atom to which they are attached, and numeric suffixes are used when multiple hydrogens share the same heavy atom. For atoms that lack direct analogues in standard amino acids, generic but CCD-compatible names are assigned while maintaining uniqueness within each residue. The resulting mapping from atom index to residue-style atom name is stored together with the coordinates and connectivity and is used when constructing AlphaFold3 inputs.

### Model

#### AlphaFold3

The advent of AlphaFold3 marks a transformative advance in molecular structure prediction, largely driven by its integrated representation of residue and atom-level features, which extends its capability to model modified peptides and small molecule ligands. Architecturally, AlphaFold3 streamlines the multiple sequence alignment (MSA) processing by substituting the evoformer from AlphaFold2 [16] with a lightweight pairformer module. More notably, it abandons the frame- and torsion-based structure module used in AlphaFold2 and adopts a diffusion-based generative process that directly infers atomic positions in 3D space. This shift enables unified modeling of heterogeneous molecular assemblies, including nonprotein residues and small molecules. These technical advances are poised to greatly expedite the identification and optimization of bioactive peptides, offering new opportunities in areas such as peptide therapeutics.

When using AlphaFold3 locally, users need to construct an input file in a specified JSON format. This file includes sequence information of the proteins, peptides, or nucleic acids to be predicted, as well as other possible modification conditions. The inclusion of these modification conditions provides AlphaFold3 with more capabilities compared to previous prediction models. For the challenge of predicting noncanonical amino acids in protein or peptide sequences, AlphaFold3 allows control over the chemical and physical properties of noncanonical amino acids in prediction results through a combined input method involving unnatural amino acid modification indices and CCD codes. However, if some uncommon residues are not listed in the CCD, the above-mentioned input method cannot be used. In such cases, HighRes_Builder allows the user to assign a custom Component ID, analogous to a CCD code, to newly constructed residue entities. Through a CSV-formatted file, users can obtain corresponding atomic properties, bond connectivity, and residue metadata. The CSV files generated by HighRes_Builder contain all necessary information for AlphaFold3 to accurately construct these residue features.

To enable AlphaFold3 to support CSV-formatted information conveniently, we modified the module of AlphaFold3 that corresponds to the CCD. This modification allows AlphaFold3 to accept two types of inputs: multiple individual CSV files specified by the user, and folders containing multiple CSV files. The detailed pseudocode for extending user-defined CCD files is provided as Supplementary Algorithm 1.

#### HighFold3

The release of AlphaFold3 has markedly advanced the modeling of biomolecular complexes and supports the inclusion of noncanonical amino acids through definitions provided by the CCD. However, its reliance on existing training data limits accurate prediction of cyclic peptide structures, falling short of the precision required for this important class of molecules. To address this gap, our previous work developed HighFold3 [17], an extension of the AlphaFold3 framework that incorporates a novel Cyclic Position Offset Encoding Matrix (CycPOEM). This model is tailored for predicting cyclic peptide structures. Benchmarking results demonstrate that HighFold3 achieves superior performance in cyclic peptide structure prediction compared to existing methods, including.

HighFold [18], HighFold2 [19], CyclicBoltz1 [20, 21], NCPepFold [22], and CABS-flex [23]. The input format follows that of AlphaFold3.

### Metrics

#### AlphaFold3 acceptance

To assess the compatibility of Highres_Builder-generated noncanonical residues with AlphaFold3, we defined a metric based on the model’s ability to complete a prediction task. An input feature was considered accepted if AlphaFold3 executed without error and produced a valid structural model. The acceptance rate for a given residue or modification was then quantified as the proportion of successful prediction runs.

#### Geometric validity

To assess the quality of the predicted structures, we performed basic geometric validation using five metrics [24–26].

d_N_C1 is the distance between the backbone amine nitrogen (N) and the first carbon along the backbone chain (C1). For alpha-amino acids, C1 corresponds to the Cα atom. The reference range is 1.382–1.534 Å.

d_Ck_Ccarb is the distance between the terminal backbone carbon (Ck; where for alpha: C1, beta: C2, gamma: C3) and the carboxyl carbon (Ccarb), analogous to the Cα–C bond in alpha-amino acids. The reference range is 1.441–1.609 Å (Cα–C = 1.525 ± 0.021 Å, mean ± 4σ).

d_Ccarb_O_mean is the average distance from the carboxyl carbon (Ccarb) to the two carboxyl oxygen atoms (O1 and O2). The reference range is 1.151–1.311 Å (C–O = 1.231 ± 0.020 Å, mean ± 4σ).

ang_Ck_Ccarb_O_mean is the mean of the two ∠(Ck–Ccarb–O) angles measured for O1 and O2. This metric reflects the approximate sp^2^ hybridization geometry around the carboxyl carbon. The reference range is 114.0–127.6° (Cα–C–O = 120.8 ± 1.7°, mean ± 4σ).

ang_O1_Ccarb_O2 is the carboxyl angle ∠(O–Ccarb–O). The reference range (sanity band) is 110–130°, consistent with the near-trigonal planar geometry expected for an sp^2^-hybridized carboxyl carbon (~120°).

#### Peptide-bond dihedral angles

For each residue embedded in peptides with the ‘GGXGG’ motif, we verified the peptide backbone dihedral angles to confirm the formation of a continuous, correctly ordered amide backbone. Records were classified based on the peptide-bond dihedral angles ω_prev and ω_next. Good, both |ω| ≥ 150° or |ω| ≤ 30°. Borderline, at least one ω with 120° ≤ |ω| < 150° and no ω in the bad range. Bad, any ω with 30° < |ω| < 120° or missing values.

#### Root mean square deviation

In this study, we employed root mean square deviation (RMSD) evaluation to assess prediction accuracy. The calculation for this metric is as follows:


$$RMSD\left({x}^{\mathrm{native}},{x}^{\mathrm{model}}\right)=\sqrt{\frac{1}{N}\sum_{i=1}^N\kern0.1em \sum_{j=1}^3\kern0.1em {\left({x}_{ij}^{\mathrm{native}}-{x}_{ij}^{\mathrm{model}}\right)}^2}$$

where ${x}^{\mathrm{native}}$ and ${x}^{\mathrm{model}}$ are the conformations of the native and the predicted molecules, respectively. The subscript $i$ index the total number of atoms N, which can be all atoms, backbone atoms, and Cα atoms, while $j$ is the atomic coordinates.

## Results

### Dataset distribution

Before presenting a detailed analysis of our method’s results, we first provide a comprehensive description of the test dataset to facilitate a deeper understanding of our experimental setup and model capabilities. Using ChEMBL_36, our screening pipeline identified 12,210 alpha/beta/gamma noncanonical residue monomer candidates; 3845 (31.5%) passed all scope and robustness filters, while 8365 (68.5%) were excluded with machine-readable reasons. Among excluded candidates, 5947 (48.7% of all candidates) were removed solely due to the predefined complexity/robustness limits, and 8171 (66.9%) triggered at least one such limit, with the most common flags being molecular weight above threshold (7323), ring count above threshold (6024), heavy-atom count above threshold (4871), and hetero-atom count above threshold (3471) (flags are nonexclusive).

**Figure 2:**
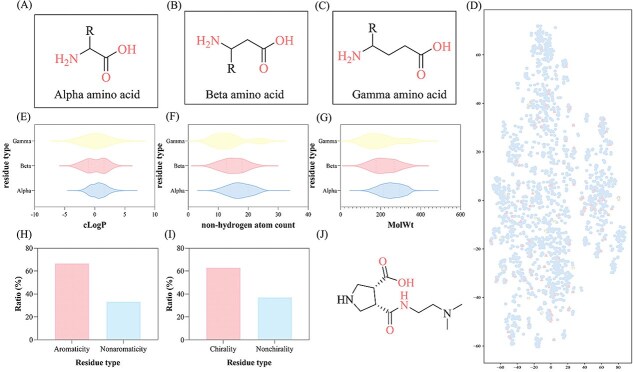
Dataset composition and molecular properties. (A–C) Structural classification of amino acid types represented in the dataset. (D) Distribution of amino acid molecules. (E) Distribution of cLogP values. (F) Distribution of nonhydrogen atom counts. (G) Distribution of MolWt values. (H) Ratio of aromatic versus nonaromatic amino acids. (I) Ratio of chiral to achiral molecules. (J) Representative example of a beta-amino acid structure.

Finally, remove the molecules included in the CCD, 3179 molecules are contained as the final CCD-absent dataset used in this study. The dataset consists of 3179 distinct samples, encompassing three prevalent types of residue backbones: alpha, beta, and gamma amino acids. The structural characteristics of these backbone architectures are visually summarized in Fig. 2A. In terms of chemical diversity, the side chains incorporate a variety of functional groups, including acetyl among others, demonstrating the ability of our model to handle a broad spectrum of molecular modifications. The detailed distribution of our dataset is displayed in Fig. 2D. We calculated the cLogP values for the dataset, with the distribution presented in Fig. 2E. Most molecules exhibit cLogP values between −2 and +3. Figure 2F shows the distribution of nonhydrogen atom counts across different residue classes. The molecular weight (MolWt) distribution of the samples, as illustrated in Fig. 2G, further highlights the chemical range covered by the dataset. The alpha-residue molecules exhibit molecular weights ranging from 180 to 380, whereas beta-residue molecules span from 200 to 400. This diversity in both backbone topology and side-chain chemistry ensures a rigorous and representative evaluation of our approach.

These noncanonical residues were processed in batch by our method using their SMILES representations. In the initial matching phase, samples were successfully matched with a 100% similarity rate in the CCD library, enabling users to directly employ these CCD templates for structure prediction tasks. For molecules without perfect matches, we employed RDKit to generate 3D atomic coordinates.

It is important to note that molecules processed through RDKit are typically annotated using small-molecule naming conventions rather than residue-specific nomenclature, resulting in generic atom types (e.g. C, O) that do not reflect their residue characteristics. To address this limitation, we developed an algorithm to convert the atom naming of these residue molecules into standard residue-compliant nomenclature. Finally, we calculated the required features for AlphaFold3, including CA, CB, and N, and compiled this information into a CSV-formatted input file to facilitate structure prediction (see Methods and Materials).

#### The HighRes_Builder web platform

**Figure 3:**
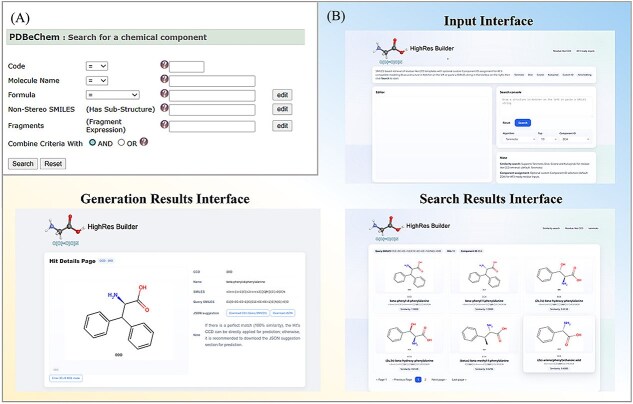
The comparison of (A) the original CCD website versus (B) the HighRes_Builder.

The original CCD web interface offers only a limited set of search options, which often fail to precisely locate relevant residue entries (see Fig. 3A). Furthermore, certain residues that have been manually modified by researchers for specific applications may not be included in the standard dataset, thereby hindering the prediction of peptides containing such noncanonical residues.

To address these limitations, we have developed a user-friendly online platform to ensure efficient access to our tool, as shown in Fig. 3B. Our website supports multiple query modes, including structure-based and SMILES-based searches, and incorporates flexible similarity calculation methods. The similarity-search engine operates on precomputed Morgan fingerprints of the ChEMBL-derived CCD library and exposes Tanimoto, Dice, cosine, and Kulczynski coefficients as selectable options. Coordinate-generation, atom-renaming, and JSON-assembly routines are organized as independent services that can be invoked from the web interface. Upon completion of a search, the platform generates an interactive results interface that displays the chemical structures of matched residues, along with their CCD identifiers and similarity scores relative to the query molecule. All results are systematically ranked by their similarity scores. A perfect score of 1.00 indicates that the query molecule is fully contained in the CCD, allowing users to directly utilize the corresponding CCD entry in AlphaFold3 for structure prediction. In cases where no exact structural match is found, users may select a closely related residue as a reference. The platform then automatically generates a CSV file containing all required feature information for AlphaFold3 input. To further assist users, we also provide a well-annotated JSON example file demonstrating how to effectively incorporate residue information into the prediction pipeline.

This streamlined workflow is designed to minimize the need for specialized knowledge in chemistry or biology. As long as the molecular structure is known, users can efficiently perform structure predictions without the time-consuming manual editing of CIF files, thereby accelerating the research process while reducing potential human error.

### The structure prediction of noncanonical residues in the CHEMBL database

**Figure 4:**
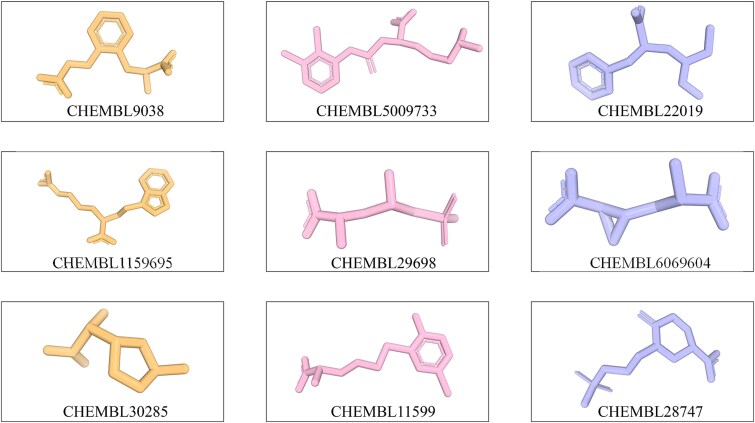
The representative examples of the predicted structure of amino acids that are not included in the CCD.

The 3179 samples were submitted to AlphaFold3 for structural prediction. The RDKit-based method consistently maintained AlphaFold3 acceptance of 100% across all residue types, demonstrating the robustness of our approach. We manually examined 200 predicted amino acid monomer molecules, most of which exhibit reasonable chemical structures. Figure 4 displays 12 representative molecular structures predicted by AlphaFold3. The chemical constitution of each molecule is identical to its corresponding 2D structure in the ChEMBL database. This confirms that AlphaFold3 has accurately modeled the true chemical structures based on our input. The predictive capability of AlphaFold3 is further demonstrated through specific examples. Notably, it produced a precise conformational output for CHEMBL6069604, a noncanonical residue characterized by a tricyclic ring. The model also yielded a correct prediction for the phosphate-containing residue CHEMBL29698. However, if such molecules are absent from existing CCD libraries, researchers aiming to utilize AI-based prediction models for noncanonical residues would face substantial manual effort in generating conformer coordinates and preparing model-compliant input data. This challenge is particularly acute for users unfamiliar with AlphaFold3’s input specifications, where the process of compiling required CCD-aligned structural and feature information presents a significant technical barrier.

Our platform effectively addresses this bottleneck by automating the generation of standardized JSON configuration files containing complete molecular representation data, enabling direct prediction of these nonstandard residues across diverse peptide contexts. This streamlined workflow demonstrates the potential to significantly lower the accessibility threshold for state-of-the-art structure prediction tools.

**Figure 5:**
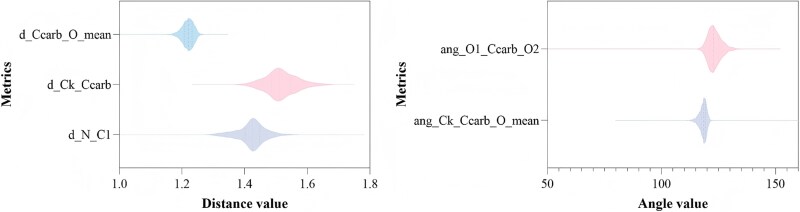
Quality assessment of predicted residue structures.

To assess the quality of the predicted structures, we performed basic geometric validation using five metrics. As shown in Fig. 5, the predicted conformations generally exhibit reasonable geometry, with the majority of bond lengths and angles falling within expected ranges. Specifically, 81.69% of the structures have d_N_C1 values within the reference range, and 97.61% have ang_Ck_Ccarb_O_mean values within the reference range. Overall, 72.44% of the structures concurrently satisfy all five geometric criteria, suggesting that they are of high structural integrity.

**Figure 6:**
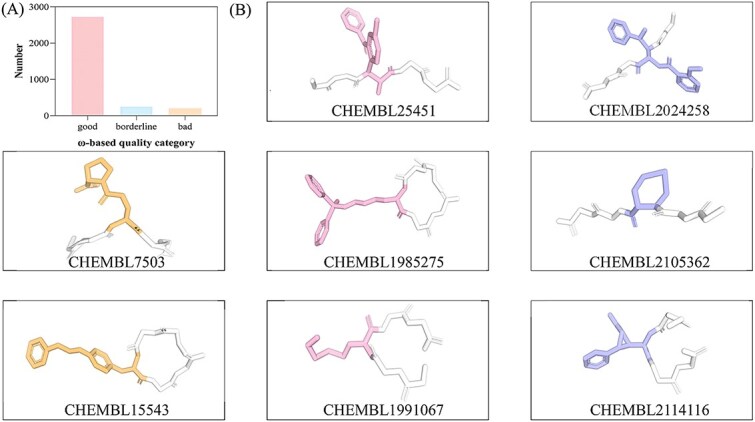
The performance of predicted noncanonical residue with the ‘GGXGG’ motif (where X denotes the noncanonical residue) (A) Distribution of ω-based quality categories for nonstandard residues. (B) The representative examples of the predicted structure of amino acids embedded in peptides with the ‘GGXGG’ motif.

To further demonstrate the practical utility of our method, we modeled all noncanonical residues embedded in peptides with the ‘GGXGG’ motif (where *X* denotes the noncanonical residue). Consistent with the single-amino acid modeling experiment, AlphaFold3 demonstrated that our method maintained a 100% AlphaFold3 acceptance rate in this task. For residue molecules, peptide-backbone dihedral angles were checked to ensure the presence of a continuous amide backbone with the correct chain order. Figure 6 demonstrates the distribution of ω. The most frequently predicted structure exhibits an excellent ω value, accounting for 85.78% of cases (Fig. 6A). It demonstrates that AlphaFold3 generates biologically plausible structures in most instances. A total of 8 representative predicted peptides are displayed in Fig. 6. All residues were successfully structuralized without atomic clashes or steric conflicts, forming chemically plausible conformations within the peptide backbone. For instance, the peptide incorporating CHEMBL2024258, which contains two aromatic rings, was assigned an appropriate conformation. Additionally, the peptide containing CHEMBL15543, a residue with a long side chain, also achieved a satisfactory fold. These results confirm that our pipeline effectively generates biophysically valid input representations compatible with AlphaFold3, while also illustrating the model’s capability to interpret and accurately structure diverse nonstandard residues based on their chemical features.

To evaluate the validity of our feature construction approach, 50 noncanonical amino acid residues were selected from the CCD and processed using our HighRes_Builder pipeline, which incorporates two conformation generation strategies, RDKit and Open Babel [27], to enable a controlled evaluation of coordinate generation strategies. This evaluation included quantitative assessments of feature geometric quality and corresponding prediction performance.

**Figure 7:**
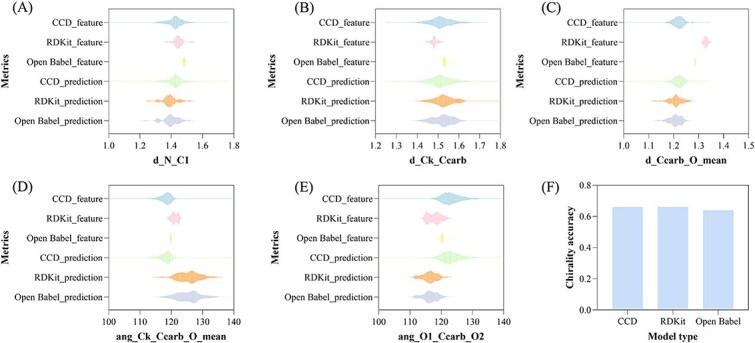
Geometric quality of the conformations generated by the feature-based strategy and AlphaFold3. (A) Distribution of d_N_C1 values. (B) Distribution of d_Ck_Ccarb values. (C) Distribution of d_Ccarb_O_mean values. (D) Distribution of ang_Ck_Ccarb_O_mean values. (E) Distribution of ang_O1_Ccarb_O2 values. (F) Chirality accuracy of AlphaFold3 predictions based on different conformation generation strategies.

The resulting features were subsequently fed into the structure prediction model, and the predictions were systematically compared (Fig. 7). The distributions of d_Ccarb_O_mean values generated by RDKit and Open Babel differ from those of the CCD reference, whereas the distributions of their corresponding predictions are similar, both peaking near 1.2 Å. Regarding the angle values, most predictions exhibit reasonable distributions, ranging from 110° to 130°. In addition, Fig. 7F demonstrates the chirality accuracy of AlphaFold3 predictions based on different conformation generation strategies. No significant difference was observed in this metric across the strategies. The chirality accuracy of the predictions remains around 64%. Unsurprisingly, AlphaFold3 cannot distinguish the chirality of residues and consistently predicts D-amino acid residues as L-amino acid residues, which is consistent with previous findings [4, 28] that AlphaFold3 does not properly handle chirality, even when the input features reflect the correct chirality.

**Figure 8:**
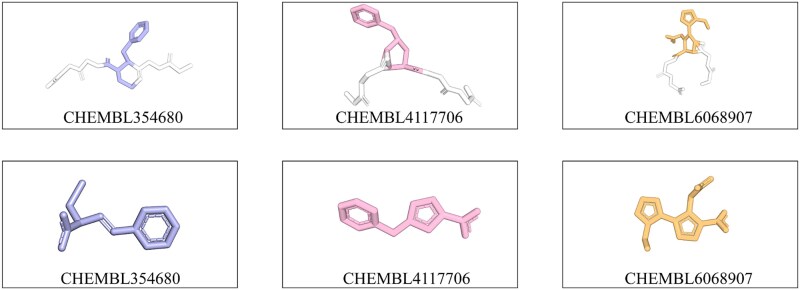
The common failure of predicted structures.

As shown in Table 1, structural similarity between predictions derived from the CCD and RDKit feature sets was assessed using RMSD, yielding an RMSD_all_atom of 0.903 Å. This result indicates that, although differences exist between the predictions generated from the two feature representations, they remain within an acceptable range. It is worth noting that RDKit was employed here primarily as a proof of concept to demonstrate the reliability and robustness of our workflow. We actively encourage the use of diverse molecular generation tools to further explore the generalizability of our pipeline across different feature construction strategies. In addition, we clearly state that HighRes_Builder is not intended as a replacement for the CCD method, but rather as an extension that enables molecules not contained in the CCD to be recognized by the structure prediction model. It should be noted that certain prediction failures also occur for noncanonical residues containing the ‘GGXGG’ motif. As shown in Fig. 8, while the predicted monomer of the noncanonical residue is chemically plausible, its conformation within the peptide context becomes implausible. Such cases typically arise for residues with cyclized backbones or overly complex structures, which may be attributed to limitations in the predictive capability of the underlying structure prediction model. For instance, AlphaFold3 has been reported to exhibit suboptimal performance in predicting chirality or aromatic ring geometries in certain contexts.

**Table 1:** The structure comparison between CCD-based and our methods.

id	RMSD_all_atom	id	RMSD_all_atom
CHEMBL285843	1.054	CHEMBL320227	1.028
CHEMBL3245365	0.694	CHEMBL3808609	0.631
CHEMBL3397516	1.326	CHEMBL4241273	1.122
CHEMBL295666	0.923	CHEMBL1230248	1.185
CHEMBL300672	1.383	CHEMBL80257	1.178
CHEMBL238496	0.371	CHEMBL493596	0.703
CHEMBL251433	1.080	CHEMBL274323	0.895
CHEMBL442895	0.766	CHEMBL94390	1.336
CHEMBL2008002	0.831	CHEMBL1437221	1.206
CHEMBL2305085	0.181	CHEMBL469662	1.199
CHEMBL1230498	0.701	CHEMBL291278	0.871
CHEMBL1486321	1.054	CHEMBL4172293	0.179
CHEMBL1359836	1.102	CHEMBL55242	0.939
CHEMBL66105	1.225	CHEMBL11722	1.071
CHEMBL5177751	1.018	CHEMBL2106977	0.817
CHEMBL1099168	0.599	CHEMBL201657	0.513
CHEMBL13239	0.429	CHEMBL2004260	1.185
CHEMBL577	1.274	CHEMBL452715	0.291
CHEMBL319354	0.364	CHEMBL1231495	0.319
CHEMBL1171434	0.584	CHEMBL60475	0.955
CHEMBL217407	1.068	CHEMBL366222	1.506
CHEMBL296408	1.230	CHEMBL1165239	1.000
CHEMBL1977283	0.942	CHEMBL484901	1.446
CHEMBL1234201	0.467	CHEMBL1099167	1.288
CHEMBL121915	0.513	CHEMBL1256517	1.104

### Structure prediction and mechanism analysis of the peptide drugs containing noncanonical residues

#### Structure prediction of the linear peptide drug Relamorelin

Relamorelin is a novel synthetic peptide drug belonging to the class of ghrelin mimetics [29, 30]. Developed by Allergan, it is specifically indicated for the treatment of gastrointestinal motility disorders. Compared to natural ghrelin, Relamorelin exhibits enhanced receptor affinity, greater plasma stability, and a prolonged half-life. It effectively activates the GHS-1a receptor (also known as the ghrelin receptor), thereby stimulating growth hormone secretion and exerting multiple regulatory effects on gastrointestinal function. Key mechanisms of action include the enhancement of gastrointestinal motility, acceleration of gastric emptying, and promotion of colonic transit. As such, Relamorelin is classified as a prokinetic agent.

**Figure 9:**
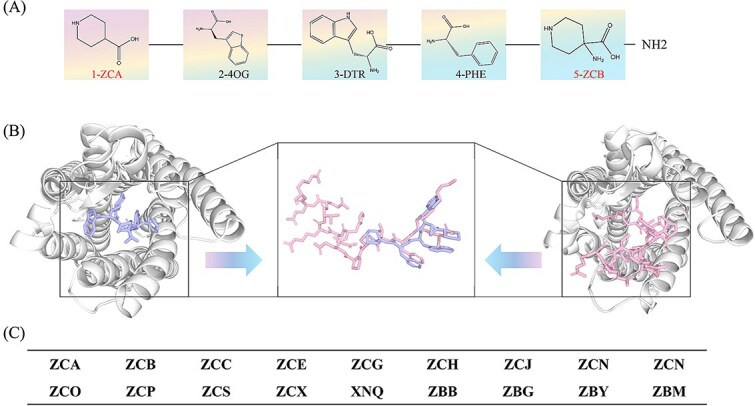
Structure prediction of Relamorelin. (A) Amino acid sequence of Relamorelin with residue identifiers queried and constructed by HighRes_Builder. (B) Structural comparison between the Relamorelin complex predicted by AlphaFold3 and the experimentally determined ghrelin complex (PDB ID: 7F9Y). (C) The set of three-letter codes representing residues absent from the CCD.

However, the three-dimensional structure of this peptide in complex with the ghrelin receptor has not been deposited in the PDB. In this study, we employed our HighRes_Builder platform to investigate the amino acid composition of this peptide drug. Three of the five amino acids were directly retrieved from the CCD database, while the remaining two residues were generated using our computational approach. It should be noted that the default three-letter code for residues not present in the CCD is ‘ZCA’. However, given that a peptide may contain multiple nonstandard residues, a table of three-letter codes not adopted in the CCD is provided in Fig. 9C to facilitate the naming of diverse amino acid molecules.

As illustrated in Fig. 9A, the two engineered residues in Relamorelin are assigned the identifiers ZCA and ZCB. Figure 9B presents a comparative structural analysis: the left panel depicts the predicted binding mode of Relamorelin with the ghrelin receptor, while the right panel shows the experimentally resolved ghrelin-receptor complex [31] (PDB: 7F9Y). Structurally, Relamorelin occupies the same characteristic barrel-shaped binding pocket, formed by alpha-helical segments of the receptor, as observed in the native ghrelin complex. The vast majority of the ligand’s conformation is embedded within this pocket, reflecting a deeply buried binding mode.

A detailed structural alignment reveals that the backbone of Relamorelin closely mirrors that of ghrelin, a similarity likely imposed by the sterically constrained nature of the pocket. Notably, the side-chain orientations in Relamorelin also recapitulate those of ghrelin, supporting a conserved binding topology. Further analysis identifies specific interactions that stabilize the complex: the amide group in the piperidine ring of residue ZCA forms a hydrogen bond with the receptor, while its aromatic group such as 1H-indole moiety engages in hydrophobic contacts.

Notably, Relamorelin is structurally smaller than ghrelin, a feature that likely facilitates deeper penetration into the binding pocket. This reduction in molecular size, while preserving key interaction features, may underlie the enhanced receptor binding affinity observed for Relamorelin compared to the native peptide.

#### Structure prediction of the cyclic peptide drug LUNA 18 and JNJ-77242113

Upon validating the feasibility of our method for linear peptides, we proceeded to extend its application to cyclic peptide construction. The structural prediction was performed using our AlphaFold3-based framework, HighFold3, which has been specifically adapted for modeling cyclic peptide architectures. Given that the predominant cyclization modalities for cyclic peptides are amide bond formation and disulfide bridging, we selected two representative drug molecules exemplifying these respective cyclization types for subsequent analysis.

LUNA18 [32, 33] is an orally bioavailable cyclic peptide that functions as a dual inhibitor of KRAS and ERK signaling. It effectively suppresses phosphorylation of ERK and AKT in RAS-mutant cancer cells [34], leading to inhibition of cell proliferation. In vivo, LUNA18 demonstrates sustained RAS pathway suppression and potent antitumor activity in mouse xenograft models by disrupting the interaction between RAS and guanine nucleotide exchange factors (GEFs). The compound exhibits marked cellular efficacy across multiple KRAS-altered cancer cell lines, including those derived from colorectal, gastric, nonsmall cell lung (NSCLC), and pancreatic cancers. Preclinical evaluations indicate that LUNA18 potently inhibits various KRAS mutants, such as G12D, G12V, and G12C. Significant antitumor activity has been observed in mutant NSCLC (NCI-H2122), pancreatic (NCI-H441), and gastric (GSU) cancer models.

**Figure 10:**
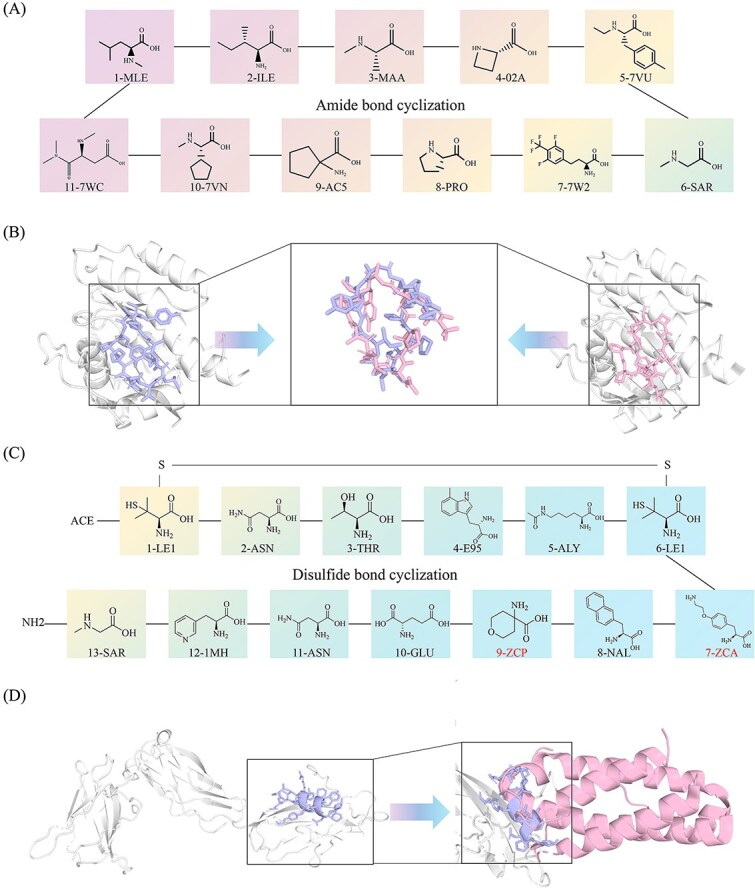
Structure prediction of cyclic peptides. (A) Amino acid sequence of LUNA18 with residue identifiers queried by HighRes_Builder. (B) Structural comparison between the LUNA18 complex predicted by AlphaFold3 and its corresponding experimentally determined complex (PDB ID: 7YV1). (C) Amino acid sequence of JNJ-77242113 with residue identifiers queried and constructed by HighRes_Builder. (D) Structural comparison between the JNJ-77242113 complex predicted by AlphaFold3 and the experimentally determined IL-23:IL-23R complex (PDB ID: 5MZV).

The residue composition of LUNA18 is shown in Fig. 10A. All constituent residues can be retrieved using our HighRes_Builder platform. We then employed HighFold3 to predict the structure of LUNA18 and compared it with the experimentally determined LUNA18 complex [35] (PDB ID: 7YV1), as shown in Fig. 10B. The predicted model accurately localized LUNA18 to the correct site on the KRAS protein, consistent with the reference structure 7YV1. A detailed structural alignment further confirms the high accuracy of the prediction. The Cα atom RMSD between the predicted and experimental structures is 2.373 Å, indicating near-perfect backbone geometry. Such low deviation value demonstrates the capability of HighFold3 to reproduce biologically relevant conformations of peptide-target complexes with near-experimental precision.

As a representative example of disulfide-cyclized peptide drugs, we have selected the recently acclaimed cyclic peptide JNJ-77242113 [36]. JNJ-77242113 is a first-in-class, orally administered IL-23 [37–39] receptor antagonist co-developed by Protagonist Therapeutics and Johnson & Johnson, offering a novel therapeutic approach for immune-mediated diseases. Its mechanism targets a well-established pathogenic pathway in psoriasis (PsO) and psoriatic arthritis (PsA): tissue injury triggers myeloid cells to release IL-23, which amplifies inflammation via the Th17/IL-17 axis [40]. By binding with high affinity to the IL-23 receptor, JNJ-77242113 selectively blocks this signaling cascade, inhibiting downstream Th17 cell activation and IL-17A release without interfering with the IL-12 pathway.

Preclinical studies demonstrated favorable pharmacokinetics and oral bioavailability for JNJ-77242113, properties later confirmed in healthy volunteers, where the peptide achieved therapeutic plasma levels and exhibited target engagement. Clinical development in moderate-to-severe plaque psoriasis has further substantiated its promise. In the Phase III ICONIC-LEAD trial, ~65% of patients achieved an IGA score of 0 or 1 (clear or almost clear skin), and ~50% attained PASI-90 after 16 weeks of treatment, results that were significantly superior to placebo. Additionally, the ICONIC-TOTAL study demonstrated efficacy in difficult-to-treat areas, including the scalp and nails [41].

However, the structural mechanism underlying the interaction between JNJ-77242113 and the IL-23 receptor remains unexplored, as no experimental structure of the complex is currently available. As illustrated in Fig. 10C, the peptide features a disulfide bond between the thiol groups of two 2-amino-3-mercapto-3-methylbutanoic acid residues, forming a six-membered ring that confers cyclization. Using the HighRes_Builder tool, we identified nine amino acids from the CCD database and generated two non-natural residue entries, designated ZCA and ZCP, based on our computational protocol.

Figure 10D presents the predicted structure of the JNJ-77242113:IL-23R complex (left) alongside the experimentally determined IL-23:IL-23R complex (right) [39]. Notably, JNJ-77242113 occupies the binding site of native IL-23, providing a structural basis for its ability to disrupt the IL-23/IL-23R interaction and inhibit downstream signaling. Interestingly, the predicted structure of JNJ-77242113 adopts a conformation featuring two alpha-helices similar to the secondary structure of IL-23 itself. One of these helices is stabilized by the disulfide-bridged cyclic scaffold. Additionally, multiple hydrogen bonds are formed between side-chain atoms of the peptide and residues within the beta-sheet-rich binding pocket of the receptor. The overall secondary structure of JNJ-77242113 closely mimics that of the native IL-23 helix region, which may partly explain its high binding affinity and potent protein–protein interaction (PPI) inhibitory activity.

## Discussion

Experimental results confirm that the proposed method maintains structural validity across diverse residue types and molecular weights, demonstrating particular robustness in handling side chains and medium-weight molecular scaffolds. This capability opens new avenues for accurate modeling of synthetic peptides and designed biomolecules, which are increasingly important in drug discovery and functional biomolecule design. We also use this method to explore the interaction mechanism for the current drugs that have recently attracted much attention such as JNJ-77242113 from structure perspective.

In summary, this work establishes an integrated computational framework combining our HighRes_Builder platform with noncanonical residue prediction such as HighFold3 and AlphaFold3 to enable accurate structure prediction of modified peptides. Conventional approaches are hindered by the limited coverage of noncanonical amino acids in the CCD database and the inefficiency of its default search tools, making structural modeling of specialized or modified peptides particularly challenging. Researchers often expend considerable effort manually preparing input files to meet the requirements of prediction algorithms, a process that is both time-consuming and tedious. Our method addresses these limitations by providing an efficient and automated solution, thereby accelerating progress in the field of peptide-based drug discovery and structural bioinformatics.

With improved access to predicted structures containing noncanonical residues, AI-based models can be more effectively utilized for drug discovery tasks such as virtual screening or interaction mechanism studies. For example, as reported by Hwang et al. [42], the noncanonical acetylation of methylenetetrahydrofolate dehydrogenase 2 (MTHFD2) at Lysine 44 (K44) by dihydrolipoyl transacetylase (DLAT) is a critical driver of chemotherapy resistance. While modeling such post-translational modifications is challenging for standard algorithms, our platform successfully h acetylated MTHFD2 (Fig. S1), providing structural insights into how this modification stabilizes the enzyme’s active site. Furthermore, our approach is applicable to AlphaFold-based screening workflows that involve noncanonical residues. One such application is the Short Linear Motif screening framework established by Stuke and Hummer [43], which introduced phosphomimetic mutations (S/T to E) at experimentally confirmed phosphorylation sites for comparison with the wild type. In contrast, our method enables the quick search and direct prediction of native phosphosite structures, as illustrated in Fig. S2.

Notably, as AlphaFold3 has established a pioneering framework for predicting non-natural amino acids, many contemporary open-source models, such as Boltz [7, 20] and Protenix [5], have adopted its architectural principles. Consequently, the residue definitions and representation formats generated by our pipeline are inherently compatible with these AlphaFold3-based ecosystems, ensuring broad applicability across prevalent workflows. For models utilizing different input protocols, we provide our AlphaFold3-based scripts in our GitHub repository to facilitate necessary feature modifications, thereby maximizing reproducibility and ease of use in peptide-based drug discovery.

In addition, our curated dataset is intentionally scoped to peptide-embeddable noncanonical amino-acid monomers rather than ‘all noncanonical residues’ in the broad chemical sense, and this scope choice inevitably introduces selection bias that we now discuss explicitly. Operationally, we include alpha/beta/gamma amino-acid-like monomers that preserve a free amine and a terminal carboxyl group connected by a short aliphatic backbone, which covers many biologically relevant noncanonical residues used in peptide/protein contexts (e.g. canonical-like side-chain substitutions such as alkyl/aryl substitutions, halogenation, hydroxylation, thioether/selenide variants, and other single-residue modifications that still form standard peptide bonds). In contrast, we exclude chemotypes that are not naturally representable as a single peptide residue definition or that fall outside robust residue-definition construction, including peptides/oligomers (multiple amide bonds), masked acids (esters/carbonates typical of protected intermediates or prodrugs), multi-acid species beyond the configured limit, and metal/coordination complexes or highly charged species (Figs S3 and S4). These rules bias the dataset toward monomeric, residue-definition-friendly chemistry and away from certain biologically important but structurally complex regimes, such as crosslinked residues, macrocycles, nonamide linkages, or highly flexible/protected intermediates, unless they can be reduced to a single-residue representation. We therefore emphasize that the dataset is designed to stress-test HighRes_Builder in the regime most relevant to residue insertion into peptide backbones, while coverage of more complex post-translationally modified or multiresidue motifs is a limitation and a natural direction for future extension.

It is important to note that the core contribution of our work lies in establishing a pipeline that integrates user-defined noncanonical amino acids with structure prediction models. Two key aspects of this pipeline warrant further clarification. First, regarding the generation of molecular structures for noncanonical residues: as a proof of concept, we employed RDKit, a widely adopted toolkit in contemporary noncanonical amino acid prediction workflows, to ensure robustness and consistency. It should be emphasized, however, that our framework is not restricted to this specific tool. The primary focus of our method is to enable structure prediction models to recognize and process custom noncanonical residue features, rather than to prescribe a particular molecular generation strategy. In principle, any reasonably generated molecular conformation can be adapted for use within our pipeline, thereby facilitating structure prediction for residue designs. Second, the plausibility and accuracy of the final predicted conformations depend critically on the performance of the underlying structure prediction model employed. Users are therefore advised to evaluate the suitability of their chosen model for specific application scenarios.

## Limitation

While this study demonstrates the feasibility of an automated pipeline for residue molecular representation, several limitations should be acknowledged. First, we observed that the performance is intrinsically linked to the foundational structure prediction model (AlphaFold3). For instance, the model occasionally makes chirality errors, limitations also noted in other studies and possibly attributable to its current architectural design. Future iterations of our pipeline will directly benefit from advances in these underlying models.

Second, the current proof-of-concept implementation utilizes RDKit for conformation generation due to its reliability and ease of integration. The modular design of our framework, however, is intended to be agnostic to the specific conformation engine. We envision that incorporating more advanced generators (e.g. deep learning-based or quantum chemistry methods) in future work could enhance the structural diversity and accuracy of the outputs.

Finally, the current scope of the platform is intentionally focused on amino acid-like molecules, reflecting the encoding paradigm of contemporary protein structure prediction models. This design choice establishes a clear baseline. A natural and important extension will be to adapt the framework for general small molecule ligands, which will require integrating specialized molecular encodings and engaging with broader featurization schemes. In the future, we aim to develop a more generalized molecular modeling platform that unifies the representation of residues and small molecules, thereby supporting a wider spectrum of drug discovery and structural bioinformatics applications.

## Conclusion

In this work, we have developed a user-friendly web platform that streamlines the preparation of nonstandard residue inputs for structure prediction systems such as AlphaFold3. By integrating the efficient CCD database and an automated residue representation generation pipeline, the platform effectively addresses the long-standing challenge of modeling non-natural amino acids and chemically modified peptides for structure prediction. Our approach not only reduces dependency on manual CCD curation but also lowers the technical barrier for researchers working with custom-designed molecular entities.

Looking forward, we believe this work lays a foundation for more accessible and scalable structure prediction workflows. By bridging the gap between experimental design and computational execution, our platform can help expand the application of AI-based structural modeling to a broader community. Future efforts will focus on extending the chemical diversity of supported residues, integrating with more prediction backends, and supporting dynamic user-defined molecular parametrization. We expect that such tools will play an increasingly critical role in accelerating the design and validation of novel bioactive molecules.

**Key Points**

- This study presents a hybrid strategy that combines experimentally determined CCD topology template with RDKit-based coordinate generation to generate noncanonical amino acid information. The corresponding CCD module in AlphaFold3 was modified to accept this information, thereby improving the accessibility and modeling accuracy of noncanonical residues in protein structure prediction.
- A user-friendly online platform was also developed to facilitate tool access and operation, minimizing the need for specialized knowledge in chemistry or biology. This design accelerates the research process while reducing potential human error.
- By integrating HighRes_Builder with structure prediction systems, using AlphaFold3 for linear peptides and HighFold3 for cyclic peptides, the study successfully modeled the linear peptide drug Relamorelin and the cyclic therapeutic peptides LUNA18 and JNJ-77242113 in complex with their target proteins. The results elucidate key structural determinants of their mechanisms of action, demonstrating the potential of this approach to accelerate research into peptide-based therapeutics.

## Supplementary Material

supporting_information_bbag272

## Data Availability

All data that this work uses are available in the Supporting Information.

## Code availability

The web of this work is available at http://hreb.duanlab.ac/. and the code is available at https://github.com/hongliangduan/HighRes_Builder.
