# Supplementary material for: HighRes_Builder: improved access and modeling of noncanonical residues for protein structure prediction
Source: Brief Bioinform. 2026 May 31;27(3):bbag272. doi: 10.1093/bib/bbag272 (PMC13222519; doi:10.1093/bib/bbag272)
Supplement: supporting_information_bbag272 [file supporting_information_bbag272.docx]

Supporting Information

**HighRes_Builder: Improved Access and Modeling of Noncanonical Residues for Protein Structure Prediction**

Yanchao Han^1^, Jianfeng Mei^1^, Gaoshuai Li^1^, Enkang Dai^1^, Hanlei Lu^1^, Chengyun Zhang^2^, Yanlu Zhang^1^, Chenshui Lin^1^, Chuanlong Zeng^3^, Hongliang Duan^2^ and Xudong Wang^1, *^.

^1^College of Pharmaceutical Sciences, Zhejiang University of Technology, Hangzhou, 310014, China.

^2^Faculty of Applied Sciences, Macao Polytechnic University, Macao, 999078, China.

^3^University of Chinese Academy of Sciences, Beijing, 100049, China.

^*^Address correspondence to: xdwang2019@zjut.edu.cn

Supplementary Algorithm 1. ExtendUserCCDFiles for accepting user-defined CCD files.

| **Algorithm** 1 ExtendUserCCDFiles |
| --- |
| from alphafold3.constants.residue_names import **CCD_NAME_TO_ONE_LETTER**  **def** ExtendUserCCDFiles(input_user_ccd, all_ccd):   1. user_ccd_files=[] 2. **if** input_user_ccd.is_file() **then** 3. user_ccd_files.append(input_user_ccd) 4. **else if** input_user_ccd.is_dir() **then** 5. user_ccd_files= **Path**(input_user_ccd).**glob**(*.) 6. **end if** 7. **for** user_ccd_file **∈** user_ccd_files **do** 8. appix_dict = {} 9. ccd_name=’’ 10. **if** user_ccd_file suffix with ***.cif*** **then** 11. appix_dict=AlphaFold3MMcifParser(user_ccd_file) 12. ccd_name=appix_dict.keys()[0] 13. **else if** user_ccd_file suffix with ***.csv*** **then** 14. appix_dict=ReadCsvFileAsDict(user_ccd_file) 15. ccd_name=ccd_data.pop(‘key’) 16. **end if** 17. **if** ccd_name not in **CCD_NAME_TO_ONE_LETTER then** 18. **CCD_NAME_TO_ONE_LETTER**[ccd_name]**=**’X’ 19. **end if** 20. all_ccd.update(appix_dict) 21. **return** all_ccd |

TableS1. the detailed information about our dataset.

| CHEMBL_ID | SMILES | Class | cLogP | MolWt | has_aromatic | has_chirality |
| --- | --- | --- | --- | --- | --- | --- |
| CHEMBL10047 | CCCCCc1c(O)noc1CC(N)C(=O)O | alpha | 1.0673 | 242.275 | TRUE | FALSE |
| CHEMBL101269 | O=C(O)C1CN(c2ccccc2P(=O)(O)O)CCN1 | alpha | -0.6476 | 286.224 | TRUE | FALSE |
| CHEMBL101713 | NC1=NC[C@@H](C[C@H](N)C(=O)O)C1 | alpha | -0.8345 | 171.2 | FALSE | TRUE |
| CHEMBL101926 | N/C(=N\CCC[C@@H](N)C(=O)O)NCc1ncc[nH]1 | alpha | -0.9939 | 254.294 | TRUE | TRUE |
| CHEMBL101999 | CC(=N)N1CC[C@H](C[C@H](N)C(=O)O)C1 | alpha | 0.10747 | 199.254 | FALSE | TRUE |
| CHEMBL102053 | CC(=N)N1CC[C@@H](C[C@H](N)C(=O)O)C1 | alpha | 0.10747 | 199.254 | FALSE | TRUE |
| CHEMBL10339 | CCCCc1c(O)noc1CC(N)C(=O)O | alpha | 0.6772 | 228.248 | TRUE | FALSE |
| CHEMBL103896 | N/C(=N\CCC[C@@H](N)C(=O)O)NCCc1ncc[nH]1 | alpha | -0.9514 | 268.321 | TRUE | TRUE |
| CHEMBL104039 | Cc1cnc(CC(N)C(=O)O)n1O | alpha | -0.61678 | 185.183 | TRUE | FALSE |
| CHEMBL104065 | Cc1ncc(CC(N)C(=O)O)n1O | alpha | -0.61678 | 185.183 | TRUE | FALSE |
| CHEMBL104197 | NC(Cc1nccn1O)C(=O)O | alpha | -0.9252 | 171.156 | TRUE | FALSE |
| CHEMBL104208 | Cc1nnn(O)c1CC(N)C(=O)O | alpha | -1.22178 | 186.171 | TRUE | FALSE |
| CHEMBL105983 | Cc1cc(C[C@H](N)C(=O)O)cc(C)c1Oc1ccc(O)c(C(C)C)c1 | alpha | 3.87914 | 343.423 | TRUE | TRUE |
| CHEMBL106423 | C=CCCNC(=N)NCCC[C@H](N)C(=O)O | alpha | -0.13143 | 228.296 | FALSE | TRUE |
| CHEMBL106820 | C=CCC(=N)NCCC[C@H](N)C(=O)O | alpha | 0.32147 | 199.254 | FALSE | TRUE |
| CHEMBL106871 | CCSC(=N)NCCC[C@H](N)C(=O)O | alpha | 0.45597 | 219.31 | FALSE | TRUE |
| CHEMBL107160 | Cc1cnn(O)c1CC(N)C(=O)O | alpha | -0.61678 | 185.183 | TRUE | FALSE |
| CHEMBL107509 | N/C(=N/CCCCC(N)C(=O)O)NO | alpha | -1.1379 | 204.23 | FALSE | FALSE |
| CHEMBL107660 | N[C@H](C[C@H]1CCCC[C@H]1CCP(=O)(O)O)C(=O)O | alpha | 1.1626 | 279.273 | FALSE | TRUE |
| CHEMBL1076865 | N[C@@H](CCP(=O)(O)CC(Cl)C(=O)O)C(=O)O | alpha | -0.2492 | 273.609 | FALSE | TRUE |
| CHEMBL107768 | Cc1onc(O)c1CCC(N)C(=O)O | alpha | 0.03312 | 200.194 | TRUE | FALSE |
| CHEMBL1078037 | N=C(N)NCCC[C@H](N[N+](=O)[O-])C(=O)O | alpha | -1.51583 | 219.201 | FALSE | TRUE |
| CHEMBL107813 | NC(Cc1ccccc1O)C(=O)O | alpha | 0.3466 | 181.191 | TRUE | FALSE |
| CHEMBL10787 | O=C(O)C1CN=C(c2ccccc2O)N1 | alpha | 0.1952 | 206.201 | TRUE | FALSE |
| CHEMBL107961 | N[C@@H](C[C@@H]1CCCC[C@@H]1CCP(=O)(O)O)C(=O)O | alpha | 1.1626 | 279.273 | FALSE | TRUE |
| CHEMBL108048 | CC1=C(C)C[C@@H](C(N)C(=O)O)[C@H](C(=O)O)C1 | alpha | 0.8455 | 227.26 | FALSE | TRUE |
| CHEMBL108096 | N[C@H](C[C@@H]1CCCC[C@@H]1CCP(=O)(O)O)C(=O)O | alpha | 1.1626 | 279.273 | FALSE | TRUE |
| CHEMBL108606 | CC(C)CCC(NC(C)C(=O)N1CCCC1C(=O)O)C(=O)O | alpha | 0.9295 | 314.382 | FALSE | FALSE |
| CHEMBL108823 | N[C@@H](C[C@H]1CCCC[C@H]1CCP(=O)(O)O)C(=O)O | alpha | 1.1626 | 279.273 | FALSE | TRUE |
| CHEMBL1088986 | NC(C(=O)O)c1ccc(-c2cccs2)s1 | alpha | 2.561 | 239.321 | TRUE | FALSE |
| CHEMBL1089174 | N[C@@H](CCC[PH](=O)O)C(=O)O | alpha | -0.3545 | 181.128 | FALSE | TRUE |
| CHEMBL1089175 | N[C@@H](CCCP(=O)(O)CCC(=O)O)C(=O)O | alpha | -0.0764 | 253.191 | FALSE | TRUE |
| CHEMBL1089311 | NC(C(=O)O)c1c[nH]cn1 | alpha | -0.5059 | 141.13 | TRUE | FALSE |
| CHEMBL1089312 | NC(C(=O)O)c1ccc(Br)s1 | alpha | 1.595 | 236.09 | TRUE | FALSE |
| CHEMBL1089320 | N[C@H](C(=O)O)c1ccc(S(=O)(=O)O)s1 | alpha | 0.0792 | 237.258 | TRUE | TRUE |
| CHEMBL1089514 | N[C@H](CCP(=O)(O)CCC(=O)O)C(=O)O | alpha | -0.4665 | 239.164 | FALSE | TRUE |
| CHEMBL1089515 | N[C@@H](CCP(=O)(O)CCC(=O)O)C(=O)O | alpha | -0.4665 | 239.164 | FALSE | TRUE |
| CHEMBL1089851 | NC(CP(=O)(O)CCC(=O)O)C(=O)O | alpha | -0.8566 | 225.137 | FALSE | FALSE |
| CHEMBL1089852 | N[C@@H](CCP(=O)(O)CC(=O)O)C(=O)O | alpha | -0.8566 | 225.137 | FALSE | TRUE |
| CHEMBL109037 | NC(Cc1cc(O)c(O)cc1O)C(=O)O | alpha | -0.2422 | 213.189 | TRUE | FALSE |
| CHEMBL1091143 | N[C@H](Cc1ccc(S(=O)(=O)O)cc1)C(=O)O | alpha | -0.1123 | 245.256 | TRUE | TRUE |
| CHEMBL1091144 | NC(Cc1ccc(Br)cc1S(=O)(=O)O)C(=O)O | alpha | 0.6502 | 324.152 | TRUE | FALSE |
| CHEMBL109133 | NC(Cc1ccc([N+](=O)[O-])c(O)c1)C(=O)O | alpha | 0.2548 | 226.188 | TRUE | FALSE |
| CHEMBL109169 | N[C@H](C(=O)O)c1ccc(P(=O)(O)O)cc1 | alpha | -0.426 | 231.144 | TRUE | TRUE |
| CHEMBL1091865 | Cc1cc(C(N)C(=O)O)sc1C | alpha | 1.44934 | 185.248 | TRUE | FALSE |
| CHEMBL1092198 | NC(C(=O)O)c1ccc(-c2ccc(C(=O)O)cc2)cc1 | alpha | 2.1362 | 271.272 | TRUE | FALSE |
| CHEMBL1092199 | NC(C(=O)O)c1csc2ccccc12 | alpha | 1.9857 | 207.254 | TRUE | FALSE |
| CHEMBL1092200 | N[C@@H](Cc1ccc(S(=O)(=O)O)cc1)C(=O)O | alpha | -0.1123 | 245.256 | TRUE | TRUE |
| CHEMBL1092212 | N[C@@H](C(=O)O)c1ccc(S(=O)(=O)O)s1 | alpha | 0.0792 | 237.258 | TRUE | TRUE |
| CHEMBL1092213 | NC(C(=O)O)c1ccc(S(=O)(=O)O)s1 | alpha | 0.0792 | 237.258 | TRUE | FALSE |
| CHEMBL1092242 | NC(CCP(=O)(O)CCCCC(=O)O)C(=O)O | alpha | 0.3137 | 267.218 | FALSE | FALSE |
| CHEMBL1092243 | N[C@@H](CCP(=O)(O)/C=C/C(=O)O)C(=O)O | alpha | -0.3429 | 237.148 | FALSE | TRUE |
| CHEMBL1092311 | CC(CP(=O)(O)CC[C@H](N)C(=O)O)C(=O)O | alpha | -0.2205 | 253.191 | FALSE | TRUE |
| CHEMBL1092727 | NC(CCP(=O)(O)CCC(=O)O)C(=O)O | alpha | -0.4665 | 239.164 | FALSE | FALSE |
| CHEMBL1093009 | N[C@@H](CCP(=O)(O)CCCC(=O)O)C(=O)O | alpha | -0.0764 | 253.191 | FALSE | TRUE |
| CHEMBL1093447 | N[C@@H](C(=O)O)c1ccc(S(=O)(=O)O)cc1 | alpha | 0.0177 | 231.229 | TRUE | TRUE |
| CHEMBL1093798 | N[C@H](C(=O)O)c1ccc(S(=O)(=O)O)cc1 | alpha | 0.0177 | 231.229 | TRUE | TRUE |
| CHEMBL1094129 | NC(Cc1nonc1O)C(=O)O | alpha | -1.2704 | 173.128 | TRUE | FALSE |
| CHEMBL109460 | NC(Cc1nc2ccccc2[nH]1)C(=O)O | alpha | 0.5173 | 205.217 | TRUE | FALSE |
| CHEMBL1094977 | NC(Cc1ccc(O)n[n+]1[O-])C(=O)O | alpha | -1.625 | 199.166 | TRUE | FALSE |
| CHEMBL1098101 | NC(CCc1nonc1O)C(=O)O | alpha | -0.8803 | 187.155 | TRUE | FALSE |
| CHEMBL1098104 | NC(CCCc1nonc1O)C(=O)O | alpha | -0.4902 | 201.182 | TRUE | FALSE |
| CHEMBL1099097 | NC(C(=O)O)c1nonc1O | alpha | -1.1404 | 159.101 | TRUE | FALSE |
| CHEMBL1099169 | Nc1nccn1CCC[C@H](N)C(=O)O | alpha | -0.3426 | 198.226 | TRUE | TRUE |
| CHEMBL1099170 | Nc1ncc(CCCC(N)C(=O)O)[nH]1 | alpha | -0.2735 | 198.226 | TRUE | FALSE |
| CHEMBL1099171 | N[C@@H](CCCNc1ncc[nH]1)C(=O)O | alpha | 0.0137 | 198.226 | TRUE | TRUE |
| CHEMBL110016 | NC(Cc1nc2c([nH]1)CCCC2)C(=O)O | alpha | 0.2429 | 209.249 | TRUE | FALSE |
| CHEMBL110335 | Cc1cc(C[C@H](N)C(=O)O)cc(C)c1Cc1ccc(O)c(C(C)C)c1 | alpha | 3.67764 | 341.451 | TRUE | TRUE |
| CHEMBL110357 | NC(Cc1cccc([N+](=O)[O-])c1O)C(=O)O | alpha | 0.2548 | 226.188 | TRUE | FALSE |
| CHEMBL111441 | Cc1onc(O)c1CCCCC(N)C(=O)O | alpha | 0.81332 | 228.248 | TRUE | FALSE |
| CHEMBL111651 | NC(Cc1c(O)noc1CBr)C(=O)O | alpha | 0.2295 | 265.063 | TRUE | FALSE |
| CHEMBL111716 | NC(Cc1cc([N+](=O)[O-])ccc1O)C(=O)O | alpha | 0.2548 | 226.188 | TRUE | FALSE |
| CHEMBL112402 | NC(Cc1c(O)noc1CO)C(=O)O | alpha | -1.1731 | 202.166 | TRUE | FALSE |
| CHEMBL112755 | CNC(C(=O)O)c1c(O)noc1C | alpha | 0.03372 | 186.167 | TRUE | FALSE |
| CHEMBL11371 | NC(Cc1[nH]cnc1[N+](=O)[O-])C(=O)O | alpha | -0.7277 | 200.154 | TRUE | FALSE |
| CHEMBL113829 | NC(Cc1nc2ccccc2nc1CCP(=O)(O)O)C(=O)O | alpha | 0.3044 | 325.261 | TRUE | FALSE |
| CHEMBL11393 | N[C@H](CCCCSc1cc(Cl)ccc1O)C(=O)O | alpha | 2.7199 | 289.784 | TRUE | TRUE |
| CHEMBL114315 | Cc1cc2nc(CC(N)C(=O)O)c(CP(=O)(O)O)nc2cc1C | alpha | 0.87874 | 339.288 | TRUE | FALSE |
| CHEMBL114551 | CC(=N)NCCSCC[C@H](N)C(=O)O | alpha | 0.10837 | 219.31 | FALSE | TRUE |
| CHEMBL115671 | NC(Cc1nc2ccc(Cl)cc2nc1CP(=O)(O)O)C(=O)O | alpha | 0.9153 | 345.679 | TRUE | FALSE |
| CHEMBL1159695 | NC(N)=NCCCC(NCc1c[nH]c2ccccc12)C(=O)O | alpha | 0.7643 | 303.366 | TRUE | FALSE |
| CHEMBL1159696 | NC(N)=NCCC[C@@H](NCc1ccc2ccccc2c1)C(=O)O | alpha | 1.4362 | 314.389 | TRUE | TRUE |
| CHEMBL1159698 | NC(N)=NCCC[C@H](NCc1ccc2ccccc2c1)C(=O)O | alpha | 1.4362 | 314.389 | TRUE | TRUE |
| CHEMBL1159704 | NC(N)=NCCC[C@@H](NCc1c[nH]c2ccccc12)C(=O)O | alpha | 0.7643 | 303.366 | TRUE | TRUE |
| CHEMBL1159706 | COc1ccc(CN[C@H](CCCN=C(N)N)C(=O)O)cc1OC | alpha | 0.3002 | 324.381 | TRUE | TRUE |
| CHEMBL1159707 | COc1ccc(CNC(CCCN=C(N)N)C(=O)O)cc1OC | alpha | 0.3002 | 324.381 | TRUE | FALSE |
| CHEMBL1159734 | CC(NP(=O)(O)C(C)N)C(=O)O | alpha | -0.4608 | 196.143 | FALSE | FALSE |
| CHEMBL1159758 | N=C(NCCC[C@H](N)C(=O)O)NC1CC1 | alpha | -0.54513 | 214.269 | FALSE | TRUE |
| CHEMBL1159826 | Cc1onc(O)c1Cn1oc(C)c(CC(N)C(=O)O)c1=O | alpha | -0.24556 | 297.267 | TRUE | FALSE |
| CHEMBL11599 | NC(CCCSc1cc(Cl)ccc1O)C(=O)O | alpha | 2.3298 | 275.757 | TRUE | FALSE |
| CHEMBL1159913 | NC(C/C=C(\c1cccnc1)c1ccc(F)cc1F)C(=O)O | alpha | 2.5935 | 304.296 | TRUE | FALSE |
| CHEMBL1159981 | CCC(CP(=O)(O)CC[C@H](N)C(=O)O)C(=O)O | alpha | 0.1696 | 267.218 | FALSE | TRUE |
| CHEMBL1159982 | CCC(CS(=O)(=O)CCC(N)C(=O)O)C(=O)O | alpha | -0.686 | 267.303 | FALSE | FALSE |
| CHEMBL1160289 | NCC(O)c1ccc(O)c2c1SCC(C(=O)O)N2 | alpha | 0.3551 | 270.31 | TRUE | FALSE |
| CHEMBL1160290 | NCC(O)c1cc(O)c2c(c1)SCC(C(=O)O)N2 | alpha | 0.3551 | 270.31 | TRUE | FALSE |
| CHEMBL1160345 | CCC(=O)SCC(C(=O)O)C(N)C(=O)O | alpha | -0.2311 | 235.261 | FALSE | FALSE |
| CHEMBL1160346 | CN(O)C(=O)SCC(C(=O)O)C(N)C(=O)O | alpha | -0.7267 | 252.248 | FALSE | FALSE |
| CHEMBL1160348 | C[C@@H](O)C(=O)SCC(C(=O)O)C(N)C(=O)O | alpha | -1.2603 | 251.26 | FALSE | TRUE |
| CHEMBL1160351 | CNC(=O)SCC(C(=O)O)C(N)C(=O)O | alpha | -0.8283 | 236.249 | FALSE | FALSE |
| CHEMBL11605 | NC(CCCCCSc1cc(Cl)ccc1O)C(=O)O | alpha | 3.11 | 303.811 | TRUE | FALSE |
| CHEMBL1160639 | CCOP(=O)(O)N[C@@H](CC(C)C)C(=O)O | alpha | 1.2122 | 239.208 | FALSE | TRUE |
| CHEMBL1160645 | CCOP(=O)(O)N[C@H](C(=O)O)[C@@H](C)CC | alpha | 1.2122 | 239.208 | FALSE | TRUE |
| CHEMBL1161513 | NC(CC1CCC(=O)C2OC12)C(=O)O | alpha | -0.4651 | 199.206 | FALSE | FALSE |
| CHEMBL1161638 | CN(CCCC(N)C(=O)O)C(=N)N | alpha | -0.99623 | 188.231 | FALSE | FALSE |
| CHEMBL1161818 | N[C@@H](C[C@]1(C(=O)O)Cc2ccccc2O1)C(=O)O.O=C(O)C(F)(F)F | alpha | 0.8802 | 365.26 | TRUE | TRUE |
| CHEMBL1161819 | N[C@@H](C[C@@]1(C(=O)O)Cc2ccccc2O1)C(=O)O.O=C(O)C(F)(F)F | alpha | 0.8802 | 365.26 | TRUE | TRUE |
| CHEMBL1161820 | Cc1ccc2c(c1)C[C@](C[C@H](N)C(=O)O)(C(=O)O)O2.O=C(O)C(F)(F)F | alpha | 1.18862 | 379.287 | TRUE | TRUE |
| CHEMBL1161821 | Cc1cccc2c1O[C@@](C[C@H](N)C(=O)O)(C(=O)O)C2.O=C(O)C(F)(F)F | alpha | 1.18862 | 379.287 | TRUE | TRUE |
| CHEMBL116299 | Cl.NC(CC[S+]([O-])CCC(=O)NCC(=O)O)C(=O)O | alpha | -1.4502 | 316.763 | FALSE | FALSE |
| CHEMBL11664 | Nc1ccccc1SCCCCCC(N)C(=O)O | alpha | 2.3332 | 268.382 | TRUE | FALSE |
| CHEMBL11665 | NC(CCCCCS(=O)(=O)c1ccccc1O)C(=O)O | alpha | 1.1382 | 301.364 | TRUE | FALSE |
| CHEMBL11666 | NC(CCCCC[S+]([O-])c1ccccc1O)C(=O)O | alpha | 1.4721 | 285.365 | TRUE | FALSE |
| CHEMBL116797 | NC(Cc1nc2cc(Cl)ccc2nc1CP(=O)(O)O)C(=O)O | alpha | 0.9153 | 345.679 | TRUE | FALSE |
| CHEMBL116811 | Cl.N[C@@H](CC[S+]([O-])Cc1ccc(C(=O)NCC(=O)O)cc1)C(=O)O | alpha | -0.0265 | 378.834 | TRUE | TRUE |
| CHEMBL116889 | O=C(O)C1CC2(CCCC(CP(=O)(O)O)C2)CCN1 | alpha | 1.1773 | 291.284 | FALSE | FALSE |
| CHEMBL1169530 | O=C(O)[C@@H]1C[C@@H](n2oc(=O)[nH]c2=O)CN1 | alpha | -1.8828 | 215.165 | TRUE | TRUE |
| CHEMBL1169531 | O=C(O)[C@@H]1C[C@H](n2oc(=O)[nH]c2=O)CN1 | alpha | -1.8828 | 215.165 | TRUE | TRUE |
| CHEMBL1173786 | NC(C(=O)O)c1ccsc1 | alpha | 0.8325 | 157.194 | TRUE | FALSE |
| CHEMBL117514 | NC(Cc1ccc(OCC(=O)O)cc1)C(=O)O | alpha | 0.1044 | 239.227 | TRUE | FALSE |
| CHEMBL1178552 | C[S+](CCCCc1c[nH]c2ccccc12)CCC(N)C(=O)O | alpha | 2.5407 | 321.466 | TRUE | FALSE |
| CHEMBL1179159 | N=C(N)c1ccc(C2=NOC(CC(=O)NC[C@H](N)C(=O)O)C2)cc1 | alpha | -0.61813 | 333.348 | TRUE | TRUE |
| CHEMBL1180190 | NC(CC[S+]([O-])CCC(=O)NCC(=O)O)C(=O)O | alpha | -1.872 | 280.302 | FALSE | FALSE |
| CHEMBL1180211 | N[C@@H](CC[S+]([O-])Cc1ccc(C(=O)NCC(=O)O)cc1)C(=O)O | alpha | -0.4483 | 342.373 | TRUE | TRUE |
| CHEMBL1180659 | Nc1ccccc1NC[C@H](N)C(=O)O | alpha | 0.0926 | 195.222 | TRUE | TRUE |
| CHEMBL1180686 | Nc1ccccc1NC[C@@H](N)C(=O)O | alpha | 0.0926 | 195.222 | TRUE | TRUE |
| CHEMBL1180714 | Cc1cc(N)c(NC[C@H](N)C(=O)O)cc1C | alpha | 0.70944 | 223.276 | TRUE | TRUE |
| CHEMBL1181550 | N[C@@H](CCSCCC(=O)NCC(=O)O)C(=O)O | alpha | -0.8875 | 264.303 | FALSE | TRUE |
| CHEMBL1182916 | CN/N=C(\N)NCCC[C@H](N)C(=O)O | alpha | -1.7828 | 203.246 | FALSE | TRUE |
| CHEMBL1182918 | N/C(=N/CCC[C@H](N)C(=O)O)N(N)CC(F)(F)F | alpha | -0.7688 | 271.243 | FALSE | TRUE |
| CHEMBL1183261 | C[S+](CCCc1c[nH]c2ccccc12)CCC(N)C(=O)O | alpha | 2.1506 | 307.439 | TRUE | FALSE |
| CHEMBL1184261 | Cc1cc(N)c(NC[C@@H](N)C(=O)O)cc1C | alpha | 0.70944 | 223.276 | TRUE | TRUE |
| CHEMBL118502 | C=C(CC)c1ccc(C(=C)[C@H]2CN[C@H](C(=O)O)[C@H]2CC(=O)O)cc1 | alpha | 2.8865 | 329.396 | TRUE | TRUE |
| CHEMBL1185292 | N/N=C(\N)NCCC[C@H](N)C(=O)O | alpha | -2.0435 | 189.219 | FALSE | TRUE |
| CHEMBL118537 | O=C(O)[C@@H]1C[C@H]2CC(CP(=O)(O)O)CC[C@H]2N1 | alpha | 0.3955 | 263.23 | FALSE | TRUE |
| CHEMBL11855 | CC(CO)[C@H](N)C(=O)O | alpha | -0.9733 | 133.147 | FALSE | TRUE |
| CHEMBL1186327 | N[C@@H](CCCN/C=C1\C(=O)Nc2ccccc21)C(=O)O | alpha | 0.7613 | 275.308 | TRUE | TRUE |
| CHEMBL1186331 | N[C@@H](CCCCN/C=C1\C(=O)Nc2ccccc21)C(=O)O | alpha | 1.1514 | 289.335 | TRUE | TRUE |
| CHEMBL118690 | CCN(CC)C(=O)[C@@]1(c2ccccc2)C[C@H]1C(N)C(=O)O | alpha | 1.2246 | 290.363 | TRUE | TRUE |
| CHEMBL1188075 | CC(=N)NCC(F)(F)CC[C@H](N)C(=O)O | alpha | 0.40057 | 223.223 | FALSE | TRUE |
| CHEMBL1188145 | N[C@@H](CCP(=O)(O)CO)C(=O)O | alpha | -0.9914 | 197.127 | FALSE | TRUE |
| CHEMBL1188310 | NCP(=O)(O)CC[C@H](N)C(=O)O | alpha | -1.025 | 196.143 | FALSE | TRUE |
| CHEMBL1188369 | CC(=N)NCC(F)(F)CCC(N)C(=O)O | alpha | 0.40057 | 223.223 | FALSE | FALSE |
| CHEMBL1189262 | NCCP(=O)(O)CC[C@H](N)C(=O)O | alpha | -0.9825 | 210.17 | FALSE | TRUE |
| CHEMBL1189353 | NC(CSc1cccc(O)c1O)C(=O)O | alpha | 0.6018 | 229.257 | TRUE | FALSE |
| CHEMBL1189517 | CNC(CN=C(N)N)C(=O)O | alpha | -2.0676 | 160.177 | FALSE | FALSE |
| CHEMBL1189573 | C[C@H](NCc1cc([N+](=O)[O-])cc2nc(O)c(O)nc12)C(=O)O | alpha | 0.5119 | 308.25 | TRUE | TRUE |
| CHEMBL1189735 | C[C@H](N)P(=O)(O)CC[C@H](N)C(=O)O | alpha | -0.6365 | 210.17 | FALSE | TRUE |
| CHEMBL1190802 | NC(CCCc1nnn[nH]1)C(=O)O | alpha | -1.0657 | 185.187 | TRUE | FALSE |
| CHEMBL1191183 | N[C@H]1CCN[C@H]1C(=O)O | alpha | -1.2398 | 130.147 | FALSE | TRUE |
| CHEMBL1191538 | O=C(O)C1NCC12CCCCC2 | alpha | 0.9933 | 169.224 | FALSE | FALSE |
| CHEMBL1192138 | NC(CS/C(S)=N/CCCc1ccccc1)C(=O)O | alpha | 2.0501 | 298.433 | TRUE | FALSE |
| CHEMBL1192177 | C[C@@H](NCc1cc([N+](=O)[O-])cc2nc(O)c(O)nc12)C(=O)O | alpha | 0.5119 | 308.25 | TRUE | TRUE |
| CHEMBL1193048 | NC(CCCCc1nnn[nH]1)C(=O)O | alpha | -0.6756 | 199.214 | TRUE | FALSE |
| CHEMBL1193049 | NC(CCCCCCc1nnn[nH]1)C(=O)O | alpha | 0.1046 | 227.268 | TRUE | FALSE |
| CHEMBL1193096 | CC(Nc1cccc(C(=O)c2ccccc2)c1)C(=O)O | alpha | 2.8026 | 269.3 | TRUE | FALSE |
| CHEMBL1193438 | C=C1CN[C@H](C(=O)O)C1 | alpha | -0.0109 | 127.143 | FALSE | TRUE |
| CHEMBL1193688 | NC(CCCCCCn1ccnc1)C(=O)O | alpha | 1.2455 | 225.292 | TRUE | FALSE |
| CHEMBL1193844 | Cc1cc2[nH]c(=O)c(=O)n(C[C@@H](N)C(=O)O)c2cc1C | alpha | -0.28146 | 277.28 | TRUE | TRUE |
| CHEMBL1194045 | Cc1cc2[nH]c(=O)c(=O)n(C[C@H](N)C(=O)O)c2cc1C | alpha | -0.28146 | 277.28 | TRUE | TRUE |
| CHEMBL1195124 | C[C@H](NCCc1cc([N+](=O)[O-])cc2nc(O)c(O)nc12)C(=O)O | alpha | 0.5544 | 322.277 | TRUE | TRUE |
| CHEMBL1195212 | NC(CS/C(S)=N/Cc1ccccc1)C(=O)O | alpha | 1.6175 | 270.379 | TRUE | FALSE |
| CHEMBL1195409 | CC[C@H](NCc1cc([N+](=O)[O-])cc2nc(O)c(O)nc12)C(=O)O | alpha | 0.902 | 322.277 | TRUE | TRUE |
| CHEMBL1195455 | C[C@H](NCc1cc(Br)cc2nc(O)c(O)nc12)C(=O)O | alpha | 1.3662 | 342.149 | TRUE | TRUE |
| CHEMBL119635 | N[C@@H](C(=O)O)C1C[C@@H]1C(=O)O | alpha | -0.881 | 159.141 | FALSE | TRUE |
| CHEMBL1196594 | C[C@@H](N)P(=O)(O)CC[C@H](N)C(=O)O | alpha | -0.6365 | 210.17 | FALSE | TRUE |
| CHEMBL1198742 | N[C@@H](Cn1c(=O)c(=O)[nH]c2ccccc21)C(=O)O | alpha | -0.8983 | 249.226 | TRUE | TRUE |
| CHEMBL119885 | NC(Cc1ccc(C(F)(F)C(=O)O)cc1)C(=O)O | alpha | 0.8174 | 259.208 | TRUE | FALSE |
| CHEMBL11996 | CC1(C)CC1C(=O)N/C(=C\CCCSCC(N)C(=O)O)C(=O)O | alpha | 1.0425 | 344.433 | FALSE | FALSE |
| CHEMBL1199778 | N[C@@H](C[C@]1(C(=O)O)Cc2ccccc2O1)C(=O)O | alpha | 0.2469 | 251.238 | TRUE | TRUE |
| CHEMBL1199779 | N[C@@H](C[C@@]1(C(=O)O)Cc2ccccc2O1)C(=O)O | alpha | 0.2469 | 251.238 | TRUE | TRUE |
| CHEMBL1199780 | Cc1ccc2c(c1)C[C@](C[C@H](N)C(=O)O)(C(=O)O)O2 | alpha | 0.55532 | 265.265 | TRUE | TRUE |
| CHEMBL119980 | CC12CC(CP(=O)(O)O)CCC1CNC(C(=O)O)C2 | alpha | 1.0332 | 291.284 | FALSE | FALSE |
| CHEMBL120003 | C=C(c1ccc(F)cc1)[C@H]1CN[C@H](C(=O)O)[C@H]1CC(=O)O | alpha | 1.6024 | 293.294 | TRUE | TRUE |
| CHEMBL1200167 | Cc1cccc2c1O[C@@](C[C@H](N)C(=O)O)(C(=O)O)C2 | alpha | 0.55532 | 265.265 | TRUE | TRUE |
| CHEMBL1200377 | CC(N)Cc1ccccc1.NC(CC(=O)O)C(=O)O | alpha | 0.4493 | 268.313 | TRUE | FALSE |
| CHEMBL120114 | COC[C@H]1[C@H](C(=O)O)[C@H]1[C@H](N)C(=O)O | alpha | -1.0085 | 203.194 | FALSE | TRUE |
| CHEMBL120288 | C=C(c1ccc(CCC)cc1)[C@H]1CN[C@H](C(=O)O)[C@H]1CC(=O)O | alpha | 2.4158 | 317.385 | TRUE | TRUE |
| CHEMBL1204440 | CP(O)(=S)N[C@@H](CCC(=O)O)C(=O)O | alpha | -0.1744 | 241.205 | FALSE | TRUE |
| CHEMBL1204441 | CCCCP(O)(=S)N[C@@H](CCC(=O)O)C(=O)O | alpha | 0.9959 | 283.286 | FALSE | TRUE |
| CHEMBL120450 | C=C(c1ccc(CCCC)cc1)[C@H]1CN[C@H](C(=O)O)[C@H]1CC(=O)O | alpha | 2.8059 | 331.412 | TRUE | TRUE |
| CHEMBL1204545 | O=C(O)CC[C@H](NP(O)(=S)c1ccccc1)C(=O)O | alpha | 0.5213 | 303.276 | TRUE | TRUE |
| CHEMBL1204546 | CCP(O)(=S)N[C@@H](CCC(=O)O)C(=O)O | alpha | 0.2157 | 255.232 | FALSE | TRUE |
| CHEMBL1204809 | NC(C/C=C(\c1ccsc1)c1ccc(F)cc1F)C(=O)O | alpha | 3.26 | 309.337 | TRUE | FALSE |
| CHEMBL1204821 | CC(C)c1cccc(/C(=C\CC(N)C(=O)O)c2ccc(F)cc2F)c1 | alpha | 4.3219 | 345.389 | TRUE | FALSE |
| CHEMBL1204823 | CC(C)c1ccc(/C(=C\CC(N)C(=O)O)c2cccnc2)cc1 | alpha | 3.4387 | 310.397 | TRUE | FALSE |
| CHEMBL1204825 | CC(C)c1ccc(/C(=C\C[C@H](N)C(=O)O)c2ccc(F)cc2F)cc1 | alpha | 4.3219 | 345.389 | TRUE | TRUE |
| CHEMBL1204826 | CCc1ccc(/C(=C\CC(N)C(=O)O)c2ccc(F)cc2F)cc1 | alpha | 3.7609 | 331.362 | TRUE | FALSE |
| CHEMBL1204831 | CC(C)c1ccc(/C(=C\CC(N)C(=O)O)c2ccc(F)cc2F)cc1 | alpha | 4.3219 | 345.389 | TRUE | FALSE |
| CHEMBL1204836 | Cc1ccc(/C(=C\CC(N)C(=O)O)c2ccc(F)cc2F)cc1 | alpha | 3.50692 | 317.335 | TRUE | FALSE |
| CHEMBL1204841 | CC(C)c1ccc(/C(=C\CC(N)C(=O)O)c2ccccc2F)cc1 | alpha | 4.1828 | 327.399 | TRUE | FALSE |
| CHEMBL1204842 | NC(C/C=C(\c1ccccc1)c1ccc(F)cc1F)C(=O)O | alpha | 3.1985 | 303.308 | TRUE | FALSE |
| CHEMBL1204843 | COc1ccc(/C(=C\CC(N)C(=O)O)c2ccc(F)cc2F)cc1 | alpha | 3.2071 | 333.334 | TRUE | FALSE |
| CHEMBL1204849 | CC(C)c1ccc(/C(=C\CC(N)C(=O)O)c2ccccc2)cc1 | alpha | 4.0437 | 309.409 | TRUE | FALSE |
| CHEMBL1204850 | COc1ccc(/C(=C/CC(N)C(=O)O)c2ccc(C(C)C)cc2)cc1 | alpha | 4.0523 | 339.435 | TRUE | FALSE |
| CHEMBL1205331 | NC(CC1CCC(CS(=O)(=O)O)CC1)C(=O)O | alpha | 0.4826 | 265.331 | FALSE | FALSE |
| CHEMBL1205335 | NC(Cc1ccccc1CS(=O)(=O)O)C(=O)O | alpha | 0.0288 | 259.283 | TRUE | FALSE |
| CHEMBL1205336 | NC(Cc1cccc(CS(=O)(=O)O)c1)C(=O)O | alpha | 0.0288 | 259.283 | TRUE | FALSE |
| CHEMBL120534 | NC(CC(O)c1cccc(O)c1)C(=O)O | alpha | 0.2276 | 211.217 | TRUE | FALSE |
| CHEMBL1205690 | CC(C)CC(NS(=O)(=O)c1ccccc1)C(=O)O | alpha | 1.4642 | 271.338 | TRUE | FALSE |
| CHEMBL1205693 | O=C(O)C(Cc1ccccc1)NS(=O)(=O)c1ccccc1 | alpha | 1.6608 | 305.355 | TRUE | FALSE |
| CHEMBL1205697 | O=C(O)C(CO)NS(=O)(=O)c1ccccc1 | alpha | -0.5896 | 245.256 | TRUE | FALSE |
| CHEMBL1205700 | NCCCCC(NS(=O)(=O)c1ccccc1)C(=O)O | alpha | 0.5471 | 286.353 | TRUE | FALSE |
| CHEMBL1205718 | CC(C)C(NS(=O)(=O)c1ccccc1)C(=O)O | alpha | 1.0741 | 257.311 | TRUE | FALSE |
| CHEMBL1206575 | CC(C)c1ccc(/C(=C\CC(N)C(=O)O)c2ccsc2)cc1 | alpha | 4.1052 | 315.438 | TRUE | FALSE |
| CHEMBL1206581 | CC(C)c1ccc(/C(=C\CC(N)C(=O)O)c2ccc(C#N)cc2)cc1 | alpha | 3.91538 | 334.419 | TRUE | FALSE |
| CHEMBL1206612 | N#Cc1ccc(/C(=C\CC(N)C(=O)O)c2ccc(F)cc2F)cc1 | alpha | 3.07018 | 328.318 | TRUE | FALSE |
| CHEMBL1206818 | NC(CC1CCCCC1CS(=O)(=O)O)C(=O)O | alpha | 0.4826 | 265.331 | FALSE | FALSE |
| CHEMBL120753 | C=C(C)c1ccc(C(=C)[C@H]2CN[C@H](C(=O)O)[C@H]2CC(=O)O)cc1 | alpha | 2.4964 | 315.369 | TRUE | TRUE |
| CHEMBL1207569 | CC(C)c1ccc(/C(=C/CC(N)C(=O)O)c2ccc(F)cc2F)cc1 | alpha | 4.3219 | 345.389 | TRUE | FALSE |
| CHEMBL1207611 | NC(CC1CCCC(CS(=O)(=O)O)C1)C(=O)O | alpha | 0.4826 | 265.331 | FALSE | FALSE |
| CHEMBL120785 | C=C(c1ccc(F)c(C)c1)[C@H]1CN[C@H](C(=O)O)[C@H]1CC(=O)O | alpha | 1.91082 | 307.321 | TRUE | TRUE |
| CHEMBL120808 | Cc1nn(C[C@H](N)C(=O)O)c(=O)[nH]c1=O | alpha | -2.34808 | 214.181 | TRUE | TRUE |
| CHEMBL1210425 | N[C@@H](CCC(=O)NNc1ccccc1O)C(=O)O | alpha | 0.0274 | 253.258 | TRUE | TRUE |
| CHEMBL1210426 | N[C@@H](CCC(=O)NNc1cccc(O)c1)C(=O)O | alpha | 0.0274 | 253.258 | TRUE | TRUE |
| CHEMBL1210427 | N[C@@H](CCC(=O)NNc1ccc(O)cc1)C(=O)O | alpha | 0.0274 | 253.258 | TRUE | TRUE |
| CHEMBL121194 | C=C(c1ccccc1)[C@@H]1CN[C@H](C(=O)O)[C@H]1CC(=O)O | alpha | 1.4633 | 275.304 | TRUE | TRUE |
| CHEMBL1213052 | O=C(O)c1ccccc1N[C@H](C(=O)O)c1ccccc1 | alpha | 2.6226 | 271.272 | TRUE | TRUE |
| CHEMBL1213195 | Cc1ccc(N[C@H](CC(=O)c2ccccc2)C(=O)O)cc1C | alpha | 3.44154 | 297.354 | TRUE | TRUE |
| CHEMBL121461 | C=C(c1cc(C)cc(C)c1)[C@H]1CN[C@H](C(=O)O)[C@H]1CC(=O)O | alpha | 2.08014 | 303.358 | TRUE | TRUE |
| CHEMBL121553 | N[C@@H](Cn1nc(I)c(=O)[nH]c1=O)C(=O)O | alpha | -2.0519 | 326.05 | TRUE | TRUE |
| CHEMBL121741 | C=C(c1ccccc1)[C@H]1CN[C@H](C(=O)O)[C@H]1CC(=O)O | alpha | 1.4633 | 275.304 | TRUE | TRUE |
| CHEMBL1221826 | N[C@@H](CC(=O)N[C@@H](CCC(=O)O)c1ccccc1)C(=O)O | alpha | 0.5107 | 294.307 | TRUE | TRUE |
| CHEMBL1221840 | C[C@H](NC=O)C(=O)O | alpha | -0.7945 | 117.104 | FALSE | TRUE |
| CHEMBL1221901 | N[C@@H](Cc1ccco1)C(=O)O | alpha | 0.234 | 155.153 | TRUE | TRUE |
| CHEMBL1222007 | Cl.O=C(O)[C@H]1CNCCN1 | alpha | -0.9458 | 166.608 | FALSE | TRUE |
| CHEMBL1222008 | Cl.O=C(O)[C@@H]1CNCCN1 | alpha | -0.9458 | 166.608 | FALSE | TRUE |
| CHEMBL122507 | C=C(c1ccc(OCCCC)cc1)[C@H]1CN[C@H](C(=O)O)[C@H]1CC(=O)O | alpha | 2.6422 | 347.411 | TRUE | TRUE |
| CHEMBL122656 | N[C@@H](Cn1ncc(=O)[nH]c1=O)C(=O)O | alpha | -2.6565 | 200.154 | TRUE | TRUE |
| CHEMBL122888 | Bc1ccc(C[C@H](N)C(=O)O)cc1 | alpha | -1.1006 | 177.012 | TRUE | TRUE |
| CHEMBL1229073 | O=C(O)[C@H]1CNCCN1 | alpha | -1.3676 | 130.147 | FALSE | TRUE |
| CHEMBL1229074 | O=C(O)[C@@H]1CNCCN1 | alpha | -1.3676 | 130.147 | FALSE | TRUE |
| CHEMBL124136 | O=C(O)[C@@H]1CCN[C@@H](C(=O)O)C1 | alpha | -0.4762 | 173.168 | FALSE | TRUE |
| CHEMBL1241388 | NCCS(=O)(=O)C[C@H](N)C(=O)O | alpha | -2.2282 | 196.228 | FALSE | TRUE |
| CHEMBL1241436 | NC/C=C/CC(N)C(=O)O | alpha | -0.6967 | 144.174 | FALSE | FALSE |
| CHEMBL1241438 | NCC(=O)CCC(N)C(=O)O | alpha | -1.2937 | 160.173 | FALSE | FALSE |
| CHEMBL124460 | O=C(O)[C@H]1CCCN[C@H]1C(=O)O | alpha | -0.4762 | 173.168 | FALSE | TRUE |
| CHEMBL124513 | O=C(O)[C@@H]1CCC[C@H](C(=O)O)N1 | alpha | -0.3337 | 173.168 | FALSE | TRUE |
| CHEMBL1253291 | NC(Cc1c[nH]c2ccc([N+](=O)[O-])cc12)C(=O)O | alpha | 1.0305 | 249.226 | TRUE | FALSE |
| CHEMBL1253292 | NC(Cc1c[nH]c2cc([N+](=O)[O-])ccc12)C(=O)O | alpha | 1.0305 | 249.226 | TRUE | FALSE |
| CHEMBL1253496 | CSCC[C@@H](NCC[C@H](O)c1ccccc1)C(=O)O | alpha | 1.906 | 283.393 | TRUE | TRUE |
| CHEMBL1253501 | N[C@H](C(=O)O)[C@@H]1[C@@H](C(=O)O)[C@@H]1C1CC1 | alpha | -0.2449 | 199.206 | FALSE | TRUE |
| CHEMBL1253502 | N[C@H](C(=O)O)[C@@H]1[C@@H](C(=O)O)[C@@H]1C1CCCCC1 | alpha | 0.9254 | 241.287 | FALSE | TRUE |
| CHEMBL1255970 | CCCCS(=O)(=O)CCC(N)C(=O)O | alpha | 0.0033 | 223.294 | FALSE | FALSE |
| CHEMBL1256174 | C[Se]C[C@H](N)C(=O)O.Cl | alpha | -0.0093 | 218.542 | FALSE | TRUE |
| CHEMBL1256243 | O=C(O)[C@H]1[C@H]2CN[C@H](C(=O)O)[C@H]21 | alpha | -1.0104 | 171.152 | FALSE | TRUE |
| CHEMBL1256245 | C[C@]1(C(=O)O)CCN[C@@H]1C(=O)O | alpha | -0.4762 | 173.168 | FALSE | TRUE |
| CHEMBL1256395 | N[C@H](CCC(=O)NCS(=O)(=O)O)C(=O)O | alpha | -1.86 | 240.237 | FALSE | TRUE |
| CHEMBL1256516 | N[C@@H](Cc1cc(O)c(O)cc1O)C(=O)O | alpha | -0.2422 | 213.189 | TRUE | TRUE |
| CHEMBL1256779 | N[C@@H](Cc1cccc(-c2ccccc2CP(=O)(O)O)c1)C(=O)O | alpha | 1.9857 | 335.296 | TRUE | TRUE |
| CHEMBL125933 | O=C(O)[C@@H]1CC[C@H](C(=O)O)NC1 | alpha | -0.4762 | 173.168 | FALSE | TRUE |
| CHEMBL126092 | O=C(O)[C@H]1CC[C@H](C(=O)O)NC1 | alpha | -0.4762 | 173.168 | FALSE | TRUE |
| CHEMBL126231 | O=C(O)[C@H]1C[C@@H](CCCc2nnn[nH]2)CCN1 | alpha | -0.0248 | 239.279 | TRUE | TRUE |
| CHEMBL126297 | O=C(O)[C@H]1C[C@@H](CSc2nn[nH]n2)CCN1 | alpha | -0.2554 | 243.292 | TRUE | TRUE |
| CHEMBL126608 | N[C@@H](CCn1oc(=O)[nH]c1=O)C(=O)O | alpha | -2.0684 | 203.154 | TRUE | TRUE |
| CHEMBL126727 | O=C(O)[C@H]1C[C@@H](CCc2nnn[nH]2)CCN1 | alpha | -0.4149 | 225.252 | TRUE | TRUE |
| CHEMBL126881 | O=C(O)[C@H]1C[C@@H](Sc2nn[nH]n2)CCN1 | alpha | -0.503 | 229.265 | TRUE | TRUE |
| CHEMBL127490 | O=C(O)[C@H]1CCN[C@@H](C(=O)O)C1 | alpha | -0.4762 | 173.168 | FALSE | TRUE |
| CHEMBL127503 | N[C@@H](CNC(=O)C1=NOC(CCCCNc2ncc[nH]2)C1)C(=O)O | alpha | -0.3351 | 338.368 | TRUE | TRUE |
| CHEMBL1275735 | O=CN[C@@H](Cc1ccc(O)cc1)C(=O)O | alpha | 0.1339 | 209.201 | TRUE | TRUE |
| CHEMBL1275922 | C[C@H]1CC[C@@H](O)[C@@H](C(=O)O)N1 | alpha | -0.4276 | 159.185 | FALSE | TRUE |
| CHEMBL1276129 | CC(C)=CCn1cc(CC(N)C(=O)O)c2ccccc21 | alpha | 2.5619 | 272.348 | TRUE | FALSE |
| CHEMBL1276130 | CC(C)=CCc1[nH]c2ccccc2c1CC(N)C(=O)O | alpha | 2.631 | 272.348 | TRUE | FALSE |
| CHEMBL127739 | CC(=N)NCCCCCC(N)C(=O)O | alpha | 0.54547 | 201.27 | FALSE | FALSE |
| CHEMBL128772 | N[C@H](C(=O)O)c1ccc(O)c(C(=O)O)c1 | alpha | 0.1748 | 211.173 | TRUE | TRUE |
| CHEMBL1288332 | N[C@@H](Cc1ccc(C(Br)P(=O)(O)O)cc1)C(=O)O | alpha | 1.2122 | 338.094 | TRUE | TRUE |
| CHEMBL1288410 | N[C@@H](Cn1cc(C(=O)O)nn1)C(=O)O | alpha | -1.6118 | 200.154 | TRUE | TRUE |
| CHEMBL1288412 | N[C@@H](Cn1nncc1C(=O)O)C(=O)O | alpha | -1.6118 | 200.154 | TRUE | TRUE |
| CHEMBL1288481 | C#CCSC[C@H](N)C(=O)O | alpha | -0.2353 | 159.21 | FALSE | TRUE |
| CHEMBL1288491 | N[C@@H](Cc1cnnn1CC(=O)O)C(=O)O | alpha | -1.6829 | 214.181 | TRUE | TRUE |
| CHEMBL1289658 | N[C@@H](Cc1cn([C@@H](Cc2ccccc2)C(=O)O)nn1)C(=O)O | alpha | 0.1009 | 304.306 | TRUE | TRUE |
| CHEMBL1289762 | N[C@@H](Cc1cnnn1[C@@H](Cc1ccccc1)C(=O)O)C(=O)O | alpha | 0.1009 | 304.306 | TRUE | TRUE |
| CHEMBL1302700 | CC(C)C[C@H](NS(=O)(=O)c1ccc(Br)cc1)C(=O)O | alpha | 2.2267 | 350.234 | TRUE | TRUE |
| CHEMBL1304069 | COc1ccc(S(=O)(=O)N[C@H](C(=O)O)C(C)C)cc1 | alpha | 1.0827 | 287.337 | TRUE | TRUE |
| CHEMBL1304941 | CC(C)CNC(CC(=O)Nc1cccc([N+](=O)[O-])c1)C(=O)O | alpha | 1.6222 | 309.322 | TRUE | FALSE |
| CHEMBL1305618 | CC(C)CNC(=O)CC(NCC(C)C)C(=O)O | alpha | 0.8475 | 244.335 | FALSE | FALSE |
| CHEMBL130651 | CCSc1ccc(C(N)C(=O)O)cc1 | alpha | 1.883 | 211.286 | TRUE | FALSE |
| CHEMBL1306538 | NC(C(=O)O)C(c1ccccc1)C(F)F | alpha | 1.4472 | 215.199 | TRUE | FALSE |
| CHEMBL1308107 | Cc1cc(C)c(C)c(S(=O)(=O)NC(C)C(=O)O)c1C | alpha | 1.67168 | 285.365 | TRUE | FALSE |
| CHEMBL1311562 | CC(C)C(Nc1ncc([N+](=O)[O-])cc1[N+](=O)[O-])C(=O)O | alpha | 1.4191 | 284.228 | TRUE | FALSE |
| CHEMBL1311694 | O=C(O)[C@H]1NC[C@H]2C[C@H]21 | alpha | -0.3211 | 127.143 | FALSE | TRUE |
| CHEMBL1317696 | C=CCc1cccc(C2NC(C(=O)O)CCC2C)c1 | alpha | 2.9289 | 259.349 | TRUE | FALSE |
| CHEMBL1317921 | N[C@@H](CSCc1ccc(I)cc1)C(=O)O | alpha | 1.9363 | 337.182 | TRUE | TRUE |
| CHEMBL1318748 | CCCC(=O)C1=C(NC(C)C(=O)O)CCCC1=O | alpha | 1.4254 | 253.298 | FALSE | FALSE |
| CHEMBL131922 | N[C@@H](CCc1nsnc1O)C(=O)O | alpha | -0.4118 | 203.223 | TRUE | TRUE |
| CHEMBL1322301 | Cc1cc(C(=O)O)sc1C(N)C(=O)O | alpha | 0.83912 | 215.23 | TRUE | FALSE |
| CHEMBL1322497 | Cc1sc2ncnc(NC(C)C(=O)O)c2c1C | alpha | 2.19314 | 251.311 | TRUE | FALSE |
| CHEMBL132382 | CSc1ccc(C(N)C(=O)O)cc1 | alpha | 1.4929 | 197.259 | TRUE | FALSE |
| CHEMBL1324617 | Cc1cccc(C)c1NC(=O)CC(NCc1ccco1)C(=O)O | alpha | 2.46804 | 316.357 | TRUE | FALSE |
| CHEMBL1328080 | CC1NC(C(=O)O)Cc2cc(O)c(O)cc21 | alpha | 0.7577 | 223.228 | TRUE | FALSE |
| CHEMBL1329593 | CC(=O)/C=C(/C)NC(Cc1c[nH]c2ccccc12)C(=O)O | alpha | 2.246 | 286.331 | TRUE | FALSE |
| CHEMBL1334067 | Cc1cc(=O)oc2cc(NC(=O)CC(N)C(=O)O)ccc12 | alpha | 0.84192 | 290.275 | TRUE | FALSE |
| CHEMBL1334284 | Cc1ccc(S(=O)(=O)NC(Cc2ccccc2)C(=O)O)cc1 | alpha | 1.96922 | 319.382 | TRUE | FALSE |
| CHEMBL1334639 | Cl.O=C(O)C1NCCc2ccccc21 | alpha | 1.3798 | 213.664 | TRUE | FALSE |
| CHEMBL1335288 | CCOc1ccc(C(=O)CC(NCc2cccs2)C(=O)O)cc1 | alpha | 2.9626 | 333.409 | TRUE | FALSE |
| CHEMBL1337648 | Cc1ccc(S(=O)(=O)N[C@H](CO)C(=O)O)cc1 | alpha | -0.28118 | 259.283 | TRUE | TRUE |
| CHEMBL1338809 | O=C(CC(Nc1ccccc1Cl)C(=O)O)c1ccccc1 | alpha | 3.4781 | 303.745 | TRUE | FALSE |
| CHEMBL1339370 | CC(Nc1nc(Cl)ccc1[N+](=O)[O-])C(=O)O | alpha | 1.5282 | 245.622 | TRUE | FALSE |
| CHEMBL1342299 | O=C(O)C1CSC(c2ccco2)N1 | alpha | 1.0678 | 199.231 | TRUE | FALSE |
| CHEMBL1342752 | Cc1ccc(C)c(S(=O)(=O)NC(CCC(N)=O)C(=O)O)c1 | alpha | 0.30044 | 314.363 | TRUE | FALSE |
| CHEMBL1345524 | CC(=O)Nc1ccc(S(=O)(=O)N[C@H](C(=O)O)C(C)C)cc1 | alpha | 1.0325 | 314.363 | TRUE | TRUE |
| CHEMBL1352408 | CSCCC(NS(=O)(=O)c1ccc2ccccc2c1)C(=O)O | alpha | 2.3244 | 339.438 | TRUE | FALSE |
| CHEMBL1352451 | Cl.NC(Cc1cccc2nsnc12)C(=O)O | alpha | 1.0675 | 259.718 | TRUE | FALSE |
| CHEMBL1357546 | N[C@H](C(=O)O)C(O)C(O)C(O)CO | alpha | -3.5267 | 195.171 | FALSE | TRUE |
| CHEMBL1357932 | N[C@@H](C(=O)O)c1ccc(C(=O)O)c(O)c1 | alpha | 0.1748 | 211.173 | TRUE | TRUE |
| CHEMBL1359060 | CCCCOc1ccc(C(=O)CCNC(C(=O)O)C(C)CC)cc1 | alpha | 3.5272 | 335.444 | TRUE | FALSE |
| CHEMBL1359852 | N[C@@H](Cc1ccnnc1)C(=O)O | alpha | -0.569 | 167.168 | TRUE | TRUE |
| CHEMBL1360548 | COc1ccccc1NC(=O)CC(NCc1ccccc1)C(=O)O | alpha | 2.2668 | 328.368 | TRUE | FALSE |
| CHEMBL136848 | Cc1ccc(S(=O)(=O)O)cc1.Nc1ccccc1NC[C@H](N)C(=O)O | alpha | 1.33432 | 367.427 | TRUE | TRUE |
| CHEMBL1369068 | CC(C)c1ccc(C2NC(C(=O)O)CS2)cc1 | alpha | 2.5982 | 251.351 | TRUE | FALSE |
| CHEMBL1369156 | O=C(O)C1CSC(c2ccc(O)c([N+](=O)[O-])c2)N1 | alpha | 1.0886 | 270.266 | TRUE | FALSE |
| CHEMBL1371127 | N[C@H](Cc1onc(O)c1Cl)C(=O)O | alpha | -0.012 | 206.585 | TRUE | TRUE |
| CHEMBL1372427 | Nc1ccc(S(=O)(=O)NC(Cc2ccccc2)C(=O)O)cc1 | alpha | 1.243 | 320.37 | TRUE | FALSE |
| CHEMBL1378278 | O=C(CC(NCc1cccnc1)C(=O)O)Nc1ccc(O)cc1 | alpha | 1.3588 | 315.329 | TRUE | FALSE |
| CHEMBL138085 | N[C@H](Cn1c(=O)c(=O)[nH]c2ccccc21)C(=O)O | alpha | -0.8983 | 249.226 | TRUE | TRUE |
| CHEMBL1382407 | Cc1ccc(CSCC(N)C(=O)O)cc1 | alpha | 1.64012 | 225.313 | TRUE | FALSE |
| CHEMBL138410 | Cc1ccc(S(=O)(=O)O)cc1.Nc1ccccc1NC[C@@H](N)C(=O)O | alpha | 1.33432 | 367.427 | TRUE | TRUE |
| CHEMBL1387275 | CSCC[C@H](Nc1ncnc2sc(C)c(C)c12)C(=O)O | alpha | 2.92634 | 311.432 | TRUE | TRUE |
| CHEMBL1388797 | CC(=O)Nc1ccc(S(=O)(=O)NC(C(=O)O)C(C)O)cc1 | alpha | -0.2427 | 316.335 | TRUE | FALSE |
| CHEMBL1388818 | CSCCC(NS(=O)(=O)/C=C/c1ccccc1)C(=O)O | alpha | 1.783 | 315.416 | TRUE | FALSE |
| CHEMBL139279 | Cc1cc(N)c(NC[C@H](N)C(=O)O)cc1C.Cc1ccc(S(=O)(=O)O)cc1 | alpha | 1.95116 | 395.481 | TRUE | TRUE |
| CHEMBL1394949 | CC1CCC(C(=O)O)NC1c1ccc(C#CCN(C)C)cc1 | alpha | 2.1135 | 300.402 | TRUE | FALSE |
| CHEMBL139714 | N[C@H](C[C@H](Cc1cccc(Br)c1)C(=O)O)C(=O)O | alpha | 1.4944 | 316.151 | TRUE | TRUE |
| CHEMBL1397746 | CN(C)c1ccc(NC(=O)CC(NCc2cccnc2)C(=O)O)cc1 | alpha | 1.7192 | 342.399 | TRUE | FALSE |
| CHEMBL139780 | N[C@H](C[C@H](C/C=C/C=C/c1ccccc1)C(=O)O)C(=O)O | alpha | 2.1489 | 289.331 | TRUE | TRUE |
| CHEMBL139876 | C#CC[C@@H](C[C@@H](N)C(=O)O)C(=O)O | alpha | -0.4875 | 185.179 | FALSE | TRUE |
| CHEMBL139883 | N[C@H](C[C@H](CCCC1CCCCC1)C(=O)O)C(=O)O | alpha | 2.2398 | 271.357 | FALSE | TRUE |
| CHEMBL139901 | CCCCCCCCCCC1NC(C(=O)O)CS1 | alpha | 3.6329 | 273.442 | FALSE | FALSE |
| CHEMBL139909 | NC(CC(=O)c1ccc(Cl)c(Cl)c1)C(=O)O | alpha | 1.9781 | 262.092 | TRUE | FALSE |
| CHEMBL140001 | N[C@H](C[C@H](Cc1ccc([N+](=O)[O-])cc1)C(=O)O)C(=O)O | alpha | 0.6401 | 282.252 | TRUE | TRUE |
| CHEMBL140049 | N[C@H](C[C@H](CCCc1ccccc1)C(=O)O)C(=O)O | alpha | 1.5121 | 265.309 | TRUE | TRUE |
| CHEMBL140164 | N[C@H](C[C@H](Cc1ccc(Cl)cc1)C(=O)O)C(=O)O | alpha | 1.3853 | 271.7 | TRUE | TRUE |
| CHEMBL140197 | N[C@H](C(=O)O)[C@H]1[C@@H](O)[C@@H]1C(=O)O | alpha | -1.9102 | 175.14 | FALSE | TRUE |
| CHEMBL1402066 | CCc1cc2c(NC(C(=O)O)C(C)C)ncnc2s1 | alpha | 2.7748 | 279.365 | TRUE | FALSE |
| CHEMBL140214 | N[C@H](C[C@H](Cc1cccs1)C(=O)O)C(=O)O | alpha | 0.7934 | 243.284 | TRUE | TRUE |
| CHEMBL1402765 | N[C@@H](C(=O)O)c1ccc(C(=O)O)cc1 | alpha | 0.4692 | 195.174 | TRUE | TRUE |
| CHEMBL140318 | N[C@H](C[C@H](Cc1sc2ccccc2c1Cl)C(=O)O)C(=O)O | alpha | 2.6 | 327.789 | TRUE | TRUE |
| CHEMBL140378 | N[C@H](C[C@H](Cc1ccccc1)C(=O)O)C(=O)O | alpha | 0.7319 | 237.255 | TRUE | TRUE |
| CHEMBL140460 | CCCCCCCc1cccc(C2N[C@H](C(=O)O)CS2)c1 | alpha | 3.9877 | 307.459 | TRUE | TRUE |
| CHEMBL140462 | N[C@H](C[C@H](C/C=C/c1ccc2ccccc2c1)C(=O)O)C(=O)O | alpha | 2.7459 | 313.353 | TRUE | TRUE |
| CHEMBL140465 | N[C@H](C[C@H](Cc1cc2ccccc2[nH]1)C(=O)O)C(=O)O | alpha | 1.2132 | 276.292 | TRUE | TRUE |
| CHEMBL140562 | N[C@H](C[C@H](Oc1ccc(Oc2ccccc2)cc1)C(=O)O)C(=O)O | alpha | 2.1129 | 331.324 | TRUE | TRUE |
| CHEMBL140563 | N[C@H](C[C@H](Oc1ccc(-c2ccccc2)cc1)C(=O)O)C(=O)O | alpha | 1.9876 | 315.325 | TRUE | TRUE |
| CHEMBL140615 | N[C@H](C[C@H](Cc1nc2ccccc2s1)C(=O)O)C(=O)O | alpha | 1.3416 | 294.332 | TRUE | TRUE |
| CHEMBL140784 | NC(C(=O)O)c1nnn[nH]1 | alpha | -1.7159 | 143.106 | TRUE | FALSE |
| CHEMBL140838 | N[C@@H](Cn1c2c(c(=O)[nH]c1=S)CCC2)C(=O)O | alpha | -0.19351 | 255.299 | TRUE | TRUE |
| CHEMBL1410826 | Cc1ccc(S(=O)(=O)N[C@H](C(=O)O)C(C)C)cc1 | alpha | 1.38252 | 271.338 | TRUE | TRUE |
| CHEMBL141153 | COc1ccc(C[C@@H](C[C@@H](N)C(=O)O)C(=O)O)cc1 | alpha | 0.7405 | 267.281 | TRUE | TRUE |
| CHEMBL1412055 | Cc1nc2ccc(S(=O)(=O)NC(CC(C)C)C(=O)O)cc2s1 | alpha | 2.38232 | 342.442 | TRUE | FALSE |
| CHEMBL1412824 | Cl.NCCO/C=C/[C@H](N)C(=O)O | alpha | -0.6909 | 196.634 | FALSE | TRUE |
| CHEMBL141293 | N[C@H](C[C@H](Cc1cc(Cl)cc(Cl)c1)C(=O)O)C(=O)O | alpha | 2.0387 | 306.145 | TRUE | TRUE |
| CHEMBL1413097 | COc1ccc([N+](=O)[O-])c2c1CCN2C(=O)CC[C@H](N)C(=O)O | alpha | 0.6846 | 323.305 | TRUE | TRUE |
| CHEMBL141353 | N[C@H](C[C@H](Cc1cccc([N+](=O)[O-])c1)C(=O)O)C(=O)O | alpha | 0.6401 | 282.252 | TRUE | TRUE |
| CHEMBL141357 | N[C@H](C[C@H](Cc1ccccc1Br)C(=O)O)C(=O)O | alpha | 1.4944 | 316.151 | TRUE | TRUE |
| CHEMBL1413580 | NC(CCC(=O)NCS(=O)(=O)O)C(=O)O | alpha | -1.86 | 240.237 | FALSE | FALSE |
| CHEMBL1415165 | O=C(O)C1CSC(CCc2ccccc2)N1 | alpha | 1.7349 | 237.324 | TRUE | FALSE |
| CHEMBL141519 | N[C@H](C[C@H](Cc1cccc(-c2ccccc2)c1)C(=O)O)C(=O)O | alpha | 2.3989 | 313.353 | TRUE | TRUE |
| CHEMBL1416409 | CSCCC(NS(=O)(=O)c1ccc(Cl)cc1)C(=O)O | alpha | 1.8246 | 323.823 | TRUE | FALSE |
| CHEMBL1416461 | O=C(O)C1Nc2ccccc2S(=O)(=O)N1 | alpha | -0.1989 | 228.229 | TRUE | FALSE |
| CHEMBL1416659 | NC(Cc1cccc(-c2ccccc2CP(=O)(O)O)c1)C(=O)O | alpha | 1.9857 | 335.296 | TRUE | FALSE |
| CHEMBL1417207 | CC(C)[C@H](Nc1ncnc2[nH]cnc12)C(=O)O | alpha | 0.874 | 235.247 | TRUE | TRUE |
| CHEMBL1419531 | COc1ccccc1NC(=O)CC(NCCN(C)C)C(=O)O | alpha | 0.6282 | 309.366 | TRUE | FALSE |
| CHEMBL141985 | N[C@@H](CCc1nnn[nH]1)C(=O)O | alpha | -1.4558 | 171.16 | TRUE | TRUE |
| CHEMBL1420678 | CC(C)C(Nc1ccc(C(F)(F)F)cc1[N+](=O)[O-])C(=O)O | alpha | 3.1347 | 306.24 | TRUE | FALSE |
| CHEMBL142252 | NC(C(=O)O)[C@@H](CC(=O)O)c1ccc(Cl)cc1 | alpha | 1.3102 | 257.673 | TRUE | TRUE |
| CHEMBL1423103 | O=C(O)[C@@H]1Cc2ccccc2N1 | alpha | 1.1078 | 163.176 | TRUE | TRUE |
| CHEMBL1423173 | CC(C)CC(Nc1nc(Cl)ccc1[N+](=O)[O-])C(=O)O | alpha | 2.5544 | 287.703 | TRUE | FALSE |
| CHEMBL1425564 | CSCCC(NS(=O)(=O)c1ccc(C)c(C(=O)O)c1)C(=O)O | alpha | 1.17782 | 347.414 | TRUE | FALSE |
| CHEMBL1426906 | Cc1cc(C)c(C)c(S(=O)(=O)NC(C(=O)O)C(C)C)c1C | alpha | 2.30778 | 313.419 | TRUE | FALSE |
| CHEMBL142749 | NC(C(=O)O)[C@H](CC(=O)O)c1ccc(Cl)cc1 | alpha | 1.3102 | 257.673 | TRUE | TRUE |
| CHEMBL142765 | CCCCCCCCCCC[C@H]1N[C@H](C(=O)O)CS1 | alpha | 4.023 | 287.469 | FALSE | TRUE |
| CHEMBL1428051 | Cc1ccc(S(=O)(=O)NC(Cc2ccccc2)C(=O)O)cc1C | alpha | 2.27764 | 333.409 | TRUE | FALSE |
| CHEMBL142994 | CCCCCCCCCCCC1NC(C(=O)O)CCS1 | alpha | 4.4131 | 301.496 | FALSE | FALSE |
| CHEMBL1430426 | CC(NS(=O)(=O)/C=C/c1ccccc1)C(=O)O | alpha | 1.0498 | 255.295 | TRUE | FALSE |
| CHEMBL143045 | CCCCCCCCCCC1NC(C(=O)O)CCS1 | alpha | 4.023 | 287.469 | FALSE | FALSE |
| CHEMBL143048 | CCCCCCCCCCCCCC1NC(C(=O)O)CCS1 | alpha | 5.1933 | 329.55 | FALSE | FALSE |
| CHEMBL143119 | CCCCCCCCCC1NC(C(=O)O)CCS1 | alpha | 3.6329 | 273.442 | FALSE | FALSE |
| CHEMBL143142 | CCCCCCCCCc1cccc(C2N[C@H](C(=O)O)CS2)c1 | alpha | 4.7679 | 335.513 | TRUE | TRUE |
| CHEMBL143151 | CCCCCCCCCCCCC1NC(C(=O)O)CS1 | alpha | 4.4131 | 301.496 | FALSE | FALSE |
| CHEMBL143172 | CCCCCCCCCCCCC1NC(C(=O)O)CCS1 | alpha | 4.8032 | 315.523 | FALSE | FALSE |
| CHEMBL143395 | COc1noc(C(=O)O)c1CC(N)C(=O)O | alpha | -0.6642 | 230.176 | TRUE | FALSE |
| CHEMBL1434796 | CC[C@@H](Nc1ncnc2[nH]cnc12)C(=O)O | alpha | 0.628 | 221.22 | TRUE | TRUE |
| CHEMBL1436464 | Cc1cn(CC[C@H](N)C(=O)O)c(=O)[nH]c1=O | alpha | -1.35298 | 227.22 | TRUE | TRUE |
| CHEMBL143651 | CCCCCCCCCc1ccc(C2N[C@H](C(=O)O)CS2)cc1 | alpha | 4.7679 | 335.513 | TRUE | TRUE |
| CHEMBL143832 | CCCCCCC1NC(C(=O)O)CS1 | alpha | 2.0725 | 217.334 | FALSE | FALSE |
| CHEMBL143888 | N[C@H](C[C@H](Cc1cccc(-c2ccco2)c1)C(=O)O)C(=O)O | alpha | 1.9919 | 303.314 | TRUE | TRUE |
| CHEMBL144040 | CC(=O)Nc1ccc(O)cc1.CC1N[C@H](C(=O)O)CS1 | alpha | 1.4726 | 298.364 | TRUE | TRUE |
| CHEMBL1440472 | CC(C)C(Nc1nc(Cl)ccc1[N+](=O)[O-])C(=O)O | alpha | 2.1643 | 273.676 | TRUE | FALSE |
| CHEMBL1440574 | NC(CSCc1ccccc1Cl)C(=O)O | alpha | 1.9851 | 245.731 | TRUE | FALSE |
| CHEMBL1440980 | CCc1cc2c(N[C@@H](C)C(=O)O)ncnc2s1 | alpha | 2.1387 | 251.311 | TRUE | TRUE |
| CHEMBL1441607 | COCC#Cc1ccc(C2NC(C(=O)O)CCC2C)cc1 | alpha | 2.1983 | 287.359 | TRUE | FALSE |
| CHEMBL144251 | NC(CCNO)C(=O)O | alpha | -1.2328 | 134.135 | FALSE | FALSE |
| CHEMBL1442851 | N[C@@H](C(=O)O)[C@@H]1C[C@H]1C(=O)O | alpha | -0.881 | 159.141 | FALSE | TRUE |
| CHEMBL144462 | O=C(O)[C@H]1CC[C@H]2CN[C@H](C(=O)O)C[C@H]2C1 | alpha | 0.55 | 227.26 | FALSE | TRUE |
| CHEMBL1447185 | CN(C)c1ccc(NC(=O)CC(NCc2ccccn2)C(=O)O)cc1 | alpha | 1.7192 | 342.399 | TRUE | FALSE |
| CHEMBL144733 | NC(Cc1c(C(=O)O)on(Cc2ccccc2)c1=O)C(=O)O | alpha | 0.1422 | 306.274 | TRUE | FALSE |
| CHEMBL144772 | Cn1oc(C(=O)O)c(CC(N)C(=O)O)c1=O | alpha | -1.3691 | 230.176 | TRUE | FALSE |
| CHEMBL1448040 | N[C@@H](Cc1c[nH]c2cc([N+](=O)[O-])ccc12)C(=O)O.O=[N+]([O-])O | alpha | 0.6828 | 312.238 | TRUE | TRUE |
| CHEMBL1448057 | O=C(O)[C@@H]1C[C@H]2C[C@@H](CP(=O)(O)O)CC[C@H]2CN1 | alpha | 0.6431 | 277.257 | FALSE | TRUE |
| CHEMBL1448318 | CCCCC(Nc1ncnc2ccccc12)C(=O)O | alpha | 2.6851 | 259.309 | TRUE | FALSE |
| CHEMBL1449605 | N[C@@H](Cc1ncccn1)C(=O)O | alpha | -0.569 | 167.168 | TRUE | TRUE |
| CHEMBL1449772 | N[C@@H](Cc1cccc([N+](=O)[O-])c1)C(=O)O | alpha | 0.5492 | 210.189 | TRUE | TRUE |
| CHEMBL145074 | O=C(O)C1CN(CCCc2nnn[nH]2)CCN1 | alpha | -1.5093 | 240.267 | TRUE | FALSE |
| CHEMBL1453118 | CSCCC(Nc1nc2ccccc2s1)C(=O)O | alpha | 2.9145 | 282.39 | TRUE | FALSE |
| CHEMBL145362 | O=C(O)C1CN(Cc2nn[nH]n2)CCN1 | alpha | -1.9419 | 212.213 | TRUE | FALSE |
| CHEMBL1454048 | CCCC(NS(=O)(=O)c1ccc(C)cc1)C(=O)O | alpha | 1.52662 | 271.338 | TRUE | FALSE |
| CHEMBL145406 | O=C(O)C1CN(CCc2nnn[nH]2)CCN1 | alpha | -1.8994 | 226.24 | TRUE | FALSE |
| CHEMBL145467 | CC(=O)Nc1ccc(O)cc1.O=C(O)C1CSCN1 | alpha | 1.0841 | 284.337 | TRUE | FALSE |
| CHEMBL1455163 | Cl.NC(Cc1ccc2nonc2c1)C(=O)O | alpha | 0.599 | 243.65 | TRUE | FALSE |
| CHEMBL146122 | NC(CCCNc1cccc(O)c1)C(=O)O | alpha | 0.9962 | 224.26 | TRUE | FALSE |
| CHEMBL146168 | N[C@H](CCc1nnn[nH]1)C(=O)O | alpha | -1.4558 | 171.16 | TRUE | TRUE |
| CHEMBL146236 | NC(CCCNc1ccc(O)cc1)C(=O)O | alpha | 0.9962 | 224.26 | TRUE | FALSE |
| CHEMBL1463075 | CCOC1=N/C(=C(\C)Cl)c2ccccc2C(C(=O)O)N1 | alpha | 2.7353 | 294.738 | TRUE | FALSE |
| CHEMBL1465998 | O=C(CC(Nc1cccc(Cl)c1Cl)C(=O)O)c1ccccc1 | alpha | 4.1315 | 338.19 | TRUE | FALSE |
| CHEMBL1466100 | NC(CCCCCP(=O)(c1ccccc1)c1ccccc1)C(=O)O | alpha | 2.9728 | 345.379 | TRUE | FALSE |
| CHEMBL146792 | O=C(O)[C@H](Cc1ccccc1)NS(=O)(=O)NCCc1ccccc1 | alpha | 1.349 | 348.424 | TRUE | TRUE |
| CHEMBL1468396 | Cc1ccc(S(=O)(=O)NC(Cc2c[nH]cn2)C(=O)O)cc1 | alpha | 0.69232 | 309.347 | TRUE | FALSE |
| CHEMBL1469741 | CCCC(NS(=O)(=O)c1ccc(OC)cc1)C(=O)O | alpha | 1.2268 | 287.337 | TRUE | FALSE |
| CHEMBL1471434 | Cc1sc2ncnc(N[C@@H](CCC(=O)O)C(=O)O)c2c1C | alpha | 2.03804 | 309.347 | TRUE | TRUE |
| CHEMBL147196 | O=C(O)[C@@H]1CSC(CCc2ccccc2O)N1 | alpha | 1.4405 | 253.323 | TRUE | TRUE |
| CHEMBL1472699 | C[Se]C[C@@H](N)C(=O)O | alpha | -0.4311 | 182.081 | FALSE | TRUE |
| CHEMBL147310 | CC(C)NS(=O)(=O)N[C@@H](Cc1ccccc1)C(=O)O | alpha | 0.5147 | 286.353 | TRUE | TRUE |
| CHEMBL1473270 | CC[C@H](C)[C@@H]1CN[C@H](C(=O)O)[C@H]1CC(=O)O | alpha | 0.796 | 229.276 | FALSE | TRUE |
| CHEMBL1474906 | CC(=O)Nc1ccc(C2NC(C(=O)O)CCC2C)cc1 | alpha | 2.1588 | 276.336 | TRUE | FALSE |
| CHEMBL1475027 | COCCCNC(CC(=O)Nc1cc(C)cc(C)c1)C(=O)O | alpha | 1.71134 | 308.378 | TRUE | FALSE |
| CHEMBL147548 | CCCCCOc1noc(C(=O)O)c1CC(N)C(=O)O | alpha | 0.8962 | 286.284 | TRUE | FALSE |
| CHEMBL1476294 | CC[C@@H](C)[C@H]1CN[C@H](C(=O)O)[C@H]1CC(=O)O | alpha | 0.796 | 229.276 | FALSE | TRUE |
| CHEMBL147805 | C=CCOc1noc(C(=O)O)c1CC(N)C(=O)O | alpha | -0.108 | 256.214 | TRUE | FALSE |
| CHEMBL147857 | NC(Cc1c(OCc2ccccc2)noc1C(=O)O)C(=O)O | alpha | 0.9062 | 306.274 | TRUE | FALSE |
| CHEMBL147911 | C/C=C/COc1noc(C(=O)O)c1CC(N)C(=O)O | alpha | 0.2821 | 270.241 | TRUE | FALSE |
| CHEMBL1479165 | COc1ccc(NC(=O)CC(NCc2ccccn2)C(=O)O)cc1 | alpha | 1.6618 | 329.356 | TRUE | FALSE |
| CHEMBL1479386 | O=C(CC(Nc1ccc(F)cc1)C(=O)O)c1ccccc1 | alpha | 2.9638 | 287.29 | TRUE | FALSE |
| CHEMBL1480198 | CC(C)CC(NS(=O)(=O)c1cc(C(=O)O)ccc1Cl)C(=O)O | alpha | 1.8158 | 349.792 | TRUE | FALSE |
| CHEMBL148028 | CCOc1noc(-c2nc[nH]n2)c1CC(N)C(=O)O | alpha | -0.1872 | 267.245 | TRUE | FALSE |
| CHEMBL148247 | CCCCOc1noc(C(=O)O)c1CC(N)C(=O)O | alpha | 0.5061 | 272.257 | TRUE | FALSE |
| CHEMBL1482527 | N[C@@H](CC1=CS(=O)(=O)c2ccc(Cl)cc21)C(=O)O | alpha | 1.2702 | 287.724 | TRUE | TRUE |
| CHEMBL148375 | CC(C)Oc1noc(C(=O)O)c1CC(N)C(=O)O | alpha | 0.1144 | 258.23 | TRUE | FALSE |
| CHEMBL1486352 | O=C(CC(Nc1ccc(Cl)cc1)C(=O)O)c1ccccc1 | alpha | 3.4781 | 303.745 | TRUE | FALSE |
| CHEMBL1488171 | Cc1ccc(S(=O)(=O)N[C@H](CN)C(=O)O)cc1 | alpha | -0.31478 | 258.299 | TRUE | TRUE |
| CHEMBL1488677 | CCCC(NS(=O)(=O)c1ccc(Br)cc1)C(=O)O | alpha | 1.9807 | 336.207 | TRUE | FALSE |
| CHEMBL149149 | NC(CNc1ccc(O)cc1)C(=O)O | alpha | 0.216 | 196.206 | TRUE | FALSE |
| CHEMBL1492035 | Cc1ccc2c(c1)NC(C(=O)O)NS2(=O)=O | alpha | 0.10952 | 242.256 | TRUE | FALSE |
| CHEMBL1492282 | Cc1ccc(C)c(S(=O)(=O)NC(CC(C)C)C(=O)O)c1 | alpha | 2.08104 | 299.392 | TRUE | FALSE |
| CHEMBL1493598 | CCC(NS(=O)(=O)c1ccc(C)cc1)C(=O)O | alpha | 1.13652 | 257.311 | TRUE | FALSE |
| CHEMBL1494002 | O=C(O)[C@@H]1NC[C@H]2ON=C(O)[C@H]21 | alpha | -1.0707 | 172.14 | FALSE | TRUE |
| CHEMBL1495312 | Cc1ccc(NC(CC(=O)c2ccccc2)C(=O)O)cc1Cl | alpha | 3.78652 | 317.772 | TRUE | FALSE |
| CHEMBL1495722 | CC(C)CCOc1ccc(C(=O)CCNC(C)C(=O)O)cc1 | alpha | 2.747 | 307.39 | TRUE | FALSE |
| CHEMBL1495924 | NC(C(=O)O)c1cc(O)c(Cl)c(O)c1 | alpha | 0.8356 | 217.608 | TRUE | FALSE |
| CHEMBL1496443 | COCCCNC(CC(=O)Nc1ccc(OC)cc1OC)C(=O)O | alpha | 1.1117 | 340.376 | TRUE | FALSE |
| CHEMBL1496855 | N[C@H](C(=O)O)c1nnn[nH]1 | alpha | -1.7159 | 143.106 | TRUE | TRUE |
| CHEMBL1497038 | COCCCNC(CC(=O)Nc1ccc(N(C)C)cc1)C(=O)O | alpha | 1.1605 | 323.393 | TRUE | FALSE |
| CHEMBL1500447 | O=C(CC(Nc1ccc(Cl)cc1Cl)C(=O)O)c1ccccc1 | alpha | 4.1315 | 338.19 | TRUE | FALSE |
| CHEMBL1503118 | CC(=O)NCc1ccc(S(=O)(=O)NC(C(=O)O)C(C)C)cc1 | alpha | 0.7102 | 328.39 | TRUE | FALSE |
| CHEMBL1505473 | CC(C)C(NS(=O)(=O)c1cccc(C(F)(F)F)c1)C(=O)O | alpha | 2.0929 | 325.308 | TRUE | FALSE |
| CHEMBL1506252 | O=C(CC(Nc1ccccc1)C(=O)O)c1ccccc1 | alpha | 2.8247 | 269.3 | TRUE | FALSE |
| CHEMBL1506853 | CC(C)C(NCc1c(Cl)cccc1Cl)C(=O)O | alpha | 3.1922 | 276.163 | TRUE | FALSE |
| CHEMBL1507491 | O=C(CC(NCc1ccccn1)C(=O)O)Nc1ccccc1Cl | alpha | 2.3066 | 333.775 | TRUE | FALSE |
| CHEMBL1509054 | CCC(NS(=O)(=O)c1ccc(NC(C)=O)cc1)C(=O)O | alpha | 0.7865 | 300.336 | TRUE | FALSE |
| CHEMBL1511784 | Cc1c(Cl)cccc1NC(CC(=O)c1ccccc1)C(=O)O | alpha | 3.78652 | 317.772 | TRUE | FALSE |
| CHEMBL151245 | O=C(O)[C@H](Cc1ccccc1)NS(=O)(=O)NCc1ccccc1 | alpha | 1.3065 | 334.397 | TRUE | TRUE |
| CHEMBL1512683 | N[C@@H](C(=O)O)c1ccc(P(=O)(O)O)cc1 | alpha | -0.426 | 231.144 | TRUE | TRUE |
| CHEMBL1513845 | N[C@@H](C(=O)O)c1ccc(O)c(C(=O)O)c1 | alpha | 0.1748 | 211.173 | TRUE | TRUE |
| CHEMBL1514670 | N[C@H](Cc1cccc(-c2ccccc2CP(=O)(O)O)c1)C(=O)O | alpha | 1.9857 | 335.296 | TRUE | TRUE |
| CHEMBL1516176 | N[C@@H](CCS(=O)O)C(=O)O | alpha | -0.9899 | 167.186 | FALSE | TRUE |
| CHEMBL151740 | Cc1cc(CC(N)C(=O)O)cc(C)c1Oc1ccc(O)c(C(C)C)c1 | alpha | 3.87914 | 343.423 | TRUE | FALSE |
| CHEMBL1518281 | CC(C)C(NS(=O)(=O)c1ccc2c(c1)OCCCO2)C(=O)O | alpha | 1.2354 | 329.374 | TRUE | FALSE |
| CHEMBL1520850 | O=C(O)[C@@H]1CSC2(CCSC2)N1 | alpha | 0.6092 | 205.304 | FALSE | TRUE |
| CHEMBL152307 | O=CC(Cc1ccccc1)NC(CCc1ccccc1)C(=O)O | alpha | 2.4722 | 311.381 | TRUE | FALSE |
| CHEMBL1524407 | CCC(C)C(NS(=O)(=O)c1ccc(C)cc1)C(=O)O | alpha | 1.77262 | 285.365 | TRUE | FALSE |
| CHEMBL152548 | O=C(O)C1Cc2[nH]cnc2CN1 | alpha | -0.4914 | 167.168 | TRUE | FALSE |
| CHEMBL152758 | CC(N[C@H](C)C(=O)N1CCC[C@H]1C(=O)O)C(=O)O | alpha | -0.4868 | 258.274 | FALSE | TRUE |
| CHEMBL1530676 | Cc1ccc(S(=O)(=O)NC(CC(N)=O)C(=O)O)cc1 | alpha | -0.39808 | 286.309 | TRUE | FALSE |
| CHEMBL1531535 | CCCCC(NS(=O)(=O)c1ccc(OC)cc1)C(=O)O | alpha | 1.6169 | 301.364 | TRUE | FALSE |
| CHEMBL1532210 | O=C(CC(NCCc1ccccc1)C(=O)O)Nc1ccc(O)cc1 | alpha | 2.0063 | 328.368 | TRUE | FALSE |
| CHEMBL153266 | NC(=O)C(F)C(N)C(=O)O | alpha | -1.7783 | 150.109 | FALSE | FALSE |
| CHEMBL153267 | NC(CNC(=O)C(Cl)Cl)C(=O)O | alpha | -0.6818 | 215.036 | FALSE | FALSE |
| CHEMBL1533312 | NC(CSCc1cccc(Br)c1)C(=O)O | alpha | 2.0942 | 290.182 | TRUE | FALSE |
| CHEMBL1535445 | O=C(O)C1Nc2cc(C(F)(F)F)ccc2S(=O)(=O)N1 | alpha | 0.8199 | 296.226 | TRUE | FALSE |
| CHEMBL1536735 | CCOc1ccc(NC(=O)CC(NCc2ccccn2)C(=O)O)cc1 | alpha | 2.0519 | 343.383 | TRUE | FALSE |
| CHEMBL1538638 | Cc1nc(NC(CC(C)C)C(=O)O)c2nc(C)oc2n1 | alpha | 2.14584 | 278.312 | TRUE | FALSE |
| CHEMBL153945 | CNC(CC1=CCCCc2onc(O)c21)C(=O)O | alpha | 1.1626 | 252.27 | TRUE | FALSE |
| CHEMBL154076 | O=C(O)C(Cc1ccccc1)NC/C(=N\O)c1ccccn1 | alpha | 1.5454 | 299.33 | TRUE | FALSE |
| CHEMBL154147 | Cc1cc(Oc2c(C)cc(CC(N)C(=O)O)cc2C)ccc1O | alpha | 3.06416 | 315.369 | TRUE | FALSE |
| CHEMBL154191 | O=C(O)[C@@H]1CSC(c2ccccc2)N1 | alpha | 1.4748 | 209.27 | TRUE | TRUE |
| CHEMBL1544825 | CCC(NS(=O)(=O)c1ccc(OC)cc1)C(=O)O | alpha | 0.8367 | 273.31 | TRUE | FALSE |
| CHEMBL154573 | O=C(O)C1Cc2cc(O)c(O)cc2CN1 | alpha | 0.1967 | 209.201 | TRUE | FALSE |
| CHEMBL154806 | NC(CC1=CCCCc2c(O)noc21)C(=O)O | alpha | 0.9019 | 238.243 | TRUE | FALSE |
| CHEMBL154895 | CC(=N)NCCS(=O)(=O)C[C@@H](N)C(=O)O | alpha | -1.60013 | 237.281 | FALSE | TRUE |
| CHEMBL155129 | CC(=N)NCCOC[C@H](N)C(=O)O | alpha | -0.99833 | 189.215 | FALSE | TRUE |
| CHEMBL155316 | CC(=N)NCCSCC[C@@H](N)C(=O)O | alpha | 0.10837 | 219.31 | FALSE | TRUE |
| CHEMBL155318 | CC(=N)NCC[S+]([O-])CCC(N)C(=O)O | alpha | -0.87613 | 235.309 | FALSE | FALSE |
| CHEMBL155413 | CC(=N)NCCOCCC(N)C(=O)O | alpha | -0.60823 | 203.242 | FALSE | FALSE |
| CHEMBL1554290 | O=C(CC(NCc1cccnc1)C(=O)O)Nc1ccc(F)cc1 | alpha | 1.7923 | 317.32 | TRUE | FALSE |
| CHEMBL15543 | N[C@H](Cc1ccc(OCCc2ccccc2)cc1)C(=O)O | alpha | 2.2625 | 285.343 | TRUE | TRUE |
| CHEMBL1555183 | N[C@@H](CSCCCO)C(=O)O | alpha | -0.4861 | 179.241 | FALSE | TRUE |
| CHEMBL155696 | O=C(O)[C@H](CCS)NC(F)F | alpha | 0.5717 | 185.195 | FALSE | TRUE |
| CHEMBL1560345 | CN(C)CCCNC(CC(=O)Nc1cccc([N+](=O)[O-])c1)C(=O)O | alpha | 0.9179 | 338.364 | TRUE | FALSE |
| CHEMBL156069 | CC1N[C@@H](C(=O)O)CS1 | alpha | 0.122 | 147.199 | FALSE | TRUE |
| CHEMBL1561409 | Cc1cccc(C)c1NC(=O)CC(NCCc1ccccc1)C(=O)O | alpha | 2.91754 | 340.423 | TRUE | FALSE |
| CHEMBL1561474 | C[Se]C[C@@H](N)C(=O)O.Cl | alpha | -0.0093 | 218.542 | FALSE | TRUE |
| CHEMBL1561937 | NC(Cc1ccc(OC(F)F)cc1)C(=O)O | alpha | 1.2424 | 231.198 | TRUE | FALSE |
| CHEMBL1564870 | CCc1ccc(C2NC(C(=O)O)CS2)cc1 | alpha | 2.0372 | 237.324 | TRUE | FALSE |
| CHEMBL1565247 | N[C@@H](Cc1n[nH]c2ccccc12)C(=O)O | alpha | 0.5173 | 205.217 | TRUE | TRUE |
| CHEMBL1567903 | CC[C@@H](C)C1CN[C@H](C(=O)O)[C@H]1CC(=O)O | alpha | 0.796 | 229.276 | FALSE | TRUE |
| CHEMBL1570733 | CCOc1ccc(C(=O)CCNC(Cc2c[nH]cn2)C(=O)O)cc1 | alpha | 1.6667 | 331.372 | TRUE | FALSE |
| CHEMBL157074 | CC(=N)NCCCOC[C@H](N)C(=O)O | alpha | -0.60823 | 203.242 | FALSE | TRUE |
| CHEMBL1571504 | CC(C)[C@@H]1CN[C@H](C(=O)O)[C@@H]1CC(=O)O | alpha | 0.4059 | 215.249 | FALSE | TRUE |
| CHEMBL157190 | CC(=N)NCCCS(=O)(=O)C[C@H](N)C(=O)O | alpha | -1.21003 | 251.308 | FALSE | TRUE |
| CHEMBL157490 | NC(CSc1ccccc1O)C(=O)O | alpha | 0.8962 | 213.258 | TRUE | FALSE |
| CHEMBL157516 | CCCCCC1N[C@H](C(=O)O)CS1 | alpha | 1.6824 | 203.307 | FALSE | TRUE |
| CHEMBL157846 | CC(=N)NCCS(=O)(=O)CCC(N)C(=O)O | alpha | -1.21003 | 251.308 | FALSE | FALSE |
| CHEMBL157866 | NC(CC(=O)c1ccc([N+](=O)[O-])cc1)C(=O)O | alpha | 0.5795 | 238.199 | TRUE | FALSE |
| CHEMBL157958 | CC(=N)NCC[S+]([O-])C[C@@H](N)C(=O)O | alpha | -1.26623 | 221.282 | FALSE | TRUE |
| CHEMBL1580657 | CCOc1ccc(S(=O)(=O)NC(Cc2c[nH]cn2)C(=O)O)cc1 | alpha | 0.7826 | 339.373 | TRUE | FALSE |
| CHEMBL1581529 | CC(NS(=O)(=O)c1ccc2c(c1)OCCCO2)C(=O)O | alpha | 0.5993 | 301.32 | TRUE | FALSE |
| CHEMBL158250 | CCC1N[C@H](C(=O)O)CS1 | alpha | 0.5121 | 161.226 | FALSE | TRUE |
| CHEMBL158297 | CC(=N)NCCCSC[C@H](N)C(=O)O | alpha | 0.10837 | 219.31 | FALSE | TRUE |
| CHEMBL158499 | NC(CC(=O)c1ccccc1[N+](=O)[O-])C(=O)O | alpha | 0.5795 | 238.199 | TRUE | FALSE |
| CHEMBL158522 | Cc1c(O)noc1C(C)C(N)C(=O)O | alpha | 0.20402 | 200.194 | TRUE | FALSE |
| CHEMBL158798 | CC(C)[C@@H](NS(=O)(=O)c1ccc(Oc2ccccc2)cc1)C(=O)O | alpha | 2.8664 | 349.408 | TRUE | TRUE |
| CHEMBL159211 | NC(CSc1nc(CC(N)C(=O)O)c[nH]1)C(=O)O | alpha | -1.1319 | 274.302 | TRUE | FALSE |
| CHEMBL1592386 | NC(C(=O)O)C(CC(=O)O)c1ccc(Cl)cc1 | alpha | 1.3102 | 257.673 | TRUE | FALSE |
| CHEMBL159245 | NC(CSc1cc(O)c(O)cc1CC(N)C(=O)O)C(=O)O | alpha | -0.4438 | 316.335 | TRUE | FALSE |
| CHEMBL1593851 | NC(C(=O)O)C(O)c1ccc(O)c(O)c1 | alpha | -0.4569 | 213.189 | TRUE | FALSE |
| CHEMBL1598913 | O=C(O)C(Cc1c[nH]cn1)NS(=O)(=O)c1ccc(Cl)cc1 | alpha | 1.0373 | 329.765 | TRUE | FALSE |
| CHEMBL1600051 | CC(C)CC(NC1=NC(C)(C)Cc2ccccc21)C(=O)O | alpha | 2.8568 | 288.391 | TRUE | FALSE |
| CHEMBL1603145 | O=C(O)C(Cc1ccccc1)NC(=S)Nc1ccccc1 | alpha | 2.6689 | 300.383 | TRUE | FALSE |
| CHEMBL160421 | O=C(O)[C@@H](CCS)NC(F)(F)F | alpha | 0.8689 | 203.185 | FALSE | TRUE |
| CHEMBL160527 | NC(CC(=O)c1cccnc1)C(=O)O | alpha | 0.0663 | 194.19 | TRUE | FALSE |
| CHEMBL1606929 | NC(=O)CC(NSc1ccccc1[N+](=O)[O-])C(=O)O | alpha | 0.5201 | 285.281 | TRUE | FALSE |
| CHEMBL1611192 | N[C@@H](C(=O)O)c1cc(O)ccc1Cl | alpha | 1.13 | 201.609 | TRUE | TRUE |
| CHEMBL161223 | Cc1cc(O)c(O)c(SCC(N)C(=O)O)c1 | alpha | 0.91022 | 243.284 | TRUE | FALSE |
| CHEMBL161454 | NC(CSc1ccc(SCC(N)C(=O)O)c(O)c1O)C(=O)O | alpha | 0.1058 | 348.402 | TRUE | FALSE |
| CHEMBL1615355 | C=C1C[C@@H]1C[C@H](N)C(=O)O | alpha | 0.3645 | 141.17 | FALSE | TRUE |
| CHEMBL1615456 | NC(=O)C(F)[C@H](N)C(=O)O | alpha | -1.7783 | 150.109 | FALSE | TRUE |
| CHEMBL161571 | NC(CSc1ccc(O)c(O)c1)C(=O)O | alpha | 0.6018 | 229.257 | TRUE | FALSE |
| CHEMBL161578 | NC(CSc1c(CC(N)C(=O)O)ccc(O)c1O)C(=O)O | alpha | -0.4438 | 316.335 | TRUE | FALSE |
| CHEMBL1616107 | O=C(O)C1NCCc2ccccc21 | alpha | 0.958 | 177.203 | TRUE | FALSE |
| CHEMBL161671 | NC(CSc1ccc(O)cc1)C(=O)O | alpha | 0.8962 | 213.258 | TRUE | FALSE |
| CHEMBL1617579 | NC(Cc1cccc2nsnc12)C(=O)O | alpha | 0.6457 | 223.257 | TRUE | FALSE |
| CHEMBL161776 | Cc1cc(NC(C)C(=O)O)cc(C)c1Oc1ccc(O)c(C(C)C)c1 | alpha | 4.80974 | 343.423 | TRUE | FALSE |
| CHEMBL1618724 | CC(C)[C@H](Nc1ccc(C(F)(F)F)cc1[N+](=O)[O-])C(=O)O | alpha | 3.1347 | 306.24 | TRUE | TRUE |
| CHEMBL1619821 | NCCO/C=C/[C@H](N)C(=O)O | alpha | -1.1127 | 160.173 | FALSE | TRUE |
| CHEMBL1619985 | C[C@H](Nc1ccc(C(F)(F)F)cc1[N+](=O)[O-])C(=O)O | alpha | 2.4986 | 278.186 | TRUE | TRUE |
| CHEMBL162061 | O=C(O)C[C@H]1[C@@H](C(=O)O)NC[C@H]1c1ccccc1O | alpha | 0.6231 | 265.265 | TRUE | TRUE |
| CHEMBL1620859 | N[C@@H](Cc1c[nH]c2cc([N+](=O)[O-])ccc12)C(=O)O | alpha | 1.0305 | 249.226 | TRUE | TRUE |
| CHEMBL1621013 | NC(Cc1ccc2nonc2c1)C(=O)O | alpha | 0.1772 | 207.189 | TRUE | FALSE |
| CHEMBL1627202 | C[S+](=N)([O-])CC[C@H](N)C(=O)O | alpha | -0.53503 | 180.229 | FALSE | TRUE |
| CHEMBL1627263 | CCC(C[S+](=N)([O-])CCC(N)C(=O)O)C(=O)O | alpha | -0.05403 | 266.319 | FALSE | FALSE |
| CHEMBL1627290 | CCCC[S+](=N)([O-])CCC(N)C(=O)O | alpha | 0.63527 | 222.31 | FALSE | FALSE |
| CHEMBL163032 | O=C(O)CC1=C(c2ccccc2)CN[C@@H]1C(=O)O | alpha | 0.9713 | 247.25 | TRUE | TRUE |
| CHEMBL164132 | COc1cccc(C[C@@H](C[C@H](N)C(=O)O)C(=O)O)c1 | alpha | 0.7405 | 267.281 | TRUE | TRUE |
| CHEMBL164614 | N[C@@H](C[C@@H](Cc1ccc(C(F)(F)F)cc1)C(=O)O)C(=O)O | alpha | 1.7507 | 305.252 | TRUE | TRUE |
| CHEMBL164687 | O=C(O)CC1=C(c2ccoc2)CN[C@@H]1C(=O)O | alpha | 0.5643 | 237.211 | TRUE | TRUE |
| CHEMBL1650467 | N[C@@H](CC([18F])C(=O)O)C(=O)O | alpha | -0.7889 | 164.122938 | FALSE | TRUE |
| CHEMBL1650468 | N[C@@H](C[C@H](F)C(=O)O)C(=O)O | alpha | -0.7889 | 165.12 | FALSE | TRUE |
| CHEMBL1650469 | N[C@@H](C[C@@H](F)C(=O)O)C(=O)O | alpha | -0.7889 | 165.12 | FALSE | TRUE |
| CHEMBL1651882 | NCOCCC(N)C(=O)O | alpha | -1.2788 | 148.162 | FALSE | FALSE |
| CHEMBL1651883 | [N-]=[N+]=CC(=O)CCC(N)C(=O)O | alpha | -0.9518 | 171.156 | FALSE | FALSE |
| CHEMBL165500 | N[C@@H](C[C@H](CCCCCc1ccccc1)C(=O)O)C(=O)O | alpha | 2.2923 | 293.363 | TRUE | TRUE |
| CHEMBL165544 | N[C@@H](C[C@@H](CCCCc1ccccc1)C(=O)O)C(=O)O | alpha | 1.9022 | 279.336 | TRUE | TRUE |
| CHEMBL166250 | O=C(O)CC1=C(c2cccs2)CN[C@@H]1C(=O)O | alpha | 1.0328 | 253.279 | TRUE | TRUE |
| CHEMBL166303 | COc1ccccc1C1=C(CC(=O)O)[C@@H](C(=O)O)NC1 | alpha | 0.9799 | 277.276 | TRUE | TRUE |
| CHEMBL166609 | O=C(O)[C@@H]1C[C@H](CCCCP(=O)(O)O)CCN1 | alpha | 0.7872 | 265.246 | FALSE | TRUE |
| CHEMBL166907 | O=C(O)[C@H]1NCCC[C@H]1CP(=O)(O)O | alpha | -0.3831 | 223.165 | FALSE | TRUE |
| CHEMBL1669237 | C[C@@H]1ON=C(C(=O)O)[C@@H]1C[C@H](N)C(=O)O | alpha | -0.7361 | 216.193 | FALSE | TRUE |
| CHEMBL1669238 | C[C@@H]1ON=C(C(=O)O)[C@@H]1C[C@@H](N)C(=O)O | alpha | -0.7361 | 216.193 | FALSE | TRUE |
| CHEMBL1669239 | C[C@H]1C(C(=O)O)=NO[C@H]1C[C@@H](N)C(=O)O | alpha | -0.7361 | 216.193 | FALSE | TRUE |
| CHEMBL1669240 | C[C@H]1C(C(=O)O)=NO[C@H]1C[C@H](N)C(=O)O | alpha | -0.7361 | 216.193 | FALSE | TRUE |
| CHEMBL168265 | CCCCCCCCCCCCCCCCCCC(N)C(=O)O | alpha | 6.0499 | 327.553 | FALSE | FALSE |
| CHEMBL1689038 | Cl.N[C@@H](CCSCCC[18F])C(=O)O | alpha | 1.303 | 230.722938 | FALSE | TRUE |
| CHEMBL1689039 | Cl.N[C@H](CCSCCC[18F])C(=O)O | alpha | 1.303 | 230.722938 | FALSE | TRUE |
| CHEMBL169182 | O=C(O)C(CS)NCc1ccccc1 | alpha | 1.1592 | 211.286 | TRUE | FALSE |
| CHEMBL1697700 | NC(CS/C(Cl)=C/Cl)C(=O)O | alpha | 1.408 | 216.089 | FALSE | FALSE |
| CHEMBL1700010 | COc1ccc(S(=O)(=O)NC(Cc2c[nH]cn2)C(=O)O)cc1 | alpha | 0.3925 | 325.346 | TRUE | FALSE |
| CHEMBL170005 | NC(C(=O)O)C(F)C(=O)O | alpha | -1.179 | 151.093 | FALSE | FALSE |
| CHEMBL1700443 | Cc1ccc(NC(=O)CC(NCC2CCCO2)C(=O)O)cc1C | alpha | 1.85384 | 320.389 | TRUE | FALSE |
| CHEMBL1700673 | CC(C)CC(NS(=O)(=O)c1cccc(C(=O)O)c1)C(=O)O | alpha | 1.1624 | 315.347 | TRUE | FALSE |
| CHEMBL1701433 | CC(c1ccccc1)C1NC(C(=O)O)CS1 | alpha | 1.9058 | 237.324 | TRUE | FALSE |
| CHEMBL1702667 | COc1ccc(NC(=O)CC(NC(C)c2ccccc2)C(=O)O)cc1 | alpha | 2.8278 | 342.395 | TRUE | FALSE |
| CHEMBL170308 | O=C(O)[C@H](CS)NCc1ccccc1 | alpha | 1.1592 | 211.286 | TRUE | TRUE |
| CHEMBL1703170 | CCc1ccccc1NC(=O)CC(NCc1ccccc1)C(=O)O | alpha | 2.8206 | 326.396 | TRUE | FALSE |
| CHEMBL170680 | CCNC(CS)C(=O)O | alpha | -0.0211 | 149.215 | FALSE | FALSE |
| CHEMBL170775 | CCCNC(CS)C(=O)O | alpha | 0.369 | 163.242 | FALSE | FALSE |
| CHEMBL170864 | CC(=N)NC[C@@H](F)CC[C@@H](N)C(=O)O | alpha | 0.10337 | 205.233 | FALSE | TRUE |
| CHEMBL171190 | O=C(O)C(CS)NCC1CCCCC1 | alpha | 1.5393 | 217.334 | FALSE | FALSE |
| CHEMBL1713207 | CC(C)C(NS(=O)(=O)c1ccc(Br)s1)C(=O)O | alpha | 1.8981 | 342.236 | TRUE | FALSE |
| CHEMBL171327 | CC(=N)NC[C@@H](F)CC[C@H](N)C(=O)O | alpha | 0.10337 | 205.233 | FALSE | TRUE |
| CHEMBL171397 | N/C(S)=N/CCCC[C@H](N)C(=O)O | alpha | -0.1869 | 205.283 | FALSE | TRUE |
| CHEMBL171617 | O=C(O)[C@@H](CS)NCCc1ccccc1 | alpha | 1.2017 | 225.313 | TRUE | TRUE |
| CHEMBL1716222 | COc1ccccc1NC(=O)CC(NC1CCCCC1)C(=O)O | alpha | 2.3993 | 320.389 | TRUE | FALSE |
| CHEMBL1717175 | NC(C(=O)O)C1ON=C(Cl)C1O | alpha | -1.2897 | 194.574 | FALSE | FALSE |
| CHEMBL172319 | CC(C)CNC(CS)C(=O)O | alpha | 0.615 | 177.269 | FALSE | FALSE |
| CHEMBL1723908 | Cl.NC(Cc1ccc2nsnc2c1)C(=O)O | alpha | 1.0675 | 259.718 | TRUE | FALSE |
| CHEMBL1724409 | COCCCNC(=O)CC(NCCCOC)C(=O)O | alpha | -0.3915 | 276.333 | FALSE | FALSE |
| CHEMBL1727361 | CC(C)C(NS(=O)(=O)c1cc(C(=O)O)ccc1Cl)C(=O)O | alpha | 1.4257 | 335.765 | TRUE | FALSE |
| CHEMBL1727604 | CCc1ccc(NC(=O)CC(NCc2cccnc2)C(=O)O)cc1 | alpha | 2.2156 | 327.384 | TRUE | FALSE |
| CHEMBL172867 | CC(=N)NCC(F)CCC(N)C(=O)O | alpha | 0.10337 | 205.233 | FALSE | FALSE |
| CHEMBL172986 | O=C(O)[C@@H](CS)NC1CCCCC1 | alpha | 1.2917 | 203.307 | FALSE | TRUE |
| CHEMBL173031 | O=C(O)[C@H]1C[C@@H](Cc2nnn[nH]2)CCN1 | alpha | -0.805 | 211.225 | TRUE | TRUE |
| CHEMBL173032 | O=C(O)[C@@H]1C[C@H](CCc2nn[nH]n2)CCN1 | alpha | -0.4149 | 225.252 | TRUE | TRUE |
| CHEMBL173228 | CC(=N)NC[C@H](F)CC[C@@H](N)C(=O)O | alpha | 0.10337 | 205.233 | FALSE | TRUE |
| CHEMBL1735573 | Cc1ccccc1NC(=O)CC(NCc1cccnc1)C(=O)O | alpha | 1.96162 | 313.357 | TRUE | FALSE |
| CHEMBL173767 | O=C(O)[C@H](CS)NCCc1ccccc1 | alpha | 1.2017 | 225.313 | TRUE | TRUE |
| CHEMBL173847 | CCCCc1c(O)noc1C[C@@H](N)C(=O)O | alpha | 0.6772 | 228.248 | TRUE | TRUE |
| CHEMBL173916 | O=C(O)C(CS)NC1CCCCC1 | alpha | 1.2917 | 203.307 | FALSE | FALSE |
| CHEMBL173967 | O=C(O)[C@H](CS)NC1CCCCC1 | alpha | 1.2917 | 203.307 | FALSE | TRUE |
| CHEMBL174022 | COc1noc(C)c1CC(N)C(=O)O | alpha | -0.05398 | 200.194 | TRUE | FALSE |
| CHEMBL1740428 | NC(Cc1ccc2nsnc2c1)C(=O)O | alpha | 0.6457 | 223.257 | TRUE | FALSE |
| CHEMBL174065 | COc1ccc(CNC(CS)C(=O)O)cc1 | alpha | 1.1678 | 241.312 | TRUE | FALSE |
| CHEMBL174116 | O=C(O)C(CS)NCc1cccc2ccccc12 | alpha | 2.3124 | 261.346 | TRUE | FALSE |
| CHEMBL174127 | O=C(O)C(CS)NCCCc1ccccc1 | alpha | 1.5918 | 239.34 | TRUE | FALSE |
| CHEMBL1741354 | NC(=O)C[S+]([O-])C[C@H](N)C(=O)O | alpha | -2.3677 | 194.212 | FALSE | TRUE |
| CHEMBL1741404 | N[C@H](C(=O)O)[C@H](N)C(=O)O | alpha | -2.1898 | 148.118 | FALSE | TRUE |
| CHEMBL1741425 | O=C(O)C[C@H](Nc1ccccc1)C(=O)O | alpha | 1.0264 | 209.201 | TRUE | TRUE |
| CHEMBL1741564 | C[N+](C)(C)c1ccc([C@H](N)C(=O)O)cc1 | alpha | 0.9677 | 209.269 | TRUE | TRUE |
| CHEMBL1741731 | N[C@@H](CNO)C(=O)O | alpha | -1.6229 | 120.108 | FALSE | TRUE |
| CHEMBL1741817 | O=CN[C@@H](Cc1c[nH]cn1)C(=O)O | alpha | -0.8486 | 183.167 | TRUE | TRUE |
| CHEMBL1741833 | Nc1ccc(C(=O)C[C@H](N)C(=O)O)cc1O | alpha | -0.0409 | 224.216 | TRUE | TRUE |
| CHEMBL1741838 | C=Cc1ccc([C@H]2N[C@@H](C(=O)O)CC[C@@H]2C)cc1 | alpha | 2.8434 | 245.322 | TRUE | TRUE |
| CHEMBL1741839 | COc1ccc(CN[C@H](C)C(=O)O)cc1 | alpha | 1.2579 | 209.245 | TRUE | TRUE |
| CHEMBL1741957 | CC(C)(SCc1ccccc1)[C@H](N)C(=O)O | alpha | 2.1103 | 239.34 | TRUE | TRUE |
| CHEMBL1742025 | N[C@@H](CSc1nc2ccccn2c1[N+](=O)[O-])C(=O)O | alpha | 0.7465 | 282.281 | TRUE | TRUE |
| CHEMBL1742052 | N[C@@H](Cc1cccnn1)C(=O)O | alpha | -0.569 | 167.168 | TRUE | TRUE |
| CHEMBL1742319 | N[C@@H](Cc1c[nH]c2ccc([N+](=O)[O-])cc12)C(=O)O | alpha | 1.0305 | 249.226 | TRUE | TRUE |
| CHEMBL1744058 | N[C@H](/C=C/CCO)C(=O)O | alpha | -0.6631 | 145.158 | FALSE | TRUE |
| CHEMBL174406 | CC(C)N[C@H](CS)C(=O)O | alpha | 0.3674 | 163.242 | FALSE | TRUE |
| CHEMBL1767337 | O=C(O)[C@@H]1CSC(c2ccccc2O)N1 | alpha | 1.1804 | 225.269 | TRUE | TRUE |
| CHEMBL1767338 | O=C(O)c1ccccc1C1N[C@H](C(=O)O)CS1 | alpha | 1.173 | 253.279 | TRUE | TRUE |
| CHEMBL1767339 | N#Cc1ccc(C2N[C@H](C(=O)O)CS2)cc1 | alpha | 1.34648 | 234.28 | TRUE | TRUE |
| CHEMBL1767340 | O=C(O)[C@@H]1CSC(c2ccccc2[N+](=O)[O-])N1 | alpha | 1.383 | 254.267 | TRUE | TRUE |
| CHEMBL1767341 | COc1cccc(C2N[C@H](C(=O)O)CS2)c1O | alpha | 1.189 | 255.295 | TRUE | TRUE |
| CHEMBL1767342 | O=C(O)[C@@H]1CSC(c2ccco2)N1 | alpha | 1.0678 | 199.231 | TRUE | TRUE |
| CHEMBL176855 | N[C@@H](Cc1onc(O)c1-c1ccccn1)C(=O)O | alpha | 0.3966 | 249.226 | TRUE | TRUE |
| CHEMBL176910 | O=C(O)[C@H]1C[C@H](c2nnn[nH]2)CN1 | alpha | -1.2702 | 183.171 | TRUE | TRUE |
| CHEMBL177381 | Cl.N[C@@H](CCSCCC(=O)NCC(=O)O)C(=O)O | alpha | -0.4657 | 300.764 | FALSE | TRUE |
| CHEMBL1778764 | O=C(O)[C@@H]1C[C@H](Oc2cccc(Cl)c2)CN1 | alpha | 1.5339 | 241.674 | TRUE | TRUE |
| CHEMBL1778765 | Cl.O=C(O)[C@@H]1C[C@H](COc2cccc(Cl)c2)CN1 | alpha | 2.2033 | 292.162 | TRUE | TRUE |
| CHEMBL1778766 | Cl.O=C(O)[C@@H]1C[C@H](Cc2cccc(F)c2)CN1 | alpha | 1.8527 | 259.708 | TRUE | TRUE |
| CHEMBL1778767 | O=C(O)[C@@H]1C[C@H](OCc2ccc(Cl)cc2)CN1 | alpha | 1.6717 | 255.701 | TRUE | TRUE |
| CHEMBL1778768 | O=C(O)[C@@H]1C[C@H](Cc2ccccc2F)CN1 | alpha | 1.4309 | 223.247 | TRUE | TRUE |
| CHEMBL1778769 | O=C(O)[C@@H]1C[C@H](COc2cccc(F)c2)CN1 | alpha | 1.2672 | 239.246 | TRUE | TRUE |
| CHEMBL1778770 | O=C(O)[C@@H]1C[C@H](Cc2ccccc2Cl)CN1 | alpha | 1.9452 | 239.702 | TRUE | TRUE |
| CHEMBL1778771 | O=C(O)[C@@H]1C[C@H](Cc2cc(Cl)ccc2Cl)CN1 | alpha | 2.5986 | 274.147 | TRUE | TRUE |
| CHEMBL1778772 | O=C(O)[C@@H]1C[C@H](Cc2cccc(OC(F)(F)F)c2)CN1 | alpha | 2.1904 | 289.253 | TRUE | TRUE |
| CHEMBL1778773 | CCc1cccc(C[C@@H]2CN[C@H](C(=O)O)C2)c1 | alpha | 1.8542 | 233.311 | TRUE | TRUE |
| CHEMBL1778774 | O=C(O)[C@@H]1C[C@H](Cc2cccc(F)c2F)CN1 | alpha | 1.57 | 241.237 | TRUE | TRUE |
| CHEMBL1778775 | COc1cccc(C[C@@H]2CN[C@H](C(=O)O)C2)c1 | alpha | 1.3004 | 235.283 | TRUE | TRUE |
| CHEMBL1778776 | O=C(O)[C@@H]1C[C@H](Cc2cc(F)ccc2F)CN1 | alpha | 1.57 | 241.237 | TRUE | TRUE |
| CHEMBL1778777 | O=C(O)[C@@H]1C[C@H](Cc2ccccc2)CN1 | alpha | 1.2918 | 205.257 | TRUE | TRUE |
| CHEMBL1778778 | O=C(O)[C@H]1C[C@@H](Cc2cccc(F)c2)CN1 | alpha | 1.4309 | 223.247 | TRUE | TRUE |
| CHEMBL1778782 | O=C(O)[C@@H]1C[C@H](Sc2ccc(Cl)cc2)CN1 | alpha | 2.2472 | 257.742 | TRUE | TRUE |
| CHEMBL1778783 | O=C(O)[C@@H]1C[C@H](Oc2ccc(Cl)cc2)CN1 | alpha | 1.5339 | 241.674 | TRUE | TRUE |
| CHEMBL1778784 | O=C(O)[C@H]1C[C@@H](Oc2ccc(Cl)cc2)CN1 | alpha | 1.5339 | 241.674 | TRUE | TRUE |
| CHEMBL1778785 | Cl.O=C(O)[C@@H]1C[C@H](Oc2cccc(Cl)c2)CN1 | alpha | 1.9557 | 278.135 | TRUE | TRUE |
| CHEMBL1778786 | O=C(O)[C@@H]1C[C@H](Oc2ccccc2)CN1 | alpha | 0.8805 | 207.229 | TRUE | TRUE |
| CHEMBL1778787 | O=C(O)[C@@H]1C[C@H](Oc2ccc(Cl)cn2)CN1 | alpha | 0.9289 | 242.662 | TRUE | TRUE |
| CHEMBL1778790 | O=C(O)[C@@H]1C[C@H](Oc2ccc(OC(F)(F)F)cc2)CN1 | alpha | 1.7791 | 291.225 | TRUE | TRUE |
| CHEMBL1778791 | O=C(O)[C@@H]1C[C@H](Oc2cccc(F)c2)CN1 | alpha | 1.0196 | 225.219 | TRUE | TRUE |
| CHEMBL1778792 | O=C(O)[C@@H]1C[C@H](Oc2ccccc2Cl)CN1 | alpha | 1.5339 | 241.674 | TRUE | TRUE |
| CHEMBL1778793 | CCC(C)O[C@@H]1CN[C@H](C(=O)O)C1 | alpha | 0.6166 | 187.239 | FALSE | TRUE |
| CHEMBL1778871 | N#Cc1ccccc1O[C@@H]1CN[C@H](C(=O)O)C1 | alpha | 0.75218 | 232.239 | TRUE | TRUE |
| CHEMBL1778872 | O=C(O)[C@@H]1C[C@H](Oc2cccc(O)c2)CN1 | alpha | 0.5861 | 223.228 | TRUE | TRUE |
| CHEMBL1778873 | O=C(O)[C@@H]1C[C@H](Oc2ccc(Cl)c(F)c2)CN1 | alpha | 1.673 | 259.664 | TRUE | TRUE |
| CHEMBL1778874 | O=C(O)[C@@H]1C[C@H](OCc2cccc(Cl)c2)CN1 | alpha | 1.6717 | 255.701 | TRUE | TRUE |
| CHEMBL1778875 | O=C(O)[C@@H]1C[C@H](Sc2cccc(Cl)c2)CN1 | alpha | 2.2472 | 257.742 | TRUE | TRUE |
| CHEMBL178746 | O=C(O)[C@@H]1C[C@H](CCCCc2nn[nH]n2)CCN1 | alpha | 0.3653 | 253.306 | TRUE | TRUE |
| CHEMBL1789443 | O=C(O)[C@@H]1C[C@H](COc2cccc(Cl)c2)CN1 | alpha | 1.7815 | 255.701 | TRUE | TRUE |
| CHEMBL1789460 | O=C(O)[C@@H]1C[C@H](Cc2cccc(F)c2)CN1 | alpha | 1.4309 | 223.247 | TRUE | TRUE |
| CHEMBL1789992 | CC(C)[C@H](Nc1ccc(C(F)(F)F)cc1[N+](=O)[O-])C(=O)O.Cl | alpha | 3.5565 | 342.701 | TRUE | TRUE |
| CHEMBL1789997 | C[C@H](Nc1ccc(C(F)(F)F)cc1[N+](=O)[O-])C(=O)O.Cl | alpha | 2.9204 | 314.647 | TRUE | TRUE |
| CHEMBL1790042 | N[C@H](C(=O)O)[C@H](OCc1cccc2ccccc12)C(=O)O | alpha | 1.2215 | 289.287 | TRUE | TRUE |
| CHEMBL1790043 | N[C@H](C(=O)O)[C@H](OCc1ccc2ccccc2c1)C(=O)O | alpha | 1.2215 | 289.287 | TRUE | TRUE |
| CHEMBL1790044 | CO[C@H](C(=O)O)[C@H](N)C(=O)O | alpha | -1.5021 | 163.129 | FALSE | TRUE |
| CHEMBL1800368 | O=C(O)[C@@H]1CC(=S)CN1 | alpha | -0.1972 | 145.183 | FALSE | TRUE |
| CHEMBL1807413 | CSCC[C@H](N/C=C1\C(=O)NN=C1c1ccccc1)C(=O)O | alpha | 1.2003 | 319.386 | TRUE | TRUE |
| CHEMBL1807414 | CSC[C@H](N/C=C1\C(=O)NN=C1c1ccccc1)C(=O)O | alpha | 0.8102 | 305.359 | TRUE | TRUE |
| CHEMBL1807415 | O=C1NN=C(c2ccccc2)/C1=C/N[C@@H](CO)C(=O)O | alpha | -0.5605 | 275.264 | TRUE | TRUE |
| CHEMBL180822 | N[C@H](C(=O)O)[C@@H]1C(C(=O)O)[C@@H]1CO | alpha | -1.6626 | 189.167 | FALSE | TRUE |
| CHEMBL1812596 | N[C@@H](Cn1c(=O)[nH]c(=O)c2cscc21)C(=O)O | alpha | -0.8368 | 255.255 | TRUE | TRUE |
| CHEMBL1812598 | N[C@@H](Cn1c(=O)[nH]c(=O)c2ccsc21)C(=O)O | alpha | -0.8368 | 255.255 | TRUE | TRUE |
| CHEMBL1812661 | N[C@@H](CCCCB(O)O)C(=O)O | alpha | -0.9586 | 174.993 | FALSE | TRUE |
| CHEMBL1818667 | N[C@@H](CCSC[C@H]1NC[C@H](O)[C@@H]1O)C(=O)O | alpha | -1.7848 | 250.32 | FALSE | TRUE |
| CHEMBL1818670 | N[C@@H](CCSC[C@H]1NC(=O)[C@H](O)[C@@H]1O)C(=O)O | alpha | -2.2582 | 264.303 | FALSE | TRUE |
| CHEMBL1818671 | NC(CCSC[C@@H]1CCC(=O)N1)C(=O)O | alpha | -0.1998 | 232.305 | FALSE | TRUE |
| CHEMBL1818672 | N[C@@H](CCSC[C@@H]1CCC(O)N1)C(=O)O | alpha | -0.408 | 234.321 | FALSE | TRUE |
| CHEMBL1818673 | CC1=N[C@H](CSCCC(N)C(=O)O)[C@@H](O)[C@H]1O | alpha | -0.9135 | 262.331 | FALSE | TRUE |
| CHEMBL1834159 | N[C@@H](CCCCC(=O)C(F)(F)F)C(=O)O | alpha | 1.0901 | 227.182 | FALSE | TRUE |
| CHEMBL1834160 | N[C@@H](CCCCC=O)C(=O)O | alpha | 0.1576 | 159.185 | FALSE | TRUE |
| CHEMBL1835329 | NC(Cc1ccccc1CCC(=O)O)C(=O)O | alpha | 0.6582 | 237.255 | TRUE | FALSE |
| CHEMBL1835330 | Cc1ccc(CCC(=O)O)c(CC(N)C(=O)O)c1 | alpha | 0.96662 | 251.282 | TRUE | FALSE |
| CHEMBL1835331 | CC(C)(C)c1ccc(CCC(=O)O)c(CC(N)C(=O)O)c1 | alpha | 1.9557 | 293.363 | TRUE | FALSE |
| CHEMBL1835332 | NC(Cc1cc(Cl)ccc1CCC(=O)O)C(=O)O | alpha | 1.3116 | 271.7 | TRUE | FALSE |
| CHEMBL1835333 | NC(Cc1cc(CO)ccc1CCC(=O)O)C(=O)O | alpha | 0.1505 | 267.281 | TRUE | FALSE |
| CHEMBL1835334 | NC(Cc1cc(Cl)c(Cl)cc1CCC(=O)O)C(=O)O | alpha | 1.965 | 306.145 | TRUE | FALSE |
| CHEMBL1835335 | NC(Cc1c(CCC(=O)O)ccc(Cl)c1Cl)C(=O)O | alpha | 1.965 | 306.145 | TRUE | FALSE |
| CHEMBL1835339 | Nc1cc(CCC(=O)O)c(CC(N)C(=O)O)cc1Cl | alpha | 0.8938 | 286.715 | TRUE | FALSE |
| CHEMBL1836522 | COc1nc(N[C@H](CCC(=O)O)C(=O)O)nc(OC)n1 | alpha | -0.3813 | 286.244 | TRUE | TRUE |
| CHEMBL184207 | N[C@@H](Cc1ccc(OCP(=O)(O)O)cc1)C(=O)O | alpha | 0.155 | 275.197 | TRUE | TRUE |
| CHEMBL18439 | C[C@H](Nc1ccc(P(=O)(O)O)cc1)C(=O)O | alpha | 0.3746 | 245.171 | TRUE | TRUE |
| CHEMBL1851934 | N=C(N)NC[C@H]1C[C@H]1[C@H](N)C(=O)O | alpha | -1.48253 | 186.215 | FALSE | TRUE |
| CHEMBL1852147 | N=C(N)NC[C@H]1C[C@@H]1[C@@H](N)C(=O)O | alpha | -1.48253 | 186.215 | FALSE | TRUE |
| CHEMBL1852151 | N=C(N)NC[C@@H]1C[C@@H]1[C@@H](N)C(=O)O | alpha | -1.48253 | 186.215 | FALSE | TRUE |
| CHEMBL1852272 | N=C(N)NC[C@@H]1C[C@H]1[C@H](N)C(=O)O | alpha | -1.48253 | 186.215 | FALSE | TRUE |
| CHEMBL1852275 | N=C(N)NC[C@H]1C[C@@H]1[C@H](N)C(=O)O | alpha | -1.48253 | 186.215 | FALSE | TRUE |
| CHEMBL1852700 | N=C(N)NC[C@H]1C[C@H]1[C@@H](N)C(=O)O | alpha | -1.48253 | 186.215 | FALSE | TRUE |
| CHEMBL1852703 | N=C(N)NC[C@@H]1C[C@H]1[C@@H](N)C(=O)O | alpha | -1.48253 | 186.215 | FALSE | TRUE |
| CHEMBL1852841 | N=C(N)NC[C@@H]1C[C@@H]1[C@H](N)C(=O)O | alpha | -1.48253 | 186.215 | FALSE | TRUE |
| CHEMBL185334 | N[C@H](CC(=O)c1ccccn1)C(=O)O | alpha | 0.0663 | 194.19 | TRUE | TRUE |
| CHEMBL185388 | N[C@H](CC(=O)c1cccs1)C(=O)O | alpha | 0.7328 | 199.231 | TRUE | TRUE |
| CHEMBL185794 | NNC(Cc1c[nH]c2ccccc12)C(=O)O | alpha | 0.6269 | 219.244 | TRUE | FALSE |
| CHEMBL1863629 | O=C(O)C1CC(CCCP(=O)(O)O)CCN1 | alpha | 0.3971 | 251.219 | FALSE | FALSE |
| CHEMBL186427 | N[C@H](Cc1ccc(OCP(=O)(O)O)cc1)C(=O)O | alpha | 0.155 | 275.197 | TRUE | TRUE |
| CHEMBL1868213 | COc1ccc(S(=O)(=O)N[C@@H](CC(C)C)C(=O)O)cc1 | alpha | 1.4728 | 301.364 | TRUE | TRUE |
| CHEMBL1872677 | O=C(O)C(Cc1ccccc1)NS(=O)(=O)c1ccc(Cl)cc1 | alpha | 2.3142 | 339.8 | TRUE | FALSE |
| CHEMBL187337 | O=C(O)[C@@H]1C[C@H](O)CCN1 | alpha | -0.8161 | 145.158 | FALSE | TRUE |
| CHEMBL1876970 | COc1ccc(S(=O)(=O)NC(Cc2ccccc2)C(=O)O)cc1 | alpha | 1.6694 | 335.381 | TRUE | FALSE |
| CHEMBL1877864 | O=C(O)C(NS(=O)(=O)c1ccccc1)C(O)c1ccccc1 | alpha | 1.1517 | 321.354 | TRUE | FALSE |
| CHEMBL1883728 | Cc1cc(NC(CC(C)C)C(=O)O)nc(Cc2ccccc2)n1 | alpha | 3.28702 | 313.401 | TRUE | FALSE |
| CHEMBL1884415 | C[S+]([O-])CCC(NS(=O)(=O)c1ccccc1)C(=O)O | alpha | 0.1867 | 305.377 | TRUE | FALSE |
| CHEMBL1885327 | CC(NS(=O)(=O)c1ccc2nc(O)c(O)nc2c1)C(=O)O | alpha | -0.2076 | 313.291 | TRUE | FALSE |
| CHEMBL189209 | COCC/N=C(\N)NCC[C@H](N)C(=O)O | alpha | -1.6609 | 218.257 | FALSE | TRUE |
| CHEMBL1893138 | O=C(O)[C@H](Cc1ccccc1)Nc1ccc([N+](=O)[O-])cn1 | alpha | 2.0976 | 287.275 | TRUE | TRUE |
| CHEMBL189477 | C/C(=C\[C@@H](N)C(=O)O)CP(=O)(O)O | alpha | -0.4777 | 209.138 | FALSE | TRUE |
| CHEMBL1898424 | CCCC(=O)C1=C(NC(C)C(=O)O)CC(c2ccccc2)CC1=O | alpha | 2.8191 | 329.396 | TRUE | FALSE |
| CHEMBL189986 | N/C(=N\CC[C@H](N)C(=O)O)N1CCCCC1 | alpha | -0.411 | 228.296 | FALSE | TRUE |
| CHEMBL1901207 | CSCC(NS(=O)(=O)c1ccc(C)cc1)C(=O)O | alpha | 1.08952 | 289.378 | TRUE | FALSE |
| CHEMBL190719 | CN(C)C(=N)NCC[C@H](N)C(=O)O | alpha | -1.12563 | 188.231 | FALSE | TRUE |
| CHEMBL1907771 | O=C(O)[C@H](NS(=O)(=O)c1ccc(Cl)cc1)c1ccccc1 | alpha | 2.4442 | 325.773 | TRUE | TRUE |
| CHEMBL1907772 | Cc1ccc(S(=O)(=O)N[C@H](C(=O)O)c2ccccc2)cc1 | alpha | 2.09922 | 305.355 | TRUE | TRUE |
| CHEMBL1907773 | O=C(O)[C@@H](NS(=O)(=O)c1ccc([N+](=O)[O-])cc1)c1ccccc1 | alpha | 1.699 | 336.325 | TRUE | TRUE |
| CHEMBL1907775 | COc1ccc(S(=O)(=O)N[C@H](C(=O)O)c2ccccc2)cc1 | alpha | 1.7994 | 321.354 | TRUE | TRUE |
| CHEMBL1907776 | O=C(O)[C@@H](NS(=O)(=O)c1ccccc1)c1ccccc1 | alpha | 1.7908 | 291.328 | TRUE | TRUE |
| CHEMBL1907813 | N[C@@H](Cc1cc(CP(=O)(O)O)cc(-c2ccccc2)c1)C(=O)O | alpha | 1.9857 | 335.296 | TRUE | TRUE |
| CHEMBL1907949 | O=C(O)[C@H](NS(=O)(=O)c1ccc([N+](=O)[O-])cc1)c1ccccc1 | alpha | 1.699 | 336.325 | TRUE | TRUE |
| CHEMBL1907950 | O=C(O)[C@H](NS(=O)(=O)c1ccc(F)cc1)c1ccccc1 | alpha | 1.9299 | 309.318 | TRUE | TRUE |
| CHEMBL1907951 | O=C(O)[C@@H](NS(=O)(=O)c1ccc(Cl)cc1)c1ccccc1 | alpha | 2.4442 | 325.773 | TRUE | TRUE |
| CHEMBL1907952 | Cc1ccc(S(=O)(=O)N[C@@H](C(=O)O)c2ccccc2)cc1 | alpha | 2.09922 | 305.355 | TRUE | TRUE |
| CHEMBL1907954 | COc1ccc(S(=O)(=O)N[C@@H](C(=O)O)c2ccccc2)cc1 | alpha | 1.7994 | 321.354 | TRUE | TRUE |
| CHEMBL1907955 | O=C(O)[C@H](NS(=O)(=O)c1ccccc1)c1ccccc1 | alpha | 1.7908 | 291.328 | TRUE | TRUE |
| CHEMBL190819 | CC(=O)N[C@@H](CC(C)C)[C@@H]1N[C@@H](C(=O)O)C[C@H]1c1ccno1 | alpha | 1.1241 | 309.366 | TRUE | TRUE |
| CHEMBL1908881 | NC(Cc1ccc(O)c(CCF)c1)C(=O)O | alpha | 0.8586 | 227.235 | TRUE | FALSE |
| CHEMBL1908882 | COc1ccc(CC(N)C(=O)O)cc1CCF | alpha | 1.1616 | 241.262 | TRUE | FALSE |
| CHEMBL1908888 | COc1ccc(CC(N)C(=O)O)cc1CCCF | alpha | 1.5517 | 255.289 | TRUE | FALSE |
| CHEMBL1908892 | N[C@@H](Cc1ccc(OCCF)cc1)C(=O)O | alpha | 0.9893 | 227.235 | TRUE | TRUE |
| CHEMBL1908903 | NC(Cc1ccc(O)c(CCCF)c1)C(=O)O | alpha | 1.2487 | 241.262 | TRUE | FALSE |
| CHEMBL1908914 | N[C@@H](CCSCCCF)C(=O)O | alpha | 0.8812 | 195.259 | FALSE | TRUE |
| CHEMBL1908915 | N[C@H](CCSCCCF)C(=O)O | alpha | 0.8812 | 195.259 | FALSE | TRUE |
| CHEMBL1908916 | N[C@@H](CC(F)C(=O)O)C(=O)O | alpha | -0.7889 | 165.12 | FALSE | TRUE |
| CHEMBL1908921 | N[C@@H](Cc1ccc(O)cc1CCF)C(=O)O | alpha | 0.8586 | 227.235 | TRUE | TRUE |
| CHEMBL1909076 | N[C@H](CSC(=S)NCc1ccccc1)C(=O)O | alpha | 1.2062 | 270.379 | TRUE | TRUE |
| CHEMBL19096 | CCOCC1[C@H](C(=O)O)CN[C@H]1C(=O)O | alpha | -0.6037 | 217.221 | FALSE | TRUE |
| CHEMBL1911372 | N[C@@H](CNC(=O)CBr)C(=O)O | alpha | -1.0906 | 225.042 | FALSE | TRUE |
| CHEMBL191512 | CC(=O)N[C@@H](CC(C)C)[C@@H]1N[C@@H](C(=O)O)C[C@H]1C=C(C)C | alpha | 1.9347 | 296.411 | FALSE | TRUE |
| CHEMBL191599 | O=C(O)[C@H]1NCCN(C(=O)c2ccccc2)[C@H]1C(=O)O | alpha | -0.3616 | 278.264 | TRUE | TRUE |
| CHEMBL19206 | O=C(O)[C@@H]1CN[C@@H](C(=O)O)C1 | alpha | -0.8663 | 159.141 | FALSE | TRUE |
| CHEMBL1922424 | C[C@H](N)P(=O)(O)N[C@H](C)C(=O)O | alpha | -0.4608 | 196.143 | FALSE | TRUE |
| CHEMBL1922533 | CC(C)[C@@H](NP(=O)(O)[C@H](C)N)C(=O)O | alpha | 0.1753 | 224.197 | FALSE | TRUE |
| CHEMBL1922534 | CC(C)C[C@@H](NP(=O)(O)[C@H](C)N)C(=O)O | alpha | 0.5654 | 238.224 | FALSE | TRUE |
| CHEMBL1922535 | C[C@H](N)P(=O)(O)N[C@H](Cc1ccccc1)C(=O)O | alpha | 0.762 | 272.241 | TRUE | TRUE |
| CHEMBL192279 | CC(=O)N[C@@H](CC(C)C)[C@@H]1N[C@@H](C(=O)O)C[C@H]1/C=C/Cl | alpha | 1.721 | 302.802 | FALSE | TRUE |
| CHEMBL192497 | CCC(=O)[C@@H]1C[C@H](C(=O)O)N[C@H]1[C@H](CC(C)C)NC(C)=O | alpha | 0.9476 | 298.383 | FALSE | TRUE |
| CHEMBL1927188 | Cc1ccc(NC(=O)CC(NCCc2ccccc2)C(=O)O)cc1C | alpha | 2.91754 | 340.423 | TRUE | FALSE |
| CHEMBL192830 | CC(C)OCC/N=C(\N)NCC[C@H](N)C(=O)O | alpha | -0.8823 | 246.311 | FALSE | TRUE |
| CHEMBL19294 | O=C(O)C[C@@H]1CN[C@H](C(=O)O)C1 | alpha | -0.4762 | 173.168 | FALSE | TRUE |
| CHEMBL193087 | CC(C)n1nnc(-c2onc(O)c2CC(N)C(=O)O)n1 | alpha | -0.431 | 282.26 | TRUE | FALSE |
| CHEMBL194226 | CCn1nnnc1-c1onc(O)c1CC(N)C(=O)O | alpha | -0.992 | 268.233 | TRUE | FALSE |
| CHEMBL1945652 | CCCC(SC[C@H](N)C(=O)O)(c1ccccc1)c1ccccc1 | alpha | 3.8754 | 329.465 | TRUE | TRUE |
| CHEMBL19489 | O=C(O)[C@@H]1CN[C@H](C(=O)O)C1 | alpha | -0.8663 | 159.141 | FALSE | TRUE |
| CHEMBL194991 | NC(C[C@@H](N)N1CCCCC1)C(=O)O | alpha | -0.4409 | 201.27 | FALSE | TRUE |
| CHEMBL195156 | CC(=O)N[C@@H](CC(C)C)[C@@H]1N[C@@H](C(=O)O)C[C@H]1/C=C\Cl | alpha | 1.721 | 302.802 | FALSE | TRUE |
| CHEMBL195288 | CC(=O)N[C@@H](CC(C)C)[C@@H]1N[C@@H](C(=O)O)C[C@H]1c1ncc[nH]1 | alpha | 0.8592 | 308.382 | TRUE | TRUE |
| CHEMBL195823 | COC[C@@H]1C[C@H](C(=O)O)N[C@H]1[C@H](CC(C)C)NC(C)=O | alpha | 0.6149 | 286.372 | FALSE | TRUE |
| CHEMBL195871 | CC(=O)N[C@@H](CC(C)C)[C@@H]1N[C@@H](C(=O)O)C[C@H]1c1c[nH]cn1 | alpha | 0.8592 | 308.382 | TRUE | TRUE |
| CHEMBL1962888 | CCOP(=O)(O)C(N[C@@H](C)C(=O)O)c1ccccc1O | alpha | 1.6754 | 303.251 | TRUE | TRUE |
| CHEMBL1962978 | CCOP(=O)(O)C(N[C@@H](CS)C(=O)O)c1ccccc1O | alpha | 1.5853 | 335.318 | TRUE | TRUE |
| CHEMBL1965663 | NC(CC1=CCC=CC1)C(=O)O | alpha | 1.0648 | 167.208 | FALSE | FALSE |
| CHEMBL1965665 | COc1ccc(CNC(C)C(=O)O)cc1 | alpha | 1.2579 | 209.245 | TRUE | FALSE |
| CHEMBL1965678 | CC(C)C(Nc1ncnc2nc[nH]c12)C(=O)O | alpha | 0.874 | 235.247 | TRUE | FALSE |
| CHEMBL1966006 | CC1(C)Oc2ccc(NC(=O)CCC(N)C(=O)O)cc2O1 | alpha | 1.3245 | 294.307 | TRUE | FALSE |
| CHEMBL1966253 | N[C@H](C(=O)O)[C@@H](Cl)CC(=O)O | alpha | -0.5196 | 181.575 | FALSE | TRUE |
| CHEMBL1966574 | O=C(O)[C@@H]1CCS(=O)(=O)N1 | alpha | -1.2373 | 165.17 | FALSE | TRUE |
| CHEMBL1966733 | NC(CCc1ncc(C(=O)O)s1)C(=O)O | alpha | 0.1858 | 230.245 | TRUE | FALSE |
| CHEMBL1966949 | C#CCNC(=O)CCC(N)C(=O)O | alpha | -1.0721 | 184.195 | FALSE | FALSE |
| CHEMBL1967253 | NC(CCCNCc1ccccc1)C(=O)O | alpha | 0.9683 | 222.288 | TRUE | FALSE |
| CHEMBL1967379 | NC(Cc1ccc(OCc2ccccc2)cn1)C(=O)O | alpha | 1.615 | 272.304 | TRUE | FALSE |
| CHEMBL1968280 | Nc1ccc(C(=O)CC(N)C(=O)O)cc1O | alpha | -0.0409 | 224.216 | TRUE | FALSE |
| CHEMBL1968841 | Cc1ccc(S(=O)(=O)NC(C)C(=O)O)cc1 | alpha | 0.74642 | 243.284 | TRUE | FALSE |
| CHEMBL1969000 | CC(NS(=O)(=O)c1ccccc1)C(=O)O | alpha | 0.438 | 229.257 | TRUE | FALSE |
| CHEMBL196956 | CC(NS(=O)(=O)C(C)N)C(=O)O | alpha | -1.3164 | 196.228 | FALSE | FALSE |
| CHEMBL1970091 | Cc1cc(=O)oc2c(CNC(C(=O)O)C(C)O)c(O)ccc12 | alpha | 0.73062 | 307.302 | TRUE | FALSE |
| CHEMBL1970553 | N[C@H](CCC(=O)NCP(=O)(O)O)C(=O)O | alpha | -1.5701 | 240.152 | FALSE | TRUE |
| CHEMBL1971044 | NC(CCc1nc(-c2ccc(Cl)cc2)cs1)C(=O)O | alpha | 2.808 | 296.779 | TRUE | FALSE |
| CHEMBL1971558 | CC1C=CC(C(N)C(=O)O)O1 | alpha | -0.2582 | 157.169 | FALSE | FALSE |
| CHEMBL197176 | CC(C)CC[C@H](C[C@H](N)C(=O)O)C(=O)O | alpha | 0.9254 | 217.265 | FALSE | TRUE |
| CHEMBL1971775 | N[C@H](C(=O)O)[C@@H]1CC(Br)=NO1 | alpha | -0.1044 | 223.026 | FALSE | TRUE |
| CHEMBL1972150 | NC(CCP(=O)(O)CCC1C=CC=CC1)C(=O)O | alpha | 1.5812 | 273.269 | FALSE | FALSE |
| CHEMBL197234 | C#CCNC(=N)NCCC[C@H](N)C(=O)O | alpha | -1.07433 | 212.253 | FALSE | TRUE |
| CHEMBL1972647 | NCCO/C=C/C(N)C(=O)O | alpha | -1.1127 | 160.173 | FALSE | FALSE |
| CHEMBL1973093 | CNC(=O)SCC(N)C(=O)O | alpha | -0.5291 | 178.213 | FALSE | FALSE |
| CHEMBL1973247 | NC(CCSCC(F)F)C(=O)O | alpha | 0.7867 | 199.222 | FALSE | FALSE |
| CHEMBL1973933 | NC(CCc1ncc(-c2ccccc2)s1)C(=O)O | alpha | 2.1546 | 262.334 | TRUE | FALSE |
| CHEMBL19742 | O=C(O)C[C@H]1CCN[C@@H]1C(=O)O | alpha | -0.4762 | 173.168 | FALSE | TRUE |
| CHEMBL1974555 | Cc1cc(=O)oc2c(CNC(CO)C(=O)O)c(O)ccc12 | alpha | 0.34212 | 293.275 | TRUE | FALSE |
| CHEMBL1976323 | CC(NS(=O)(=O)c1ccc([N+](=O)[O-])cc1)C(=O)O | alpha | 0.3462 | 274.254 | TRUE | FALSE |
| CHEMBL1976865 | NC(CSS(=O)(=O)c1ccccc1)C(=O)O | alpha | 0.5204 | 261.324 | TRUE | FALSE |
| CHEMBL1978052 | O=C(O)[C@H](Cc1ccccc1)NCCN1CCC[C@H]1C(=O)O | alpha | 0.8209 | 306.362 | TRUE | TRUE |
| CHEMBL1978685 | NC(CCCNCc1ccccc1O)C(=O)O | alpha | 0.6739 | 238.287 | TRUE | FALSE |
| CHEMBL1979499 | C#CCNC(CSSCC(N)C(=O)O)C(=O)O | alpha | -0.5443 | 278.355 | FALSE | FALSE |
| CHEMBL197976 | N[C@H](C(=O)O)[C@H]1C[C@@H]1P(=O)(O)O | alpha | -1.0355 | 195.111 | FALSE | TRUE |
| CHEMBL1979899 | CC(C)(SCc1ccccc1)C(N)C(=O)O.Cl | alpha | 2.5321 | 275.801 | TRUE | FALSE |
| CHEMBL1980108 | NC(Cc1cccnn1)C(=O)O | alpha | -0.569 | 167.168 | TRUE | FALSE |
| CHEMBL198025 | CC(C)[C@@H](NS(=O)(=O)c1ccc(-c2ccc(O)cc2)cc1)C(=O)O | alpha | 2.4467 | 349.408 | TRUE | TRUE |
| CHEMBL1980324 | Cl.NC(CCC(=O)c1ccccn1)C(=O)O | alpha | 0.8782 | 244.678 | TRUE | FALSE |
| CHEMBL1981261 | NC(CSc1nc2ccccn2c1[N+](=O)[O-])C(=O)O | alpha | 0.7465 | 282.281 | TRUE | FALSE |
| CHEMBL1981355 | C#CCNC(Cc1c[nH]cn1)C(=O)O | alpha | -0.3718 | 193.206 | TRUE | FALSE |
| CHEMBL198142 | NC(C(=O)O)C1(S)CCCC1 | alpha | 0.6409 | 175.253 | FALSE | FALSE |
| CHEMBL198184 | C[C@@H](NS(=O)(=O)c1ccc(-c2ccccc2)cc1)C(=O)O | alpha | 2.105 | 305.355 | TRUE | TRUE |
| CHEMBL1983099 | Cl.NC(CCCNc1ccc(O)cc1)C(=O)O | alpha | 1.418 | 260.721 | TRUE | FALSE |
| CHEMBL198315 | NC(CC(=O)Nc1ccc(-c2ccccc2)cc1)C(=O)O | alpha | 2.0941 | 284.315 | TRUE | FALSE |
| CHEMBL1983258 | CCCCCCCCCCCCP(=O)(O)OCC(N)C(=O)O.N | alpha | 3.6832 | 354.428 | FALSE | FALSE |
| CHEMBL1983612 | NC(=O)C[S+]([O-])CC(N)C(=O)O | alpha | -2.3677 | 194.212 | FALSE | FALSE |
| CHEMBL1983936 | C[C@@H](NS(=O)(=O)c1ccc(NC(=O)c2ccccc2)cc1)C(=O)O | alpha | 1.6903 | 348.38 | TRUE | TRUE |
| CHEMBL1984151 | NC(=O)c1nc[nH]c1/N=N/SCC(N)C(=O)O | alpha | -0.3476 | 258.263 | TRUE | FALSE |
| CHEMBL1984528 | NC(CCOCCP(=O)(O)O)C(=O)O | alpha | -1.0173 | 227.153 | FALSE | FALSE |
| CHEMBL198511 | N[C@@H](Cn1ccc(=O)n(CCCC(=O)O)c1=O)C(=O)O | alpha | -1.7133 | 285.256 | TRUE | TRUE |
| CHEMBL1985244 | Cl.NC(CCCNc1cccc(O)c1)C(=O)O | alpha | 1.418 | 260.721 | TRUE | FALSE |
| CHEMBL1985275 | NC(CCCCCP(=O)(c1ccccc1)C1C=CC=CC1)C(=O)O | alpha | 3.532 | 347.395 | TRUE | FALSE |
| CHEMBL1985401 | NC(CSCc1ccc(I)cc1)C(=O)O | alpha | 1.9363 | 337.182 | TRUE | FALSE |
| CHEMBL1985424 | NC(CCOCCP(=O)(O)CCC1C=CC=CC1)C(=O)O | alpha | 1.5978 | 317.322 | FALSE | FALSE |
| CHEMBL1986009 | NC(C(=O)O)C(N)C(=O)O | alpha | -2.1898 | 148.118 | FALSE | FALSE |
| CHEMBL198618 | N[C@@H](C[C@@H](Cc1ccccc1)C(=O)O)C(=O)O | alpha | 0.7319 | 237.255 | TRUE | TRUE |
| CHEMBL198632 | Cn1c(=O)ccn(C[C@H](N)C(=O)O)c1=O | alpha | -2.0411 | 213.193 | TRUE | TRUE |
| CHEMBL1986963 | C#CCNC(CCCNC(=N)N)C(=O)O | alpha | -1.07433 | 212.253 | FALSE | FALSE |
| CHEMBL1987288 | Cc1cn(CCC(N)C(=O)O)c(=O)[nH]c1=O | alpha | -1.35298 | 227.22 | TRUE | FALSE |
| CHEMBL1987761 | C/C(=N\NC(=S)NC(CCC(=O)O)C(=O)O)c1ccccn1 | alpha | 0.5877 | 324.362 | TRUE | FALSE |
| CHEMBL1988193 | Cc1ccc(S(=O)(=O)NCCCCC(N)C(=O)O)cc1 | alpha | 0.85552 | 300.38 | TRUE | FALSE |
| CHEMBL1988336 | NC(CCc1nc(-c2cccc([N+](=O)[O-])c2)cs1)C(=O)O | alpha | 2.0628 | 307.331 | TRUE | FALSE |
| CHEMBL1988651 | NC(CCCC(N)P(=O)(O)O)C(=O)O | alpha | -0.9689 | 226.169 | FALSE | FALSE |
| CHEMBL1988860 | NC(Cc1n[nH]c2ccccc12)C(=O)O | alpha | 0.5173 | 205.217 | TRUE | FALSE |
| CHEMBL1989357 | N[C@@H](CCC(=O)NCP(=O)(O)O)C(=O)O | alpha | -1.5701 | 240.152 | FALSE | TRUE |
| CHEMBL1990458 | NC(Cc1ccnnc1)C(=O)O | alpha | -0.569 | 167.168 | TRUE | FALSE |
| CHEMBL1991067 | NC(CCSCCF)C(=O)O | alpha | 0.4911 | 181.232 | FALSE | FALSE |
| CHEMBL1991096 | NC(Cc1c[nH]c2cc([N+](=O)[O-])ccc12)C(=O)O.O=[N+]([O-])O | alpha | 0.6828 | 312.238 | TRUE | FALSE |
| CHEMBL1991156 | C[C@H](NS(=O)(=O)c1ccc(NC(=O)c2ccccc2)cc1)C(=O)O | alpha | 1.6903 | 348.38 | TRUE | TRUE |
| CHEMBL1991295 | N[C@H](C(=O)O)[C@H]1ON=C(Cl)[C@@H]1O | alpha | -1.2897 | 194.574 | FALSE | TRUE |
| CHEMBL1991454 | CC(C)CC(Nc1c(O)ccc2ccccc12)C(=O)O | alpha | 3.4566 | 273.332 | TRUE | FALSE |
| CHEMBL1991584 | NC(CNO)C(=O)O | alpha | -1.6229 | 120.108 | FALSE | FALSE |
| CHEMBL1991668 | CC(NS(=O)(=O)c1ccc(Cl)cc1)C(=O)O | alpha | 1.0914 | 263.702 | TRUE | FALSE |
| CHEMBL1991850 | N[C@@H](C(=O)O)[C@@H]1CC(Br)=NO1 | alpha | -0.1044 | 223.026 | FALSE | TRUE |
| CHEMBL1991937 | C/C(=N\NC(=S)NC(CCC(=O)O)C(=O)O)c1cnccn1 | alpha | -0.0173 | 325.35 | TRUE | FALSE |
| CHEMBL1992207 | Cc1ccc(S(=O)(=O)NC(CN)C(=O)O)cc1 | alpha | -0.31478 | 258.299 | TRUE | FALSE |
| CHEMBL199232 | N[C@@H](Cn1ccc(=O)n(CC(=O)O)c1=O)C(=O)O | alpha | -2.4935 | 257.202 | TRUE | TRUE |
| CHEMBL199233 | N[C@@H](Cn1c(=O)ccn(CC(=O)O)c1=O)C(=O)O | alpha | -2.4935 | 257.202 | TRUE | TRUE |
| CHEMBL1993059 | N[C@H](C(=O)O)[C@H](Cl)CC(=O)O | alpha | -0.5196 | 181.575 | FALSE | TRUE |
| CHEMBL1993242 | CC(Nc1ccccc1)C(=O)O | alpha | 1.5716 | 165.192 | TRUE | FALSE |
| CHEMBL199331 | O=C(O)[C@H]1CC2CC1[C@@H](C(=O)O)N2 | alpha | -0.4778 | 185.179 | FALSE | TRUE |
| CHEMBL1993905 | Cc1cc(=O)oc2c(CNC(C)C(=O)O)c(O)ccc12 | alpha | 1.36972 | 277.276 | TRUE | FALSE |
| CHEMBL1994418 | NC(CCCCNCc1ccccc1O)C(=O)O | alpha | 1.064 | 252.314 | TRUE | FALSE |
| CHEMBL1995135 | NC(CCc1nc(-c2ccccc2)cs1)C(=O)O | alpha | 2.1546 | 262.334 | TRUE | FALSE |
| CHEMBL1995372 | CC(NC(=N)N)C(=O)O | alpha | -1.05743 | 131.135 | FALSE | FALSE |
| CHEMBL1995504 | NC(Cc1cccc([N+](=O)[O-])c1)C(=O)O | alpha | 0.5492 | 210.189 | TRUE | FALSE |
| CHEMBL199564 | CC(C)[C@H](C[C@H](N)C(=O)O)C(=O)O | alpha | 0.1452 | 189.211 | FALSE | TRUE |
| CHEMBL199617 | CC(C)C[C@H](C[C@H](N)C(=O)O)C(=O)O | alpha | 0.5353 | 203.238 | FALSE | TRUE |
| CHEMBL199626 | N[C@H](C(=O)O)[C@@H]1C[C@H]1P(=O)(O)O | alpha | -1.0355 | 195.111 | FALSE | TRUE |
| CHEMBL1996562 | C#CCNC(Cc1ccccc1)C(=O)O | alpha | 0.9051 | 203.241 | TRUE | FALSE |
| CHEMBL1996598 | Cl.NC(CNc1ccc(O)cc1)C(=O)O | alpha | 0.6378 | 232.667 | TRUE | FALSE |
| CHEMBL1996668 | NC(CCCCCCCCCCP(=O)(O)O)C(=O)O | alpha | 2.0869 | 295.316 | FALSE | FALSE |
| CHEMBL1997465 | CC(NCCS(=O)(=O)O)C(=O)O | alpha | -1.0631 | 197.212 | FALSE | FALSE |
| CHEMBL1997474 | O=C(O)CC(Nc1ccccc1)C(=O)O | alpha | 1.0264 | 209.201 | TRUE | FALSE |
| CHEMBL1997706 | COc1ccccc1-c1csc(CCC(N)C(=O)O)n1 | alpha | 2.1632 | 292.36 | TRUE | FALSE |
| CHEMBL1997911 | Cl.NC(CC1=CS(=O)(=O)c2ccc(Cl)cc21)C(=O)O | alpha | 1.692 | 324.185 | TRUE | FALSE |
| CHEMBL1998164 | NC(Cc1ccc(O)cn1)C(=O)O | alpha | -0.2584 | 182.179 | TRUE | FALSE |
| CHEMBL1998500 | CCC(Nc1ncnc2nc[nH]c12)C(=O)O | alpha | 0.628 | 221.22 | TRUE | FALSE |
| CHEMBL199943 | CC(C)[C@@H](NS(=O)(=O)c1cccc(-c2ccccc2)c1)C(=O)O | alpha | 2.7411 | 333.409 | TRUE | TRUE |
| CHEMBL1999612 | O=C(O)C(NCc1ccccc1)C(NCc1ccccc1)C(=O)O | alpha | 1.4724 | 328.368 | TRUE | FALSE |
| CHEMBL2000077 | C=CSCCC(N)C(=O)O | alpha | 0.6651 | 161.226 | FALSE | FALSE |
| CHEMBL2000099 | CCCC[S+]([O-])CCC(N)C(=O)O | alpha | 0.3372 | 207.295 | FALSE | FALSE |
| CHEMBL2000562 | CC(NCc1c(O)ccc2ccc(=O)oc12)C(=O)O | alpha | 1.0613 | 263.249 | TRUE | FALSE |
| CHEMBL2000610 | Cc1ccc(S(=O)(=O)NC(C(=O)O)C(C)C)cc1 | alpha | 1.38252 | 271.338 | TRUE | FALSE |
| CHEMBL2000647 | O=C(O)[C@@H](NS(=O)(=O)c1ccc(F)cc1)c1ccccc1 | alpha | 1.9299 | 309.318 | TRUE | TRUE |
| CHEMBL200237 | N[C@H](Cn1ccc(=O)n(CCCC(=O)O)c1=O)C(=O)O | alpha | -1.7133 | 285.256 | TRUE | TRUE |
| CHEMBL2002949 | O=C(O)[C@@H]1NC[C@H]2C(Br)=NO[C@H]21 | alpha | -0.2338 | 235.037 | FALSE | TRUE |
| CHEMBL2002998 | O=CNC(Cc1c[nH]cn1)C(=O)O | alpha | -0.8486 | 183.167 | TRUE | FALSE |
| CHEMBL2003019 | C#CCNC(CS)C(=O)O | alpha | -0.4078 | 159.21 | FALSE | FALSE |
| CHEMBL2003547 | CSCCC(NCc1c(O)ccc2ccc(=O)oc12)C(=O)O | alpha | 1.7945 | 323.37 | TRUE | FALSE |
| CHEMBL2003661 | O=C(O)[C@H]1NC[C@H]2C(Br)=NO[C@@H]12 | alpha | -0.2338 | 235.037 | FALSE | TRUE |
| CHEMBL2004046 | Cc1csc(CCC(N)C(=O)O)n1 | alpha | 0.79602 | 200.263 | TRUE | FALSE |
| CHEMBL2004214 | NC(=O)SCCC(N)C(=O)O | alpha | -0.3997 | 178.213 | FALSE | FALSE |
| CHEMBL2004584 | C[N+](C)(C)c1ccc(C(N)C(=O)O)cc1.Cl.[Cl-] | alpha | -1.6065 | 281.183 | TRUE | FALSE |
| CHEMBL200466 | CC(C)[C@@H](NS(=O)(=O)c1ccc(-c2ccccc2)c(N)c1)C(=O)O | alpha | 2.3233 | 348.424 | TRUE | TRUE |
| CHEMBL2004821 | C/C(=C\CSCC(N)C(=O)O)CC/C=C(\C)CNc1ccccc1 | alpha | 3.9164 | 348.512 | TRUE | FALSE |
| CHEMBL2004913 | NC(CCSCC(F)(F)F)C(=O)O | alpha | 1.0839 | 217.212 | FALSE | FALSE |
| CHEMBL2005444 | Cl.NC(Cc1ncccn1)C(=O)O | alpha | -0.1472 | 203.629 | TRUE | FALSE |
| CHEMBL2006744 | Cc1ccc(S(=O)(=O)NC(CO)C(=O)O)cc1 | alpha | -0.28118 | 259.283 | TRUE | FALSE |
| CHEMBL2006912 | O=C(O)[C@H]1NC[C@@H]2ON=C(Br)[C@@H]21 | alpha | -0.2338 | 235.037 | FALSE | TRUE |
| CHEMBL200749 | CC(C)[C@@H](NS(=O)(=O)c1ccc(-c2cccc(O)c2)cc1)C(=O)O | alpha | 2.4467 | 349.408 | TRUE | TRUE |
| CHEMBL2007614 | Cl.NC(Cc1c[nH]c(I)n1)C(=O)O | alpha | 0.3905 | 317.514 | TRUE | FALSE |
| CHEMBL2007845 | C[C@H](Nc1nc(N)c(N=O)c(=O)n1C)C(=O)O | alpha | -0.3546 | 241.207 | TRUE | TRUE |
| CHEMBL2008452 | C[N+](C)(C)c1ccc(C(N)C(=O)O)cc1 | alpha | 0.9677 | 209.269 | TRUE | FALSE |
| CHEMBL2009137 | NC(Cc1ncccn1)C(=O)O | alpha | -0.569 | 167.168 | TRUE | FALSE |
| CHEMBL2009665 | CCCCCCCCCCCCP(=O)(O)OCC(N)C(=O)O | alpha | 3.5212 | 337.397 | FALSE | FALSE |
| CHEMBL2009675 | NC(CC1=CS(=O)(=O)c2ccc(Cl)cc21)C(=O)O | alpha | 1.2702 | 287.724 | TRUE | FALSE |
| CHEMBL2010136 | CC(C)(SCc1ccccc1)C(N)C(=O)O | alpha | 2.1103 | 239.34 | TRUE | FALSE |
| CHEMBL2010171 | NC(CCC(=O)c1ccccn1)C(=O)O | alpha | 0.4564 | 208.217 | TRUE | FALSE |
| CHEMBL2010433 | NC(Cc1c[nH]c(I)n1)C(=O)O | alpha | -0.0313 | 281.053 | TRUE | FALSE |
| CHEMBL2021354 | N[C@@H](CC(CC(=O)O)Nc1ccc(O)cc1)C(=O)O | alpha | 0.4494 | 268.269 | TRUE | TRUE |
| CHEMBL2024251 | CC(NC(CC(=O)c1ccc(F)cc1)C(=O)O)c1ccccc1 | alpha | 3.2025 | 315.344 | TRUE | FALSE |
| CHEMBL2024253 | C[C@H](NC(CC(=O)c1ccccc1)C(=O)O)c1ccccc1 | alpha | 3.0634 | 297.354 | TRUE | TRUE |
| CHEMBL2024254 | C[C@H](NC(CC(=O)c1ccc(F)cc1)C(=O)O)c1ccccc1 | alpha | 3.2025 | 315.344 | TRUE | TRUE |
| CHEMBL2024255 | C[C@H](NC(CC(=O)c1ccc(Cl)cc1)C(=O)O)c1ccccc1 | alpha | 3.7168 | 331.799 | TRUE | TRUE |
| CHEMBL2024256 | C[C@H](NC(CC(=O)c1ccccc1Cl)C(=O)O)c1ccccc1 | alpha | 3.7168 | 331.799 | TRUE | TRUE |
| CHEMBL2024258 | COc1ccccc1C(=O)CC(N[C@@H](C)c1ccccc1)C(=O)O | alpha | 3.072 | 327.38 | TRUE | TRUE |
| CHEMBL2024260 | C[C@H](NC(C(=O)O)C(C)(C)C(=O)c1ccccc1)c1ccccc1 | alpha | 3.6995 | 325.408 | TRUE | TRUE |
| CHEMBL2024261 | C[C@H](NC(CC(O)c1ccc(Cl)cc1)C(=O)O)c1ccccc1 | alpha | 3.5675 | 333.815 | TRUE | TRUE |
| CHEMBL2024265 | CC(C(=O)c1ccc(Cl)cc1)C(N[C@@H](C)c1ccccc1)C(=O)O | alpha | 3.9628 | 345.826 | TRUE | TRUE |
| CHEMBL2024334 | [2H]C([2H])(C(=O)c1ccccc1Cl)C(N[C@@H](C)c1ccccc1)C(=O)O | alpha | 3.7168 | 333.8112036 | TRUE | TRUE |
| CHEMBL202786 | N[C@H](Cn1ccc(=O)n(Cc2ccsc2C(=O)O)c1=O)C(=O)O | alpha | -0.7701 | 339.329 | TRUE | TRUE |
| CHEMBL2028905 | N[C@@H](CCCCNc1ccc([N+](=O)[O-])cc1[N+](=O)[O-])C(=O)O | alpha | 1.4971 | 312.282 | TRUE | TRUE |
| CHEMBL203616 | NC(CCSCc1cccc(Cl)c1)C(=O)O | alpha | 2.3752 | 259.758 | TRUE | FALSE |
| CHEMBL203743 | Cc1ccccc1CSCCC(N)C(=O)O | alpha | 2.03022 | 239.34 | TRUE | FALSE |
| CHEMBL203746 | NC(CCSCc1ccc(Br)cc1)C(=O)O | alpha | 2.4843 | 304.209 | TRUE | FALSE |
| CHEMBL203813 | NC(CCSCc1cccc(OC(F)(F)F)c1)C(=O)O | alpha | 2.6204 | 309.309 | TRUE | FALSE |
| CHEMBL203905 | NC(C(=O)O)c1ccc(F)c(Cl)c1 | alpha | 1.5635 | 203.6 | TRUE | FALSE |
| CHEMBL2040644 | NC(CC(=O)c1ccccc1)C(=O)O | alpha | 0.6713 | 193.202 | TRUE | FALSE |
| CHEMBL204096 | NC(CCSCc1ccc(-c2ccccc2)cc1)C(=O)O | alpha | 3.3888 | 301.411 | TRUE | FALSE |
| CHEMBL204179 | Cc1ccccc1[C@H](N)C(=O)O | alpha | 1.07942 | 165.192 | TRUE | TRUE |
| CHEMBL204478 | NC(CCSCc1cccc([N+](=O)[O-])c1)C(=O)O | alpha | 1.63 | 270.31 | TRUE | FALSE |
| CHEMBL204479 | N#Cc1ccccc1CSCCC(N)C(=O)O | alpha | 1.59348 | 250.323 | TRUE | FALSE |
| CHEMBL204611 | NC(CCSCc1cccc(F)c1)C(=O)O | alpha | 1.8609 | 243.303 | TRUE | FALSE |
| CHEMBL204646 | NC(CCSCc1ccccc1Br)C(=O)O | alpha | 2.4843 | 304.209 | TRUE | FALSE |
| CHEMBL204655 | NC(C(=O)O)c1cccc(Cl)c1 | alpha | 1.4244 | 185.61 | TRUE | FALSE |
| CHEMBL204698 | NC(C(=O)O)c1ccc(C(F)(F)F)cc1 | alpha | 1.7898 | 219.162 | TRUE | FALSE |
| CHEMBL204804 | NC(C(=O)O)c1cccc(F)c1F | alpha | 1.0492 | 187.145 | TRUE | FALSE |
| CHEMBL204818 | NC(CCSCc1cc(Cl)cc(Cl)c1)C(=O)O | alpha | 3.0286 | 294.203 | TRUE | FALSE |
| CHEMBL204870 | Cc1ccc(CSCCC(N)C(=O)O)cc1C | alpha | 2.33864 | 253.367 | TRUE | FALSE |
| CHEMBL204961 | COc1ccccc1CSCCC(N)C(=O)O | alpha | 1.7304 | 255.339 | TRUE | FALSE |
| CHEMBL204963 | COc1cccc(CSCCC(N)C(=O)O)c1 | alpha | 1.7304 | 255.339 | TRUE | FALSE |
| CHEMBL205126 | NC(CCSCc1cc(Cl)ccc1Cl)C(=O)O | alpha | 3.0286 | 294.203 | TRUE | FALSE |
| CHEMBL2062984 | O=C/C=C1/C=C(C(=O)O)N[C@H](C(=O)O)C1 | alpha | -0.4733 | 211.173 | FALSE | TRUE |
| CHEMBL2064046 | O=C(O)C1COC(C(=O)O)N1 | alpha | -1.5299 | 161.113 | FALSE | FALSE |
| CHEMBL2064048 | O=C(O)C1CCC(C(=O)O)N1 | alpha | -0.7238 | 159.141 | FALSE | FALSE |
| CHEMBL206556 | N[C@@H](CCCCNC(=O)CS)C(=O)O | alpha | -0.3854 | 220.294 | FALSE | TRUE |
| CHEMBL206725 | N[C@@H](CCCCN(O)C=O)C(=O)O | alpha | -0.5838 | 190.199 | FALSE | TRUE |
| CHEMBL2068493 | NC(=O)BCCNC(CC(=O)O)C(=O)O | alpha | -1.5626 | 216.002 | FALSE | FALSE |
| CHEMBL2074648 | N[C@@H](CC(=O)NCC(=O)O)C(=O)O | alpha | -2.0108 | 190.155 | FALSE | TRUE |
| CHEMBL2074942 | CC(=O)Nc1ccc(O)c(SCC(N)C(=O)O)c1 | alpha | 0.8546 | 270.31 | TRUE | FALSE |
| CHEMBL2074983 | N[C@@H](CSc1nc2ccccc2s1)C(=O)O | alpha | 1.8003 | 254.336 | TRUE | TRUE |
| CHEMBL2074985 | N[C@H](CSC(F)(F)C(F)Cl)C(=O)O | alpha | 1.2587 | 237.63 | FALSE | TRUE |
| CHEMBL2074986 | N[C@H](CS/C(Cl)=C/Cl)C(=O)O | alpha | 1.408 | 216.089 | FALSE | TRUE |
| CHEMBL2074987 | N[C@H](CSc1nc2ccccc2s1)C(=O)O | alpha | 1.8003 | 254.336 | TRUE | TRUE |
| CHEMBL20849 | CCC(NN=C(N)N)C(=O)O | alpha | -1.3724 | 160.177 | FALSE | FALSE |
| CHEMBL208607 | NC(CCSCCCCCC(=O)O)C(=O)O | alpha | 1.1666 | 249.332 | FALSE | FALSE |
| CHEMBL208759 | NC(CCSCCOCP(=O)(O)O)C(=O)O | alpha | -0.3266 | 273.247 | FALSE | FALSE |
| CHEMBL2087630 | O=C(O)[C@@H]1CSC(Cc2ccccc2)N1 | alpha | 1.3448 | 223.297 | TRUE | TRUE |
| CHEMBL208833 | NC(CCS(=O)(=O)CCCCC(=O)O)C(=O)O | alpha | -0.5419 | 267.303 | FALSE | FALSE |
| CHEMBL208893 | NC(CCSCC(=O)NCP(=O)(O)O)C(=O)O | alpha | -1.227 | 286.246 | FALSE | FALSE |
| CHEMBL209218 | N[C@@H](CSCCCCC(=O)O)C(=O)O | alpha | 0.3864 | 221.278 | FALSE | TRUE |
| CHEMBL209229 | NC(CC[S+]([O-])CCCCC(=O)O)C(=O)O | alpha | -0.208 | 251.304 | FALSE | FALSE |
| CHEMBL209241 | NC(CCSCC(N)C(=O)O)C(=O)O | alpha | -1.0665 | 222.266 | FALSE | FALSE |
| CHEMBL209316 | NC(CCSCCCCP(=O)(O)O)C(=O)O | alpha | 0.4795 | 271.275 | FALSE | FALSE |
| CHEMBL209393 | C=CCSCCC(N)C(=O)O | alpha | 0.7076 | 175.253 | FALSE | FALSE |
| CHEMBL209532 | NC(CCSc1ccccc1C(=O)O)C(=O)O | alpha | 1.2789 | 255.295 | TRUE | FALSE |
| CHEMBL210169 | NC(CCSc1cccc(C(=O)O)c1)C(=O)O | alpha | 1.2789 | 255.295 | TRUE | FALSE |
| CHEMBL2103812 | N[C@H](CCC(=O)N[C@@H](Cc1c[nH]c2ccccc12)C(=O)O)C(=O)O | alpha | 0.4719 | 333.344 | TRUE | TRUE |
| CHEMBL2103827 | N[C@H](C(=O)O)[C@H](O)c1ccc(O)c(O)c1 | alpha | -0.4569 | 213.189 | TRUE | TRUE |
| CHEMBL2104778 | Cc1ncc(CO)c(C2NC(C(=O)O)CCS2)c1O | alpha | 0.76622 | 284.337 | TRUE | FALSE |
| CHEMBL2105362 | O=C(O)C1CCSCN1 | alpha | 0.1236 | 147.199 | FALSE | FALSE |
| CHEMBL2105470 | C[C@@H]1C[S+]([O-])C(C)(C)[C@H](C(=O)O)N1 | alpha | -0.0414 | 205.279 | FALSE | TRUE |
| CHEMBL2105861 | CC(C)=CCSC[C@H](N)C(=O)O | alpha | 1.0977 | 189.28 | FALSE | TRUE |
| CHEMBL2106758 | N[C@@H](CCC(=O)NCCS(=O)(=O)O)C(=O)O | alpha | -1.8175 | 254.264 | FALSE | TRUE |
| CHEMBL2107075 | N[C@@H](Cc1cccc(N(CCCl)CCCl)c1)C(=O)O | alpha | 1.925 | 305.205 | TRUE | TRUE |
| CHEMBL2107426 | [2H][C@@](N)(CF)C(=O)O | alpha | -0.6322 | 108.0901018 | FALSE | TRUE |
| CHEMBL210750 | NC(CCSCc1ccc(CC(=O)O)cc1)C(=O)O | alpha | 1.3489 | 283.349 | TRUE | FALSE |
| CHEMBL210903 | CCCCCCCCCCCCCCC(N)C(=O)O | alpha | 4.4895 | 271.445 | FALSE | FALSE |
| CHEMBL2111117 | N[C@H](CF)C(=O)O | alpha | -0.6322 | 107.084 | FALSE | TRUE |
| CHEMBL2111523 | CC/C=C\C[C@H](C[C@H](N)C(=O)O)C(=O)O | alpha | 0.8455 | 215.249 | FALSE | TRUE |
| CHEMBL2111734 | CC1N[C@@H](C(=O)O)C[Se]1 | alpha | -0.4887 | 194.092 | FALSE | TRUE |
| CHEMBL2112459 | CC(C)CNC(=O)[C@H]1N[C@H]1C(=O)O | alpha | -0.8164 | 186.211 | FALSE | TRUE |
| CHEMBL2112529 | CC1N[C@H](C(=O)O)C[Se]1 | alpha | -0.4887 | 194.092 | FALSE | TRUE |
| CHEMBL2113110 | Cc1ccccc1-c1ccc(NC(=O)C[C@H](N)C(=O)O)cc1 | alpha | 2.40252 | 298.342 | TRUE | TRUE |
| CHEMBL2113121 | N[C@@H](CC(=O)Nc1ccc(Oc2ccc(F)c(F)c2)cc1)C(=O)O | alpha | 2.4976 | 336.294 | TRUE | TRUE |
| CHEMBL2113125 | Cc1cc(NC(=O)C[C@H](N)C(=O)O)ccc1-c1ccc(Cl)cc1 | alpha | 3.05592 | 332.787 | TRUE | TRUE |
| CHEMBL2113129 | N[C@@H](CC(=O)Nc1ccc(Cc2ccccc2)cc1)C(=O)O | alpha | 2.0179 | 298.342 | TRUE | TRUE |
| CHEMBL2114105 | N[C@H](C(=O)O)[C@@H]1[C@H](C(=O)O)[C@@H]1c1ccccc1 | alpha | 0.5127 | 235.239 | TRUE | TRUE |
| CHEMBL2114106 | N[C@H](C(=O)O)[C@@H]1[C@@H](C(=O)O)[C@H]1c1ccccc1 | alpha | 0.5127 | 235.239 | TRUE | TRUE |
| CHEMBL2114109 | N[C@H](C(=O)O)[C@H]1[C@@H](C(=O)O)[C@@H]1c1ccccc1 | alpha | 0.5127 | 235.239 | TRUE | TRUE |
| CHEMBL2114110 | N[C@@H](C(=O)O)[C@@H]1[C@H](C(=O)O)[C@H]1c1ccccc1 | alpha | 0.5127 | 235.239 | TRUE | TRUE |
| CHEMBL2114112 | N[C@H](C(=O)O)[C@H]1[C@@H](C(=O)O)[C@H]1c1ccccc1 | alpha | 0.5127 | 235.239 | TRUE | TRUE |
| CHEMBL2114115 | N[C@H](C(=O)O)[C@H]1[C@H](C(=O)O)[C@@H]1c1ccccc1 | alpha | 0.5127 | 235.239 | TRUE | TRUE |
| CHEMBL2114116 | N[C@H](C(=O)O)[C@H]1[C@H](C(=O)O)[C@H]1c1ccccc1 | alpha | 0.5127 | 235.239 | TRUE | TRUE |
| CHEMBL2114361 | O=C(O)[C@H]1CN(C(c2ccccc2)P(=O)(O)O)CCN1 | alpha | 0.2213 | 300.251 | TRUE | TRUE |
| CHEMBL2114471 | Cc1ccc([Se]C[C@@H](N)C(=O)O)cc1 | alpha | 0.15472 | 258.179 | TRUE | TRUE |
| CHEMBL2114472 | CCC[Se]C[C@@H](N)C(=O)O | alpha | 0.3491 | 210.135 | FALSE | TRUE |
| CHEMBL2114475 | Cc1ccc(C[Se]C[C@@H](N)C(=O)O)cc1 | alpha | 1.02942 | 272.206 | TRUE | TRUE |
| CHEMBL2115087 | N[C@@H](C(=O)O)[C@@H]1[C@H](C(=O)O)C1(F)F | alpha | -0.6358 | 195.121 | FALSE | TRUE |
| CHEMBL2115088 | N[C@@H](C(=O)O)[C@H]1[C@@H](C(=O)O)C1(F)F | alpha | -0.6358 | 195.121 | FALSE | TRUE |
| CHEMBL2115151 | N[C@@H](C(=O)O)[C@@H]1[C@@H](C(=O)O)[C@@H]1c1ccccc1 | alpha | 0.5127 | 235.239 | TRUE | TRUE |
| CHEMBL2115152 | N[C@@H](C(=O)O)[C@@H]1[C@H](C(=O)O)[C@@H]1c1ccccc1 | alpha | 0.5127 | 235.239 | TRUE | TRUE |
| CHEMBL2115153 | N[C@@H](C(=O)O)[C@@H]1[C@@H](C(=O)O)[C@H]1c1ccccc1 | alpha | 0.5127 | 235.239 | TRUE | TRUE |
| CHEMBL2115156 | N[C@@H](C(=O)O)[C@H]1[C@@H](C(=O)O)[C@@H]1c1ccccc1 | alpha | 0.5127 | 235.239 | TRUE | TRUE |
| CHEMBL2115157 | N[C@@H](C(=O)O)[C@H]1[C@@H](C(=O)O)[C@H]1c1ccccc1 | alpha | 0.5127 | 235.239 | TRUE | TRUE |
| CHEMBL2115159 | N[C@@H](C(=O)O)[C@H]1[C@H](C(=O)O)[C@@H]1c1ccccc1 | alpha | 0.5127 | 235.239 | TRUE | TRUE |
| CHEMBL2115160 | N[C@@H](C(=O)O)[C@H]1[C@H](C(=O)O)[C@H]1c1ccccc1 | alpha | 0.5127 | 235.239 | TRUE | TRUE |
| CHEMBL2115546 | O=C(O)[C@H]1Cc2c(CCP(=O)(O)O)cccc2CN1 | alpha | 0.5057 | 285.236 | TRUE | TRUE |
| CHEMBL211648 | NC(CCSCC[S+]([O-])CC(=O)O)C(=O)O | alpha | -0.6451 | 269.344 | FALSE | FALSE |
| CHEMBL211961 | NC(CCSCCSCC(=O)O)C(=O)O | alpha | 0.3394 | 253.345 | FALSE | FALSE |
| CHEMBL212056 | NC(CCSSCCC(N)C(=O)O)C(=O)O | alpha | -0.0282 | 268.36 | FALSE | FALSE |
| CHEMBL212166 | CSCSCCC(N)C(=O)O | alpha | 0.8421 | 195.309 | FALSE | FALSE |
| CHEMBL212367 | NC(CC[S+]([O-])CC(N)C(=O)O)C(=O)O | alpha | -2.051 | 238.265 | FALSE | FALSE |
| CHEMBL212727 | NC(CCSCCCC(=O)O)C(=O)O | alpha | 0.3864 | 221.278 | FALSE | FALSE |
| CHEMBL213575 | NC(CSCc1oc(-c2ccccc2)cc1C(=O)O)C(=O)O | alpha | 2.2899 | 321.354 | TRUE | FALSE |
| CHEMBL2147117 | Nc1nc(NC(CO)C(=O)O)c(N=O)c(=O)[nH]1 | alpha | -1.3926 | 243.179 | TRUE | FALSE |
| CHEMBL2147118 | CCC(C)C(Nc1nc(N)[nH]c(=O)c1N=O)C(=O)O | alpha | 0.6612 | 269.261 | TRUE | FALSE |
| CHEMBL214720 | CC(Nc1ccnc2cc(Cl)ccc12)C(=O)O | alpha | 2.7732 | 250.685 | TRUE | FALSE |
| CHEMBL2147945 | CS(=O)(=O)N[C@@H](Cc1ccc(OCc2ccccc2)cc1)C(=O)O | alpha | 1.8105 | 349.408 | TRUE | TRUE |
| CHEMBL2148203 | O=C(O)C1C[C@@H](O)CCN1 | alpha | -0.8161 | 145.158 | FALSE | TRUE |
| CHEMBL2164721 | N[C@@H](CCSCSCC[C@H](N)C(=O)O)C(=O)O | alpha | 0.0143 | 282.387 | FALSE | TRUE |
| CHEMBL2164722 | N[C@@H](CCSCCSCC[C@H](N)C(=O)O)C(=O)O | alpha | 0.0568 | 296.414 | FALSE | TRUE |
| CHEMBL2164723 | NC(CCCCCSCC[C@H](N)C(=O)O)C(=O)O | alpha | 0.4939 | 278.374 | FALSE | TRUE |
| CHEMBL2164724 | CC(CCSCC[C@H](N)C(=O)O)CCC(N)C(=O)O | alpha | 0.7399 | 292.401 | FALSE | TRUE |
| CHEMBL2164725 | CC(C)(CCSCC[C@H](N)C(=O)O)CCC(N)C(=O)O | alpha | 1.13 | 306.428 | FALSE | TRUE |
| CHEMBL2164726 | N[C@@H](CCSCC[S+]([O-])CC[C@H](N)C(=O)O)C(=O)O | alpha | -0.9277 | 312.413 | FALSE | TRUE |
| CHEMBL2164727 | N[C@@H](CC[S+]([O-])CC[S+]([O-])CC[C@H](N)C(=O)O)C(=O)O | alpha | -1.9122 | 328.412 | FALSE | TRUE |
| CHEMBL2164728 | C[S+](CCSCC[C@H](N)C(=O)O)CC[C@H](N)C(=O)O | alpha | -0.4283 | 311.449 | FALSE | TRUE |
| CHEMBL21822 | N=C(N)NNC(C(=O)O)c1ccccc1 | alpha | -0.20003 | 208.221 | TRUE | FALSE |
| CHEMBL218352 | CCCCCCCCCCC1N[C@H](C(=O)O)CS1 | alpha | 3.6329 | 273.442 | FALSE | TRUE |
| CHEMBL22019 | N/N=C(\NN)N[C@@H](Cc1ccccc1)C(=O)O | alpha | -1.0351 | 237.263 | TRUE | TRUE |
| CHEMBL220289 | CCCCCCCCCCCCCCC(NCCCC)C(=O)O | alpha | 5.9205 | 327.553 | FALSE | FALSE |
| CHEMBL2208196 | N[C@H](CCCCNC(=O)C(=O)c1c[nH]c2cc(O)ccc12)C(=O)O | alpha | 0.7546 | 333.344 | TRUE | TRUE |
| CHEMBL22168 | NC(C(=O)O)c1ccc(CP(=O)(O)O)cc1 | alpha | 0.4487 | 245.171 | TRUE | FALSE |
| CHEMBL222029 | CCCCCCCCCCCC1N[C@H](C(=O)O)CS1 | alpha | 4.023 | 287.469 | FALSE | TRUE |
| CHEMBL222521 | Cc1cn(C[C@H](N)C(=O)O)c(=O)n(Cc2ccccc2C(=O)O)c1=O | alpha | -0.52318 | 347.327 | TRUE | TRUE |
| CHEMBL2235645 | N[C@H](C[C@H](C(=O)O)c1cccc(-c2ccco2)c1)C(=O)O | alpha | 1.9168 | 289.287 | TRUE | TRUE |
| CHEMBL223662 | CC1(C)ON=C(C(=O)O)[C@H]1C[C@@H](N)C(=O)O | alpha | -0.346 | 230.22 | FALSE | TRUE |
| CHEMBL223663 | CC1(C)ON=C(C(=O)O)[C@H]1C[C@H](N)C(=O)O | alpha | -0.346 | 230.22 | FALSE | TRUE |
| CHEMBL224678 | O=C(O)C1CN(C/C=C/P(=O)(O)O)CCN1 | alpha | -0.9638 | 250.191 | FALSE | FALSE |
| CHEMBL2253493 | N[C@@H](CCCCNC(=O)COc1ccccc1)C(=O)O | alpha | 0.7638 | 280.324 | TRUE | TRUE |
| CHEMBL2253494 | N[C@@H](CCCCNC(=O)COc1ccc(Cl)cc1)C(=O)O | alpha | 1.4172 | 314.769 | TRUE | TRUE |
| CHEMBL2253495 | N[C@@H](CCCCNC(=O)COc1ccc(Cl)cc1Cl)C(=O)O | alpha | 2.0706 | 349.214 | TRUE | TRUE |
| CHEMBL2253496 | Cc1cc(Cl)ccc1OCC(=O)NCCCC[C@H](N)C(=O)O | alpha | 1.72562 | 328.796 | TRUE | TRUE |
| CHEMBL2253497 | N[C@@H](CCCCNC(=O)COc1ccc([N+](=O)[O-])cc1)C(=O)O | alpha | 0.672 | 325.321 | TRUE | TRUE |
| CHEMBL2253498 | Nc1ccc(OCC(=O)NCCCC[C@H](N)C(=O)O)cc1 | alpha | 0.346 | 295.339 | TRUE | TRUE |
| CHEMBL2253500 | N[C@H](CCCCNC(=O)COc1ccc(Cl)cc1Cl)C(=O)O | alpha | 2.0706 | 349.214 | TRUE | TRUE |
| CHEMBL2261742 | NCCC[C@H](NN)C(=O)O | alpha | -1.3582 | 147.178 | FALSE | TRUE |
| CHEMBL2261744 | N[C@@H](CCCNC(=O)CI)C(=O)O | alpha | -0.2703 | 300.096 | FALSE | TRUE |
| CHEMBL2261745 | N[C@@H](CCCCNC(=O)CI)C(=O)O | alpha | 0.1198 | 314.123 | FALSE | TRUE |
| CHEMBL2261746 | N[C@@H](CCCNC(=O)CCl)C(=O)O | alpha | -0.4665 | 208.645 | FALSE | TRUE |
| CHEMBL2273024 | NC(Cc1ccco1)C(=O)O | alpha | 0.234 | 155.153 | TRUE | FALSE |
| CHEMBL22740 | N[C@@H](CC(=O)c1cccc([N+](=O)[O-])c1)C(=O)O | alpha | 0.5795 | 238.199 | TRUE | TRUE |
| CHEMBL2282164 | O=C(O)C(NNc1ccc(F)cc1)C(=O)O | alpha | 0.28 | 228.179 | TRUE | FALSE |
| CHEMBL23074 | Cc1cc(C)c(CCP(=O)(O)O)c(C[C@H](N)C(=O)O)c1 | alpha | 0.97804 | 301.279 | TRUE | TRUE |
| CHEMBL23081 | O=C(O)C1Cc2ccccc2N1 | alpha | 1.1078 | 163.176 | TRUE | FALSE |
| CHEMBL23085 | Cc1ccc(CCP(=O)(O)O)c(C[C@H](N)C(=O)O)c1 | alpha | 0.66962 | 287.252 | TRUE | TRUE |
| CHEMBL230852 | N[C@@H](C(=O)O)[C@@H]1C[C@H]1S(=O)(=O)O | alpha | -1.3254 | 195.196 | FALSE | TRUE |
| CHEMBL230949 | N[C@@H](C(=O)O)[C@@H]1C[C@@H]1S(=O)(=O)O | alpha | -1.3254 | 195.196 | FALSE | TRUE |
| CHEMBL230950 | N[C@@H](C(=O)O)[C@H]1C[C@H]1S(=O)(=O)O | alpha | -1.3254 | 195.196 | FALSE | TRUE |
| CHEMBL2311075 | N[C@@H](CS/C(Cl)=C/Cl)C(=O)O | alpha | 1.408 | 216.089 | FALSE | TRUE |
| CHEMBL231157 | N[C@H](C(=O)O)[C@H]1C[C@@H]1S(=O)(=O)O | alpha | -1.3254 | 195.196 | FALSE | TRUE |
| CHEMBL2312396 | C#CCNC(=O)CC[C@H](C[C@H](N)C(=O)O)C(=O)O | alpha | -0.9812 | 256.258 | FALSE | TRUE |
| CHEMBL2312397 | N[C@@H](C[C@@H](CCC(=O)Nc1ccccc1)C(=O)O)C(=O)O | alpha | 0.9081 | 294.307 | TRUE | TRUE |
| CHEMBL2312398 | CN(C)C(=O)CC[C@H](C[C@H](N)C(=O)O)C(=O)O | alpha | -0.6424 | 246.263 | FALSE | TRUE |
| CHEMBL2312399 | CCN(CC)C(=O)CC[C@H](C[C@H](N)C(=O)O)C(=O)O | alpha | 0.1378 | 274.317 | FALSE | TRUE |
| CHEMBL2312400 | N[C@@H](C[C@@H](CCC(=O)N1CCCCC1)C(=O)O)C(=O)O | alpha | 0.2819 | 286.328 | FALSE | TRUE |
| CHEMBL2312401 | CC(C)(C)ONC(=O)CC[C@H](C[C@H](N)C(=O)O)C(=O)O | alpha | 0.1157 | 290.316 | FALSE | TRUE |
| CHEMBL2312402 | N[C@@H](C[C@@H](CCC(=O)NOCc1ccccc1)C(=O)O)C(=O)O | alpha | 0.5174 | 324.333 | TRUE | TRUE |
| CHEMBL2312680 | N[C@@H](C[C@@H](CCC(=O)NNc1ccccc1)C(=O)O)C(=O)O | alpha | 0.4127 | 309.322 | TRUE | TRUE |
| CHEMBL2312682 | N#CCC[C@H](C[C@H](N)C(=O)O)C(=O)O | alpha | -0.20702 | 200.194 | FALSE | TRUE |
| CHEMBL2312683 | N[C@@H](C[C@@H](CCC(=O)NO)C(=O)O)C(=O)O | alpha | -1.2252 | 234.208 | FALSE | TRUE |
| CHEMBL2312684 | NCC[C@H](C[C@H](N)C(=O)O)C(=O)O | alpha | -1.162 | 190.199 | FALSE | TRUE |
| CHEMBL2312685 | NCCC[C@H](C[C@H](N)C(=O)O)C(=O)O | alpha | -0.7719 | 204.226 | FALSE | TRUE |
| CHEMBL2312686 | O=C(O)[C@@H]1CCCN[C@H](C(=O)O)C1 | alpha | -0.0861 | 187.195 | FALSE | TRUE |
| CHEMBL2314664 | N[C@@H](Cc1ccc(O[C@H]2C[C@@H](F)C2)cc1)C(=O)O | alpha | 1.5203 | 253.273 | TRUE | TRUE |
| CHEMBL2314665 | N[C@@H](Cc1ccc(O[C@H]2C[C@@H]([18F])C2)cc1)C(=O)O | alpha | 1.5203 | 252.275938 | TRUE | TRUE |
| CHEMBL2316774 | COc1ccc([C@]2(O)CN[C@@H](C(=O)O)C2)cc1 | alpha | 0.3293 | 237.255 | TRUE | TRUE |
| CHEMBL2316776 | COc1ccc([C@@]2(O)CN[C@H](C(=O)O)C2)cc1 | alpha | 0.3293 | 237.255 | TRUE | TRUE |
| CHEMBL23182 | Cc1ccc(CCP(=O)(O)O)c(CC(N)C(=O)O)c1 | alpha | 0.66962 | 287.252 | TRUE | FALSE |
| CHEMBL23183 | Cc1cccc(CCP(=O)(O)O)c1CC(N)C(=O)O | alpha | 0.66962 | 287.252 | TRUE | FALSE |
| CHEMBL23190 | NC(Cc1cc(Cl)ccc1CCP(=O)(O)O)C(=O)O | alpha | 1.0146 | 307.67 | TRUE | FALSE |
| CHEMBL23192 | CC(C)(C)c1ccc(CCP(=O)(O)O)c(CC(N)C(=O)O)c1 | alpha | 1.6587 | 329.333 | TRUE | FALSE |
| CHEMBL231992 | CC(C)(C)SC[C@H](N)C(=O)O | alpha | 0.93 | 177.269 | FALSE | TRUE |
| CHEMBL2326088 | N[C@H](CCCCB(O)O)C(=O)O | alpha | -0.9586 | 174.993 | FALSE | TRUE |
| CHEMBL2333947 | C=CNC(=N)NCCC[C@H](N)C(=O)O | alpha | -0.56403 | 200.242 | FALSE | TRUE |
| CHEMBL233401 | O=C(O)C[C@@H](NS(=O)(=O)c1ccccc1)C(=O)O | alpha | -0.1072 | 273.266 | TRUE | TRUE |
| CHEMBL2336912 | C[C@H](NC[C@@H](N)Cc1ccccc1)C(=O)O | alpha | 0.6191 | 222.288 | TRUE | TRUE |
| CHEMBL234138 | NC(C(=O)O)[C@@H]1[C@H](c2ccccc2)[C@H]1P(=O)(O)O | alpha | 0.3582 | 271.209 | TRUE | TRUE |
| CHEMBL2347886 | O=C(O)C[C@H]1CNN[C@@H]1C(=O)O | alpha | -1.3617 | 174.156 | FALSE | TRUE |
| CHEMBL2348221 | CC[C@]1(C(=O)O)C[C@H]1[C@H](N)C(=O)O.Cl | alpha | 0.321 | 223.656 | FALSE | TRUE |
| CHEMBL2348222 | CCC[C@]1(C(=O)O)C[C@H]1[C@H](N)C(=O)O.Cl | alpha | 0.7111 | 237.683 | FALSE | TRUE |
| CHEMBL2348224 | C[C@]1(C(=O)O)C[C@H]1[C@H](N)C(=O)O.Cl | alpha | -0.0691 | 209.629 | FALSE | TRUE |
| CHEMBL2348500 | NC(CCCCB(O)O)C(=O)O | alpha | -0.9586 | 174.993 | FALSE | FALSE |
| CHEMBL235058 | Cn1cnc(C[C@H](NCCN)C(=O)O)c1 | alpha | -1.0359 | 212.253 | TRUE | TRUE |
| CHEMBL235277 | CCn1cnc(C[C@H](NCCN)C(=O)O)c1 | alpha | -0.553 | 226.28 | TRUE | TRUE |
| CHEMBL2355249 | N[C@H](C(=O)O)[C@@H](O)c1ccc(O)c(O)c1 | alpha | -0.4569 | 213.189 | TRUE | TRUE |
| CHEMBL235697 | NCCN[C@@H](Cc1c[nH]cn1)C(=O)O | alpha | -1.0463 | 198.226 | TRUE | TRUE |
| CHEMBL236362 | CC(C)Cn1cnc(C[C@H](NCCN)C(=O)O)c1 | alpha | 0.0831 | 254.334 | TRUE | TRUE |
| CHEMBL236363 | NCCN[C@@H](Cc1cn(Cc2ccccc2)cn1)C(=O)O | alpha | 0.4754 | 288.351 | TRUE | TRUE |
| CHEMBL236547 | CCCn1cnc(C[C@H](NCCN)C(=O)O)c1 | alpha | -0.1629 | 240.307 | TRUE | TRUE |
| CHEMBL236549 | CCCCn1cnc(C[C@H](NCCN)C(=O)O)c1 | alpha | 0.2272 | 254.334 | TRUE | TRUE |
| CHEMBL2365741 | CCC[C@]1(C(=O)O)C[C@H]1[C@H](N)C(=O)O | alpha | 0.2893 | 201.222 | FALSE | TRUE |
| CHEMBL2365742 | C[C@]1(C(=O)O)C[C@H]1[C@H](N)C(=O)O | alpha | -0.4909 | 173.168 | FALSE | TRUE |
| CHEMBL2365799 | CC[C@]1(C(=O)O)C[C@H]1[C@H](N)C(=O)O | alpha | -0.1008 | 187.195 | FALSE | TRUE |
| CHEMBL2367805 | N[C@@H](C(=O)O)[C@H]1C[C@H]1C(=O)O | alpha | -0.881 | 159.141 | FALSE | TRUE |
| CHEMBL2371228 | CCC[C@H](NCC(=O)N1C2CCCCC2C[C@H]1C(=O)O)C(=O)O | alpha | 1.0736 | 326.393 | FALSE | TRUE |
| CHEMBL2371229 | O=C(O)[C@H](CCc1ccccc1)NCC(=O)N1CCC[C@H]1C(=O)O | alpha | 0.7376 | 334.372 | TRUE | TRUE |
| CHEMBL23728 | Cc1cccc(CC(N)C(=O)O)c1CCP(=O)(O)O | alpha | 0.66962 | 287.252 | TRUE | FALSE |
| CHEMBL2373300 | C[C@]1([C@H](N)C(=O)O)CC[C@H]1C(=O)O | alpha | -0.1008 | 187.195 | FALSE | TRUE |
| CHEMBL23770 | C[S+](CCc1ccccc1)CCC(N)C(=O)O | alpha | 1.2792 | 254.375 | TRUE | FALSE |
| CHEMBL23774 | COc1ccccc1CC[S+](C)CCC(N)C(=O)O | alpha | 1.2878 | 284.401 | TRUE | FALSE |
| CHEMBL238437 | O=C(O)C1CSC(c2ccc([N+](=O)[O-])cc2)N1 | alpha | 1.383 | 254.267 | TRUE | FALSE |
| CHEMBL23848 | CCc1ccc(CCP(=O)(O)O)c(CC(N)C(=O)O)c1 | alpha | 0.9236 | 301.279 | TRUE | FALSE |
| CHEMBL238495 | Cc1ccc(C2NC(C(=O)O)CS2)cc1 | alpha | 1.78322 | 223.297 | TRUE | FALSE |
| CHEMBL238524 | NC(Cc1ccc(O)c(CCC[18F])c1)C(=O)O | alpha | 1.2487 | 240.264938 | TRUE | FALSE |
| CHEMBL238550 | COc1ccc(CC(N)C(=O)O)cc1CC[18F] | alpha | 1.1616 | 240.264938 | TRUE | FALSE |
| CHEMBL238653 | O=C(O)C1CSC(c2ccc(Cl)cc2)N1 | alpha | 2.1282 | 243.715 | TRUE | FALSE |
| CHEMBL239500 | O=C(O)C1CSC(c2ccc(O)cc2)N1 | alpha | 1.1804 | 225.269 | TRUE | FALSE |
| CHEMBL239501 | COc1ccc(C2NC(C(=O)O)CS2)cc1OC | alpha | 1.492 | 269.322 | TRUE | FALSE |
| CHEMBL239567 | NC(Cc1ccc(O)c(CC[18F])c1)C(=O)O | alpha | 0.8586 | 226.237938 | TRUE | FALSE |
| CHEMBL23983 | NC(CC(=O)c1cccc([N+](=O)[O-])c1)C(=O)O | alpha | 0.5795 | 238.199 | TRUE | FALSE |
| CHEMBL2398468 | CN(CCCC(=O)O)c1noc(CC[C@H](N)C(=O)O)n1 | alpha | -0.2849 | 286.288 | TRUE | TRUE |
| CHEMBL2398469 | CN(CCCC(=O)O)/C(=N\O)NC(=O)CC[C@H](N)C(=O)O | alpha | -1.1635 | 304.303 | FALSE | TRUE |
| CHEMBL2403309 | O=C(O)CCCCS[C@@H]1CN[C@H](C(=O)O)C1 | alpha | 0.7896 | 247.316 | FALSE | TRUE |
| CHEMBL2403310 | O=C(O)CCCCS[C@H]1CN[C@H](C(=O)O)C1 | alpha | 0.7896 | 247.316 | FALSE | TRUE |
| CHEMBL240354 | O=C(O)C1CSC(c2ccc(Cl)cc2Cl)N1 | alpha | 2.7816 | 278.16 | TRUE | FALSE |
| CHEMBL241972 | N[C@@H](CC[PH](=O)O)C(=O)O | alpha | -0.7446 | 167.101 | FALSE | TRUE |
| CHEMBL242343 | CCCCc1c(C(N)C(=O)O)cnn1O | alpha | 0.5474 | 213.237 | TRUE | FALSE |
| CHEMBL242344 | NC(C(=O)O)c1cnn(O)c1CC1CCCCC1 | alpha | 1.3276 | 253.302 | TRUE | FALSE |
| CHEMBL242379 | O=C(O)[C@@H]1C[C@@]2(CN1)CC(O)=NO2 | alpha | -0.5365 | 186.167 | FALSE | TRUE |
| CHEMBL242380 | O=C(O)[C@H]1C[C@]2(CN1)CC(O)=NO2 | alpha | -0.5365 | 186.167 | FALSE | TRUE |
| CHEMBL242381 | O=C(O)C1=NO[C@]2(CN[C@H](C(=O)O)C2)C1 | alpha | -0.9674 | 214.177 | FALSE | TRUE |
| CHEMBL242583 | O=C(O)C1=NO[C@]2(CN[C@@H](C(=O)O)C2)C1 | alpha | -0.9674 | 214.177 | FALSE | TRUE |
| CHEMBL2426753 | N[C@H](C[C@H]1CC(C(=O)O)=NN1c1ccccc1)C(=O)O | alpha | 0.5079 | 277.28 | TRUE | TRUE |
| CHEMBL2426754 | N[C@H](C[C@@H]1CC(C(=O)O)=NN1c1ccccc1)C(=O)O | alpha | 0.5079 | 277.28 | TRUE | TRUE |
| CHEMBL2426755 | Cl.N[C@H](C[C@H]1CC(C(=O)O)=NN1)C(=O)O | alpha | -0.9873 | 237.643 | FALSE | TRUE |
| CHEMBL2426756 | Cl.N[C@H](C[C@@H]1CC(C(=O)O)=NN1)C(=O)O | alpha | -0.9873 | 237.643 | FALSE | TRUE |
| CHEMBL2426757 | Cl.N[C@H](C[C@H]1CC(C(=O)O)=NN1Cc1ccccc1)C(=O)O | alpha | 0.9253 | 327.768 | TRUE | TRUE |
| CHEMBL2426758 | Cl.N[C@H](C[C@@H]1CC(C(=O)O)=NN1Cc1ccccc1)C(=O)O | alpha | 0.9253 | 327.768 | TRUE | TRUE |
| CHEMBL2429972 | CC(C)(C)[Si](F)(c1cn(CC(N)C(=O)O)nn1)C(C)(C)C | alpha | 1.4122 | 316.453 | TRUE | FALSE |
| CHEMBL2429973 | CC(C)(C)[Si](F)(Cc1cn(CC(N)C(=O)O)nn1)C(C)(C)C | alpha | 2.2869 | 330.48 | TRUE | FALSE |
| CHEMBL2429974 | CC(C)(C)[Si](F)(CCn1cc(CC(N)C(=O)O)nn1)C(C)(C)C | alpha | 2.7477 | 344.507 | TRUE | FALSE |
| CHEMBL2429983 | CC(C)(C)[Si]([18F])(CCn1cc(CC(N)C(=O)O)nn1)C(C)(C)C | alpha | 2.7477 | 343.509938 | TRUE | FALSE |
| CHEMBL243925 | N[C@@H](CCP(O)(O)=S)C(=O)O | alpha | -0.9175 | 199.168 | FALSE | TRUE |
| CHEMBL2449332 | CC(C)[C@H](N[C@H]1c2cc(C#N)ccc2OC(C)(C)[C@@H]1O)C(=O)O | alpha | 1.83008 | 318.373 | TRUE | TRUE |
| CHEMBL2449333 | CC(C)[C@H](N[C@@H]1c2cc(C#N)ccc2OC(C)(C)[C@H]1O)C(=O)O | alpha | 1.83008 | 318.373 | TRUE | TRUE |
| CHEMBL24845 | CC(Nc1ccccc1Oc1ccccc1)C(=O)O | alpha | 3.3639 | 257.289 | TRUE | FALSE |
| CHEMBL250947 | N[C@@H](CSNC(=O)c1ccccc1)C(=O)O | alpha | 0.4765 | 240.284 | TRUE | TRUE |
| CHEMBL25293 | CC(Nc1ccccc1C(=O)c1ccccc1)C(=O)O | alpha | 2.8026 | 269.3 | TRUE | FALSE |
| CHEMBL25451 | CC(Nc1ccc(Cl)cc1C(=O)c1ccccc1)C(=O)O | alpha | 3.456 | 303.745 | TRUE | FALSE |
| CHEMBL25452 | COc1ccc(NC(C)C(=O)O)c(C(=O)c2ccccc2)c1 | alpha | 2.8112 | 299.326 | TRUE | FALSE |
| CHEMBL255480 | O=C(O)[C@@H]1NCC=C[C@H]1O | alpha | -1.0401 | 143.142 | FALSE | TRUE |
| CHEMBL257807 | O=C(O)[C@@H]1NCC=C[C@@H]1O | alpha | -1.0401 | 143.142 | FALSE | TRUE |
| CHEMBL259256 | CCCCC(SC[C@H](N)C(=O)O)(c1ccccc1)c1ccccc1 | alpha | 4.2655 | 343.492 | TRUE | TRUE |
| CHEMBL259257 | CC(C)C(SC[C@H](N)C(=O)O)(c1ccccc1)c1ccccc1 | alpha | 3.7313 | 329.465 | TRUE | TRUE |
| CHEMBL260122 | N[C@H](C(=O)O)[C@H]1CC(O)=NO1 | alpha | -0.9413 | 160.129 | FALSE | TRUE |
| CHEMBL260327 | N[C@@H](C(=O)O)[C@H]1CC(O)=NO1 | alpha | -0.9413 | 160.129 | FALSE | TRUE |
| CHEMBL260328 | N[C@H](C(=O)O)[C@@H]1CC(O)=NO1 | alpha | -0.9413 | 160.129 | FALSE | TRUE |
| CHEMBL260501 | CCC(Nc1ccc([N+](=O)[O-])cc1[N+](=O)[O-])C(=O)O | alpha | 1.7781 | 269.213 | TRUE | FALSE |
| CHEMBL260628 | CN(CCC[C@H](N)C(=O)O)/C(=N/O)NO | alpha | -1.1657 | 220.229 | FALSE | TRUE |
| CHEMBL262416 | Cc1onc(O)c1CC[C@@H](N)C(=O)O | alpha | 0.03312 | 200.194 | TRUE | TRUE |
| CHEMBL263926 | CCCCCc1cccc(C2N[C@H](C(=O)O)CS2)c1 | alpha | 3.2075 | 279.405 | TRUE | TRUE |
| CHEMBL264383 | C[C@@H](O)[C@H]1C(=O)N2C(C(=O)O)=C(SC[C@@H](N)C(=O)O)S[C@H]12 | alpha | -0.7027 | 334.375 | FALSE | TRUE |
| CHEMBL26475 | O=C(O)[C@H]1C[C@@H](/C=C/CP(=O)(O)O)CCN1 | alpha | 0.1731 | 249.203 | FALSE | TRUE |
| CHEMBL264976 | O=C(O)[C@@H]1C[C@H](C(=O)O)c2c(Cl)cc(Cl)cc2N1 | alpha | 2.4304 | 290.102 | TRUE | TRUE |
| CHEMBL265996 | O=C(O)C[C@H]1[C@@H](C(=O)O)NC[C@@H]1c1ccccc1O | alpha | 0.6231 | 265.265 | TRUE | TRUE |
| CHEMBL267077 | C=C(C[C@@H](N)C(=O)O)C[C@H](N)C(=O)O | alpha | -0.8534 | 202.21 | FALSE | TRUE |
| CHEMBL267209 | NC(CCC(=O)Nc1ccc(O)cc1)C(=O)O | alpha | 0.5228 | 238.243 | TRUE | FALSE |
| CHEMBL268238 | CC(CC(N)C(=O)O)CC(N)C(=O)O | alpha | -0.7735 | 204.226 | FALSE | FALSE |
| CHEMBL268472 | CCCCCCc1c(O)noc1C[C@@H](N)C(=O)O | alpha | 1.4574 | 256.302 | TRUE | TRUE |
| CHEMBL268483 | NC(CCCSc1ccccc1O)C(=O)O | alpha | 1.6764 | 241.312 | TRUE | FALSE |
| CHEMBL268762 | Cc1c(O)noc1CC(N)C(=O)O | alpha | -0.35698 | 186.167 | TRUE | FALSE |
| CHEMBL269180 | C/C(=N\CCC[C@H](N)C(=O)O)NO | alpha | -0.4243 | 189.215 | FALSE | TRUE |
| CHEMBL269358 | NC(CCC(=O)NC1(C(=O)O)CCCC1)C(=O)O | alpha | -0.3079 | 258.274 | FALSE | FALSE |
| CHEMBL269574 | NC(CCC(=O)NCCCC(=O)O)C(=O)O | alpha | -0.8405 | 232.236 | FALSE | FALSE |
| CHEMBL272814 | CC(=O)O.CN/N=C(\N)NCCC[C@H](N)C(=O)O | alpha | -1.6919 | 263.298 | FALSE | TRUE |
| CHEMBL272851 | CC(=O)O.N/C(=N/CCC[C@H](N)C(=O)O)N(N)CC(F)(F)F | alpha | -0.6779 | 331.295 | FALSE | TRUE |
| CHEMBL273216 | O=C(O)[C@H](Cc1ccc(-c2ccccc2)cc1)NCP(=O)(O)O | alpha | 2.0741 | 335.296 | TRUE | TRUE |
| CHEMBL273291 | CCCCCCc1c(O)noc1C[C@H](N)C(=O)O | alpha | 1.4574 | 256.302 | TRUE | TRUE |
| CHEMBL273541 | O=C(O)[C@@H]1CCCN[C@H]1C(=O)O | alpha | -0.4762 | 173.168 | FALSE | TRUE |
| CHEMBL274216 | NC(CCCCCSc1ccccc1O)C(=O)O | alpha | 2.4566 | 269.366 | TRUE | FALSE |
| CHEMBL274440 | NC(CCCCCP(=O)(O)O)C(=O)O | alpha | 0.1364 | 225.181 | FALSE | FALSE |
| CHEMBL27567 | CC(C)C(NCc1cccc(CP(=O)(O)O)c1)C(=O)O | alpha | 1.5631 | 301.279 | TRUE | FALSE |
| CHEMBL277475 | NC(C(=O)O)c1ccc(P(=O)(O)O)cc1 | alpha | -0.426 | 231.144 | TRUE | FALSE |
| CHEMBL277671 | Cc1ccc(CC(N)C(=O)O)c(CCP(=O)(O)O)c1 | alpha | 0.66962 | 287.252 | TRUE | FALSE |
| CHEMBL27832 | CC(Nc1ccccc1C(=O)c1ccc(Cl)cc1Cl)C(=O)O | alpha | 4.1094 | 338.19 | TRUE | FALSE |
| CHEMBL278396 | COc1ccc(CCP(=O)(O)O)c(CC(N)C(=O)O)c1 | alpha | 0.3698 | 303.251 | TRUE | FALSE |
| CHEMBL27859 | NC(C(=O)O)C1CCC(CP(=O)(O)O)CC1 | alpha | 0.3824 | 251.219 | FALSE | FALSE |
| CHEMBL27873 | CC(Nc1ccccc1C(=O)c1ccc(Cl)cc1)C(=O)O | alpha | 3.456 | 303.745 | TRUE | FALSE |
| CHEMBL278925 | O=C(O)[C@H]1NC[C@H]1C(=O)O | alpha | -1.2564 | 145.114 | FALSE | TRUE |
| CHEMBL279028 | NC(Cc1cccc(-c2ccccc2)c1CCP(=O)(O)O)C(=O)O | alpha | 2.0282 | 349.323 | TRUE | FALSE |
| CHEMBL27928 | CC(Nc1ccccc1C(=O)c1ccc(Br)cc1)C(=O)O | alpha | 3.5651 | 348.196 | TRUE | FALSE |
| CHEMBL279561 | CC(C)[C@H]1CN[C@H](C(=O)O)[C@H]1CC(=O)O | alpha | 0.4059 | 215.249 | FALSE | TRUE |
| CHEMBL279731 | O=C(O)[C@H]1C[C@@H](CP(=O)(O)O)CCN1 | alpha | -0.3831 | 223.165 | FALSE | TRUE |
| CHEMBL280197 | Cc1ccc(C(=O)c2ccccc2)c(NC(C)C(=O)O)c1 | alpha | 3.11102 | 283.327 | TRUE | FALSE |
| CHEMBL280563 | N[C@H](C(=O)O)[C@H]1C[C@@H]1C(=O)O | alpha | -0.881 | 159.141 | FALSE | TRUE |
| CHEMBL280572 | Nc1ccccc1S(=O)(=O)CC(N)C(=O)O | alpha | -0.5456 | 244.272 | TRUE | FALSE |
| CHEMBL280585 | Cc1onc(O)c1C(N)C(=O)O | alpha | -0.22698 | 172.14 | TRUE | FALSE |
| CHEMBL280677 | NC(Cc1cc(F)ccc1CCP(=O)(O)O)C(=O)O | alpha | 0.5003 | 291.215 | TRUE | FALSE |
| CHEMBL280807 | O=C(O)C1NCCc2cc(CP(=O)(O)O)ccc21 | alpha | 0.6357 | 271.209 | TRUE | FALSE |
| CHEMBL280814 | O=C(O)C1CC(CP(=O)(O)O)c2ccccc2N1 | alpha | 1.2167 | 271.209 | TRUE | FALSE |
| CHEMBL280846 | NC(C(=O)O)c1ccc(CP(=O)(O)O)o1 | alpha | 0.0417 | 235.132 | TRUE | FALSE |
| CHEMBL280850 | O=C(O)C1Cc2cc(CP(=O)(O)O)ccc2CN1 | alpha | 0.4632 | 271.209 | TRUE | FALSE |
| CHEMBL280911 | O=C(O)C1CN(CCCCP(=O)(O)O)CCN1 | alpha | -0.6973 | 266.234 | FALSE | FALSE |
| CHEMBL280935 | N/N=C(\NN)N[C@H](C(=O)O)c1ccccc1 | alpha | -0.9051 | 223.236 | TRUE | TRUE |
| CHEMBL28109 | CC(Nc1ccc(C(=O)c2ccccc2)cc1)C(=O)O | alpha | 2.8026 | 269.3 | TRUE | FALSE |
| CHEMBL281190 | O=C(CP(=O)(O)O)[C@H]1CCCN[C@H]1C(=O)O | alpha | -0.814 | 251.175 | FALSE | TRUE |
| CHEMBL281438 | CC1N[C@@H](C(=O)O)C(C)(C)S1 | alpha | 0.9006 | 175.253 | FALSE | TRUE |
| CHEMBL281485 | CC([C@H]1CCN[C@@H](C(=O)O)C1)P(=O)(O)O | alpha | 0.0054 | 237.192 | FALSE | TRUE |
| CHEMBL281641 | Cc1ccc(NC(C)C(=O)O)c(C(=O)c2ccccc2)c1 | alpha | 3.11102 | 283.327 | TRUE | FALSE |
| CHEMBL28171 | Cc1ccc(C(=O)c2ccccc2NC(C)C(=O)O)cc1 | alpha | 3.11102 | 283.327 | TRUE | FALSE |
| CHEMBL281842 | Cc1cc(C)c(CCP(=O)(O)O)c(CC(N)C(=O)O)c1 | alpha | 0.97804 | 301.279 | TRUE | FALSE |
| CHEMBL281854 | NC(Cc1cc(-c2ccccc2)ccc1CCP(=O)(O)O)C(=O)O | alpha | 2.0282 | 349.323 | TRUE | FALSE |
| CHEMBL281856 | NC(Cc1cc(C(F)(F)F)ccc1CCP(=O)(O)O)C(=O)O | alpha | 1.38 | 341.222 | TRUE | FALSE |
| CHEMBL282119 | NC(C(=O)O)c1ccc(CP(=O)(O)O)c2ccccc12 | alpha | 1.6019 | 295.231 | TRUE | FALSE |
| CHEMBL282162 | C[C@@H]1C[C@H](CP(=O)(O)O)C[C@H](C(=O)O)N1 | alpha | 0.0054 | 237.192 | FALSE | TRUE |
| CHEMBL28253 | O=C(O)[C@H]1CCCN[C@@H]1C(=O)O | alpha | -0.4762 | 173.168 | FALSE | TRUE |
| CHEMBL28259 | N[C@H](C(=O)O)[C@H](O)[C@H](O)C(=O)O | alpha | -2.7953 | 179.128 | FALSE | TRUE |
| CHEMBL282656 | O=C(O)[C@@H]1NCCC[C@@H]1C(O)CP(=O)(O)O | alpha | -1.0222 | 253.191 | FALSE | TRUE |
| CHEMBL282842 | N[C@H](C(=O)O)[C@@H]1C[C@@H]1C(=O)O | alpha | -0.881 | 159.141 | FALSE | TRUE |
| CHEMBL282983 | O=C(O)CCCN1CCN[C@@H](C(=O)O)C1 | alpha | -0.7904 | 216.237 | FALSE | TRUE |
| CHEMBL283004 | CC(NCc1ccccc1CP(=O)(O)O)C(=O)O | alpha | 0.927 | 273.225 | TRUE | FALSE |
| CHEMBL283032 | O=C(O)[C@H]1C[C@H](/C=C/CP(=O)(O)O)CCN1 | alpha | 0.1731 | 249.203 | FALSE | TRUE |
| CHEMBL283198 | COc1ccc(C(=O)c2ccccc2NC(C)C(=O)O)cc1 | alpha | 2.8112 | 299.326 | TRUE | FALSE |
| CHEMBL283725 | O=C(O)[C@@H]1CN[C@H]1C(=O)O | alpha | -1.2564 | 145.114 | FALSE | TRUE |
| CHEMBL283864 | CCCC(Nc1ccc(C(=O)O)cc1)C(=O)O | alpha | 2.05 | 237.255 | TRUE | FALSE |
| CHEMBL283999 | N=C(N)NNC(Cc1ccccc1)C(=O)O | alpha | -0.33003 | 222.248 | TRUE | FALSE |
| CHEMBL284445 | C[C@H]1CN[C@@H](C(=O)O)C[C@H]1CP(=O)(O)O | alpha | -0.1371 | 237.192 | FALSE | TRUE |
| CHEMBL28468 | Cc1ccc(N)c(S(=O)(=O)CC(N)C(=O)O)c1 | alpha | -0.23718 | 258.299 | TRUE | FALSE |
| CHEMBL284895 | NC(C(=O)O)c1cc(O)no1 | alpha | -0.5354 | 158.113 | TRUE | FALSE |
| CHEMBL28500 | CC(NCc1cccc(CP(=O)(O)O)c1)C(=O)O | alpha | 0.927 | 273.225 | TRUE | FALSE |
| CHEMBL28501 | O=C(O)C1Cc2c(cccc2P(=O)(O)O)CN1 | alpha | -0.4115 | 257.182 | TRUE | FALSE |
| CHEMBL28523 | O=C(O)[C@H]1C[C@H](C/C=C/P(=O)(O)O)CCN1 | alpha | 0.5207 | 249.203 | FALSE | TRUE |
| CHEMBL285369 | N[C@H](C(=O)O)[C@H]1C[C@H]1C(=O)O | alpha | -0.881 | 159.141 | FALSE | TRUE |
| CHEMBL285641 | O=C(O)C(NS(=O)(=O)c1ccc(F)cc1)c1ccccc1 | alpha | 1.9299 | 309.318 | TRUE | FALSE |
| CHEMBL28582 | NC(Cc1ccccc1P(=O)(O)O)C(=O)O | alpha | -0.556 | 245.171 | TRUE | FALSE |
| CHEMBL28647 | O=C(O)[C@H]1CCN[C@H](C(=O)O)C1 | alpha | -0.4762 | 173.168 | FALSE | TRUE |
| CHEMBL286622 | O=C(O)C(NS(=O)(=O)c1ccccc1)c1ccccc1 | alpha | 1.7908 | 291.328 | TRUE | FALSE |
| CHEMBL286883 | O=C(O)[C@H](Cc1ccc(-c2cccnc2)cc1)NCP(=O)(O)O | alpha | 1.4691 | 336.284 | TRUE | TRUE |
| CHEMBL28713 | O=C(O)C1NCCc2ccc(P(=O)(O)O)cc21 | alpha | -0.239 | 257.182 | TRUE | FALSE |
| CHEMBL28744 | NC(C(=O)O)c1cc(CP(=O)(O)O)cc(-c2ccccc2)c1 | alpha | 2.1157 | 321.269 | TRUE | FALSE |
| CHEMBL28747 | O=C(O)C1CN(CCCP(=O)(O)O)C(=O)CN1 | alpha | -1.5608 | 266.19 | FALSE | FALSE |
| CHEMBL287624 | NC(C(=O)O)c1cccc(CCP(=O)(O)O)c1 | alpha | 0.4912 | 259.198 | TRUE | FALSE |
| CHEMBL287737 | CCNC(C(=O)O)c1cc(O)no1 | alpha | 0.1154 | 186.167 | TRUE | FALSE |
| CHEMBL287785 | O=C(O)C(NCc1ccccc1)c1cc(O)no1 | alpha | 1.2957 | 248.238 | TRUE | FALSE |
| CHEMBL287851 | O=C(O)C1NCCc2cc(O)c(O)cc21 | alpha | 0.3692 | 209.201 | TRUE | FALSE |
| CHEMBL288031 | NC(Cc1nc2ccccc2n1CCP(=O)(O)O)C(=O)O | alpha | 0.1684 | 313.25 | TRUE | FALSE |
| CHEMBL288035 | NC(CCSCCCCc1c[nH]c2ccccc12)C(=O)O | alpha | 3.0258 | 306.431 | TRUE | FALSE |
| CHEMBL288364 | O=C(O)[C@@H](Cc1ccc(-c2ccccc2)cc1)NCP(=O)(O)O | alpha | 2.0741 | 335.296 | TRUE | TRUE |
| CHEMBL28838 | O=C(O)C1CN(C2CCC(P(=O)(O)O)C2)CCN1 | alpha | -0.5564 | 278.245 | FALSE | FALSE |
| CHEMBL288475 | NC(Cc1nc2ccccc2n1CC(=O)O)C(=O)O | alpha | 0.0753 | 263.253 | TRUE | FALSE |
| CHEMBL288590 | NC(Cc1cccc(P(=O)(O)O)c1)C(=O)O | alpha | -0.556 | 245.171 | TRUE | FALSE |
| CHEMBL288651 | NC(=O)[C@@H]1C[C@H](C(=O)O)N1 | alpha | -1.7132 | 144.13 | FALSE | TRUE |
| CHEMBL288908 | N[C@H](Cc1nc2ccccc2cc1CP(=O)(O)O)C(=O)O | alpha | 0.8669 | 310.246 | TRUE | TRUE |
| CHEMBL28900 | O=C(O)C1CC(CP(=O)(O)O)C2CCCCC2N1 | alpha | 0.7856 | 277.257 | FALSE | FALSE |
| CHEMBL289121 | NC(CCCc1ccccc1CP(=O)(O)O)C(=O)O | alpha | 1.0989 | 287.252 | TRUE | FALSE |
| CHEMBL28931 | O=C(O)C1CC(CCP(=O)(O)O)C2CCCCC2N1 | alpha | 1.1757 | 291.284 | FALSE | FALSE |
| CHEMBL28994 | O=C(O)C1CN(Cc2ccccc2CP(=O)(O)O)CCN1 | alpha | 0.2227 | 314.278 | TRUE | FALSE |
| CHEMBL290155 | O=C(O)C(Cc1ccccc1-c1ccccc1)NCP(=O)(O)O | alpha | 2.0741 | 335.296 | TRUE | FALSE |
| CHEMBL290293 | CC(C)([C@@H]1CCN[C@H](C(=O)O)C1)P(=O)(O)O | alpha | 0.3955 | 251.219 | FALSE | TRUE |
| CHEMBL290437 | C=C(C)[C@H]1CN[C@H](C(=O)O)[C@H]1CP(=O)(O)O | alpha | 0.029 | 249.203 | FALSE | TRUE |
| CHEMBL29072 | O=C(O)C1Cc2c(cccc2CP(=O)(O)O)CN1 | alpha | 0.4632 | 271.209 | TRUE | FALSE |
| CHEMBL29081 | NC(Cc1cccc(C(Br)P(=O)(O)O)c1)C(=O)O | alpha | 1.2122 | 338.094 | TRUE | FALSE |
| CHEMBL291210 | CSCC(C)C(N)C(=O)O | alpha | 0.3974 | 163.242 | FALSE | FALSE |
| CHEMBL291274 | Cc1onc(OCC(=O)O)c1CC(N)C(=O)O | alpha | -0.59918 | 244.203 | TRUE | FALSE |
| CHEMBL291301 | O=C(O)CC[C@H](NP(=O)(O)c1ccccc1)C(=O)O | alpha | 0.4049 | 287.208 | TRUE | TRUE |
| CHEMBL29137 | Nc1c(F)cccc1S(=O)(=O)CC(N)C(=O)O | alpha | -0.4065 | 262.262 | TRUE | FALSE |
| CHEMBL291491 | N[C@@H](C[C@@H](C/C=C/c1ccc(C(F)(F)F)cc1)C(=O)O)C(=O)O | alpha | 2.6115 | 331.29 | TRUE | TRUE |
| CHEMBL291538 | N[C@H](Cc1cccc(-c2ccccc2)c1)C(=O)O | alpha | 2.308 | 241.29 | TRUE | TRUE |
| CHEMBL29178 | O=C(O)C1CN(Cc2ccccc2P(=O)(O)O)CCN1 | alpha | -0.652 | 300.251 | TRUE | FALSE |
| CHEMBL29184 | NC(C(=O)O)c1cccc(P(=O)(O)O)c1 | alpha | -0.426 | 231.144 | TRUE | FALSE |
| CHEMBL291856 | N[C@@H](C[Se]Cc1ccc(Cl)cc1)C(=O)O | alpha | 1.3744 | 292.624 | TRUE | TRUE |
| CHEMBL291902 | O=C(O)[C@H]1NCC[C@H]1C(=O)O | alpha | -0.8663 | 159.141 | FALSE | TRUE |
| CHEMBL292117 | N[C@@H](C(=O)O)[C@@H]1[C@@H](C(=O)O)C1(F)F | alpha | -0.6358 | 195.121 | FALSE | TRUE |
| CHEMBL29244 | O=C(O)C1CN(CP(=O)(O)O)CCN1 | alpha | -1.52 | 224.153 | FALSE | FALSE |
| CHEMBL292685 | Cc1onc(O)c1[C@H](N)C(=O)O | alpha | -0.22698 | 172.14 | TRUE | TRUE |
| CHEMBL292745 | Cc1ccc(/C=C/C[C@H](C[C@H](N)C(=O)O)C(=O)O)cc1 | alpha | 1.90112 | 277.32 | TRUE | TRUE |
| CHEMBL292896 | N[C@@H](C[C@@H](C/C=C/c1ccc(-c2ccccc2)cc1)C(=O)O)C(=O)O | alpha | 3.2597 | 339.391 | TRUE | TRUE |
| CHEMBL293118 | N[C@@H](C[C@@H](C/C=C/c1ccc(Cl)c(Cl)c1)C(=O)O)C(=O)O | alpha | 2.8995 | 332.183 | TRUE | TRUE |
| CHEMBL293275 | NC(=O)[C@H]1C[C@@H](C(=O)O)Nc2cc(Cl)cc(Cl)c21 | alpha | 1.8311 | 289.118 | TRUE | TRUE |
| CHEMBL29340 | NC(Cc1ccccc1CCP(=O)(O)O)C(=O)O | alpha | 0.3612 | 273.225 | TRUE | FALSE |
| CHEMBL293473 | NC(CC(N)C(=O)O)CP(=O)(O)O | alpha | -1.7066 | 212.142 | FALSE | FALSE |
| CHEMBL29348 | O=C(O)CCc1cccc2c1CCNC2C(=O)O | alpha | 0.9752 | 249.266 | TRUE | FALSE |
| CHEMBL293523 | O=C(O)C[C@H]1C[C@H](C(=O)O)Nc2cc(Cl)cc(Cl)c21 | alpha | 2.8205 | 304.129 | TRUE | TRUE |
| CHEMBL293548 | N[C@@H](C[C@@H](C/C=C/c1ccccc1Cl)C(=O)O)C(=O)O | alpha | 2.2461 | 297.738 | TRUE | TRUE |
| CHEMBL293753 | N[C@@H](C(=O)O)[C@H]1[C@H](C(=O)O)C1(F)F | alpha | -0.6358 | 195.121 | FALSE | TRUE |
| CHEMBL29378 | O=C(O)C1NCCc2ccc(CP(=O)(O)O)cc21 | alpha | 0.6357 | 271.209 | TRUE | FALSE |
| CHEMBL293829 | N#Cc1ccc(/C=C/C[C@H](C[C@H](N)C(=O)O)C(=O)O)cc1 | alpha | 1.46438 | 288.303 | TRUE | TRUE |
| CHEMBL294005 | N[C@H](C(=O)O)[C@@H]1[C@H](C(=O)O)C1(F)F | alpha | -0.6358 | 195.121 | FALSE | TRUE |
| CHEMBL294139 | C/C=C/C[C@H](C[C@H](N)C(=O)O)C(=O)O | alpha | 0.4554 | 201.222 | FALSE | TRUE |
| CHEMBL29418 | O=C(O)C1NCCc2ccc(CCP(=O)(O)O)cc21 | alpha | 0.6782 | 285.236 | TRUE | FALSE |
| CHEMBL294300 | N[C@@H]1C[C@H](C(=O)O)Nc2cc(Cl)cc(Cl)c21 | alpha | 2.262 | 261.108 | TRUE | TRUE |
| CHEMBL29432 | NC(C(=O)O)c1ccc(CCCP(=O)(O)O)cc1 | alpha | 0.8813 | 273.225 | TRUE | FALSE |
| CHEMBL294419 | NC(=O)[C@H]1C[C@H](C(=O)O)Nc2cc(Cl)cc(Cl)c21 | alpha | 1.8311 | 289.118 | TRUE | TRUE |
| CHEMBL29446 | N[C@@H](CNCP(=O)(O)O)C(=O)O | alpha | -1.8769 | 198.115 | FALSE | TRUE |
| CHEMBL29465 | CC(C)([C@H]1CCN[C@@H](C(=O)O)C1)P(=O)(O)O | alpha | 0.3955 | 251.219 | FALSE | TRUE |
| CHEMBL294683 | N[C@@H](C[C@@H](C/C=C/c1ccc(F)cc1)C(=O)O)C(=O)O | alpha | 1.7318 | 281.283 | TRUE | TRUE |
| CHEMBL294697 | Cc1cc(C)c2c(c1)NC(C(=O)O)CC2=O | alpha | 1.75494 | 219.24 | TRUE | FALSE |
| CHEMBL295021 | N[C@H](Cc1nc2cccc(Cl)c2cc1CP(=O)(O)O)C(=O)O | alpha | 1.5203 | 344.691 | TRUE | TRUE |
| CHEMBL295052 | Cn1cc(CC(N)C(=O)O)nc1C(C)(C)C | alpha | 0.672 | 225.292 | TRUE | FALSE |
| CHEMBL29520 | NC(CCCCC(N)C(=O)O)C(=O)O | alpha | -0.6294 | 204.226 | FALSE | FALSE |
| CHEMBL295310 | NC(Cc1cc(OCC(=O)O)no1)C(=O)O | alpha | -0.9076 | 230.176 | TRUE | FALSE |
| CHEMBL295647 | NC(=O)[C@@H]1c2ccc(Cl)cc2N[C@H]1C(=O)O | alpha | 0.7876 | 240.646 | TRUE | TRUE |
| CHEMBL295692 | NC(Cc1c(OCC(=O)O)noc1-c1cccs1)C(=O)O | alpha | 0.8209 | 312.303 | TRUE | FALSE |
| CHEMBL295778 | CCCC(CCC)c1onc(OCC(=O)O)c1CC(N)C(=O)O | alpha | 1.7762 | 328.365 | TRUE | FALSE |
| CHEMBL29584 | O=C(O)C1NCCC2CCC(P(=O)(O)O)CC21 | alpha | 0.3955 | 263.23 | FALSE | FALSE |
| CHEMBL29587 | NC(CCCCP(=O)(O)O)C(=O)O | alpha | -0.2537 | 211.154 | FALSE | FALSE |
| CHEMBL296048 | NC(Cc1c[nH]c(C2CCCC2)n1)C(=O)O | alpha | 1.0217 | 223.276 | TRUE | FALSE |
| CHEMBL296108 | NC(Cc1c[nH]c(C2CCCCC2)n1)C(=O)O | alpha | 1.4118 | 237.303 | TRUE | FALSE |
| CHEMBL29613 | Cc1cc(C(N)C(=O)O)c(C)cc1CP(=O)(O)O | alpha | 1.06554 | 273.225 | TRUE | FALSE |
| CHEMBL29624 | O=C(O)C1CN(C(c2ccccc2)P(=O)(O)O)CCN1 | alpha | 0.2213 | 300.251 | TRUE | FALSE |
| CHEMBL296325 | CC(C)(C)c1ncc(CC(N)C(=O)O)n1Cc1ccccc1 | alpha | 2.1833 | 301.39 | TRUE | FALSE |
| CHEMBL29634 | NC(Cc1ccccc1CP(=O)(O)O)C(=O)O | alpha | 0.3187 | 259.198 | TRUE | FALSE |
| CHEMBL296485 | Cc1ccc2cc(CP(=O)(O)O)c(C[C@@H](N)C(=O)O)nc2c1 | alpha | 1.17532 | 324.273 | TRUE | TRUE |
| CHEMBL296705 | CC(C)(C)c1onc(OCC(=O)O)c1CC(N)C(=O)O | alpha | 0.3899 | 286.284 | TRUE | FALSE |
| CHEMBL29687 | NC(C/C=C/P(=O)(O)O)C(=O)O | alpha | -0.5202 | 195.111 | FALSE | FALSE |
| CHEMBL29692 | O=C(O)[C@H]1C[C@@H](C/C=C/P(=O)(O)O)CCN1 | alpha | 0.5207 | 249.203 | FALSE | TRUE |
| CHEMBL29698 | NC(C[S+]([O-])CP(=O)(O)O)C(=O)O | alpha | -1.7178 | 231.166 | FALSE | FALSE |
| CHEMBL297209 | Cc1nc(CC(N)C(=O)O)c[nH]1 | alpha | -0.32748 | 169.184 | TRUE | FALSE |
| CHEMBL297283 | N[C@@H](C/C(=C\c1ccc(Cl)cc1)C(=O)O)C(=O)O | alpha | 1.61 | 269.684 | TRUE | TRUE |
| CHEMBL297411 | N[C@H](Cc1nc2ccc(F)cc2cc1CP(=O)(O)O)C(=O)O | alpha | 1.006 | 328.236 | TRUE | TRUE |
| CHEMBL297591 | CS[C@H](C)C[C@H](N)C(=O)O | alpha | 0.5399 | 163.242 | FALSE | TRUE |
| CHEMBL297757 | Cn1c(CC(N)C(=O)O)cnc1C1CCCCC1 | alpha | 1.4222 | 251.33 | TRUE | FALSE |
| CHEMBL297853 | N[C@H](Cc1nc2ccncc2cc1CP(=O)(O)O)C(=O)O | alpha | 0.2619 | 311.234 | TRUE | TRUE |
| CHEMBL29788 | NC(C(=O)O)c1ccc(CP(=O)(O)O)cc1Br | alpha | 1.2112 | 324.067 | TRUE | FALSE |
| CHEMBL297919 | CCCC/C=C(/C[C@H](N)C(=O)O)C(=O)O | alpha | 0.9896 | 215.249 | FALSE | TRUE |
| CHEMBL29811 | C/C(=C\C(N)C(=O)O)CP(=O)(O)O | alpha | -0.4777 | 209.138 | FALSE | FALSE |
| CHEMBL298242 | NC(Cc1cc2ccccc2cc1CP(=O)(O)O)C(=O)O | alpha | 1.4719 | 309.258 | TRUE | FALSE |
| CHEMBL298310 | C=CCn1cnc(CC(N)C(=O)O)c1 | alpha | 0.0235 | 195.222 | TRUE | FALSE |
| CHEMBL298404 | NC(N)=Nc1ccccc1CC(N)C(=O)O | alpha | -0.4541 | 222.248 | TRUE | FALSE |
| CHEMBL298435 | O=C(O)C[C@@H]1c2ccc(Cl)cc2N[C@H]1C(=O)O | alpha | 1.777 | 255.657 | TRUE | TRUE |
| CHEMBL298490 | CCCCc1onc(OCC(=O)O)c1CC(N)C(=O)O | alpha | 0.435 | 286.284 | TRUE | FALSE |
| CHEMBL29877 | O=C(O)C1Cc2c(CCP(=O)(O)O)cccc2CN1 | alpha | 0.5057 | 285.236 | TRUE | FALSE |
| CHEMBL298779 | CC(C)=C(C[C@H](N)C(=O)O)C(=O)O | alpha | 0.2094 | 187.195 | FALSE | TRUE |
| CHEMBL298800 | CC(C)/C=C(/C[C@H](N)C(=O)O)C(=O)O | alpha | 0.4554 | 201.222 | FALSE | TRUE |
| CHEMBL29884 | NC(CC(=O)NCP(=O)(O)O)C(=O)O | alpha | -1.9602 | 226.125 | FALSE | FALSE |
| CHEMBL299180 | CC(C)/C=C(\C[C@H](N)C(=O)O)C(=O)O | alpha | 0.4554 | 201.222 | FALSE | TRUE |
| CHEMBL299243 | Cc1onc(O)c1[C@@H](N)C(=O)O | alpha | -0.22698 | 172.14 | TRUE | TRUE |
| CHEMBL299291 | N[C@@H](C/C(=C\C1CCCC1)C(=O)O)C(=O)O | alpha | 0.9896 | 227.26 | FALSE | TRUE |
| CHEMBL299365 | CC(C)C/C=C(\C[C@H](N)C(=O)O)C(=O)O | alpha | 0.8455 | 215.249 | FALSE | TRUE |
| CHEMBL299449 | C=C(Cl)C[C@H](C[C@H](N)C(=O)O)C(=O)O | alpha | 0.6318 | 221.64 | FALSE | TRUE |
| CHEMBL29975 | CC([C@H]1CCN[C@H](C(=O)O)C1)P(=O)(O)O | alpha | 0.0054 | 237.192 | FALSE | TRUE |
| CHEMBL30012 | NC(C(=O)O)c1cccc(C(=O)O)c1 | alpha | 0.4692 | 195.174 | TRUE | FALSE |
| CHEMBL30021 | NC(C(=O)O)c1cccc(CP(=O)(O)O)c1 | alpha | 0.4487 | 245.171 | TRUE | FALSE |
| CHEMBL300368 | CSc1ccc(/C=C/C[C@H](C[C@H](N)C(=O)O)C(=O)O)cc1 | alpha | 2.3146 | 309.387 | TRUE | TRUE |
| CHEMBL300768 | CCCC(=O)N[C@H]1C[C@H](C(=O)O)Nc2cc(Cl)cc(Cl)c21 | alpha | 3.2196 | 331.199 | TRUE | TRUE |
| CHEMBL300928 | C/C=C/C=C(/C[C@H](N)C(=O)O)C(=O)O | alpha | 0.3755 | 199.206 | FALSE | TRUE |
| CHEMBL30119 | O=C(O)C1Cc2ccc(P(=O)(O)O)cc2CN1 | alpha | -0.4115 | 257.182 | TRUE | FALSE |
| CHEMBL30127 | C[C@H]1[C@@H](CP(=O)(O)O)CCN[C@H]1C(=O)O | alpha | -0.1371 | 237.192 | FALSE | TRUE |
| CHEMBL30140 | O=C(O)C1Cc2ccc(CCP(=O)(O)O)cc2CN1 | alpha | 0.5057 | 285.236 | TRUE | FALSE |
| CHEMBL30149 | O=C(O)C1Cc2ccc(CP(=O)(O)O)cc2CN1 | alpha | 0.4632 | 271.209 | TRUE | FALSE |
| CHEMBL301536 | N[C@@H](C[C@@H](C/C=C/c1ccc2ccccc2c1)C(=O)O)C(=O)O | alpha | 2.7459 | 313.353 | TRUE | TRUE |
| CHEMBL30161 | O=C(O)[C@H]1C[C@H](CP(=O)(O)O)CCN1 | alpha | -0.3831 | 223.165 | FALSE | TRUE |
| CHEMBL30163 | NC(C#CP(=O)(O)O)C(=O)O | alpha | -1.4631 | 179.068 | FALSE | FALSE |
| CHEMBL301915 | N[C@@H](C[C@@H](C/C=C/c1ccc(Br)cc1)C(=O)O)C(=O)O | alpha | 2.3552 | 342.189 | TRUE | TRUE |
| CHEMBL30201 | COc1cccc(S(=O)(=O)CC(N)C(=O)O)c1N | alpha | -0.537 | 274.298 | TRUE | FALSE |
| CHEMBL302212 | COc1ccc(CSC[C@H](N)C(=O)O)cc1 | alpha | 1.3403 | 241.312 | TRUE | TRUE |
| CHEMBL302402 | CCCC#CC[C@H](C[C@H](N)C(=O)O)C(=O)O | alpha | 0.6828 | 227.26 | FALSE | TRUE |
| CHEMBL302680 | CC(C)c1ccc(/C=C/C[C@H](C[C@H](N)C(=O)O)C(=O)O)cc1 | alpha | 2.7161 | 305.374 | TRUE | TRUE |
| CHEMBL302744 | NC(CCCC(N)(CCl)C(=O)O)C(=O)O | alpha | -0.4105 | 238.671 | FALSE | FALSE |
| CHEMBL302783 | N[C@H](CCCCCP(=O)(O)O)C(=O)O | alpha | 0.1364 | 225.181 | FALSE | TRUE |
| CHEMBL302828 | CC(C)[Se]C[C@H](N)C(=O)O | alpha | 0.3491 | 210.135 | FALSE | TRUE |
| CHEMBL30285 | N[C@H](C(=O)O)c1cc(O)no1 | alpha | -0.5354 | 158.113 | TRUE | TRUE |
| CHEMBL302889 | NC(CC(O)CP(=O)(O)O)C(=O)O | alpha | -1.673 | 213.126 | FALSE | FALSE |
| CHEMBL303143 | C[C@@H](O)[C@H]1C(=O)N2C(C(=O)O)=C(CSC[C@H](N)C(=O)O)S[C@H]12 | alpha | -0.6602 | 348.402 | FALSE | TRUE |
| CHEMBL303393 | C[C@]1(C(=O)CP(=O)(O)O)CCCN[C@H]1C(=O)O | alpha | -0.4239 | 265.202 | FALSE | TRUE |
| CHEMBL3037879 | C[C@H](N[C@H](C(=O)O)[C@@H]1CCCN1)C(=O)N1CCC[C@H]1C(=O)O | alpha | -0.7547 | 313.354 | FALSE | TRUE |
| CHEMBL303862 | NC(CCCC(N)(CF)C(=O)O)C(=O)O | alpha | -0.6798 | 222.216 | FALSE | FALSE |
| CHEMBL3039035 | N[C@@H](C(=O)O)[C@@H](O)[C@H](O)[C@H](O)CO | alpha | -3.5267 | 195.171 | FALSE | TRUE |
| CHEMBL3040418 | N[C@H](C[C@H]1CC(C(=O)O)=NN1)C(=O)O | alpha | -1.4091 | 201.182 | FALSE | TRUE |
| CHEMBL3040423 | N[C@H](C[C@@H]1CC(C(=O)O)=NN1)C(=O)O | alpha | -1.4091 | 201.182 | FALSE | TRUE |
| CHEMBL3040467 | N[C@H](C[C@@H]1CC(C(=O)O)=NN1Cc1ccccc1)C(=O)O | alpha | 0.5035 | 291.307 | TRUE | TRUE |
| CHEMBL3040512 | N[C@H](C[C@H]1CC(C(=O)O)=NN1Cc1ccccc1)C(=O)O | alpha | 0.5035 | 291.307 | TRUE | TRUE |
| CHEMBL304434 | CC/C=C/C[C@H](C[C@H](N)C(=O)O)C(=O)O | alpha | 0.8455 | 215.249 | FALSE | TRUE |
| CHEMBL304488 | N[C@@H](C[Se]Cc1ccc(Cl)c(Cl)c1)C(=O)O | alpha | 2.0278 | 327.069 | TRUE | TRUE |
| CHEMBL304676 | CC[Se]C[C@H](N)C(=O)O | alpha | -0.041 | 196.108 | FALSE | TRUE |
| CHEMBL305346 | COc1cccc(/C=C/C[C@H](C[C@H](N)C(=O)O)C(=O)O)c1 | alpha | 1.6013 | 293.319 | TRUE | TRUE |
| CHEMBL305387 | N[C@@H](C[C@@H](CCCc1ccccc1)C(=O)O)C(=O)O | alpha | 1.5121 | 265.309 | TRUE | TRUE |
| CHEMBL305487 | C[C@]1(C(=O)CP(=O)(O)O)C=CCN[C@H]1C(=O)O | alpha | -0.6479 | 263.186 | FALSE | TRUE |
| CHEMBL305791 | C[C@@H](O)[C@H]1C(=O)N2C(C(=O)O)=C(CC[C@@H](N)C(=O)O)S[C@H]12 | alpha | -0.6132 | 316.335 | FALSE | TRUE |
| CHEMBL306851 | O=C(O)CC[C@@H]1CC[C@H]2CN[C@H](C(=O)O)C[C@H]2C1 | alpha | 1.3302 | 255.314 | FALSE | TRUE |
| CHEMBL308011 | C[C@@H]1CCN[C@@H](C(=O)O)[C@H]1C(=O)CP(=O)(O)O | alpha | -0.568 | 265.202 | FALSE | TRUE |
| CHEMBL310623 | O=C(O)C1C=CCN1 | alpha | -0.401 | 113.116 | FALSE | FALSE |
| CHEMBL310749 | N[C@@H](CC1=N[C@H](C(=O)O)CO1)C(=O)O | alpha | -1.3297 | 202.166 | FALSE | TRUE |
| CHEMBL310897 | NC(Cc1c(O)noc1-c1nccs1)C(=O)O | alpha | 0.4581 | 255.255 | TRUE | FALSE |
| CHEMBL311541 | N[C@@H](CCCNc1ccc(F)cc1[N+](=O)[O-])C(=O)O | alpha | 1.3379 | 271.248 | TRUE | TRUE |
| CHEMBL311632 | O=C(O)[C@@H]1C[C@H]2C[C@H](CCS(=O)(=O)O)CC[C@H]2CN1 | alpha | 0.7433 | 291.369 | FALSE | TRUE |
| CHEMBL311920 | NCC(F)(F)CC(N)C(=O)O | alpha | -0.6176 | 168.143 | FALSE | FALSE |
| CHEMBL3121397 | N[C@H](C(=O)O)[C@@H]1CC(O)=NN1Cc1ccc(C(=O)O)cc1 | alpha | 0.2425 | 293.279 | TRUE | TRUE |
| CHEMBL3121398 | CCN1N=C(O)C[C@@H]1[C@H](N)C(=O)O | alpha | -0.636 | 187.199 | FALSE | TRUE |
| CHEMBL3121399 | N[C@H](C(=O)O)[C@H]1CC(O)=NN1Cc1ccccc1 | alpha | 0.5443 | 249.27 | TRUE | TRUE |
| CHEMBL3121403 | N[C@H](C(=O)O)[C@H]1CC(O)=NN1Cc1cccc(C(=O)O)c1 | alpha | 0.2425 | 293.279 | TRUE | TRUE |
| CHEMBL3121404 | N[C@H](C(=O)O)[C@H]1CC(O)=NN1Cc1ccc(C(=O)O)cc1 | alpha | 0.2425 | 293.279 | TRUE | TRUE |
| CHEMBL3121405 | N[C@H](C(=O)O)C1CC(O)=NO1 | alpha | -0.9413 | 160.129 | FALSE | TRUE |
| CHEMBL3121406 | CCN1N=C(O)C[C@H]1[C@H](N)C(=O)O | alpha | -0.636 | 187.199 | FALSE | TRUE |
| CHEMBL3121407 | N[C@H](C(=O)O)[C@@H]1CC(O)=NN1Cc1ccccc1 | alpha | 0.5443 | 249.27 | TRUE | TRUE |
| CHEMBL3121411 | N[C@H](C(=O)O)[C@@H]1CC(O)=NN1Cc1cccc(C(=O)O)c1 | alpha | 0.2425 | 293.279 | TRUE | TRUE |
| CHEMBL312192 | CC[C@H]1[C@H](C(=O)O)[C@@H]1[C@H](N)C(=O)O | alpha | -0.2449 | 187.195 | FALSE | TRUE |
| CHEMBL312860 | NC(Cc1c(O)noc1-c1cccnc1)C(=O)O | alpha | 0.3966 | 249.226 | TRUE | FALSE |
| CHEMBL312899 | N[C@@H](CCCNc1c([N+](=O)[O-])cccc1[N+](=O)[O-])C(=O)O | alpha | 1.107 | 298.255 | TRUE | TRUE |
| CHEMBL312976 | NC(Cc1cccc(CC(=O)NO)c1)C(=O)O | alpha | -0.3111 | 238.243 | TRUE | FALSE |
| CHEMBL313039 | N[C@H](Cc1c(O)noc1-c1ccccn1)C(=O)O | alpha | 0.3966 | 249.226 | TRUE | TRUE |
| CHEMBL313124 | Cc1c(C(N)C(=O)O)ccc(C(=O)O)c1O | alpha | 0.48322 | 225.2 | TRUE | FALSE |
| CHEMBL3133410 | O=C(O)[C@@H]1C[C@H](O)[C@@H](CO)N1 | alpha | -1.8453 | 161.157 | FALSE | TRUE |
| CHEMBL313526 | Cc1ccc([N+](=O)[O-])c(NCC[C@H](N)C(=O)O)c1 | alpha | 1.11712 | 253.258 | TRUE | TRUE |
| CHEMBL313577 | N[C@H](C/C(CP(=O)(O)O)=N/OCc1ccccc1)C(=O)O | alpha | 0.5389 | 316.25 | TRUE | TRUE |
| CHEMBL313693 | NC(CC1CCCCC1CS(=O)(=O)[O-])C(=O)O.[Na+] | alpha | -2.856 | 287.313 | FALSE | FALSE |
| CHEMBL3137306 | N[C@H](CCC(=O)N[C@H](Cc1c[nH]c2ccccc12)C(=O)O)C(=O)O | alpha | 0.4719 | 333.344 | TRUE | TRUE |
| CHEMBL3138615 | N[C@@H](CSCSC[C@H](N)C(=O)O)C(=O)O | alpha | -0.7659 | 254.333 | FALSE | TRUE |
| CHEMBL314426 | N[C@@H](CCCNc1c(Cl)cccc1[N+](=O)[O-])C(=O)O | alpha | 1.8522 | 287.703 | TRUE | TRUE |
| CHEMBL314690 | O=C(O)C[C@H]1CN[C@@H]1C(=O)O | alpha | -0.8663 | 159.141 | FALSE | TRUE |
| CHEMBL314706 | O=C(O)CN1CCCC(NC(CCc2ccccc2)C(=O)O)C1=O | alpha | 0.7376 | 334.372 | TRUE | FALSE |
| CHEMBL314801 | Cc1ccc([N+](=O)[O-])c(NCCCC[C@H](N)C(=O)O)c1 | alpha | 1.89732 | 281.312 | TRUE | TRUE |
| CHEMBL315203 | N[C@H](Cc1c(O)noc1-c1ccccc1)C(=O)O | alpha | 1.0016 | 248.238 | TRUE | TRUE |
| CHEMBL315268 | Cc1onc(O)c1CC[C@H](N)C(=O)O | alpha | 0.03312 | 200.194 | TRUE | TRUE |
| CHEMBL315416 | NC(Cc1c(O)noc1-c1ccc(Br)o1)C(=O)O | alpha | 1.3571 | 317.095 | TRUE | FALSE |
| CHEMBL315591 | Cc1cc(C(=O)O)ccc1C(N)C(=O)O | alpha | 0.77762 | 209.201 | TRUE | FALSE |
| CHEMBL315663 | CCCCc1c(O)noc1C[C@H](N)C(=O)O | alpha | 0.6772 | 228.248 | TRUE | TRUE |
| CHEMBL315803 | N[C@@H](CC(=O)c1ccccc1)C(=O)O | alpha | 0.6713 | 193.202 | TRUE | TRUE |
| CHEMBL315906 | Cc1cccc(-c2onc(O)c2CC(N)C(=O)O)n1 | alpha | 0.70502 | 263.253 | TRUE | FALSE |
| CHEMBL317007 | O=C(O)C1CN(c2cccc(P(=O)(O)O)c2)CCN1 | alpha | -0.6476 | 286.224 | TRUE | FALSE |
| CHEMBL317028 | NC1=NC[C@H](C[C@H](N)C(=O)O)C1 | alpha | -0.8345 | 171.2 | FALSE | TRUE |
| CHEMBL317372 | C[S+](C)CC(=O)CC[C@H](N)C(=O)O | alpha | -0.3745 | 206.287 | FALSE | TRUE |
| CHEMBL317504 | N[C@@H](CSC(=O)c1ccccc1O)C(=O)O | alpha | 0.6775 | 241.268 | TRUE | TRUE |
| CHEMBL317790 | Cn1nnnc1-c1onc(O)c1CC(N)C(=O)O | alpha | -1.4749 | 254.206 | TRUE | FALSE |
| CHEMBL318467 | NC(Cc1c(O)noc1-c1nc[nH]n1)C(=O)O | alpha | -0.8803 | 239.191 | TRUE | FALSE |
| CHEMBL3185307 | Cl.NC(=O)CC[C@H](Nc1ccc([N+](=O)[O-])cc1)C(=O)O | alpha | 1.1472 | 303.702 | TRUE | TRUE |
| CHEMBL3187160 | CC(NC1=NC(C)(C)Cc2ccccc21)C(=O)O | alpha | 1.8306 | 246.31 | TRUE | FALSE |
| CHEMBL318872 | O=C(O)CCCN1CCNC(C(=O)O)C1 | alpha | -0.7904 | 216.237 | FALSE | FALSE |
| CHEMBL31902 | CC(NC/C=C\CP(=O)(O)O)C(=O)O | alpha | -0.217 | 223.165 | FALSE | FALSE |
| CHEMBL319347 | COC1=CC(=O)C(N[C@@H](CO)C(=O)O)=CC1=O | alpha | -1.4125 | 241.199 | FALSE | TRUE |
| CHEMBL320290 | NC(Cc1ccnn1O)C(=O)O | alpha | -0.9252 | 171.156 | TRUE | FALSE |
| CHEMBL3207368 | CCSCCC(NC1=CC(=O)CC(C)(C)C1)C(=O)O | alpha | 2.4454 | 285.409 | FALSE | FALSE |
| CHEMBL320841 | N/C(=N\CCC[C@@H](N)C(=O)O)NCCc1c[nH]cn1 | alpha | -0.9514 | 268.321 | TRUE | TRUE |
| CHEMBL320959 | NC(C(=O)O)[C@@H]1CC=CC[C@H]1C(=O)O | alpha | 0.0653 | 199.206 | FALSE | TRUE |
| CHEMBL3210004 | CSCCC(NC=C1C(=O)CC(C)(C)CC1=O)C(=O)O | alpha | 1.6244 | 299.392 | FALSE | FALSE |
| CHEMBL321112 | O=C[C@@H](NS(=O)(=O)c1ccccc1Oc1ccccc1)C(=O)O | alpha | 1.4093 | 335.337 | TRUE | TRUE |
| CHEMBL321164 | NC(Cc1cnnn1O)C(=O)O | alpha | -1.5302 | 172.144 | TRUE | FALSE |
| CHEMBL3215866 | Cl.Cl.N=C(N)NC[C@H]1C[C@@H]1[C@H](N)C(=O)O | alpha | -0.63893 | 259.137 | FALSE | TRUE |
| CHEMBL3215977 | Cl.Cl.NCP(=O)(O)CC[C@H](N)C(=O)O | alpha | -0.1814 | 269.065 | FALSE | TRUE |
| CHEMBL3216104 | Cl.Cl.N=C(N)NC[C@@H]1C[C@@H]1[C@@H](N)C(=O)O | alpha | -0.63893 | 259.137 | FALSE | TRUE |
| CHEMBL3216308 | Cl.Cl.N=C(N)NC[C@H]1C[C@H]1[C@@H](N)C(=O)O | alpha | -0.63893 | 259.137 | FALSE | TRUE |
| CHEMBL3216309 | Cl.Cl.N=C(N)NC[C@@H]1C[C@H]1[C@H](N)C(=O)O | alpha | -0.63893 | 259.137 | FALSE | TRUE |
| CHEMBL3216530 | Cl.Cl.N=C(N)NC[C@@H]1C[C@H]1[C@@H](N)C(=O)O | alpha | -0.63893 | 259.137 | FALSE | TRUE |
| CHEMBL3216763 | Cl.Cl.N=C(N)NC[C@H]1C[C@@H]1[C@@H](N)C(=O)O | alpha | -0.63893 | 259.137 | FALSE | TRUE |
| CHEMBL3216764 | Cl.Cl.N=C(N)NC[C@@H]1C[C@@H]1[C@H](N)C(=O)O | alpha | -0.63893 | 259.137 | FALSE | TRUE |
| CHEMBL3216981 | Cl.Cl.N=C(N)NC[C@H]1C[C@H]1[C@H](N)C(=O)O | alpha | -0.63893 | 259.137 | FALSE | TRUE |
| CHEMBL3217070 | Cl.Cl.NCCP(=O)(O)CC[C@H](N)C(=O)O | alpha | -0.1389 | 283.092 | FALSE | TRUE |
| CHEMBL3217090 | Cl.Cl.N[C@@H](CCP(=O)(O)CCC(=O)O)C(=O)O | alpha | 0.3771 | 312.086 | FALSE | TRUE |
| CHEMBL321802 | N/C(=N\CCC[C@H](N)C(=O)O)NCc1c[nH]cn1 | alpha | -0.9939 | 254.294 | TRUE | TRUE |
| CHEMBL321843 | N#CCC[C@H](N)C(=O)O | alpha | -0.29792 | 128.131 | FALSE | TRUE |
| CHEMBL3219495 | C[C@@H](NCCN)C(=O)O | alpha | -0.9922 | 132.163 | FALSE | TRUE |
| CHEMBL322304 | NC(CC1CCCCC1CCP(=O)(O)O)C(=O)O | alpha | 1.1626 | 279.273 | FALSE | FALSE |
| CHEMBL3228255 | Cc1cnc(C)c(O)c1CNC(Cc1ccc(O)c(O)c1)C(=O)O | alpha | 1.60074 | 332.356 | TRUE | FALSE |
| CHEMBL323289 | Cc1ncn(O)c1CC(N)C(=O)O | alpha | -0.61678 | 185.183 | TRUE | FALSE |
| CHEMBL3235131 | CCCn1cnc(CC(NCCN)C(=O)O)c1 | alpha | -0.1629 | 240.307 | TRUE | FALSE |
| CHEMBL3235236 | N[C@H](C/C=C/c1ccccc1C(=O)O)C(=O)O | alpha | 1.2 | 235.239 | TRUE | TRUE |
| CHEMBL3235237 | N[C@H](C/C=C/c1cscc1C(=O)O)C(=O)O | alpha | 1.2615 | 241.268 | TRUE | TRUE |
| CHEMBL3235238 | N[C@H](CCCc1ccccc1C(=O)O)C(=O)O | alpha | 1.1194 | 237.255 | TRUE | TRUE |
| CHEMBL3235239 | N[C@H](CCCc1cscc1C(=O)O)C(=O)O | alpha | 1.1809 | 243.284 | TRUE | TRUE |
| CHEMBL323742 | CC(C)(C)c1onc(O)c1C(N)C(=O)O | alpha | 0.7621 | 214.221 | TRUE | FALSE |
| CHEMBL323830 | NC(Cc1nc2ccccc2nc1/C=C/P(=O)(O)O)C(=O)O | alpha | 0.7326 | 323.245 | TRUE | FALSE |
| CHEMBL3244431 | C[As](C)SC(C)(C)C(N)C(=O)O.Cl | alpha | 1.583 | 289.66 | FALSE | FALSE |
| CHEMBL3244471 | N[C@@H](CCC(=O)Nc1ccc(O)cc1)C(=O)O | alpha | 0.5228 | 238.243 | TRUE | TRUE |
| CHEMBL3245366 | O=C(O)[C@@H]1CSCCN1 | alpha | -0.224 | 147.199 | FALSE | TRUE |
| CHEMBL3245369 | N[C@@H](CSCCSC[C@H](N)C(=O)O)C(=O)O | alpha | -0.7234 | 268.36 | FALSE | TRUE |
| CHEMBL3245980 | CCC(c1c[nH]cn1)C(N)C(=O)O | alpha | 0.3152 | 183.211 | TRUE | FALSE |
| CHEMBL3245981 | CCCCCCC(c1c[nH]cn1)C(N)C(=O)O | alpha | 1.8756 | 239.319 | TRUE | FALSE |
| CHEMBL324686 | Cc1onc(O)c1CCCC(N)C(=O)O | alpha | 0.42322 | 214.221 | TRUE | FALSE |
| CHEMBL324706 | NC(Cc1nc2ccccc2nc1CP(=O)(O)O)C(=O)O | alpha | 0.2619 | 311.234 | TRUE | FALSE |
| CHEMBL3247376 | C[As](C)SC(C)(C)C(N)C(=O)O | alpha | 1.1612 | 253.199 | FALSE | FALSE |
| CHEMBL3247486 | NC(Cc1ccccc1N(CCCl)CCCl)C(=O)O | alpha | 1.925 | 305.205 | TRUE | FALSE |
| CHEMBL3247538 | NCCC(O)C(N)C(=O)O | alpha | -1.892 | 148.162 | FALSE | FALSE |
| CHEMBL3247544 | NC/C=C/C(N)C(=O)O | alpha | -1.0868 | 130.147 | FALSE | FALSE |
| CHEMBL3248521 | N[C@@H](CCC(=O)Nc1cccc(O)c1)C(=O)O | alpha | 0.5228 | 238.243 | TRUE | TRUE |
| CHEMBL3248565 | N[C@@H](CCC(=O)Nc1ccc(O)c(O)c1)C(=O)O | alpha | 0.2284 | 254.242 | TRUE | TRUE |
| CHEMBL3248566 | COc1ccc(NC(=O)CC[C@H](N)C(=O)O)cc1 | alpha | 0.8258 | 252.27 | TRUE | TRUE |
| CHEMBL3248567 | N[C@@H](CCC(=O)Nc1ccc2c(c1)OCO2)C(=O)O | alpha | 0.5459 | 266.253 | TRUE | TRUE |
| CHEMBL3248568 | N[C@@H](CCC(=O)Nc1cc(O)ccc1O)C(=O)O | alpha | 0.2284 | 254.242 | TRUE | TRUE |
| CHEMBL3248569 | N[C@@H](CC(=O)Nc1ccc(O)cc1)C(=O)O | alpha | 0.1327 | 224.216 | TRUE | TRUE |
| CHEMBL324862 | NC(Cc1nc2cc(Cl)c(Cl)cc2[nH]1)C(=O)O | alpha | 1.8241 | 274.107 | TRUE | FALSE |
| CHEMBL3250951 | N[C@@H](CS(=O)(=O)NO)C(=O)O | alpha | -2.2931 | 184.173 | FALSE | TRUE |
| CHEMBL3250952 | N[C@@H](CS(=O)(=O)NCCCl)C(=O)O | alpha | -1.4435 | 230.673 | FALSE | TRUE |
| CHEMBL3250956 | N[C@@H](CS(N)(=O)=O)C(=O)O | alpha | -2.3132 | 168.174 | FALSE | TRUE |
| CHEMBL3251297 | N[C@@H](CC(=O)NCc1ccc(S(=O)(=O)F)cc1)C(=O)O | alpha | -0.237 | 304.299 | TRUE | TRUE |
| CHEMBL3251671 | N[C@@H](Cc1nc[nH]c1[N+](=O)[O-])C(=O)O | alpha | -0.7277 | 200.154 | TRUE | TRUE |
| CHEMBL3251934 | N[C@@H](CC1=CCC=CC1)C(=O)O | alpha | 1.0648 | 167.208 | FALSE | TRUE |
| CHEMBL325291 | NC(Cc1onc(O)c1CCO)C(=O)O | alpha | -1.1306 | 216.193 | TRUE | FALSE |
| CHEMBL325325 | Cc1cc2nc(CC(N)C(=O)O)[nH]c2cc1C | alpha | 1.13414 | 233.271 | TRUE | FALSE |
| CHEMBL325882 | O=C(O)CC1CCCC2(CCNC(C(=O)O)C2)C1 | alpha | 1.4743 | 255.314 | FALSE | FALSE |
| CHEMBL3260778 | Cn1ccn(CC(N)C(=O)O)c1=S | alpha | -0.03201 | 201.251 | TRUE | FALSE |
| CHEMBL326557 | NC(Cc1cc2nccnc2cc1CP(=O)(O)O)C(=O)O | alpha | 0.2619 | 311.234 | TRUE | FALSE |
| CHEMBL327092 | NC(Cc1cc(O)ccc1[N+](=O)[O-])C(=O)O | alpha | 0.2548 | 226.188 | TRUE | FALSE |
| CHEMBL3272401 | NC(CCc1c[nH]cn1)C(=O)O | alpha | -0.2458 | 169.184 | TRUE | FALSE |
| CHEMBL3272603 | NC(Cc1ccc2ccccc2c1Cl)C(=O)O | alpha | 2.4476 | 249.697 | TRUE | FALSE |
| CHEMBL3272604 | NC(Cc1ccc2ccccc2c1Br)C(=O)O | alpha | 2.5567 | 294.148 | TRUE | FALSE |
| CHEMBL3272607 | NC(Cc1ccc(O)cc1Cl)C(=O)O | alpha | 1 | 215.636 | TRUE | FALSE |
| CHEMBL3274159 | CCCc1cc(Oc2c(C)cc(CC(N)C(=O)O)cc2C)ccc1O | alpha | 3.70824 | 343.423 | TRUE | FALSE |
| CHEMBL3274625 | NCC(F)(F)CCC(N)C(=O)O | alpha | -0.2275 | 182.17 | FALSE | FALSE |
| CHEMBL3274626 | CC(C)(CN)CCC(N)C(=O)O.Cl | alpha | 0.5852 | 210.705 | FALSE | FALSE |
| CHEMBL3275220 | Cc1cc(Oc2c(C)cc(C[C@H](N)C(=O)O)cc2C)ccc1O | alpha | 3.06416 | 315.369 | TRUE | TRUE |
| CHEMBL3275221 | Cc1cc(Oc2c(C)cc(C[C@H](N)C(=O)O)cc2C)cc(C)c1O | alpha | 3.37258 | 329.396 | TRUE | TRUE |
| CHEMBL3275993 | Cc1ccc(S(=O)(=O)N[C@@H](Cc2ccccc2)C(=O)O)cc1 | alpha | 1.96922 | 319.382 | TRUE | TRUE |
| CHEMBL3276853 | CC(C)(c1c[nH]cn1)[C@H](N)C(=O)O | alpha | 0.0992 | 183.211 | TRUE | TRUE |
| CHEMBL3276855 | NC(C(=O)O)C(F)(F)C(=O)O | alpha | -0.8818 | 169.083 | FALSE | FALSE |
| CHEMBL3276856 | N[C@@H](C(=O)O)C(F)(F)C(=O)O | alpha | -0.8818 | 169.083 | FALSE | TRUE |
| CHEMBL3276857 | N[C@H](C(=O)O)C(F)(F)C(=O)O | alpha | -0.8818 | 169.083 | FALSE | TRUE |
| CHEMBL3276858 | NC(=O)C(F)(F)C(N)C(=O)O | alpha | -1.4811 | 168.099 | FALSE | FALSE |
| CHEMBL3277364 | N[C@@H](Cc1ccc2[nH]cnc2c1)C(=O)O | alpha | 0.5173 | 205.217 | TRUE | TRUE |
| CHEMBL3277554 | Cc1ccc([C@@H]2N[C@H](C(=O)O)CS2)cc1 | alpha | 1.78322 | 223.297 | TRUE | TRUE |
| CHEMBL3277559 | Cc1ccc(C2NC(C(=O)O)C(C)(C)S2)cc1 | alpha | 2.56182 | 251.351 | TRUE | FALSE |
| CHEMBL328290 | O=C(O)[C@@H]1CN1 | alpha | -0.9572 | 87.078 | FALSE | TRUE |
| CHEMBL328310 | Cn1ncnc1-c1onc(O)c1CC(N)C(=O)O | alpha | -0.8699 | 253.218 | TRUE | FALSE |
| CHEMBL328501 | N[C@@H](C(=O)O)[C@@H]1C[C@@H]1C(=O)O | alpha | -0.881 | 159.141 | FALSE | TRUE |
| CHEMBL328880 | CSC(=NCCC[C@H](N)C(=O)O)SC | alpha | 1.2605 | 236.362 | FALSE | TRUE |
| CHEMBL328984 | NC(C(=O)O)c1ccc(C(=O)O)cc1 | alpha | 0.4692 | 195.174 | TRUE | FALSE |
| CHEMBL3291282 | CCCCCNC(CNC(=O)[C@H](O)C(C)(C)CO)C(=O)O | alpha | -0.285 | 304.387 | FALSE | TRUE |
| CHEMBL329663 | CC(C(=O)CP(=O)(O)O)C(N)C(=O)O | alpha | -1.2188 | 225.137 | FALSE | FALSE |
| CHEMBL329820 | Cc1ccc(NCCC[C@H](N)C(=O)O)c([N+](=O)[O-])c1 | alpha | 1.50722 | 267.285 | TRUE | TRUE |
| CHEMBL329883 | N[C@@H](CCCNc1ccccc1[N+](=O)[O-])C(=O)O | alpha | 1.1988 | 253.258 | TRUE | TRUE |
| CHEMBL329905 | Cc1cc(C(=O)O)c(O)cc1C(N)C(=O)O | alpha | 0.48322 | 225.2 | TRUE | FALSE |
| CHEMBL329918 | CC(=[N+]=[N-])C(=O)CC[C@H](N)C(=O)O | alpha | -0.5617 | 185.183 | FALSE | TRUE |
| CHEMBL330028 | CC(C)C(NS(=O)(=O)c1ccc(-c2ccccc2)cc1)C(=O)O | alpha | 2.7411 | 333.409 | TRUE | FALSE |
| CHEMBL3303794 | NC(=O)CC[C@H](Nc1ccc([N+](=O)[O-])cc1)C(=O)O | alpha | 0.7254 | 267.241 | TRUE | TRUE |
| CHEMBL3305736 | CC(C)(CN)CCC(N)C(=O)O | alpha | 0.1634 | 174.244 | FALSE | FALSE |
| CHEMBL330808 | C[C@@H]1[C@H](C(=O)O)[C@H]1[C@H](N)C(=O)O | alpha | -0.635 | 173.168 | FALSE | TRUE |
| CHEMBL330845 | NCCCCCC/C(=C\C(N)C(=O)O)CP(=O)(O)O | alpha | 0.4116 | 294.288 | FALSE | FALSE |
| CHEMBL3311414 | O=C(O)C1CSC=C(c2ccccc2)N1 | alpha | 1.7746 | 221.281 | TRUE | FALSE |
| CHEMBL331339 | C=C(c1ccc(C(C)(C)C)cc1)[C@H]1CN[C@H](C(=O)O)[C@H]1CC(=O)O | alpha | 2.7608 | 331.412 | TRUE | TRUE |
| CHEMBL331477 | CC(c1ccccc1)[C@H]1CN[C@H](C(=O)O)[C@H]1CC(=O)O | alpha | 1.5536 | 277.32 | TRUE | TRUE |
| CHEMBL331644 | N[C@@H](Cn1nc(Br)c(=O)[nH]c1=O)C(=O)O | alpha | -1.894 | 279.05 | TRUE | TRUE |
| CHEMBL331696 | N[C@@H](Cn1nc(Cl)c(=O)[nH]c1=O)C(=O)O | alpha | -2.0031 | 234.599 | TRUE | TRUE |
| CHEMBL332126 | C=C(C)c1ccc(C(=C)[C@@H]2CN[C@H](C(=O)O)[C@H]2CC(=O)O)cc1 | alpha | 2.4964 | 315.369 | TRUE | TRUE |
| CHEMBL332168 | C=C(c1cccc(OC)c1)[C@H]1CN[C@H](C(=O)O)[C@H]1CC(=O)O | alpha | 1.4719 | 305.33 | TRUE | TRUE |
| CHEMBL332334 | C=C(c1ccc(C)cc1)[C@H]1CN[C@H](C(=O)O)[C@H]1CC(=O)O | alpha | 1.77172 | 289.331 | TRUE | TRUE |
| CHEMBL332467 | C=C(c1ccc(CC)cc1)[C@H]1CN[C@H](C(=O)O)[C@H]1CC(=O)O | alpha | 2.0257 | 303.358 | TRUE | TRUE |
| CHEMBL332818 | C=C(c1cccc(C)c1)[C@H]1CN[C@H](C(=O)O)[C@H]1CC(=O)O | alpha | 1.77172 | 289.331 | TRUE | TRUE |
| CHEMBL333015 | C=C(c1ccc(OC)cc1)[C@H]1CN[C@H](C(=O)O)[C@H]1CC(=O)O | alpha | 1.4719 | 305.33 | TRUE | TRUE |
| CHEMBL333229 | CC/N=C(/N)NCCCC(N)C(=O)O | alpha | -0.8973 | 202.258 | FALSE | FALSE |
| CHEMBL333456 | COC[C@@H]1[C@H](C(=O)O)[C@H]1[C@H](N)C(=O)O | alpha | -1.0085 | 203.194 | FALSE | TRUE |
| CHEMBL3337652 | NCC(=O)Nc1ccc(C[C@H](N)C(=O)O)c(CCC(=O)O)c1 | alpha | -0.4446 | 309.322 | TRUE | TRUE |
| CHEMBL3337653 | CCS(=O)(=O)Nc1ccc(C[C@H](N)C(=O)O)c(CCC(=O)O)c1 | alpha | 0.4198 | 344.389 | TRUE | TRUE |
| CHEMBL3337661 | CCC(=O)Nc1ccc(C[C@H](N)C(=O)O)c(CCC(=O)O)c1 | alpha | 1.0067 | 308.334 | TRUE | TRUE |
| CHEMBL334119 | C=C(c1ccc(C)c(C)c1)[C@H]1CN[C@H](C(=O)O)[C@H]1CC(=O)O | alpha | 2.08014 | 303.358 | TRUE | TRUE |
| CHEMBL334160 | C[C@H]1[C@H](C(=O)O)[C@H]1[C@H](N)C(=O)O | alpha | -0.635 | 173.168 | FALSE | TRUE |
| CHEMBL334583 | O=C1NO[C@H]2CN[C@H](C(=O)O)[C@@H]12 | alpha | -1.911 | 172.14 | FALSE | TRUE |
| CHEMBL334842 | N[C@H](CCc1nsnc1O)C(=O)O | alpha | -0.4118 | 203.223 | TRUE | TRUE |
| CHEMBL3349901 | O=C(O)[C@H]1CN[C@@H](C(=O)O)C1 | alpha | -0.8663 | 159.141 | FALSE | TRUE |
| CHEMBL3358461 | N[C@@H](Cc1ccc([N+](=O)[O-])cc1CCC(=O)O)C(=O)O | alpha | 0.5664 | 282.252 | TRUE | TRUE |
| CHEMBL3358462 | Nc1ccc(C[C@H](N)C(=O)O)c(CCC(=O)O)c1 | alpha | 0.2404 | 252.27 | TRUE | TRUE |
| CHEMBL3359687 | Cl.NC(C(=O)O)c1ccc(-n2cccn2)cc1 | alpha | 1.3785 | 253.689 | TRUE | FALSE |
| CHEMBL336222 | N[C@@H](C/N=c1\c(O)c(O)c1=O)C(=O)O | alpha | -2.3538 | 200.15 | TRUE | TRUE |
| CHEMBL336488 | N[C@H](C[C@H](CC#Cc1ccccc1)C(=O)O)C(=O)O | alpha | 0.931 | 261.277 | TRUE | TRUE |
| CHEMBL336983 | COC[C@@H]1[C@H](C(=O)O)[C@@H]1[C@H](N)C(=O)O | alpha | -1.0085 | 203.194 | FALSE | TRUE |
| CHEMBL337075 | N[C@H](Cc1cc(O)no1)C(=O)O | alpha | -0.6654 | 172.14 | TRUE | TRUE |
| CHEMBL337089 | N[C@H](C[C@H](Cc1ccccc1-c1ccccc1)C(=O)O)C(=O)O | alpha | 2.3989 | 313.353 | TRUE | TRUE |
| CHEMBL337769 | N[C@H](C[C@H](Cc1cc2ccccc2o1)C(=O)O)C(=O)O | alpha | 1.4781 | 277.276 | TRUE | TRUE |
| CHEMBL338894 | O=C(O)[C@H]1C[C@@H](OCc2nn[nH]n2)CCN1 | alpha | -1.0785 | 227.224 | TRUE | TRUE |
| CHEMBL339737 | O=C(O)[C@H]1C[C@@H](COCc2nn[nH]n2)CCN1 | alpha | -0.8309 | 241.251 | TRUE | TRUE |
| CHEMBL3398496 | N[C@@H](C(=O)O)[C@H]1CCCN1.O=C(O)C(F)(F)F | alpha | -0.2164 | 258.196 | FALSE | TRUE |
| CHEMBL3398497 | N[C@H](C(=O)O)[C@H]1CCCN1.O=C(O)C(F)(F)F | alpha | -0.2164 | 258.196 | FALSE | TRUE |
| CHEMBL340085 | NC(C[C@@H]1CCCC[C@H]1CCP(=O)(O)O)C(=O)O | alpha | 1.1626 | 279.273 | FALSE | TRUE |
| CHEMBL3400971 | [11CH3]c1ccc(O)cc1C[C@H](N)C(=O)O | alpha | 0.65502 | 194.2184336 | TRUE | TRUE |
| CHEMBL3400972 | N[C@@H](Cc1cc(O)c(O)cc1[18F])C(=O)O | alpha | 0.1913 | 214.182938 | TRUE | TRUE |
| CHEMBL340224 | C#CC/N=C(/N)NCCCC(N)C(=O)O | alpha | -1.284 | 212.253 | FALSE | FALSE |
| CHEMBL3403589 | CCN1CCNC(C(=O)O)C1 | alpha | -0.6353 | 158.201 | FALSE | FALSE |
| CHEMBL340591 | O=C(O)C1=NO[C@H]2[C@@H]1CN[C@@H]2C(=O)O | alpha | -1.5016 | 200.15 | FALSE | TRUE |
| CHEMBL341344 | O=C(O)[C@H]1C[C@H](Sc2nn[nH]n2)CCN1 | alpha | -0.503 | 229.265 | TRUE | TRUE |
| CHEMBL341556 | Cc1cc(N)c(NC[C@@H](N)C(=O)O)cc1C.Cc1ccc(S(=O)(=O)O)cc1 | alpha | 1.95116 | 395.481 | TRUE | TRUE |
| CHEMBL3417719 | NC(CSCc1cccc2ccccc12)C(=O)O | alpha | 2.4849 | 261.346 | TRUE | FALSE |
| CHEMBL341827 | N[C@H](C[C@H](Cc1cc2ccccc2s1)C(=O)O)C(=O)O | alpha | 1.9466 | 293.344 | TRUE | TRUE |
| CHEMBL34209 | NC(CCSCCCc1c[nH]c2ccccc12)C(=O)O | alpha | 2.6357 | 292.404 | TRUE | FALSE |
| CHEMBL342129 | N[C@H](C[C@H](Cc1ccc(-c2ccsc2)cc1)C(=O)O)C(=O)O | alpha | 2.4604 | 319.382 | TRUE | TRUE |
| CHEMBL342185 | N[C@H](C[C@H](Oc1ccccc1)C(=O)O)C(=O)O | alpha | 0.3206 | 239.227 | TRUE | TRUE |
| CHEMBL342556 | N[C@H](C(=O)O)[C@@H](CC(=O)O)c1ccc(Cl)cc1 | alpha | 1.3102 | 257.673 | TRUE | TRUE |
| CHEMBL342587 | N[C@H](C[C@H](Cc1cccc(C(F)(F)F)c1)C(=O)O)C(=O)O | alpha | 1.7507 | 305.252 | TRUE | TRUE |
| CHEMBL342597 | N[C@H](C[C@H](Cc1ccc(C(F)(F)F)cc1)C(=O)O)C(=O)O | alpha | 1.7507 | 305.252 | TRUE | TRUE |
| CHEMBL342695 | N[C@H](C[C@H](Cc1ccc(-c2ccccc2)cc1)C(=O)O)C(=O)O | alpha | 2.3989 | 313.353 | TRUE | TRUE |
| CHEMBL342979 | N[C@@H](Cc1c[nH]c2c(F)ccc(F)c12)C(=O)O | alpha | 1.4005 | 240.209 | TRUE | TRUE |
| CHEMBL343147 | N[C@H](C[C@H](Cc1cccc(Cl)c1)C(=O)O)C(=O)O | alpha | 1.3853 | 271.7 | TRUE | TRUE |
| CHEMBL343291 | N[C@H](C[C@H](Cc1ccccc1Cl)C(=O)O)C(=O)O | alpha | 1.3853 | 271.7 | TRUE | TRUE |
| CHEMBL343684 | CCCCCCCc1ccc(C2N[C@H](C(=O)O)CS2)cc1 | alpha | 3.9877 | 307.459 | TRUE | TRUE |
| CHEMBL343685 | CCCCCc1ccc(C2N[C@H](C(=O)O)CS2)cc1 | alpha | 3.2075 | 279.405 | TRUE | TRUE |
| CHEMBL343750 | NC(CN1C=CC(=O)C(O)C1)C(=O)O | alpha | -1.8424 | 200.194 | FALSE | FALSE |
| CHEMBL344069 | NC(Cc1c(O)noc1C(=O)O)C(=O)O | alpha | -0.9672 | 216.149 | TRUE | FALSE |
| CHEMBL344311 | CCOc1noc(C(=O)O)c1CC(N)C(=O)O | alpha | -0.2741 | 244.203 | TRUE | FALSE |
| CHEMBL344354 | CCCCCCCCCC1NC(C(=O)O)CS1 | alpha | 3.2428 | 259.415 | FALSE | FALSE |
| CHEMBL345040 | N[C@H](C[C@H](Cc1cccc2ccccc12)C(=O)O)C(=O)O | alpha | 1.8851 | 287.315 | TRUE | TRUE |
| CHEMBL345066 | N[C@H](C[C@H](CNC(=O)c1ccccc1)C(=O)O)C(=O)O | alpha | -0.0808 | 280.28 | TRUE | TRUE |
| CHEMBL345381 | O=C(N[C@@]1(C(=O)O)CN[C@@H](C(=O)O)C1)c1ccccc1 | alpha | -0.3137 | 278.264 | TRUE | TRUE |
| CHEMBL345498 | NC(C(=O)O)c1cc(O)ccc1Cl | alpha | 1.13 | 201.609 | TRUE | FALSE |
| CHEMBL345555 | CNS(=O)(=O)N[C@@H](Cc1ccccc1)C(=O)O | alpha | -0.2639 | 258.299 | TRUE | TRUE |
| CHEMBL3459111 | CCC(Nc1nc(C(F)(F)F)nc2ccccc12)C(=O)O | alpha | 2.9237 | 299.252 | TRUE | FALSE |
| CHEMBL346187 | NC(Cc1snc(O)c1Br)C(=O)O | alpha | 0.5656 | 267.104 | TRUE | FALSE |
| CHEMBL346491 | CCCC1N[C@H](C(=O)O)CS1 | alpha | 0.9022 | 175.253 | FALSE | TRUE |
| CHEMBL346731 | Cc1cc(Oc2c(C)cc(CC(N)C(=O)O)cc2C)cc(C)c1O | alpha | 3.37258 | 329.396 | TRUE | FALSE |
| CHEMBL346976 | NC(CSc1cc(CC(N)C(=O)O)ccc1O)C(=O)O | alpha | -0.1494 | 300.336 | TRUE | FALSE |
| CHEMBL347581 | NC(Cc1ccccc1C(=O)O)C(=O)O | alpha | 0.3392 | 209.201 | TRUE | FALSE |
| CHEMBL347973 | NC(Cc1cc(O)ns1)C(=O)O | alpha | -0.1969 | 188.208 | TRUE | FALSE |
| CHEMBL348230 | CCCCCCCCCC[C@H]1N[C@H](C(=O)O)CS1 | alpha | 3.6329 | 273.442 | FALSE | TRUE |
| CHEMBL348461 | N[C@H](C[C@H](C/C=C/c1ccccc1)C(=O)O)C(=O)O | alpha | 1.5927 | 263.293 | TRUE | TRUE |
| CHEMBL3484942 | CC(C)C(Nc1nc(C(F)(F)F)nc2ccccc12)C(=O)O | alpha | 3.1697 | 313.279 | TRUE | FALSE |
| CHEMBL348670 | O=C(O)[C@@H]1CSC(COc2ccccc2O)N1 | alpha | 0.8867 | 255.295 | TRUE | TRUE |
| CHEMBL348773 | O=C(O)CC1=C(c2ccsc2)CN[C@@H]1C(=O)O | alpha | 1.0328 | 253.279 | TRUE | TRUE |
| CHEMBL348985 | Cc1ccc(C[C@@H](C[C@H](N)C(=O)O)C(=O)O)cc1 | alpha | 1.04032 | 251.282 | TRUE | TRUE |
| CHEMBL349384 | O=C(O)[C@H]1CN[C@H](C(=O)O)C1 | alpha | -0.8663 | 159.141 | FALSE | TRUE |
| CHEMBL349447 | NC(CCCc1cc(O)no1)C(=O)O | alpha | 0.1148 | 200.194 | TRUE | FALSE |
| CHEMBL350208 | NC(CC[Te]c1ccccc1)C(=O)O | alpha | 0.2364 | 306.819 | TRUE | FALSE |
| CHEMBL350497 | Cc1ccc(C[C@H](C[C@H](N)C(=O)O)C(=O)O)cc1 | alpha | 1.04032 | 251.282 | TRUE | TRUE |
| CHEMBL350704 | N[C@@H](C[C@H](CCC(c1ccccc1)c1ccccc1)C(=O)O)C(=O)O | alpha | 3.1015 | 341.407 | TRUE | TRUE |
| CHEMBL351031 | O=C(O)C[C@@H]1[C@@H](C(=O)O)NC[C@@H]1c1ccccc1O | alpha | 0.6231 | 265.265 | TRUE | TRUE |
| CHEMBL351255 | O=C(O)C[C@@H]1[C@@H](C(=O)O)NC[C@H]1c1ccccc1O | alpha | 0.6231 | 265.265 | TRUE | TRUE |
| CHEMBL351653 | N[C@@H](C[C@H](CCCc1ccccc1)C(=O)O)C(=O)O | alpha | 1.5121 | 265.309 | TRUE | TRUE |
| CHEMBL351787 | CC(=N)NCCSC[C@H](N)C(=O)O | alpha | -0.28173 | 205.283 | FALSE | TRUE |
| CHEMBL351832 | NC(CCc1cc(O)no1)C(=O)O | alpha | -0.2753 | 186.167 | TRUE | FALSE |
| CHEMBL351946 | N[C@@H](C[C@H](Cc1ccc(C(F)(F)F)cc1)C(=O)O)C(=O)O | alpha | 1.7507 | 305.252 | TRUE | TRUE |
| CHEMBL3526571 | N[C@@H](CSCC(=O)N1CC([N+](=O)[O-])C1)C(=O)O | alpha | -1.381 | 263.275 | FALSE | TRUE |
| CHEMBL3527517 | COc1cc2nccc(N[C@@H](CS)C(=O)O)c2cc1C(N)=O | alpha | 1.1372 | 321.358 | TRUE | TRUE |
| CHEMBL35289 | Nc1ccc(CON[C@@H](CCC(=O)O)C(=O)O)cc1 | alpha | 0.608 | 268.269 | TRUE | TRUE |
| CHEMBL353121 | N[C@@H](C[C@H](CC1CCC(c2ccccc2)CC1)C(=O)O)C(=O)O | alpha | 2.8533 | 319.401 | TRUE | TRUE |
| CHEMBL353124 | N[C@@H](C[C@H](CCCCc1ccccc1)C(=O)O)C(=O)O | alpha | 1.9022 | 279.336 | TRUE | TRUE |
| CHEMBL353261 | NC(Cc1c(O)noc1-c1ccccc1)C(=O)O | alpha | 1.0016 | 248.238 | TRUE | FALSE |
| CHEMBL353345 | O=C(O)[C@H]1NC[C@@H]2O[C@@H]21 | alpha | -1.1898 | 129.115 | FALSE | TRUE |
| CHEMBL353794 | N=C(N)N(CCC[C@H](N)C(=O)O)[N+](=O)[O-] | alpha | -1.43433 | 219.201 | FALSE | TRUE |
| CHEMBL354235 | O=C(O)CC1=C(c2ccco2)CN[C@@H]1C(=O)O | alpha | 0.5643 | 237.211 | TRUE | TRUE |
| CHEMBL3544511 | N[C@@H](Cc1ccc(N(CCO)CCCl)cc1)C(=O)O | alpha | 0.6785 | 286.759 | TRUE | TRUE |
| CHEMBL3544512 | N[C@@H](Cc1ccc(N(CCO)CCO)cc1)C(=O)O | alpha | -0.568 | 268.313 | TRUE | TRUE |
| CHEMBL3544531 | CC(C)(SSC[C@H](N)C(=O)O)[C@@H](N)C(=O)O | alpha | -0.0298 | 268.36 | FALSE | TRUE |
| CHEMBL3544532 | CC(C)(SSC(C)(C)[C@@H](N)C(=O)O)[C@H](N)C(=O)O | alpha | 0.7488 | 296.414 | FALSE | TRUE |
| CHEMBL3544554 | CSC(C)(C)[C@@H](N)C(=O)O | alpha | 0.5399 | 163.242 | FALSE | TRUE |
| CHEMBL3544680 | CC1(C)S[C@H](CNC(=O)Cc2ccccc2)N[C@H]1C(=O)O | alpha | 1.2396 | 308.403 | TRUE | TRUE |
| CHEMBL3545351 | Nc1cc(Cl)ccc1C(=O)CC(N)C(=O)O | alpha | 0.9069 | 242.662 | TRUE | FALSE |
| CHEMBL3545929 | NC(C(=O)O)c1ccc(-n2cccn2)cc1 | alpha | 0.9567 | 217.228 | TRUE | FALSE |
| CHEMBL3546423 | N[C@@H](C(=O)O)[C@H]1CCCN1 | alpha | -0.8497 | 144.174 | FALSE | TRUE |
| CHEMBL35466 | N=C(N)Nc1cccc(C(=O)Nc2ccc(CC(N)C(=O)O)cc2)c1 | alpha | 1.19867 | 341.371 | TRUE | FALSE |
| CHEMBL3546750 | N[C@H](C(=O)O)[C@H]1CCCN1 | alpha | -0.8497 | 144.174 | FALSE | TRUE |
| CHEMBL354680 | O=C(O)[C@@H](CS)NCc1ccccc1 | alpha | 1.1592 | 211.286 | TRUE | TRUE |
| CHEMBL3547033 | Cc1ccc(O)cc1C[C@H](N)C(=O)O | alpha | 0.65502 | 195.218 | TRUE | TRUE |
| CHEMBL3547132 | N[C@@H](Cc1cc(O)c(O)cc1F)C(=O)O | alpha | 0.1913 | 215.18 | TRUE | TRUE |
| CHEMBL355615 | O=C(O)[C@@H]1C[C@H](CCP(=O)(O)O)CCN1 | alpha | 0.007 | 237.192 | FALSE | TRUE |
| CHEMBL355775 | CC(C)NC(CS)C(=O)O | alpha | 0.3674 | 163.242 | FALSE | FALSE |
| CHEMBL3559410 | CC[C@@H](CC(=O)O)[C@H](N)C(=O)O | alpha | -0.1008 | 175.184 | FALSE | TRUE |
| CHEMBL3559411 | CCC[C@@H](CC(=O)O)[C@H](N)C(=O)O | alpha | 0.2893 | 189.211 | FALSE | TRUE |
| CHEMBL3559412 | CC(C)C[C@@H](CC(=O)O)[C@H](N)C(=O)O | alpha | 0.5353 | 203.238 | FALSE | TRUE |
| CHEMBL3559413 | N[C@H](C(=O)O)[C@H](CC(=O)O)c1ccccc1 | alpha | 0.6568 | 223.228 | TRUE | TRUE |
| CHEMBL35635 | O=C(O)C(NS(=O)(=O)c1ccc(Cl)cc1)c1ccccc1 | alpha | 2.4442 | 325.773 | TRUE | FALSE |
| CHEMBL356537 | N[C@H](C[C@H](Cc1csc2ccccc12)C(=O)O)C(=O)O | alpha | 1.9466 | 293.344 | TRUE | TRUE |
| CHEMBL356601 | C/C(=C\c1cccc([N+](=O)[O-])c1)C1CN[C@H](C(=O)O)C1CC(=O)O | alpha | 1.7616 | 334.328 | TRUE | TRUE |
| CHEMBL356771 | CC1N[C@H](C(=O)O)CS1 | alpha | 0.122 | 147.199 | FALSE | TRUE |
| CHEMBL356867 | NC(CNC(=O)CBr)C(=O)O | alpha | -1.0906 | 225.042 | FALSE | FALSE |
| CHEMBL356989 | O=C(O)C1CN(CCCCc2nnn[nH]2)CCN1 | alpha | -1.1192 | 254.294 | TRUE | FALSE |
| CHEMBL356999 | CCn1oc(C(=O)O)c(CC(N)C(=O)O)c1=O | alpha | -0.8862 | 244.203 | TRUE | FALSE |
| CHEMBL357043 | N[C@@H](C(=O)O)[C@@H]1[C@H](O)[C@H]1C(=O)O | alpha | -1.9102 | 175.14 | FALSE | TRUE |
| CHEMBL357135 | CC(C)(C)c1ccc(C[C@@H](C[C@@H](N)C(=O)O)C(=O)O)cc1 | alpha | 2.0294 | 293.363 | TRUE | TRUE |
| CHEMBL357463 | NC(CCCCCc1nnn[nH]1)C(=O)O | alpha | -0.2855 | 213.241 | TRUE | FALSE |
| CHEMBL357500 | CCCCCCCCCCCCCC1NC(C(=O)O)CS1 | alpha | 4.8032 | 315.523 | FALSE | FALSE |
| CHEMBL3576926 | N[C@@H](CCC(=O)Nc1ccccc1Cc1ccccc1)C(=O)O | alpha | 2.408 | 312.369 | TRUE | TRUE |
| CHEMBL3576927 | N[C@@H](CCC(=O)Nc1ccccc1N1CCOCC1)C(=O)O | alpha | 0.6538 | 307.35 | TRUE | TRUE |
| CHEMBL3576929 | N[C@@H](CCC(=O)Nc1ccccc1CN1CCOCC1)C(=O)O | alpha | 0.6494 | 321.377 | TRUE | TRUE |
| CHEMBL3576930 | N[C@@H](CCC(=O)Nc1ccccc1CN1CCCC1)C(=O)O | alpha | 1.413 | 305.378 | TRUE | TRUE |
| CHEMBL3576931 | N[C@@H](CCC(=O)Nc1ccccc1N1CCNCC1)C(=O)O | alpha | 0.2268 | 306.366 | TRUE | TRUE |
| CHEMBL3576932 | N[C@@H](CCC(=O)Nc1ccccc1-c1ccccc1)C(=O)O | alpha | 2.4842 | 298.342 | TRUE | TRUE |
| CHEMBL3576933 | N[C@@H](CCC(=O)Nc1cccc(N2CCOCC2)c1)C(=O)O | alpha | 0.6538 | 307.35 | TRUE | TRUE |
| CHEMBL3576934 | CN1CCN(c2ccccc2NC(=O)CC[C@H](N)C(=O)O)CC1 | alpha | 0.569 | 320.393 | TRUE | TRUE |
| CHEMBL3576935 | N[C@@H](CCC(=O)Nc1ccc(N2CCOCC2)cc1)C(=O)O | alpha | 0.6538 | 307.35 | TRUE | TRUE |
| CHEMBL3576936 | N[C@@H](CCC(=O)Nc1ccccc1N1CCCCC1)C(=O)O | alpha | 1.8075 | 305.378 | TRUE | TRUE |
| CHEMBL3576937 | N[C@@H](CCC(=O)Nc1ccccc1-n1cccc1)C(=O)O | alpha | 1.6079 | 287.319 | TRUE | TRUE |
| CHEMBL3576939 | CN1CCN(c2ccccc2CNC(=O)CC[C@H](N)C(=O)O)CC1 | alpha | 0.2467 | 334.42 | TRUE | TRUE |
| CHEMBL3576940 | N[C@@H](CCC(=O)Nc1ccccc1CCc1ccncc1)C(=O)O | alpha | 1.9974 | 327.384 | TRUE | TRUE |
| CHEMBL3576941 | N[C@@H](CCC(=O)NCc1ccccc1CN1CCOCC1)C(=O)O | alpha | 0.3271 | 335.404 | TRUE | TRUE |
| CHEMBL3576942 | N[C@@H](CCC(=O)NCc1ccccc1N1CCOCC1)C(=O)O | alpha | 0.3315 | 321.377 | TRUE | TRUE |
| CHEMBL3576944 | CN1CCN(Cc2ccccc2CNC(=O)CC[C@H](N)C(=O)O)CC1 | alpha | 0.2423 | 348.447 | TRUE | TRUE |
| CHEMBL3577706 | NC(Cc1cn(CCF)c2ccccc12)C(=O)O | alpha | 1.5652 | 250.273 | TRUE | FALSE |
| CHEMBL3577709 | CN(C=O)c1ccccc1C(=O)C[C@H](N)C(=O)O | alpha | 0.2639 | 250.254 | TRUE | TRUE |
| CHEMBL3577710 | NC(CC(=O)c1ccccc1N(C=O)CCF)C(=O)O | alpha | 0.6036 | 282.271 | TRUE | FALSE |
| CHEMBL3577711 | CNc1ccccc1C(=O)C[C@H](N)C(=O)O | alpha | 0.713 | 222.244 | TRUE | TRUE |
| CHEMBL3577712 | Nc1ccc(O)cc1C(=O)C[C@H](N)C(=O)O | alpha | -0.0409 | 224.216 | TRUE | TRUE |
| CHEMBL3577713 | NC(CC(=O)c1ccccc1NCCF)C(=O)O | alpha | 1.0527 | 254.261 | TRUE | FALSE |
| CHEMBL3577861 | CC(C)C[C@@H](Nc1ncc2ccc(=O)n(C(C)C)c2n1)C(=O)O | alpha | 2.2836 | 318.377 | TRUE | TRUE |
| CHEMBL357939 | C/C(=C/c1cccc([N+](=O)[O-])c1)C1CN[C@H](C(=O)O)C1CC(=O)O | alpha | 1.7616 | 334.328 | TRUE | TRUE |
| CHEMBL358019 | Cc1snc(O)c1CC(N)C(=O)O | alpha | 0.11152 | 202.235 | TRUE | FALSE |
| CHEMBL358347 | N[C@@H](Cc1c[nH]c2c(F)cc(F)cc12)C(=O)O | alpha | 1.4005 | 240.209 | TRUE | TRUE |
| CHEMBL358628 | O=C(O)[C@@H]1CCSCN1 | alpha | 0.1236 | 147.199 | FALSE | TRUE |
| CHEMBL35957 | N[C@H](CC(=O)c1cccc([N+](=O)[O-])c1)C(=O)O | alpha | 0.5795 | 238.199 | TRUE | TRUE |
| CHEMBL3617722 | CC[C@H](NS(=O)(=O)c1ccc([N+](=O)[O-])cc1)C(=O)O | alpha | 0.7363 | 288.281 | TRUE | TRUE |
| CHEMBL362277 | O=C(O)[C@@H]1C[C@H](CCCc2nn[nH]n2)CCN1 | alpha | -0.0248 | 239.279 | TRUE | TRUE |
| CHEMBL3628563 | C[C@H](NC1=CC(=O)C=C(C(C)(C)C)C1=O)C(=O)O | alpha | 1.0573 | 251.282 | FALSE | TRUE |
| CHEMBL3628565 | CC(C)[C@@H](NC1=CC(=O)C=C(C(C)(C)C)C1=O)C(=O)O | alpha | 1.6934 | 279.336 | FALSE | TRUE |
| CHEMBL3628566 | CC(C)C[C@H](NC1=CC(=O)C=C(C(C)(C)C)C1=O)C(=O)O | alpha | 2.0835 | 293.363 | FALSE | TRUE |
| CHEMBL3628568 | CSCC[C@H](NC1=CC(=O)C=C(C(C)(C)C)C1=O)C(=O)O | alpha | 1.7905 | 311.403 | FALSE | TRUE |
| CHEMBL3628569 | CC(C)(C)C1=CC(=O)C=C(N[C@@H](Cc2ccccc2)C(=O)O)C1=O | alpha | 2.2801 | 327.38 | TRUE | TRUE |
| CHEMBL3629572 | CCS(=O)(=O)c1ccc(C(=O)C[C@H](N)C(=O)O)cc1 | alpha | 0.4649 | 285.321 | TRUE | TRUE |
| CHEMBL362993 | CC(=O)N[C@@H](CC(C)C)[C@@H]1N[C@@H](C(=O)O)C[C@H]1c1cc[nH]n1 | alpha | 0.8592 | 308.382 | TRUE | TRUE |
| CHEMBL363422 | CC(C)n1nnnc1-c1onc(O)c1CC(N)C(=O)O | alpha | -0.431 | 282.26 | TRUE | FALSE |
| CHEMBL363635 | CC(=O)N[C@@H](CC(C)C)[C@@H]1N[C@@H](C(=O)O)C[C@H]1N | alpha | -0.3204 | 257.334 | FALSE | TRUE |
| CHEMBL363649 | O=C(O)[C@H]1NCCN(C(=O)/C=C/c2ccccc2)[C@H]1C(=O)O | alpha | 0.038 | 304.302 | TRUE | TRUE |
| CHEMBL363702 | NCCCC[C@H](N)C(=O)ON[C@H](Cc1c[nH]c2ccccc12)C(=O)O | alpha | 0.6677 | 348.403 | TRUE | TRUE |
| CHEMBL364535 | NCCCC[C@H](N)C(=O)ON[C@@H](Cc1c[nH]c2ccccc12)C(=O)O | alpha | 0.6677 | 348.403 | TRUE | TRUE |
| CHEMBL364952 | C/N=C(\N)NCC[C@H](N)C(=O)O | alpha | -1.6775 | 174.204 | FALSE | TRUE |
| CHEMBL365976 | CC(=O)N[C@@H](CC(C)C)[C@@H]1N[C@@H](C(=O)O)C[C@H]1c1ccon1 | alpha | 1.1241 | 309.366 | TRUE | TRUE |
| CHEMBL366026 | C=C[C@@H]1C[C@H](C(=O)O)N[C@H]1[C@H](CC(C)C)NC(C)=O | alpha | 1.1545 | 268.357 | FALSE | TRUE |
| CHEMBL3665072 | O=C(O)c1ccc(Br)c(C2NC(C(=O)O)CS2)c1O | alpha | 1.6411 | 348.174 | TRUE | FALSE |
| CHEMBL366555 | CC(C)N[C@@H](CS)C(=O)O | alpha | 0.3674 | 163.242 | FALSE | TRUE |
| CHEMBL366591 | O=C(O)[C@H]1C[C@H](Cc2nn[nH]n2)CCN1 | alpha | -0.805 | 211.225 | TRUE | TRUE |
| CHEMBL367027 | N[C@@H](Cc1onc(O)c1-c1ccc(O)cc1)C(=O)O | alpha | 0.7072 | 264.237 | TRUE | TRUE |
| CHEMBL367030 | O=C(O)[C@@H]1C[C@@H](c2nnn[nH]2)CN1 | alpha | -1.2702 | 183.171 | TRUE | TRUE |
| CHEMBL36709 | COP(=O)(N[C@@H](CCC(=O)O)C(=O)O)c1ccccc1 | alpha | 1.059 | 301.235 | TRUE | TRUE |
| CHEMBL367185 | O=C(O)C(CS)NCc1ccc2ccccc2c1 | alpha | 2.3124 | 261.346 | TRUE | FALSE |
| CHEMBL367189 | N[C@@H](Cc1onc(O)c1CCc1ccccc1)C(=O)O | alpha | 1.1198 | 276.292 | TRUE | TRUE |
| CHEMBL367332 | O=C(O)C(CS)NCCc1ccccc1 | alpha | 1.2017 | 225.313 | TRUE | FALSE |
| CHEMBL36766 | CC([C@@H]1CCN[C@H](C(=O)O)C1)P(=O)(O)O | alpha | 0.0054 | 237.192 | FALSE | TRUE |
| CHEMBL36825 | NC(Cc1nc2c(Cl)cccc2n1CP(=O)(O)O)C(=O)O | alpha | 0.7793 | 333.668 | TRUE | FALSE |
| CHEMBL368556 | N[C@@H](Cc1onc(O)c1-c1cccc(O)c1)C(=O)O | alpha | 0.7072 | 264.237 | TRUE | TRUE |
| CHEMBL368858 | N[C@@H](Cc1onc(O)c1-c1ccccc1O)C(=O)O | alpha | 0.7072 | 264.237 | TRUE | TRUE |
| CHEMBL369349 | N=C(N)CCCCC(N)C(=O)O | alpha | -0.10533 | 173.216 | FALSE | FALSE |
| CHEMBL370047 | C/C=C\[C@@H]1C[C@H](C(=O)O)N[C@H]1[C@H](CC(C)C)NC(C)=O | alpha | 1.5446 | 282.384 | FALSE | TRUE |
| CHEMBL370170 | C/C(N)=N\CC[C@@H](C)[C@H](N)C(=O)O | alpha | -0.1984 | 187.243 | FALSE | TRUE |
| CHEMBL370793 | N[C@H](Cn1ccc(=O)n(CC(=O)O)c1=O)C(=O)O | alpha | -2.4935 | 257.202 | TRUE | TRUE |
| CHEMBL370941 | CCn1nnc(-c2onc(O)c2CC(N)C(=O)O)n1 | alpha | -0.992 | 268.233 | TRUE | FALSE |
| CHEMBL371301 | O=C(O)[C@H]1CC[C@H]2CN[C@H](C(=O)O)[C@H]21 | alpha | -0.2302 | 199.206 | FALSE | TRUE |
| CHEMBL37140 | O=C(O)Cc1cc(Cl)c2c(c1)CCC(C(=O)O)N2 | alpha | 1.7784 | 269.684 | TRUE | FALSE |
| CHEMBL371784 | CCCn1nnnc1-c1onc(O)c1CC(N)C(=O)O | alpha | -0.6019 | 282.26 | TRUE | FALSE |
| CHEMBL371944 | N[C@@H](Cn1ccc(=O)n(Cc2cccc(C(=O)O)c2)c1=O)C(=O)O | alpha | -0.8316 | 333.3 | TRUE | TRUE |
| CHEMBL372124 | CC[C@H](C[C@H](N)C(=O)O)C(=O)O | alpha | -0.1008 | 175.184 | FALSE | TRUE |
| CHEMBL372643 | CCCn1nnc(-c2onc(O)c2CC(N)C(=O)O)n1 | alpha | -0.6019 | 282.26 | TRUE | FALSE |
| CHEMBL373336 | CC(=O)N[C@@H](CC(C)C)[C@@H]1N[C@@H](C(=O)O)C[C@H]1c1nccs1 | alpha | 1.5926 | 325.434 | TRUE | TRUE |
| CHEMBL373568 | CCCCCCCCCCCCCC1N[C@H](C(=O)O)CS1 | alpha | 4.8032 | 315.523 | FALSE | TRUE |
| CHEMBL3740036 | N[C@@H](CCC(=O)N1C[C@@H]2ON=C(O)[C@@H]2C1)C(=O)O | alpha | -1.0928 | 257.246 | FALSE | TRUE |
| CHEMBL3740477 | N[C@H](CCC(=O)N1C[C@@H]2ON=C(C(=O)O)[C@@H]2C1)C(=O)O | alpha | -1.5237 | 285.256 | FALSE | TRUE |
| CHEMBL3740786 | N[C@@H](CCC(=O)N1C[C@@H]2C(C(=O)O)=NO[C@@H]2C1)C(=O)O | alpha | -1.5237 | 285.256 | FALSE | TRUE |
| CHEMBL3740885 | N[C@H](CCC(=O)N1C[C@@H]2ON=C(O)[C@@H]2C1)C(=O)O | alpha | -1.0928 | 257.246 | FALSE | TRUE |
| CHEMBL3741345 | N[C@H](CCC(=O)N1C[C@@H]2C(C(=O)O)=NO[C@@H]2C1)C(=O)O | alpha | -1.5237 | 285.256 | FALSE | TRUE |
| CHEMBL3741912 | N[C@@H](CCC(=O)N1C[C@@H]2ON=C(C(=O)O)[C@@H]2C1)C(=O)O | alpha | -1.5237 | 285.256 | FALSE | TRUE |
| CHEMBL3741936 | N[C@H](CCC(=O)N1C[C@@H]2C(O)=NO[C@@H]2C1)C(=O)O | alpha | -1.0928 | 257.246 | FALSE | TRUE |
| CHEMBL3741954 | N[C@@H](CCC(=O)N1C[C@@H]2C(O)=NO[C@@H]2C1)C(=O)O | alpha | -1.0928 | 257.246 | FALSE | TRUE |
| CHEMBL3742299 | Cl.Cn1nnc(O)c1CC(N)C(=O)O | alpha | -1.1031 | 222.632 | TRUE | FALSE |
| CHEMBL3742451 | Cn1nnc(O)c1CC(N)C(=O)O | alpha | -1.5249 | 186.171 | TRUE | FALSE |
| CHEMBL3753768 | Cc1nc(CSCC(N)C(=O)O)c(C)nc1CO | alpha | 0.23084 | 271.342 | TRUE | FALSE |
| CHEMBL3758318 | NC(Cc1cc(Cl)c(Cl)c(-c2ccc(O)nc2)c1)C(=O)O | alpha | 2.7154 | 327.167 | TRUE | FALSE |
| CHEMBL3759138 | Nc1cccc(-c2cc(CC(N)C(=O)O)cc(Cl)c2Cl)c1 | alpha | 3.197 | 325.195 | TRUE | FALSE |
| CHEMBL3759334 | NC(Cc1cc(Cl)c(Cl)c(-c2cc(O)cc(O)c2)c1)C(=O)O | alpha | 3.026 | 342.178 | TRUE | FALSE |
| CHEMBL3759390 | NC(Cc1cc(Cl)c(Cl)c(-c2cccc(CO)c2)c1)C(=O)O | alpha | 3.1071 | 340.206 | TRUE | FALSE |
| CHEMBL3759469 | NC(Cc1cc(Cl)c(Cl)c(-c2ccc(O)c(O)c2)c1)C(=O)O | alpha | 3.026 | 342.178 | TRUE | FALSE |
| CHEMBL3759527 | COc1cccc(-c2cc(CC(N)C(=O)O)cc(Cl)c2Cl)c1 | alpha | 3.6234 | 340.206 | TRUE | FALSE |
| CHEMBL3759809 | NC(Cc1cc(Cl)c(Cl)c(-c2ccc(CO)cc2)c1)C(=O)O | alpha | 3.1071 | 340.206 | TRUE | FALSE |
| CHEMBL3763577 | N[C@@H](CCC(=O)NCCS)C(=O)O | alpha | -0.7755 | 206.267 | FALSE | TRUE |
| CHEMBL3770255 | Nc1ccccc1C1=CNC(C(=O)O)C1 | alpha | 1.0562 | 204.229 | TRUE | FALSE |
| CHEMBL377079 | NC(CCSCc1ccc(Cl)cc1)C(=O)O | alpha | 2.3752 | 259.758 | TRUE | FALSE |
| CHEMBL377442 | NC(CCSc1ccc(C(=O)O)cc1)C(=O)O | alpha | 1.2789 | 255.295 | TRUE | FALSE |
| CHEMBL3780051 | O=C(O)[C@@H]1CSC(c2cccc(Cl)c2)N1 | alpha | 2.1282 | 243.715 | TRUE | TRUE |
| CHEMBL3780297 | O=C(O)[C@@H]1CSC(c2ccc(Cl)cc2)N1 | alpha | 2.1282 | 243.715 | TRUE | TRUE |
| CHEMBL3780336 | N[C@@H](CC[C@]1(O)CNC1=O)C(=O)O | alpha | -1.9606 | 188.183 | FALSE | TRUE |
| CHEMBL3780565 | Cc1ccc(C2N[C@H](C(=O)O)CS2)cc1 | alpha | 1.78322 | 223.297 | TRUE | TRUE |
| CHEMBL3780917 | O=C(O)[C@@H]1CSC(c2cccc(F)c2F)N1 | alpha | 1.753 | 245.25 | TRUE | TRUE |
| CHEMBL3781315 | Cc1ccccc1C1N[C@H](C(=O)O)CS1 | alpha | 1.78322 | 223.297 | TRUE | TRUE |
| CHEMBL3781515 | N#Cc1ccccc1C1N[C@H](C(=O)O)CS1 | alpha | 1.34648 | 234.28 | TRUE | TRUE |
| CHEMBL3781641 | O=C(O)[C@@H]1CSC(c2c(F)cccc2F)N1 | alpha | 1.753 | 245.25 | TRUE | TRUE |
| CHEMBL378166 | N[C@H](COc1cncc(/C=C/c2ccncc2)c1)C(=O)O | alpha | 1.4377 | 285.303 | TRUE | TRUE |
| CHEMBL3781761 | O=C(O)[C@@H]1CSC(c2cccc(Cl)c2Cl)N1 | alpha | 2.7816 | 278.16 | TRUE | TRUE |
| CHEMBL3781802 | O=C(O)[C@@H]1CSC(c2ccccc2Br)N1 | alpha | 2.2373 | 288.166 | TRUE | TRUE |
| CHEMBL3781828 | O=C(O)[C@@H]1CSC(c2ccc([N+](=O)[O-])cc2)N1 | alpha | 1.383 | 254.267 | TRUE | TRUE |
| CHEMBL3781865 | COc1ccccc1C1N[C@H](C(=O)O)CS1 | alpha | 1.4834 | 239.296 | TRUE | TRUE |
| CHEMBL37852 | O=C(O)[C@H]1NCCC[C@H]1CCP(=O)(O)O | alpha | 0.007 | 237.192 | FALSE | TRUE |
| CHEMBL3785412 | O=C(O)[C@@H]1C[C@@H]2C[C@@H]2N1 | alpha | -0.1786 | 127.143 | FALSE | TRUE |
| CHEMBL3785525 | O=C(O)[C@H]1NCc2ccccc21 | alpha | 0.9155 | 163.176 | TRUE | TRUE |
| CHEMBL378582 | Cc1ccc([C@H](N)C(=O)O)cc1 | alpha | 1.07942 | 165.192 | TRUE | TRUE |
| CHEMBL3785905 | CC(=O)Nc1ccc(O)c(SC[C@H](N)C(=O)O)c1 | alpha | 0.8546 | 270.31 | TRUE | TRUE |
| CHEMBL3786320 | O=C(O)[C@@H]1C[S+]([O-])CN1 | alpha | -1.251 | 149.171 | FALSE | TRUE |
| CHEMBL3786983 | O=C(O)[C@H]1NC[C@@H]2C[C@@H]21 | alpha | -0.3211 | 127.143 | FALSE | TRUE |
| CHEMBL3787284 | O=C(O)[C@@H]1CS(=O)(=O)CN1 | alpha | -1.5849 | 165.17 | FALSE | TRUE |
| CHEMBL3787316 | O=C(O)[C@@H]1C[C@H]2C[C@H]2N1 | alpha | -0.1786 | 127.143 | FALSE | TRUE |
| CHEMBL37876 | C[C@@H]1CN[C@H](C(=O)O)C[C@@H]1CP(=O)(O)O | alpha | -0.1371 | 237.192 | FALSE | TRUE |
| CHEMBL379182 | N[C@@H](CCCCNC(=O)CCS)C(=O)O | alpha | 0.0047 | 234.321 | FALSE | TRUE |
| CHEMBL379442 | N[C@@H](Cn1ccc(=O)n(Cc2ccsc2C(=O)O)c1=O)C(=O)O | alpha | -0.7701 | 339.329 | TRUE | TRUE |
| CHEMBL37949 | N[C@H](Cc1nc2cc(Cl)ccc2n1CP(=O)(O)O)C(=O)O | alpha | 0.7793 | 333.668 | TRUE | TRUE |
| CHEMBL3798541 | N/C(=N\C(=O)c1ccc(C[C@H](N)C(=O)O)cc1)NCc1ccccc1 | alpha | 0.8857 | 340.383 | TRUE | TRUE |
| CHEMBL3798809 | NC(N)=NC(=O)c1ccc(C[C@H](N)C(=O)O)cc1 | alpha | -0.9454 | 250.258 | TRUE | TRUE |
| CHEMBL3804963 | C[C@H](Nc1nc(N)nc2[nH]cnc12)C(=O)O | alpha | -0.1799 | 222.208 | TRUE | TRUE |
| CHEMBL380506 | NC(CCSCc1ccccc1[N+](=O)[O-])C(=O)O | alpha | 1.63 | 270.31 | TRUE | FALSE |
| CHEMBL380507 | N#Cc1cccc(CSCCC(N)C(=O)O)c1 | alpha | 1.59348 | 250.323 | TRUE | FALSE |
| CHEMBL3805293 | CC(C)[C@H](Nc1nc(N)nc2[nH]cnc12)C(=O)O | alpha | 0.4562 | 250.262 | TRUE | TRUE |
| CHEMBL380670 | NC(CCSCc1cccc(C(F)(F)F)c1)C(=O)O | alpha | 2.7406 | 293.31 | TRUE | FALSE |
| CHEMBL380671 | NC(CCCC1CCCCC1)C(=O)O | alpha | 2.1489 | 199.294 | FALSE | FALSE |
| CHEMBL3808694 | CC(C)c1cc(C[C@H](N)C(=O)O)ccc1O | alpha | 1.47 | 223.272 | TRUE | TRUE |
| CHEMBL3808862 | CCc1cccc(C[C@H](N)C(=O)O)c1 | alpha | 1.2034 | 193.246 | TRUE | TRUE |
| CHEMBL38090 | C[C@@H]1[C@H](CP(=O)(O)O)CCN[C@@H]1C(=O)O | alpha | -0.1371 | 237.192 | FALSE | TRUE |
| CHEMBL3809069 | CC(Nc1nnc2nncn2n1)C(=O)O | alpha | -1.2007 | 209.169 | TRUE | FALSE |
| CHEMBL380911 | N[C@@H](CCSCc1ccccc1)C(=O)O | alpha | 1.7218 | 225.313 | TRUE | TRUE |
| CHEMBL3809112 | N[C@@H](Cc1cccc(Cc2ccccc2)c1)C(=O)O | alpha | 2.2318 | 255.317 | TRUE | TRUE |
| CHEMBL3809257 | CCc1cc(C[C@H](N)C(=O)O)ccc1O | alpha | 0.909 | 209.245 | TRUE | TRUE |
| CHEMBL380929 | NC(CCSCc1cccc(Br)c1)C(=O)O | alpha | 2.4843 | 304.209 | TRUE | FALSE |
| CHEMBL380950 | Cc1cc(C)cc(CSCCC(N)C(=O)O)c1 | alpha | 2.33864 | 253.367 | TRUE | FALSE |
| CHEMBL3809645 | N[C@@H](Cc1ccc(O)c(Cc2ccccc2)c1)C(=O)O | alpha | 1.9374 | 271.316 | TRUE | TRUE |
| CHEMBL3809837 | CC(C)c1cccc(C[C@H](N)C(=O)O)c1 | alpha | 1.7644 | 207.273 | TRUE | TRUE |
| CHEMBL3809882 | N[C@@H](Cc1ccc(O)c(-c2ccccc2)c1)C(=O)O | alpha | 2.0136 | 257.289 | TRUE | TRUE |
| CHEMBL3810016 | COc1ccc(C[C@H](N)C(=O)O)cc1C | alpha | 0.95802 | 209.245 | TRUE | TRUE |
| CHEMBL381004 | CCCC[C@H](C[C@H](N)C(=O)O)C(=O)O | alpha | 0.6794 | 203.238 | FALSE | TRUE |
| CHEMBL3810204 | CC(C)(C)c1cccc(C[C@H](N)C(=O)O)c1 | alpha | 1.9385 | 221.3 | TRUE | TRUE |
| CHEMBL38117 | C[C@H]1C[C@@H](CP(=O)(O)O)C[C@@H](C(=O)O)N1 | alpha | 0.0054 | 237.192 | FALSE | TRUE |
| CHEMBL381281 | NC(CCSCc1ccc(Cl)c(Cl)c1Cl)C(=O)O | alpha | 3.682 | 328.648 | TRUE | FALSE |
| CHEMBL381383 | NC(CCSCc1ccccc1C(F)(F)F)C(=O)O | alpha | 2.7406 | 293.31 | TRUE | FALSE |
| CHEMBL382117 | CC(C)[C@@H](NS(=O)(=O)c1ccc(-c2ccccc2)cc1)C(=O)O | alpha | 2.7411 | 333.409 | TRUE | TRUE |
| CHEMBL382322 | CC(=O)N[C@@H](CC(C)C)[C@@H]1N[C@@H](C(=O)O)C[C@H]1C=C(F)F | alpha | 1.7489 | 304.337 | FALSE | TRUE |
| CHEMBL382371 | NC(C(=O)O)c1cccc(Br)c1 | alpha | 1.5335 | 230.061 | TRUE | FALSE |
| CHEMBL382570 | NC(C(=O)O)c1cccs1 | alpha | 0.8325 | 157.194 | TRUE | FALSE |
| CHEMBL382621 | NC(C(=O)O)c1ccccc1C(F)(F)F | alpha | 1.7898 | 219.162 | TRUE | FALSE |
| CHEMBL382622 | NC(C(=O)O)c1ccc(F)cc1F | alpha | 1.0492 | 187.145 | TRUE | FALSE |
| CHEMBL382682 | NC(CCSCc1ccc(Cl)c(Cl)c1)C(=O)O | alpha | 3.0286 | 294.203 | TRUE | FALSE |
| CHEMBL38274 | O=C(O)[C@H](Cc1ccccc1)NCP(=O)(O)O | alpha | 0.4071 | 259.198 | TRUE | TRUE |
| CHEMBL38308 | NC(Cc1nc2cccc(Cl)c2n1CP(=O)(O)O)C(=O)O | alpha | 0.7793 | 333.668 | TRUE | FALSE |
| CHEMBL38325 | O=C(O)C(Cc1cccc(-c2ccccc2)c1)NCP(=O)(O)O | alpha | 2.0741 | 335.296 | TRUE | FALSE |
| CHEMBL383441 | CC(C)(C)c1ccc(CSCCC(N)C(=O)O)cc1 | alpha | 3.0193 | 281.421 | TRUE | FALSE |
| CHEMBL38365 | O=C(O)[C@H](Cc1ccc(-c2ccccc2)cc1)NCCP(=O)(O)O | alpha | 2.1166 | 349.323 | TRUE | TRUE |
| CHEMBL383715 | NC(C(=O)O)c1ccccc1F | alpha | 0.9101 | 169.155 | TRUE | FALSE |
| CHEMBL38499 | C=C(C[C@H](N)C(=O)O)C(=O)O | alpha | -0.5708 | 159.141 | FALSE | TRUE |
| CHEMBL38817 | C=C(CC(N)C(=O)O)C(=O)O | alpha | -0.5708 | 159.141 | FALSE | FALSE |
| CHEMBL3883546 | O=C(O)C[C@H](NCCCCc1ccccc1)C(=O)O | alpha | 1.5268 | 265.309 | TRUE | TRUE |
| CHEMBL3884120 | O=C(O)C[C@H](NCCCc1ccccc1)C(=O)O | alpha | 1.1367 | 251.282 | TRUE | TRUE |
| CHEMBL3884832 | O=C(O)C[C@H](NCc1ccccc1)C(=O)O | alpha | 0.7041 | 223.228 | TRUE | TRUE |
| CHEMBL3885473 | O=C(O)C[C@H](NCCc1ccccc1)C(=O)O | alpha | 0.7466 | 237.255 | TRUE | TRUE |
| CHEMBL38924 | C=C(C[C@@H](N)C(=O)O)C(=O)O | alpha | -0.5708 | 159.141 | FALSE | TRUE |
| CHEMBL3894202 | NC(Cc1cc(Cl)cc(-c2cccc(C(=O)O)c2)c1)C(=O)O | alpha | 2.6596 | 319.744 | TRUE | FALSE |
| CHEMBL3894806 | N[C@@H](C[C@@H]1CC(c2ccc(C(=O)O)cc2)=NO1)C(=O)O | alpha | 0.6798 | 278.264 | TRUE | TRUE |
| CHEMBL3895296 | Nc1ccc(CC(N)C(=O)O)cc1-c1cccc(C(=O)O)c1 | alpha | 1.5884 | 300.314 | TRUE | FALSE |
| CHEMBL389555 | N[C@H](C(=O)O)[C@@H]1C[C@H]1S(=O)(=O)O | alpha | -1.3254 | 195.196 | FALSE | TRUE |
| CHEMBL389556 | N[C@@H](C(=O)O)[C@H]1C[C@@H]1S(=O)(=O)O | alpha | -1.3254 | 195.196 | FALSE | TRUE |
| CHEMBL389557 | N[C@H](C(=O)O)[C@H]1C[C@H]1S(=O)(=O)O | alpha | -1.3254 | 195.196 | FALSE | TRUE |
| CHEMBL389558 | N[C@H](C(=O)O)[C@@H]1C[C@@H]1S(=O)(=O)O | alpha | -1.3254 | 195.196 | FALSE | TRUE |
| CHEMBL389585 | CCCc1c(C(N)C(=O)O)cnn1O | alpha | 0.1573 | 199.21 | TRUE | FALSE |
| CHEMBL389833 | CCCCCc1c(C(N)C(=O)O)cnn1O | alpha | 0.9375 | 227.264 | TRUE | FALSE |
| CHEMBL3898911 | N[C@@H](C[C@@H]1CC(/C=C/C(=O)O)=NO1)C(=O)O | alpha | -0.4259 | 228.204 | FALSE | TRUE |
| CHEMBL3899726 | N[C@H](C(=O)O)C(NS(=O)(=O)c1cccs1)C(=O)O | alpha | -1.1085 | 294.31 | TRUE | TRUE |
| CHEMBL389980 | NC(C(=O)O)c1cnn(O)c1Br | alpha | -0.0327 | 236.025 | TRUE | FALSE |
| CHEMBL389999 | NC(C(=O)O)c1cnn(O)c1CC1CC1 | alpha | 0.1573 | 211.221 | TRUE | FALSE |
| CHEMBL390026 | NC(C(=O)O)c1cnn(O)c1-c1ccccc1 | alpha | 0.8718 | 233.227 | TRUE | FALSE |
| CHEMBL3901844 | N[C@H](C(=O)O)C(NS(=O)(=O)Cc1ccccc1)C(=O)O | alpha | -1.0289 | 302.308 | TRUE | TRUE |
| CHEMBL3903821 | CC(C)(C)c1ccc(S(=O)(=O)NC(C(=O)O)[C@H](N)C(=O)O)cc1 | alpha | 0.1275 | 344.389 | TRUE | TRUE |
| CHEMBL3904209 | NC(Cc1ccc(Cl)c(-c2cccc(C(=O)O)c2)c1)C(=O)O | alpha | 2.6596 | 319.744 | TRUE | FALSE |
| CHEMBL3907939 | N[C@@H](C[C@@H]1CC(CCC(=O)O)=NO1)C(=O)O | alpha | -0.2019 | 230.22 | FALSE | TRUE |
| CHEMBL3908316 | COc1cc(OC)c(CN[C@@H](CO)C(=O)O)c(OC)c1 | alpha | 0.2475 | 285.296 | TRUE | TRUE |
| CHEMBL390863 | CCc1c(C(N)C(=O)O)cnn1O | alpha | -0.2328 | 185.183 | TRUE | FALSE |
| CHEMBL3911432 | N[C@H](C(=O)O)[C@@H](NS(=O)(=O)c1ccccc1)C(=O)O | alpha | -1.17 | 288.281 | TRUE | TRUE |
| CHEMBL3912672 | NC(CCP(=O)(Oc1ccccc1)Oc1ccccc1)C(=O)O | alpha | 3.1395 | 335.296 | TRUE | FALSE |
| CHEMBL3913275 | N[C@H](C(=O)O)C(NC(=O)c1ccccc1)C(=O)O | alpha | -0.7185 | 252.226 | TRUE | TRUE |
| CHEMBL3914965 | CC(C)[C@H](NC1=CC(=O)c2ccccc2C1=O)C(=O)O | alpha | 1.6483 | 273.288 | TRUE | TRUE |
| CHEMBL3919821 | Cc1cc(C)cc(S(=O)(=O)NC(C(=O)O)[C@H](N)C(=O)O)c1 | alpha | -0.55316 | 316.335 | TRUE | TRUE |
| CHEMBL3920012 | N[C@H](C(=O)O)C(NS(=O)(=O)c1ccccc1Cl)C(=O)O | alpha | -0.5166 | 322.726 | TRUE | TRUE |
| CHEMBL3921460 | N[C@H](C(=O)O)C(C(=O)O)n1cc(-c2ccccc2)nn1 | alpha | -0.0173 | 276.252 | TRUE | TRUE |
| CHEMBL3922229 | Nc1c(Cl)cc(CC(N)C(=O)O)cc1-c1cccc(C(=O)O)c1 | alpha | 2.2418 | 334.759 | TRUE | FALSE |
| CHEMBL392419 | NC(C(=O)O)[C@@H]1[C@@H](c2ccccc2)[C@H]1P(=O)(O)O | alpha | 0.3582 | 271.209 | TRUE | TRUE |
| CHEMBL392420 | NC(C(=O)O)[C@H]1[C@H](c2ccccc2)[C@@H]1P(=O)(O)O | alpha | 0.3582 | 271.209 | TRUE | TRUE |
| CHEMBL3928498 | N[C@H](C(=O)O)C(NS(=O)(=O)c1ccc2ccccc2c1)C(=O)O | alpha | -0.0168 | 338.341 | TRUE | TRUE |
| CHEMBL3929003 | COc1cc(OC)c(CN[C@@H](Cc2ccccc2)C(=O)O)c(OC)c1 | alpha | 2.4979 | 345.395 | TRUE | TRUE |
| CHEMBL3931163 | COP(=O)(CCC(N)C(=O)O)Oc1cccc(CC(N)=O)c1 | alpha | 0.7347 | 330.277 | TRUE | FALSE |
| CHEMBL3931457 | N[C@@H](C[C@@H]1CC(c2ccccc2C(=O)O)=NO1)C(=O)O | alpha | 0.6798 | 278.264 | TRUE | TRUE |
| CHEMBL393395 | NCCN[C@@H](Cc1cn(CCc2ccccc2)cn1)C(=O)O | alpha | 0.6698 | 302.378 | TRUE | TRUE |
| CHEMBL3934366 | NC(Cc1cc(-c2cccc(C(=O)O)c2)cc([N+](=O)[O-])c1)C(=O)O | alpha | 1.9144 | 330.296 | TRUE | FALSE |
| CHEMBL3936563 | N[C@H](C(=O)O)C(NS(=O)(=O)c1cccnc1)C(=O)O | alpha | -1.775 | 289.269 | TRUE | TRUE |
| CHEMBL39372 | N[C@H](C(=O)O)c1ccc(C(=O)O)c(O)c1 | alpha | 0.1748 | 211.173 | TRUE | TRUE |
| CHEMBL3937517 | N[C@H](C(=O)O)[C@H](NS(=O)(=O)c1ccccc1)C(=O)O | alpha | -1.17 | 288.281 | TRUE | TRUE |
| CHEMBL3937980 | N[C@@H](C[C@H]1CC(c2ccc(C(=O)O)cc2)=NO1)C(=O)O | alpha | 0.6798 | 278.264 | TRUE | TRUE |
| CHEMBL3938810 | Cc1ccccc1S(=O)(=O)NC(C(=O)O)[C@H](N)C(=O)O | alpha | -0.86158 | 302.308 | TRUE | TRUE |
| CHEMBL3940746 | N[C@@H](C[C@H]1CC(CCC(=O)O)=NO1)C(=O)O | alpha | -0.2019 | 230.22 | FALSE | TRUE |
| CHEMBL3940791 | CCC(NC(CC(C)C)C1(c2ccc(Cl)cc2)CCC1)C(=O)O | alpha | 4.6292 | 337.891 | TRUE | FALSE |
| CHEMBL394108 | CCCCC1NC(C(=O)O)CS1 | alpha | 1.2923 | 189.28 | FALSE | FALSE |
| CHEMBL3941122 | N[C@@H](CCC(=O)Nc1cccc(S(=O)(=O)O)n1)C(=O)O | alpha | -0.5411 | 303.296 | TRUE | TRUE |
| CHEMBL394319 | COc1ccc(CC(N)C(=O)O)cc1CCC[18F] | alpha | 1.5517 | 254.291938 | TRUE | FALSE |
| CHEMBL394653 | O=C(O)C1CSC(c2ccc(Cl)c(Cl)c2)N1 | alpha | 2.7816 | 278.16 | TRUE | FALSE |
| CHEMBL3947294 | Cl.O=C(O)[C@@H]1C[C@H]2[C@@H](O)CCC[C@H]2N1 | alpha | 0.3843 | 221.684 | FALSE | TRUE |
| CHEMBL3947904 | N[C@@H](C[C@H]1CC(c2ccccc2C(=O)O)=NO1)C(=O)O | alpha | 0.6798 | 278.264 | TRUE | TRUE |
| CHEMBL3948303 | Nc1ccc(S(=O)(=O)NC(C(=O)O)[C@H](N)C(=O)O)cc1 | alpha | -1.5878 | 303.296 | TRUE | TRUE |
| CHEMBL394893 | O=C(O)C1CSC(c2c(F)cccc2Cl)N1 | alpha | 2.2673 | 261.705 | TRUE | FALSE |
| CHEMBL3950379 | N[C@H](C(=O)O)C(NS(=O)(=O)c1cccc(Cl)c1)C(=O)O | alpha | -0.5166 | 322.726 | TRUE | TRUE |
| CHEMBL39524 | NC(Cc1nc2ccc(Cl)cc2n1CP(=O)(O)O)C(=O)O | alpha | 0.7793 | 333.668 | TRUE | FALSE |
| CHEMBL39532 | NC(Cc1nc2ccccc2n1CP(=O)(O)O)C(=O)O | alpha | 0.1259 | 299.223 | TRUE | FALSE |
| CHEMBL3955047 | N[C@H](C(=O)O)C(NS(=O)(=O)c1ccccc1)C(=O)O | alpha | -1.17 | 288.281 | TRUE | TRUE |
| CHEMBL3955619 | O=C(O)[C@H](Cc1ccc(O)cc1)NS(=O)(=O)Cc1ccccc1 | alpha | 1.5075 | 335.381 | TRUE | TRUE |
| CHEMBL39573 | N[C@H](C(=O)O)[C@@H]1[C@@H](C(=O)O)[C@@H]1c1ccccc1 | alpha | 0.5127 | 235.239 | TRUE | TRUE |
| CHEMBL3958805 | COc1cc(OC)c(CN[C@@H](Cc2cnc[nH]2)C(=O)O)c(OC)c1 | alpha | 1.221 | 335.36 | TRUE | TRUE |
| CHEMBL395889 | NCCN[C@@H](Cc1cn(CC2CCC2)cn1)C(=O)O | alpha | 0.2272 | 266.345 | TRUE | TRUE |
| CHEMBL3960774 | N[C@H](C(=O)O)C(NS(=O)(=O)c1ccc(Cl)cc1)C(=O)O | alpha | -0.5166 | 322.726 | TRUE | TRUE |
| CHEMBL39613 | COc1cc2c(cc1O)C(C(=O)O)NCC2 | alpha | 0.6722 | 223.228 | TRUE | FALSE |
| CHEMBL396319 | NC(C(=O)O)[C@H]1[C@@H](c2ccccc2)[C@@H]1P(=O)(O)O | alpha | 0.3582 | 271.209 | TRUE | TRUE |
| CHEMBL396389 | NCCN[C@H](Cc1c[nH]cn1)C(=O)O | alpha | -1.0463 | 198.226 | TRUE | TRUE |
| CHEMBL3964129 | CN(C(C(=O)O)[C@H](N)C(=O)O)S(=O)(=O)c1ccccc1 | alpha | -0.8278 | 302.308 | TRUE | TRUE |
| CHEMBL3964385 | N[C@@H](C[C@@H]1CC(c2cccc(C(=O)O)c2)=NO1)C(=O)O | alpha | 0.6798 | 278.264 | TRUE | TRUE |
| CHEMBL39664 | O=C(O)[C@@H]1C[C@H](CP(=O)(O)O)CCN1 | alpha | -0.3831 | 223.165 | FALSE | TRUE |
| CHEMBL3966590 | NC(Cc1ccc([N+](=O)[O-])c(-c2cccc(C(=O)O)c2)c1)C(=O)O | alpha | 1.9144 | 330.296 | TRUE | FALSE |
| CHEMBL3967211 | N[C@H](C(=O)O)C(NS(=O)(=O)c1cccc2ccccc12)C(=O)O | alpha | -0.0168 | 338.341 | TRUE | TRUE |
| CHEMBL3967554 | Nc1c(-c2cccc(C(=O)O)c2)cc(CC(N)C(=O)O)cc1[N+](=O)[O-] | alpha | 1.4966 | 345.311 | TRUE | FALSE |
| CHEMBL396967 | O=C(O)C1CSC(c2cc(Br)ccc2O)N1 | alpha | 1.9429 | 304.165 | TRUE | FALSE |
| CHEMBL397014 | O=C(O)[C@@H]1C[C@]2(CN1)CC(O)=NO2 | alpha | -0.5365 | 186.167 | FALSE | TRUE |
| CHEMBL397015 | O=C(O)[C@H]1C[C@@]2(CN1)CC(O)=NO2 | alpha | -0.5365 | 186.167 | FALSE | TRUE |
| CHEMBL397016 | O=C(O)C1=NO[C@@]2(CN[C@H](C(=O)O)C2)C1 | alpha | -0.9674 | 214.177 | FALSE | TRUE |
| CHEMBL397048 | NC(C(=O)O)c1cnn(O)c1CCc1ccccc1 | alpha | 0.99 | 261.281 | TRUE | FALSE |
| CHEMBL397157 | CCCCCn1cnc(C[C@H](NCCN)C(=O)O)c1 | alpha | 0.6173 | 268.361 | TRUE | TRUE |
| CHEMBL3971971 | COP(=O)(CCC(N)C(=O)O)Oc1ccccc1 | alpha | 1.7069 | 273.225 | TRUE | FALSE |
| CHEMBL3973038 | N[C@@H](C[C@H]1CC(c2cccc(C(=O)O)c2)=NO1)C(=O)O | alpha | 0.6798 | 278.264 | TRUE | TRUE |
| CHEMBL3974720 | Cc1ccc(S(=O)(=O)NC(C(=O)O)[C@H](N)C(=O)O)cc1 | alpha | -0.86158 | 302.308 | TRUE | TRUE |
| CHEMBL397625 | CC(C)n1cnc(C[C@H](NCCN)C(=O)O)c1 | alpha | 0.008 | 240.307 | TRUE | TRUE |
| CHEMBL3978429 | N[C@@H](C[C@H]1CC(/C=C/C(=O)O)=NO1)C(=O)O | alpha | -0.4259 | 228.204 | FALSE | TRUE |
| CHEMBL3978735 | COc1cc(OC)c(CN[C@@H](C)C(=O)O)c(OC)c1 | alpha | 1.2751 | 269.297 | TRUE | TRUE |
| CHEMBL3978873 | N[C@@H](CSC(=O)Nc1cccc(S(=O)(=O)O)c1)C(=O)O | alpha | 0.6103 | 320.348 | TRUE | TRUE |
| CHEMBL39812 | O=C(O)[C@H](CC#Cc1ccc(F)cc1F)NCP(=O)(O)O | alpha | 0.8844 | 319.2 | TRUE | TRUE |
| CHEMBL3983064 | NC(Cc1cc(Cl)c(O)c(-c2cccc(C(=O)O)c2)c1)C(=O)O | alpha | 2.3652 | 335.743 | TRUE | FALSE |
| CHEMBL3983719 | N[C@H](C(=O)O)C(NS(=O)(=O)c1ccc(F)cc1)C(=O)O | alpha | -1.0309 | 306.271 | TRUE | TRUE |
| CHEMBL3989075 | CCc1cc2c(NC(CC(C)C)C(=O)O)nc(C)nc2s1 | alpha | 3.47332 | 307.419 | TRUE | FALSE |
| CHEMBL3989166 | Cc1nc(NC(C(=O)O)C(C)C)c2c(C)c(C)sc2n1 | alpha | 3.13766 | 293.392 | TRUE | FALSE |
| CHEMBL3989202 | CCc1nc(NC(C(=O)O)C(C)C)c2cc(CC)sc2n1 | alpha | 3.3372 | 307.419 | TRUE | FALSE |
| CHEMBL3989298 | CCc1cc2c(NC(C(=O)O)C(C)C)nc(C)nc2s1 | alpha | 3.08322 | 293.392 | TRUE | FALSE |
| CHEMBL3990685 | O=C(O)[C@@H]1C[C@H]2[C@@H](O)CCC[C@H]2N1 | alpha | -0.0375 | 185.223 | FALSE | TRUE |
| CHEMBL39908 | O=C(O)[C@H]1C[C@@H](CO)N1 | alpha | -1.2062 | 131.131 | FALSE | TRUE |
| CHEMBL39936 | Cc1cc2nc(CC(N)C(=O)O)n(CP(=O)(O)O)c2cc1C | alpha | 0.74274 | 327.277 | TRUE | FALSE |
| CHEMBL39943 | NC(CCc1nc2ccccc2n1CP(=O)(O)O)C(=O)O | alpha | 0.516 | 313.25 | TRUE | FALSE |
| CHEMBL40023 | NC(CCc1ccccc1CP(=O)(O)O)C(=O)O | alpha | 0.7088 | 273.225 | TRUE | FALSE |
| CHEMBL40024 | O=C(O)[C@@H]1C[C@H](CCCP(=O)(O)O)CCN1 | alpha | 0.3971 | 251.219 | FALSE | TRUE |
| CHEMBL40123 | N[C@@H](C[C@H](CC(c1ccccc1)c1ccccc1)C(=O)O)C(=O)O | alpha | 2.7114 | 327.38 | TRUE | TRUE |
| CHEMBL403077 | O=C(O)[C@H]1NCCC[C@H]1O | alpha | -0.8161 | 145.158 | FALSE | TRUE |
| CHEMBL403386 | O=C(O)[C@@H]1NCCC[C@@H]1O | alpha | -0.8161 | 145.158 | FALSE | TRUE |
| CHEMBL403603 | O=C(O)[C@H]1NCC=C[C@@H]1O | alpha | -1.0401 | 143.142 | FALSE | TRUE |
| CHEMBL403604 | O=C(O)[C@H]1NCC=C[C@H]1O | alpha | -1.0401 | 143.142 | FALSE | TRUE |
| CHEMBL403757 | O=C(O)[C@H]1NCCC[C@@H]1O | alpha | -0.8161 | 145.158 | FALSE | TRUE |
| CHEMBL40449 | N[C@H](Cc1nc2ccc(Cl)cc2cc1CP(=O)(O)O)C(=O)O | alpha | 1.5203 | 344.691 | TRUE | TRUE |
| CHEMBL404859 | O=C(O)[C@@H]1NCCC[C@H]1O | alpha | -0.8161 | 145.158 | FALSE | TRUE |
| CHEMBL405231 | CC(=O)O.N/N=C(\N)NCCC[C@H](N)C(=O)O | alpha | -1.9526 | 249.271 | FALSE | TRUE |
| CHEMBL405609 | CCC(SC[C@H](N)C(=O)O)(c1ccccc1)c1ccccc1 | alpha | 3.4853 | 315.438 | TRUE | TRUE |
| CHEMBL405611 | CC(SC[C@H](N)C(=O)O)(c1ccccc1)c1ccccc1 | alpha | 3.0952 | 301.411 | TRUE | TRUE |
| CHEMBL4059673 | N[C@@H](CCP(=O)(O)C(O)c1c[nH]c2ncccc12)C(=O)O | alpha | 0.6262 | 313.25 | TRUE | TRUE |
| CHEMBL4059687 | O=C(O)C1CC2(C(=O)O)C=CC(O)C(C2)N1 | alpha | -0.8067 | 227.216 | FALSE | FALSE |
| CHEMBL4059945 | Cl.N#Cc1cccc(C[C@H]2CN[C@H](C(=O)O)C2)c1 | alpha | 1.58528 | 266.728 | TRUE | TRUE |
| CHEMBL4059974 | N[C@@H](CCP(=O)(O)C(O)c1ccc(O)nc1)C(=O)O | alpha | -0.1495 | 290.212 | TRUE | TRUE |
| CHEMBL4060165 | Cl.O=C(O)[C@@H]1C[C@@H](Cc2ccccc2Br)CN1 | alpha | 2.4761 | 320.614 | TRUE | TRUE |
| CHEMBL4060341 | N[C@H](C(=O)O)c1cccc2ccccc12 | alpha | 1.9242 | 201.225 | TRUE | TRUE |
| CHEMBL4060745 | Cl.O=C(O)[C@H]1NCC[C@@H]1c1cccc(B(O)O)c1 | alpha | -0.6817 | 271.509 | TRUE | TRUE |
| CHEMBL4060934 | O=C(O)C1CN1 | alpha | -0.9572 | 87.078 | FALSE | FALSE |
| CHEMBL4061538 | Cl.Cl.N[C@@H](Cc1ccc([N+]([O-])(CCCl)CCCl)cc1)C(=O)O | alpha | 2.7673 | 394.126 | TRUE | TRUE |
| CHEMBL4062205 | Nc1ccc(Cl)cc1C(=O)C[C@H](N)C(=O)O | alpha | 0.9069 | 242.662 | TRUE | TRUE |
| CHEMBL4062289 | O=C(O)c1cccc(S[C@H]2CN[C@H](C(=O)O)C2)c1 | alpha | 1.292 | 267.306 | TRUE | TRUE |
| CHEMBL4062684 | Cl.O=C(O)[C@@H]1C[C@@H](Cc2cccc(Br)c2)CN1 | alpha | 2.4761 | 320.614 | TRUE | TRUE |
| CHEMBL4063034 | N[C@@H](CC1CC1)C(=O)O | alpha | 0.1984 | 129.159 | FALSE | TRUE |
| CHEMBL4063058 | Cc1cccc(C(=O)O)c1O[C@H]1CN[C@H](C(=O)O)C1 | alpha | 0.88712 | 265.265 | TRUE | TRUE |
| CHEMBL4063402 | CC(C)(C)c1ccc(C[C@H]2CN[C@H](C(=O)O)C2)cc1.Cl | alpha | 3.0111 | 297.826 | TRUE | TRUE |
| CHEMBL4063644 | NC(C(=O)O)c1cccc(F)c1 | alpha | 0.9101 | 169.155 | TRUE | FALSE |
| CHEMBL4063860 | CC[C@H](C)[C@H](NCC(=O)NC1CCCCC1)C(=O)O | alpha | 1.5242 | 270.373 | FALSE | TRUE |
| CHEMBL4063884 | C#CC[C@H]1CN[C@H](C(=O)O)C1.Cl | alpha | 0.4942 | 189.642 | FALSE | TRUE |
| CHEMBL4064030 | O=C(O)[C@@H]1C[C@@H](Cc2ccc([N+](=O)[O-])cc2)CN1 | alpha | 1.2 | 250.254 | TRUE | TRUE |
| CHEMBL4064178 | NC(C(=O)O)C1CC2C=CC1C2 | alpha | 0.6105 | 167.208 | FALSE | FALSE |
| CHEMBL4064187 | N[C@@H](CCP(=O)(O)Cc1cccc([N+](=O)[O-])c1)C(=O)O | alpha | 1.1672 | 302.223 | TRUE | TRUE |
| CHEMBL4064465 | Cl.O=C(O)[C@@H]1C[C@@H](Cc2ccccc2C(F)(F)F)CN1 | alpha | 2.7324 | 309.715 | TRUE | TRUE |
| CHEMBL4064690 | NC(C(=O)O)c1cccc([N+](=O)[O-])c1 | alpha | 0.6792 | 196.162 | TRUE | FALSE |
| CHEMBL4064924 | O=C(O)[C@@H]1C[C@@H](Cc2ccc(Cl)cc2Cl)CN1 | alpha | 2.5986 | 274.147 | TRUE | TRUE |
| CHEMBL4065054 | Cl.O=C(O)c1ccc(O[C@@H]2CN[C@H](C(=O)O)C2)cc1 | alpha | 1.0005 | 287.699 | TRUE | TRUE |
| CHEMBL4065170 | NC(CC(=O)Nc1ccccc1)C(=O)O | alpha | 0.4271 | 208.217 | TRUE | FALSE |
| CHEMBL4065253 | NC(Cn1ncc2ccc(Cl)cc21)C(=O)O | alpha | 1.1016 | 239.662 | TRUE | FALSE |
| CHEMBL4065496 | N[C@@H](CCP(=O)(O)C(O)c1ccc(O)cc1)C(=O)O | alpha | 0.4555 | 289.224 | TRUE | TRUE |
| CHEMBL406621 | N[C@@H](C(=O)O)[C@@H]1CC(O)=NO1 | alpha | -0.9413 | 160.129 | FALSE | TRUE |
| CHEMBL4066249 | N[C@@H](CCP(=O)(O)C(O)c1ccncc1)C(=O)O | alpha | 0.1449 | 274.213 | TRUE | TRUE |
| CHEMBL4066253 | C=CC[C@H]1CN[C@H](C(=O)O)C1 | alpha | 0.6252 | 155.197 | FALSE | TRUE |
| CHEMBL4066560 | NC(C(=O)O)c1ccc([N+](=O)[O-])cc1 | alpha | 0.6792 | 196.162 | TRUE | FALSE |
| CHEMBL4066881 | Cl.O=C(O)[C@@H]1C[C@@H](Cc2cccc(Cl)c2)CN1 | alpha | 2.367 | 276.163 | TRUE | TRUE |
| CHEMBL4067080 | Cc1cccc(C(=O)CC(N)C(=O)O)c1N | alpha | 0.56192 | 222.244 | TRUE | FALSE |
| CHEMBL4067782 | CC(=O)NC1=CC(=O)C(N[C@H](C(=O)O)C(C)C)=CC1=O | alpha | -0.2591 | 280.28 | FALSE | TRUE |
| CHEMBL4068036 | Cl.O=C(O)[C@@H]1C[C@@H](Cc2ccccc2)CN1 | alpha | 1.7136 | 241.718 | TRUE | TRUE |
| CHEMBL4068205 | CC(=O)c1ccc(C(O)P(=O)(O)CC[C@H](N)C(=O)O)cc1 | alpha | 0.9525 | 315.262 | TRUE | TRUE |
| CHEMBL4068579 | Cc1cc(C(N)C(=O)O)ccc1F | alpha | 1.21852 | 183.182 | TRUE | FALSE |
| CHEMBL4068592 | Cl.O=C(O)c1cccc(C[C@H]2CN[C@H](C(=O)O)C2)c1 | alpha | 1.4118 | 285.727 | TRUE | TRUE |
| CHEMBL4068651 | Cl.O=C(O)c1cc([C@H]2CCN[C@@H]2C(=O)O)ccc1Br | alpha | 2.0992 | 350.596 | TRUE | TRUE |
| CHEMBL4068819 | Cl.O=C(O)[C@@H]1C[C@@H](Cc2ccc(F)c(F)c2)CN1 | alpha | 1.9918 | 277.698 | TRUE | TRUE |
| CHEMBL4068976 | O=C(O)Cc1cccc(O[C@H]2CN[C@H](C(=O)O)C2)c1 | alpha | 0.5076 | 265.265 | TRUE | TRUE |
| CHEMBL4069204 | O=C(CN[C@@H](Cc1ccccc1)C(=O)O)NC1CCCCC1 | alpha | 1.7208 | 304.39 | TRUE | TRUE |
| CHEMBL4069527 | NC(C(=O)O)c1ccc(F)c(F)c1 | alpha | 1.0492 | 187.145 | TRUE | FALSE |
| CHEMBL4069702 | N[C@@H](CCC(=O)Nc1ccc2ccccc2c1)C(=O)O | alpha | 1.9704 | 272.304 | TRUE | TRUE |
| CHEMBL4070483 | CCC(C)[C@@H](NC1=CC(=O)C(NC(C)=O)=CC1=O)C(=O)O | alpha | 0.131 | 294.307 | FALSE | TRUE |
| CHEMBL4070817 | Nc1c(Cl)cccc1C(=O)CC(N)C(=O)O | alpha | 0.9069 | 242.662 | TRUE | FALSE |
| CHEMBL4070950 | CC(C)C[C@H](NC(C(=O)NC1CCCCC1)c1ccccc1)C(=O)O | alpha | 3.2655 | 346.471 | TRUE | TRUE |
| CHEMBL4071517 | Cl.O=C(O)[C@@H]1C[C@@H](Cc2ccccc2F)CN1 | alpha | 1.8527 | 259.708 | TRUE | TRUE |
| CHEMBL4072409 | NC(c1cccc([N+](=O)[O-])c1)P(=O)(O)CC[C@H](N)C(=O)O | alpha | 0.6245 | 317.238 | TRUE | TRUE |
| CHEMBL4072680 | NC(C(=O)O)c1ccccc1O | alpha | 0.4766 | 167.164 | TRUE | FALSE |
| CHEMBL407294 | O=C(O)CC(NC(=S)NCc1ccccc1)C(=O)O | alpha | 0.5786 | 282.321 | TRUE | FALSE |
| CHEMBL4073045 | Cl.O=C(O)[C@@H]1C[C@@H](Cc2ccc(Cl)cc2)CN1 | alpha | 2.367 | 276.163 | TRUE | TRUE |
| CHEMBL4073502 | Cl.Cl.Nc1cccc([C@H]2CCN[C@@H]2C(=O)O)c1 | alpha | 1.6425 | 279.167 | TRUE | TRUE |
| CHEMBL4073637 | Cl.O=C(O)c1cccc(O[C@H]2CN[C@H](C(=O)O)C2)c1 | alpha | 1.0005 | 287.699 | TRUE | TRUE |
| CHEMBL4073653 | O=C(O)[C@@H]1C[C@@H](Cc2ccco2)CN1 | alpha | 0.8848 | 195.218 | TRUE | TRUE |
| CHEMBL4073665 | O=C(O)[C@@H]1C[C@@H](Cc2ccsc2)CN1 | alpha | 1.3533 | 211.286 | TRUE | TRUE |
| CHEMBL4073793 | O=C(O)C1NCc2ccccc21 | alpha | 0.9155 | 163.176 | TRUE | FALSE |
| CHEMBL4074114 | N[C@@H](CCP(=O)(O)C(O)c1ccc(C(F)(F)F)cc1)C(=O)O | alpha | 1.7687 | 341.222 | TRUE | TRUE |
| CHEMBL4075713 | CC(C)C[C@H](NC(C(=O)NC1CCCCC1)c1cccnc1)C(=O)O | alpha | 2.6605 | 347.459 | TRUE | TRUE |
| CHEMBL4076031 | N[C@@H](CCP(=O)(O)C(O)c1cccnc1)C(=O)O | alpha | 0.1449 | 274.213 | TRUE | TRUE |
| CHEMBL4076785 | Cl.O=C(O)[C@H]1NCC[C@@H]1c1cccc(Cl)c1 | alpha | 2.2919 | 262.136 | TRUE | TRUE |
| CHEMBL4076857 | N[C@@H](CCP(=O)(O)C(O)c1ccnc(O)c1)C(=O)O | alpha | -0.1495 | 290.212 | TRUE | TRUE |
| CHEMBL4077066 | CC[C@H](C)[C@H](NC(C(=O)NC1CCCCC1)c1ccccc1)C(=O)O | alpha | 3.2655 | 346.471 | TRUE | TRUE |
| CHEMBL4077223 | CC(CC=O)SC[C@H](N)C(=O)O | alpha | 0.109 | 191.252 | FALSE | TRUE |
| CHEMBL4077258 | CC1OC(O)CC1SC[C@@H](N)C(=O)O | alpha | -0.3727 | 221.278 | FALSE | TRUE |
| CHEMBL4077941 | NC(CCC(=O)Nc1ccccc1)C(=O)O | alpha | 0.8172 | 222.244 | TRUE | FALSE |
| CHEMBL4077985 | O=C(O)[C@H](CO)NCc1ccccc1 | alpha | 0.2217 | 195.218 | TRUE | TRUE |
| CHEMBL4078902 | Cl.O=C(O)[C@@H]1C[C@@H](Cc2cccc(F)c2)CN1 | alpha | 1.8527 | 259.708 | TRUE | TRUE |
| CHEMBL4079614 | Cl.Cl.O=C(O)[C@@H]1C[C@@H](Cc2ccccn2)CN1 | alpha | 1.5304 | 279.167 | TRUE | TRUE |
| CHEMBL4080252 | Cl.O=C(O)[C@@H]1C[C@@H](Cc2ccc(C(F)(F)F)cc2)CN1 | alpha | 2.7324 | 309.715 | TRUE | TRUE |
| CHEMBL4080302 | Cl.O=C(O)[C@@H]1C[C@H](Cc2cccc(Cl)c2)CN1 | alpha | 2.367 | 276.163 | TRUE | TRUE |
| CHEMBL4080583 | O=C(O)[C@@H]1C[C@@H](Cc2cccc(F)c2)CN1 | alpha | 1.4309 | 223.247 | TRUE | TRUE |
| CHEMBL40806 | CC(C)C[C@H](N[C@H]1CCc2ccccc2N(CC(=O)O)C1=O)C(=O)O | alpha | 1.5079 | 348.399 | TRUE | TRUE |
| CHEMBL40807 | C=C(C)[C@H]1CN[C@H](C(=O)O)[C@H]1C(=O)O | alpha | -0.0641 | 199.206 | FALSE | TRUE |
| CHEMBL4080922 | O=C(O)[C@@H]1NCC[C@H]1Cc1ccccc1 | alpha | 1.2918 | 205.257 | TRUE | TRUE |
| CHEMBL4081069 | O=C(O)[C@H]1NCC[C@@H]1c1ccc(O)c(O)c1 | alpha | 0.6279 | 223.228 | TRUE | TRUE |
| CHEMBL4081369 | NC(C(=O)O)C(O)c1cccnc1 | alpha | -0.4731 | 182.179 | TRUE | FALSE |
| CHEMBL4081865 | Cl.O=C(O)[C@@H]1C[C@@H](Cc2ccc(Cl)c(Cl)c2)CN1 | alpha | 3.0204 | 310.608 | TRUE | TRUE |
| CHEMBL4082832 | Cl.O=C(O)[C@@H]1C[C@@H](Cc2ccncc2)CN1 | alpha | 1.1086 | 242.706 | TRUE | TRUE |
| CHEMBL4083155 | NC(C(=O)O)c1ccc2c(c1)OCO2 | alpha | 0.4997 | 195.174 | TRUE | FALSE |
| CHEMBL4083194 | Cc1ccc(SC[C@H](N)C(=O)O)cc1 | alpha | 1.49902 | 211.286 | TRUE | TRUE |
| CHEMBL4083417 | N[C@@H](CSCCC=O)C(=O)O | alpha | -0.2795 | 177.225 | FALSE | TRUE |
| CHEMBL4083473 | Cc1ccccc1C[C@H]1CN[C@H](C(=O)O)C1.Cl | alpha | 2.02202 | 255.745 | TRUE | TRUE |
| CHEMBL4083586 | Cl.N#Cc1cccc([C@H]2CCN[C@@H]2C(=O)O)c1 | alpha | 1.51018 | 252.701 | TRUE | TRUE |
| CHEMBL4083894 | Cl.O=C(O)[C@@H]1C[C@@H](Cc2ccc(Cl)cc2Cl)CN1 | alpha | 3.0204 | 310.608 | TRUE | TRUE |
| CHEMBL4084004 | O=C(O)c1ccccc1S[C@H]1CN[C@H](C(=O)O)C1 | alpha | 1.292 | 267.306 | TRUE | TRUE |
| CHEMBL4084026 | O=C(O)[C@@H]1C[C@@H](c2ccccc2)CN1 | alpha | 1.2167 | 191.23 | TRUE | TRUE |
| CHEMBL4084540 | Cl.O=C(O)c1cc([C@H]2CCN[C@@H]2C(=O)O)ccc1Cl | alpha | 1.9901 | 306.145 | TRUE | TRUE |
| CHEMBL4084974 | Cl.O=C(O)c1cccc(O[C@@H]2CN[C@H](C(=O)O)C2)c1 | alpha | 1.0005 | 287.699 | TRUE | TRUE |
| CHEMBL4085254 | CC1NC(NCCC[C@H](N)C(=O)O)=NC1=O | alpha | -1.3576 | 228.252 | FALSE | TRUE |
| CHEMBL4085994 | Cl.O=C(O)[C@@H]1C[C@@H](Cc2ccc(I)cc2)CN1 | alpha | 2.3182 | 367.614 | TRUE | TRUE |
| CHEMBL4086199 | NC(C(=O)O)c1cccc(C(F)(F)F)c1 | alpha | 1.7898 | 219.162 | TRUE | FALSE |
| CHEMBL4086387 | O=C(O)[C@H]1NCC[C@@H]1c1ccccc1 | alpha | 1.2167 | 191.23 | TRUE | TRUE |
| CHEMBL4086516 | C#CC[C@H]1CN[C@H](C(=O)O)C1 | alpha | 0.0724 | 153.181 | FALSE | TRUE |
| CHEMBL4086929 | COc1ccc(C(O)P(=O)(O)CC[C@H](N)C(=O)O)cc1 | alpha | 0.7585 | 303.251 | TRUE | TRUE |
| CHEMBL4087475 | O=C(O)c1cccc(S[C@@H]2CN[C@H](C(=O)O)C2)c1 | alpha | 1.292 | 267.306 | TRUE | TRUE |
| CHEMBL408756 | CN(N)/C(N)=N\CCC[C@H](N)C(=O)O | alpha | -1.7013 | 203.246 | FALSE | TRUE |
| CHEMBL4088574 | NC(C(=O)O)c1ccc2c(c1)OCCO2 | alpha | 0.5422 | 209.201 | TRUE | FALSE |
| CHEMBL4088806 | N[C@H](C(=O)O)c1ccc(F)cc1 | alpha | 0.9101 | 169.155 | TRUE | TRUE |
| CHEMBL4088958 | Cl.NC(=O)c1cccc([C@H]2CCN[C@@H]2C(=O)O)c1 | alpha | 0.7374 | 270.716 | TRUE | TRUE |
| CHEMBL408899 | NC(C(=O)O)C1CC(O)=NO1 | alpha | -0.9413 | 160.129 | FALSE | FALSE |
| CHEMBL4088995 | CSCC[C@@H](NC1=CC(=O)C(NC(C)=O)=CC1=O)C(=O)O | alpha | -0.162 | 312.347 | FALSE | TRUE |
| CHEMBL4090214 | Nc1ccc(Br)cc1C(=O)C[C@H](N)C(=O)O | alpha | 1.016 | 287.113 | TRUE | TRUE |
| CHEMBL4090312 | Nc1ccc(C(O)P(=O)(O)CC[C@H](N)C(=O)O)cc1 | alpha | 0.3321 | 288.24 | TRUE | TRUE |
| CHEMBL4090544 | NC(C(=O)O)c1ccccc1Cl | alpha | 1.4244 | 185.61 | TRUE | FALSE |
| CHEMBL4090699 | N#Cc1cccc(C(O)P(=O)(O)CC[C@H](N)C(=O)O)c1 | alpha | 0.62158 | 298.235 | TRUE | TRUE |
| CHEMBL4090961 | CC(=O)c1ccc(O[C@H]2CN[C@H](C(=O)O)C2)c(C(=O)O)c1 | alpha | 0.7813 | 293.275 | TRUE | TRUE |
| CHEMBL4091261 | O=C(O)[C@H]1NCCC1c1ccccc1 | alpha | 1.2167 | 191.23 | TRUE | TRUE |
| CHEMBL4091369 | Cl.O=C(O)[C@H]1NCC[C@@H]1c1cccc(C(F)(F)F)c1 | alpha | 2.6573 | 295.688 | TRUE | TRUE |
| CHEMBL4091662 | C[C@H]1CCN[C@@H]1C(=O)O | alpha | 0.069 | 129.159 | FALSE | TRUE |
| CHEMBL4091914 | N[C@H](C(=O)O)C1CC1 | alpha | -0.1917 | 115.132 | FALSE | TRUE |
| CHEMBL4091926 | N[C@@H](C(=O)O)C1=CCC=CC1 | alpha | 0.6747 | 153.181 | FALSE | TRUE |
| CHEMBL4092520 | CNC(C(=O)O)c1ccc(OC)cc1 | alpha | 1.0403 | 195.218 | TRUE | FALSE |
| CHEMBL4092574 | CC(C)C[C@H](NCC(=O)NC1CCCCC1)C(=O)O | alpha | 1.5242 | 270.373 | FALSE | TRUE |
| CHEMBL4092735 | O=C(O)c1ccccc1S[C@@H]1CN[C@H](C(=O)O)C1 | alpha | 1.292 | 267.306 | TRUE | TRUE |
| CHEMBL4093105 | Cl.O=C(O)[C@@H]1C[C@@H](Cc2ccccc2I)CN1 | alpha | 2.3182 | 367.614 | TRUE | TRUE |
| CHEMBL4093745 | O=C(O)[C@@H]1C[C@@H](Cc2ccc(Br)cc2)CN1 | alpha | 2.0543 | 284.153 | TRUE | TRUE |
| CHEMBL4094281 | Cc1ccc(C[C@H]2CN[C@H](C(=O)O)C2)cc1 | alpha | 1.60022 | 219.284 | TRUE | TRUE |
| CHEMBL4094390 | N[C@H](C(=O)O)c1ccco1 | alpha | 0.364 | 141.126 | TRUE | TRUE |
| CHEMBL4094552 | Nc1c(Cl)cc(Br)cc1C(=O)CC(N)C(=O)O | alpha | 1.6694 | 321.558 | TRUE | FALSE |
| CHEMBL4095986 | N[C@@H](CCC(=O)Nc1cccc2ccccc12)C(=O)O | alpha | 1.9704 | 272.304 | TRUE | TRUE |
| CHEMBL4097017 | Cl.O=C(O)[C@@H]1C[C@@H](Cc2ccccc2Cl)CN1 | alpha | 2.367 | 276.163 | TRUE | TRUE |
| CHEMBL4097657 | CC(=O)NC1=CC(=O)C(N[C@H](Cc2ccccc2)C(=O)O)=CC1=O | alpha | 0.3276 | 328.324 | TRUE | TRUE |
| CHEMBL4097766 | O=C(O)Cc1ccccc1O[C@H]1CN[C@H](C(=O)O)C1 | alpha | 0.5076 | 265.265 | TRUE | TRUE |
| CHEMBL4098554 | Cl.O=C(O)[C@@H]1C[C@@H](Cc2ccccc2[N+](=O)[O-])CN1 | alpha | 1.6218 | 286.715 | TRUE | TRUE |
| CHEMBL4098618 | O=C(O)[C@@H]1C[C@@H](Cc2ccccc2F)CN1 | alpha | 1.4309 | 223.247 | TRUE | TRUE |
| CHEMBL4098956 | O=C(O)[C@@H]1NCC[C@H]1Cc1ccc(F)cc1 | alpha | 1.4309 | 223.247 | TRUE | TRUE |
| CHEMBL4099104 | Cl.O=C(O)c1cc([C@H]2CCN[C@@H]2C(=O)O)ccc1F | alpha | 1.4758 | 289.69 | TRUE | TRUE |
| CHEMBL4100460 | Cl.O=C(O)c1ccc(O[C@H]2CN[C@H](C(=O)O)C2)cc1 | alpha | 1.0005 | 287.699 | TRUE | TRUE |
| CHEMBL4100815 | Cl.N#Cc1ccc(C[C@H]2CN[C@H](C(=O)O)C2)cc1 | alpha | 1.58528 | 266.728 | TRUE | TRUE |
| CHEMBL4100876 | Cl.O=C(O)[C@@H]1C[C@@H](Cc2cccc(C(F)(F)F)c2)CN1 | alpha | 2.7324 | 309.715 | TRUE | TRUE |
| CHEMBL4101153 | NC(C(=O)O)c1ccc(CO)c(O)c1 | alpha | -0.0311 | 197.19 | TRUE | FALSE |
| CHEMBL4101466 | Cl.N#Cc1ccccc1C[C@H]1CN[C@H](C(=O)O)C1 | alpha | 1.58528 | 266.728 | TRUE | TRUE |
| CHEMBL4101687 | CC(O)[C@H](C)[C@H](N)C(=O)O | alpha | -0.5848 | 147.174 | FALSE | TRUE |
| CHEMBL4101691 | N[C@H](C(=O)O)c1ccc(C(F)(F)F)cc1 | alpha | 1.7898 | 219.162 | TRUE | TRUE |
| CHEMBL4101796 | Nc1ncc(C(O)P(=O)(O)CC[C@H](N)C(=O)O)s1 | alpha | -0.2114 | 295.257 | TRUE | TRUE |
| CHEMBL4102013 | O=C(O)[C@@H]1C[C@@H](Cc2ccc(F)c(F)c2)CN1 | alpha | 1.57 | 241.237 | TRUE | TRUE |
| CHEMBL4102130 | N[C@H](C(=O)O)c1ccsc1 | alpha | 0.8325 | 157.194 | TRUE | TRUE |
| CHEMBL4102364 | O=C(CN[C@@H](Cc1ccccc1)C(=O)O)NCc1ccccc1 | alpha | 1.5883 | 312.369 | TRUE | TRUE |
| CHEMBL4102488 | Cc1ccc([C@H]2CCN[C@@H]2C(=O)O)cc1C(=O)O.Cl | alpha | 1.64512 | 285.727 | TRUE | TRUE |
| CHEMBL4102802 | O=C(O)[C@@H]1CS[C@H](c2ccc(F)cc2)N1 | alpha | 1.6139 | 227.26 | TRUE | TRUE |
| CHEMBL4103084 | [N-]=[N+]=NCC[C@H](N)C(=O)O | alpha | 0.0987 | 144.134 | FALSE | TRUE |
| CHEMBL4103180 | Cl.O=C(O)[C@@H]1C[C@@H](Cc2ccc(F)cc2)CN1 | alpha | 1.8527 | 259.708 | TRUE | TRUE |
| CHEMBL4103332 | N[C@@H](CCP(=O)(O)Cc1cccc(C(=O)O)c1)C(=O)O | alpha | 0.9572 | 301.235 | TRUE | TRUE |
| CHEMBL4103550 | CC(C)[C@@H](NC1=CC(=O)C(NC(=O)c2ccccc2)=CC1=O)C(=O)O | alpha | 1.0348 | 342.351 | TRUE | TRUE |
| CHEMBL4103658 | Cc1ccc(C(N)C(=O)O)cc1Cl | alpha | 1.73282 | 199.637 | TRUE | FALSE |
| CHEMBL4103869 | Cl.O=C(O)[C@@H]1C[C@@H](Oc2cccc(Cl)c2)CN1 | alpha | 1.9557 | 278.135 | TRUE | TRUE |
| CHEMBL4104042 | Cc1cc(Br)cc(C(=O)CC(N)C(=O)O)c1N | alpha | 1.32442 | 301.14 | TRUE | FALSE |
| CHEMBL4105362 | CC(=O)NC1=CC(=O)C(N[C@@H](C(=O)O)C(C)C)=CC1=O | alpha | -0.2591 | 280.28 | FALSE | TRUE |
| CHEMBL4105498 | Cc1ccc(O[C@H]2CN[C@H](C(=O)O)C2)c(C(=O)O)c1 | alpha | 0.88712 | 265.265 | TRUE | TRUE |
| CHEMBL4105620 | NC(C(=O)O)C(O)c1cccs1 | alpha | 0.1934 | 187.22 | TRUE | FALSE |
| CHEMBL4113291 | CC(Nc1ccc(S(C)(=O)=O)cc1[N+](=O)[O-])C(=O)O | alpha | 0.8833 | 288.281 | TRUE | FALSE |
| CHEMBL4116013 | Cc1ccccc1C[C@H]1CN[C@H](C(=O)O)C1 | alpha | 1.60022 | 219.284 | TRUE | TRUE |
| CHEMBL4116041 | NC(=O)c1cccc([C@H]2CCN[C@@H]2C(=O)O)c1 | alpha | 0.3156 | 234.255 | TRUE | TRUE |
| CHEMBL4116079 | O=C(O)[C@@H]1C[C@@H](Oc2cccc(Cl)c2)CN1 | alpha | 1.5339 | 241.674 | TRUE | TRUE |
| CHEMBL4116117 | O=C(O)[C@H]1NCC[C@@H]1c1cccc(C(F)(F)F)c1 | alpha | 2.2355 | 259.227 | TRUE | TRUE |
| CHEMBL4116128 | O=C(O)[C@@H]1C[C@@H](Cc2ccc(Cl)cc2)CN1 | alpha | 1.9452 | 239.702 | TRUE | TRUE |
| CHEMBL4116208 | O=C(O)[C@H]1NCC[C@@H]1c1cccc(Cl)c1 | alpha | 1.8701 | 225.675 | TRUE | TRUE |
| CHEMBL4116235 | N#Cc1ccccc1C[C@H]1CN[C@H](C(=O)O)C1 | alpha | 1.16348 | 230.267 | TRUE | TRUE |
| CHEMBL4116241 | O=C(O)c1ccc(O[C@@H]2CN[C@H](C(=O)O)C2)cc1 | alpha | 0.5787 | 251.238 | TRUE | TRUE |
| CHEMBL4116280 | CC(C)(C)c1ccc(C[C@H]2CN[C@H](C(=O)O)C2)cc1 | alpha | 2.5893 | 261.365 | TRUE | TRUE |
| CHEMBL4116285 | O=C(O)[C@@H]1C[C@@H](Cc2ccccc2Br)CN1 | alpha | 2.0543 | 284.153 | TRUE | TRUE |
| CHEMBL4116371 | O=C(O)c1cccc(O[C@@H]2CN[C@H](C(=O)O)C2)c1 | alpha | 0.5787 | 251.238 | TRUE | TRUE |
| CHEMBL4116540 | O=C(O)c1cc([C@H]2CCN[C@@H]2C(=O)O)ccc1F | alpha | 1.054 | 253.229 | TRUE | TRUE |
| CHEMBL4116695 | O=C(O)[C@@H]1C[C@@H](Cc2ccc(I)cc2)CN1 | alpha | 1.8964 | 331.153 | TRUE | TRUE |
| CHEMBL4116701 | O=C(O)c1cccc(O[C@H]2CN[C@H](C(=O)O)C2)c1 | alpha | 0.5787 | 251.238 | TRUE | TRUE |
| CHEMBL4116731 | O=C(O)c1cc([C@H]2CCN[C@@H]2C(=O)O)ccc1Br | alpha | 1.6774 | 314.135 | TRUE | TRUE |
| CHEMBL4116825 | N#Cc1cccc(C[C@H]2CN[C@H](C(=O)O)C2)c1 | alpha | 1.16348 | 230.267 | TRUE | TRUE |
| CHEMBL4116826 | O=C(O)[C@@H]1C[C@@H](Cc2ccc(F)cc2)CN1 | alpha | 1.4309 | 223.247 | TRUE | TRUE |
| CHEMBL4116845 | O=C(O)[C@H]1NCC[C@@H]1c1cccc(B(O)O)c1 | alpha | -1.1035 | 235.048 | TRUE | TRUE |
| CHEMBL4116896 | O=C(O)[C@@H]1C[C@@H](Cc2ccccc2I)CN1 | alpha | 1.8964 | 331.153 | TRUE | TRUE |
| CHEMBL4116901 | O=C(O)c1cccc(C[C@H]2CN[C@H](C(=O)O)C2)c1 | alpha | 0.99 | 249.266 | TRUE | TRUE |
| CHEMBL4117049 | O=C(O)[C@@H]1C[C@H](Cc2cccc(Cl)c2)CN1 | alpha | 1.9452 | 239.702 | TRUE | TRUE |
| CHEMBL4117156 | O=C(O)[C@@H]1C[C@@H](Cc2ccccc2)CN1 | alpha | 1.2918 | 205.257 | TRUE | TRUE |
| CHEMBL4117180 | O=C(O)[C@@H]1C[C@@H](Cc2cccc(Cl)c2)CN1 | alpha | 1.9452 | 239.702 | TRUE | TRUE |
| CHEMBL4117181 | O=C(O)[C@@H]1C[C@@H](Cc2ccc(Cl)c(Cl)c2)CN1 | alpha | 2.5986 | 274.147 | TRUE | TRUE |
| CHEMBL4117326 | O=C(O)c1cc([C@H]2CCN[C@@H]2C(=O)O)ccc1Cl | alpha | 1.5683 | 269.684 | TRUE | TRUE |
| CHEMBL4117445 | O=C(O)[C@@H]1C[C@@H](Cc2ccncc2)CN1 | alpha | 0.6868 | 206.245 | TRUE | TRUE |
| CHEMBL4117514 | O=C(O)[C@@H]1C[C@@H](Cc2cccc(Br)c2)CN1 | alpha | 2.0543 | 284.153 | TRUE | TRUE |
| CHEMBL4117516 | N[C@@H](Cc1ccc([N+]([O-])(CCCl)CCCl)cc1)C(=O)O | alpha | 1.9237 | 321.204 | TRUE | TRUE |
| CHEMBL4117535 | Cc1ccc([C@H]2CCN[C@@H]2C(=O)O)cc1C(=O)O | alpha | 1.22332 | 249.266 | TRUE | TRUE |
| CHEMBL4117574 | O=C(O)[C@@H]1C[C@@H](Cc2ccc(C(F)(F)F)cc2)CN1 | alpha | 2.3106 | 273.254 | TRUE | TRUE |
| CHEMBL4117606 | N#Cc1cccc([C@H]2CCN[C@@H]2C(=O)O)c1 | alpha | 1.08838 | 216.24 | TRUE | TRUE |
| CHEMBL4117640 | O=C(O)c1ccc(O[C@H]2CN[C@H](C(=O)O)C2)cc1 | alpha | 0.5787 | 251.238 | TRUE | TRUE |
| CHEMBL4117705 | O=C(O)[C@@H]1C[C@@H](Cc2ccccc2Cl)CN1 | alpha | 1.9452 | 239.702 | TRUE | TRUE |
| CHEMBL4117706 | O=C(O)[C@@H]1C[C@@H](Cc2ccccn2)CN1 | alpha | 0.6868 | 206.245 | TRUE | TRUE |
| CHEMBL4117734 | Nc1cccc([C@H]2CCN[C@@H]2C(=O)O)c1 | alpha | 0.7989 | 206.245 | TRUE | TRUE |
| CHEMBL4117779 | O=C(O)[C@@H]1C[C@@H](Cc2cccc(C(F)(F)F)c2)CN1 | alpha | 2.3106 | 273.254 | TRUE | TRUE |
| CHEMBL4117842 | O=C(O)[C@@H]1C[C@@H](Cc2ccccc2C(F)(F)F)CN1 | alpha | 2.3106 | 273.254 | TRUE | TRUE |
| CHEMBL4117843 | N#Cc1ccc(C[C@H]2CN[C@H](C(=O)O)C2)cc1 | alpha | 1.16348 | 230.267 | TRUE | TRUE |
| CHEMBL4117844 | O=C(O)[C@@H]1C[C@@H](Cc2ccccc2[N+](=O)[O-])CN1 | alpha | 1.2 | 250.254 | TRUE | TRUE |
| CHEMBL412649 | Cc1ncc(CO)c(CN[C@@H](C)C(=O)O)c1O | alpha | 0.15062 | 240.259 | TRUE | TRUE |
| CHEMBL4128630 | NC(=O)CCCCC[C@H](N)C(=O)O | alpha | -0.1659 | 188.227 | FALSE | TRUE |
| CHEMBL413468 | NCCN[C@@H](Cc1cn(CCCO)cn1)C(=O)O | alpha | -1.1905 | 256.306 | TRUE | TRUE |
| CHEMBL41352 | CNC(C(=O)O)c1cc(O)no1 | alpha | -0.2747 | 172.14 | TRUE | FALSE |
| CHEMBL413545 | NNC(N)NCCCC(N)C(=O)O | alpha | -2.5263 | 191.235 | FALSE | FALSE |
| CHEMBL413572 | NC(CCSCc1cccc(Cl)c1Cl)C(=O)O | alpha | 3.0286 | 294.203 | TRUE | FALSE |
| CHEMBL41492 | O=C(O)C(Cc1ccccc1)NP(=O)(O)O | alpha | 0.3646 | 245.171 | TRUE | FALSE |
| CHEMBL415760 | CC(=N)NC[C@H](F)CC[C@H](N)C(=O)O | alpha | 0.10337 | 205.233 | FALSE | TRUE |
| CHEMBL415865 | Cc1ccc(S(=O)(=O)NC(C(=O)O)c2ccccc2)cc1 | alpha | 2.09922 | 305.355 | TRUE | FALSE |
| CHEMBL4159467 | N[C@H](C(=O)O)[C@H](OCC1CC1)C(=O)O | alpha | -0.7219 | 203.194 | FALSE | TRUE |
| CHEMBL416060 | O=C(O)[C@@H]1C[C@H](C/C=C/P(=O)(O)O)CCN1 | alpha | 0.5207 | 249.203 | FALSE | TRUE |
| CHEMBL4161764 | N[C@@H](C(=O)O)[C@H](OCC1CC1)C(=O)O | alpha | -0.7219 | 203.194 | FALSE | TRUE |
| CHEMBL4163105 | N[C@H](C(=O)O)[C@H](OCc1cccs1)C(=O)O | alpha | 0.1298 | 245.256 | TRUE | TRUE |
| CHEMBL4163625 | N[C@H](C(=O)O)[C@H](OCC1CCCCCC1)C(=O)O | alpha | 0.8385 | 259.302 | FALSE | TRUE |
| CHEMBL4164207 | N[C@H](C(=O)O)[C@H](OCC1CCCCC1)C(=O)O | alpha | 0.4484 | 245.275 | FALSE | TRUE |
| CHEMBL4165574 | N[C@H](C(=O)O)[C@H](OCC1CCCC1)C(=O)O | alpha | 0.0583 | 231.248 | FALSE | TRUE |
| CHEMBL41659 | N[C@H](C(=O)O)[C@@H]1C[C@H]1C(=O)O | alpha | -0.881 | 159.141 | FALSE | TRUE |
| CHEMBL4166282 | N#CCO[C@H](C(=O)O)[C@H](N)C(=O)O | alpha | -1.60832 | 188.139 | FALSE | TRUE |
| CHEMBL4166545 | N[C@H](C(=O)O)[C@H](OCc1ccco1)C(=O)O | alpha | -0.3387 | 229.188 | TRUE | TRUE |
| CHEMBL4166966 | C#CCO[C@H](C(=O)O)[C@H](N)C(=O)O | alpha | -1.4987 | 187.151 | FALSE | TRUE |
| CHEMBL41680 | O=C(O)[C@@H]1C[C@H](C(=O)O)N1 | alpha | -1.1139 | 145.114 | FALSE | TRUE |
| CHEMBL4168620 | N#CCO[C@H](C(=O)O)[C@@H](N)C(=O)O | alpha | -1.60832 | 188.139 | FALSE | TRUE |
| CHEMBL4169695 | N[C@H](C(=O)O)[C@H](OCc1ccsc1)C(=O)O | alpha | 0.1298 | 245.256 | TRUE | TRUE |
| CHEMBL4173825 | N[C@H](C(=O)O)[C@H](OCC1CCC1)C(=O)O | alpha | -0.3318 | 217.221 | FALSE | TRUE |
| CHEMBL417386 | Cc1cc(C)c2c(c1)N[C@@H](C(=O)O)[C@@H](C)C2=O | alpha | 2.00094 | 233.267 | TRUE | TRUE |
| CHEMBL4174337 | CC(C)[C@H](Nc1nccc(Nc2cccc(CN)c2)n1)C(=O)O | alpha | 2.2 | 315.377 | TRUE | TRUE |
| CHEMBL4175200 | C/C(=N/N)NCCCC[C@H](N)C(=O)O | alpha | -0.5497 | 202.258 | FALSE | TRUE |
| CHEMBL4175625 | NC(CCCCCCC(N)C(=O)O)C(=O)O | alpha | 0.1508 | 232.28 | FALSE | FALSE |
| CHEMBL4176899 | N[C@@H](C(=O)O)[C@H](OCc1ccsc1)C(=O)O | alpha | 0.1298 | 245.256 | TRUE | TRUE |
| CHEMBL4177165 | N[C@H](C(=O)O)[C@H](OCc1ccoc1)C(=O)O | alpha | -0.3387 | 229.188 | TRUE | TRUE |
| CHEMBL417846 | NC(Cc1cccc(CCP(=O)(O)O)c1)C(=O)O | alpha | 0.3612 | 273.225 | TRUE | FALSE |
| CHEMBL417847 | NC(C(=O)O)c1ccc(CCP(=O)(O)O)cc1 | alpha | 0.4912 | 259.198 | TRUE | FALSE |
| CHEMBL418204 | NC(Cc1cccc(CP(=O)(O)O)c1)C(=O)O | alpha | 0.3187 | 259.198 | TRUE | FALSE |
| CHEMBL41852 | N[C@H](CCC(=O)N1c2ccccc2C[C@H]1C(=O)O)C(=O)O | alpha | 0.221 | 292.291 | TRUE | TRUE |
| CHEMBL419673 | C[C@H](NCc1ccc(OCc2ccccc2)cc1)C(=O)O | alpha | 2.8283 | 285.343 | TRUE | TRUE |
| CHEMBL41985 | CCCC[C@H](N[C@H]1CCc2ccccc2N(CC(=O)O)C1=O)C(=O)O | alpha | 1.652 | 348.399 | TRUE | TRUE |
| CHEMBL419860 | NC(CC1CCCC(CS(=O)(=O)[O-])C1)C(=O)O.[Na+] | alpha | -2.856 | 287.313 | FALSE | FALSE |
| CHEMBL419885 | Cc1ccccc1Oc1ccccc1S(=O)(=O)N[C@H](C=O)C(=O)O | alpha | 1.71772 | 349.364 | TRUE | TRUE |
| CHEMBL420125 | NC(C(=O)O)c1c(O)noc1CO | alpha | -1.0431 | 188.139 | TRUE | FALSE |
| CHEMBL4202695 | NC(Cc1cc(-c2cncc(O)c2)c(Cl)c([N+](=O)[O-])c1)C(=O)O | alpha | 1.9702 | 337.719 | TRUE | FALSE |
| CHEMBL420305 | O=C(O)[C@@H]1C[Se]CN1 | alpha | -0.8772 | 180.065 | FALSE | TRUE |
| CHEMBL4203096 | N[C@@H](CCC(=O)NNc1cc(O)cc(O)c1)C(=O)O | alpha | -0.267 | 269.257 | TRUE | TRUE |
| CHEMBL4204686 | NC(Cc1ccc([N+](=O)[O-])c(-c2ccc(O)cc2)c1)C(=O)O | alpha | 1.9218 | 302.286 | TRUE | FALSE |
| CHEMBL4205175 | NC(Cc1cc(-c2ccc(O)cc2)c(Cl)c([N+](=O)[O-])c1)C(=O)O | alpha | 2.5752 | 336.731 | TRUE | FALSE |
| CHEMBL4205389 | CC(C)C1=C(P(=O)(O)O)N[C@H](C(=O)O)CS1 | alpha | 0.7788 | 267.243 | FALSE | TRUE |
| CHEMBL4205785 | Nc1c(Cl)cc(CC(N)C(=O)O)cc1-c1cccc(O)c1 | alpha | 2.2492 | 306.749 | TRUE | FALSE |
| CHEMBL4206294 | NC(Cc1ccc([N+](=O)[O-])c(-c2cccc(O)c2)c1)C(=O)O | alpha | 1.9218 | 302.286 | TRUE | FALSE |
| CHEMBL4206690 | N[C@@H](CCC(=O)Nc1ccccc1O)C(=O)O | alpha | 0.5228 | 238.243 | TRUE | TRUE |
| CHEMBL4206982 | N[C@@H](CC(=O)CO)C(=O)O | alpha | -1.6502 | 147.13 | FALSE | TRUE |
| CHEMBL4207107 | CCCCCCC1=C(P(=O)(O)O)N[C@H](C(=O)O)CS1 | alpha | 2.0932 | 309.324 | FALSE | TRUE |
| CHEMBL4208296 | Cc1ccc(NNC(=O)CC[C@H](N)C(=O)O)cc1 | alpha | 0.63022 | 251.286 | TRUE | TRUE |
| CHEMBL4208422 | N[C@@H](CCC(=O)NNc1ccc(F)cc1)C(=O)O | alpha | 0.4609 | 255.249 | TRUE | TRUE |
| CHEMBL4208730 | Cc1cccc(NNC(=O)CC[C@H](N)C(=O)O)c1 | alpha | 0.63022 | 251.286 | TRUE | TRUE |
| CHEMBL4209031 | Nc1c(-c2cccc(O)c2)cc(CC(N)C(=O)O)cc1[N+](=O)[O-] | alpha | 1.504 | 317.301 | TRUE | FALSE |
| CHEMBL4209124 | O=C(O)C1=CSC[C@@H](C(=O)O)N1 | alpha | -0.298 | 189.192 | FALSE | TRUE |
| CHEMBL4209470 | N[C@@H](CCC(=O)NNc1ccccc1F)C(=O)O | alpha | 0.4609 | 255.249 | TRUE | TRUE |
| CHEMBL4209549 | NC(Cc1cc(Cl)cc(-c2ccc(O)cc2)c1)C(=O)O | alpha | 2.667 | 291.734 | TRUE | FALSE |
| CHEMBL4210650 | NC(Cc1cc(Cl)cc(-c2cccc(O)c2)c1)C(=O)O | alpha | 2.667 | 291.734 | TRUE | FALSE |
| CHEMBL4211059 | N[C@@H](Cc1cc(Cl)cc(-c2cccc(O)c2)c1)C(=O)O | alpha | 2.667 | 291.734 | TRUE | TRUE |
| CHEMBL4211074 | COc1c(Cl)cc(CC(N)C(=O)O)cc1-c1cccc(O)c1 | alpha | 2.6756 | 321.76 | TRUE | FALSE |
| CHEMBL42114 | CC(C)[C@H](NS(=O)(=O)c1ccc(-c2ccc(N)cc2)cc1)C(=O)O | alpha | 2.3233 | 348.424 | TRUE | TRUE |
| CHEMBL4211537 | CS(=O)(=O)c1ccc(NNC(=O)CC[C@H](N)C(=O)O)cc1 | alpha | -0.2747 | 315.351 | TRUE | TRUE |
| CHEMBL4212461 | N[C@@H](CCC(=O)NNc1cccc(F)c1)C(=O)O | alpha | 0.4609 | 255.249 | TRUE | TRUE |
| CHEMBL4212533 | N[C@H](Cc1cc(Cl)cc(-c2cccc(O)c2)c1)C(=O)O | alpha | 2.667 | 291.734 | TRUE | TRUE |
| CHEMBL4213197 | CCCCC1=C(P(=O)(O)O)N[C@H](C(=O)O)CS1 | alpha | 1.313 | 281.27 | FALSE | TRUE |
| CHEMBL4213426 | NC(Cc1cc(-c2cccc(O)c2)cc([N+](=O)[O-])c1)C(=O)O | alpha | 1.9218 | 302.286 | TRUE | FALSE |
| CHEMBL4213952 | NC(Cc1ccc(Cl)c(-c2ccc(O)cc2)c1)C(=O)O | alpha | 2.667 | 291.734 | TRUE | FALSE |
| CHEMBL4214543 | NC(Cc1ccc(Cl)c(-c2cccc(O)c2)c1)C(=O)O | alpha | 2.667 | 291.734 | TRUE | FALSE |
| CHEMBL4214817 | C/N=[N+](\[O-])CC[C@H](N)C(=O)O | alpha | -0.6193 | 161.161 | FALSE | TRUE |
| CHEMBL4215190 | CS(=O)(=O)c1ccc(NNC(=O)CC[C@H](N)C(=O)O)c(F)c1 | alpha | -0.1356 | 333.341 | TRUE | TRUE |
| CHEMBL4215322 | N[C@@H](CCC(=O)NNc1ccc(O)cc1O)C(=O)O | alpha | -0.267 | 269.257 | TRUE | TRUE |
| CHEMBL4215417 | Cc1ccccc1NNC(=O)CC[C@H](N)C(=O)O | alpha | 0.63022 | 251.286 | TRUE | TRUE |
| CHEMBL4215471 | N[C@@H](Cc1ccc(Cl)c(-c2cccc(O)c2)c1)C(=O)O | alpha | 2.667 | 291.734 | TRUE | TRUE |
| CHEMBL4216018 | N[C@H](Cc1ccc(Cl)c(-c2cccc(O)c2)c1)C(=O)O | alpha | 2.667 | 291.734 | TRUE | TRUE |
| CHEMBL4216037 | NC(Cc1cc(Cl)c(O)c(-c2cccc(O)c2)c1)C(=O)O | alpha | 2.3726 | 307.733 | TRUE | FALSE |
| CHEMBL4216443 | Cc1ccc(NNC(=O)CC[C@H](N)C(=O)O)c(C)c1 | alpha | 0.93864 | 265.313 | TRUE | TRUE |
| CHEMBL4216884 | NC(Cc1cc(-c2ccc(O)cc2)cc([N+](=O)[O-])c1)C(=O)O | alpha | 1.9218 | 302.286 | TRUE | FALSE |
| CHEMBL4217996 | Nc1ccc(CC(N)C(=O)O)cc1-c1cccc(O)c1 | alpha | 1.5958 | 272.304 | TRUE | FALSE |
| CHEMBL4218347 | NC(Cc1cc(-c2cccc(O)c2)c(Cl)c([N+](=O)[O-])c1)C(=O)O | alpha | 2.5752 | 336.731 | TRUE | FALSE |
| CHEMBL4218704 | CCc1ccccc1NNC(=O)CC[C@H](N)C(=O)O | alpha | 0.8842 | 265.313 | TRUE | TRUE |
| CHEMBL423104 | N[C@@H](C[C@@H](CCc1ccccc1)C(=O)O)C(=O)O | alpha | 1.122 | 251.282 | TRUE | TRUE |
| CHEMBL42380 | O=C(O)[C@@H]1Nc2cc(Cl)ccc2[C@H]1CO | alpha | 1.2946 | 227.647 | TRUE | TRUE |
| CHEMBL4238000 | N[C@@H](Cc1cccc(C(=O)O)c1)C(=O)O | alpha | 0.3392 | 209.201 | TRUE | TRUE |
| CHEMBL4239335 | CC(=O)c1cccc(C[C@H](N)C(=O)O)c1 | alpha | 0.8436 | 207.229 | TRUE | TRUE |
| CHEMBL424016 | N[C@@H](C[C@H](Cc1ccccc1)C(=O)O)C(=O)O | alpha | 0.7319 | 237.255 | TRUE | TRUE |
| CHEMBL4240267 | N[C@@H](Cc1cn(Cc2ccccc2)cn1)C(=O)O | alpha | 0.8858 | 245.282 | TRUE | TRUE |
| CHEMBL4240451 | CNC(=O)c1cccc(C[C@H](N)C(=O)O)c1 | alpha | 0.0006 | 222.244 | TRUE | TRUE |
| CHEMBL4241715 | N[C@@H](Cc1cccc(CC(=O)O)c1)C(=O)O | alpha | 0.2681 | 223.228 | TRUE | TRUE |
| CHEMBL4242544 | Cl.NC(Cc1cccc(C(=O)NCCc2ccc(O)c(O)c2)c1)C(=O)O | alpha | 1.4465 | 380.828 | TRUE | FALSE |
| CHEMBL4243343 | N[C@@H](Cc1ccccn1)C(=O)O | alpha | 0.036 | 166.18 | TRUE | TRUE |
| CHEMBL4244201 | NCc1cccc(C[C@H](N)C(=O)O)c1 | alpha | 0.0997 | 194.234 | TRUE | TRUE |
| CHEMBL4244287 | N[C@@H](CSCCB(O)O)C(=O)O | alpha | -1.3957 | 193.033 | FALSE | TRUE |
| CHEMBL4244654 | N[C@@H](CCC(=O)Nc1ccc(O)c(C(=O)O)c1)C(=O)O | alpha | 0.221 | 282.252 | TRUE | TRUE |
| CHEMBL424568 | O=C(O)C(CS)Nc1ccccc1 | alpha | 1.4815 | 197.259 | TRUE | FALSE |
| CHEMBL4247184 | Nc1cccc(C[C@H](N)C(=O)O)c1 | alpha | 0.2232 | 180.207 | TRUE | TRUE |
| CHEMBL4247994 | N[C@H](Cc1cccc(C(=O)O)c1)C(=O)O | alpha | 0.3392 | 209.201 | TRUE | TRUE |
| CHEMBL424821 | C/C=C/[C@@H]1C[C@H](C(=O)O)N[C@H]1[C@H](CC(C)C)NC(C)=O | alpha | 1.5446 | 282.384 | FALSE | TRUE |
| CHEMBL424838 | Cc1cc(-c2ccccc2)ccc1NC(=O)CC(N)C(=O)O | alpha | 2.40252 | 298.342 | TRUE | FALSE |
| CHEMBL4248453 | CC(=O)Nc1cccc(C[C@H](N)C(=O)O)c1 | alpha | 0.5994 | 222.244 | TRUE | TRUE |
| CHEMBL4248655 | Cc1cc(C[C@H](N)C(=O)O)cc(C(=O)O)c1 | alpha | 0.64762 | 223.228 | TRUE | TRUE |
| CHEMBL42499 | O=C(O)[C@@H]1c2ccc(Cl)cc2N[C@H]1C(=O)O | alpha | 1.3869 | 241.63 | TRUE | TRUE |
| CHEMBL4251478 | N[C@@H](Cc1cccc(CO)c1)C(=O)O | alpha | 0.1333 | 195.218 | TRUE | TRUE |
| CHEMBL425610 | NC(CCSSCCC(=O)O)C(=O)O | alpha | 0.6445 | 239.318 | FALSE | FALSE |
| CHEMBL42604 | NC(Cc1oc2ccccc2c1CP(=O)(O)O)C(=O)O | alpha | 1.0649 | 299.219 | TRUE | FALSE |
| CHEMBL426267 | CCC/C(=C\[C@@H](N)C(=O)O)CP(=O)(O)O | alpha | 0.3025 | 237.192 | FALSE | TRUE |
| CHEMBL426810 | CC(=O)N[C@@H](CC(C)C)[C@@H]1N[C@@H](C(=O)O)C[C@H]1c1cscn1 | alpha | 1.5926 | 325.434 | TRUE | TRUE |
| CHEMBL4277216 | CSCCC(NS(=O)(=O)c1ccc2[nH]c(=O)oc2c1)C(=O)O | alpha | 0.6057 | 346.386 | TRUE | FALSE |
| CHEMBL428337 | C/C(=C\C(N)C(=O)O)CC(N)C(=O)O | alpha | -0.8534 | 202.21 | FALSE | FALSE |
| CHEMBL42885 | O=C(O)COc1noc2c1CC(C(=O)O)NC2 | alpha | -0.7631 | 242.187 | TRUE | FALSE |
| CHEMBL4297204 | NCCC[C@H](N)C(=O)O.O=C(O)Cc1ccccc1 | alpha | 0.4509 | 268.313 | TRUE | TRUE |
| CHEMBL4297357 | N[C@H](Cc1ccc(OC[18F])cc1)C(=O)O | alpha | 0.9468 | 212.210938 | TRUE | TRUE |
| CHEMBL4297406 | CC(=O)N[C@@H](CSSC[C@H](N)C(=O)O)C(=O)O | alpha | -0.631 | 282.343 | FALSE | TRUE |
| CHEMBL429806 | COc1cc(C2NC(C(=O)O)CS2)ccc1O | alpha | 1.189 | 255.295 | TRUE | FALSE |
| CHEMBL4301257 | NC(Cc1cccc(C(=O)NCCc2ccc(O)c(O)c2)c1)C(=O)O | alpha | 1.0247 | 344.367 | TRUE | FALSE |
| CHEMBL4302826 | N[C@H](Cc1ccc(OCF)cc1)C(=O)O | alpha | 0.9468 | 213.208 | TRUE | TRUE |
| CHEMBL4303391 | N#[N+]/C=C(\[O-])CC[C@H](N)C(=O)O | alpha | -0.76672 | 171.156 | FALSE | TRUE |
| CHEMBL4303469 | O=C(O)C(Cc1ccccc1)NCc1ccc(Cl)cc1 | alpha | 3.1255 | 289.762 | TRUE | FALSE |
| CHEMBL430460 | CS/C(S)=N/CCC[C@H](N)C(=O)O | alpha | 0.8273 | 222.335 | FALSE | TRUE |
| CHEMBL431029 | N[C@H](CC(=O)CP(=O)(O)O)C(=O)O | alpha | -1.4648 | 211.11 | FALSE | TRUE |
| CHEMBL43113 | Cc1ccc(-c2ccc(S(=O)(=O)N[C@H](C(=O)O)C(C)C)cc2)cc1 | alpha | 3.04952 | 347.436 | TRUE | TRUE |
| CHEMBL43131 | O=C(O)[C@H]1C[C@H](C(=O)O)N1 | alpha | -1.1139 | 145.114 | FALSE | TRUE |
| CHEMBL431311 | O=C(O)[C@@H]1C[C@H](/C=C/CP(=O)(O)O)CCN1 | alpha | 0.1731 | 249.203 | FALSE | TRUE |
| CHEMBL43147 | CC(C)[C@H](NS(=O)(=O)c1ccc(-c2ccccc2)cc1)C(=O)O | alpha | 2.7411 | 333.409 | TRUE | TRUE |
| CHEMBL431504 | O=C(O)[C@H]1NCCC[C@H]1C(=O)O | alpha | -0.4762 | 173.168 | FALSE | TRUE |
| CHEMBL431657 | C=C(c1cccc(OCCCC)c1)[C@H]1CN[C@H](C(=O)O)[C@H]1CC(=O)O | alpha | 2.6422 | 347.411 | TRUE | TRUE |
| CHEMBL431839 | C=C(c1ccc(Cl)cc1)[C@H]1CN[C@H](C(=O)O)[C@H]1CC(=O)O | alpha | 2.1167 | 309.749 | TRUE | TRUE |
| CHEMBL431848 | Cc1ccc(Oc2ccccc2S(=O)(=O)N[C@H](C=O)C(=O)O)cc1 | alpha | 1.71772 | 349.364 | TRUE | TRUE |
| CHEMBL431929 | NC(Cc1cn(Cc2ccccc2)cn1)C(=O)O | alpha | 0.8858 | 245.282 | TRUE | FALSE |
| CHEMBL433077 | CC(C)c1nc(CC(N)C(=O)O)cn1C | alpha | 0.4979 | 211.265 | TRUE | FALSE |
| CHEMBL433333 | N[C@H](C(=O)O)[C@@H]1[C@@H](COCc2ccccc2)[C@@H]1C(=O)O | alpha | 0.5619 | 279.292 | TRUE | TRUE |
| CHEMBL433998 | N[C@@H](CSC(c1ccccc1)c1ccccc1)C(=O)O | alpha | 2.9211 | 287.384 | TRUE | TRUE |
| CHEMBL434118 | CCCOc1noc(C(=O)O)c1CC(N)C(=O)O | alpha | 0.116 | 258.23 | TRUE | FALSE |
| CHEMBL434119 | N[C@@H](C(=O)O)[C@H](CC(=O)O)c1ccc(Cl)cc1 | alpha | 1.3102 | 257.673 | TRUE | TRUE |
| CHEMBL434515 | CCOc1noc(-c2nn[nH]n2)c1CC(N)C(=O)O | alpha | -0.7922 | 268.233 | TRUE | FALSE |
| CHEMBL43480 | CCC/C(=C\C(N)C(=O)O)CP(=O)(O)O | alpha | 0.3025 | 237.192 | FALSE | FALSE |
| CHEMBL434994 | Cc1ccc(C(N)C(=O)O)cc1 | alpha | 1.07942 | 165.192 | TRUE | FALSE |
| CHEMBL435131 | CC(C)[C@@H](NS(=O)(=O)c1ccc(-c2ccccc2O)cc1)C(=O)O | alpha | 2.4467 | 349.408 | TRUE | TRUE |
| CHEMBL43528 | O=C(O)[C@@H]1C[C@@H](C(=O)O)N1 | alpha | -1.1139 | 145.114 | FALSE | TRUE |
| CHEMBL435854 | CC(=N)NCCSC[C@@H](N)C(=O)O | alpha | -0.28173 | 205.283 | FALSE | TRUE |
| CHEMBL435890 | CC/N=C(\N)NCC[C@H](N)C(=O)O | alpha | -1.2874 | 188.231 | FALSE | TRUE |
| CHEMBL435945 | NC(C(=O)O)c1ccc(CP(=O)(O)O)s1 | alpha | 0.5102 | 251.2 | TRUE | FALSE |
| CHEMBL436148 | O=C(O)C1CN(CCP(=O)(O)O)CCN1 | alpha | -1.4775 | 238.18 | FALSE | FALSE |
| CHEMBL436579 | N#Cc1ccc(NC(=O)C(N)C(=O)O)cc1 | alpha | -0.09132 | 219.2 | TRUE | FALSE |
| CHEMBL437169 | NC(C(=O)O)c1cnn(O)c1CCCc1ccccc1 | alpha | 1.3801 | 275.308 | TRUE | FALSE |
| CHEMBL437383 | O=C(O)C1=NO[C@@]2(CN[C@@H](C(=O)O)C2)C1 | alpha | -0.9674 | 214.177 | FALSE | TRUE |
| CHEMBL43839 | NC(C(=O)O)C1CC1CP(=O)(O)O | alpha | -0.7879 | 209.138 | FALSE | FALSE |
| CHEMBL438441 | N[C@@H](CCCCNC(=O)CCCS)C(=O)O | alpha | 0.3948 | 248.348 | FALSE | TRUE |
| CHEMBL43889 | CC(C)c1ncc(CC(N)C(=O)O)n1Cc1ccccc1 | alpha | 2.0092 | 287.363 | TRUE | FALSE |
| CHEMBL43890 | Cn1c(CC(N)C(=O)O)cnc1C(C)(C)C | alpha | 0.672 | 225.292 | TRUE | FALSE |
| CHEMBL43899 | Cc1cc2cc(CP(=O)(O)O)c(C[C@H](N)C(=O)O)nc2cc1C | alpha | 1.48374 | 338.3 | TRUE | TRUE |
| CHEMBL440855 | O=C(O)[C@@H]1CCN[C@H]1C(=O)O | alpha | -0.8663 | 159.141 | FALSE | TRUE |
| CHEMBL44139 | CCC[C@H](N[C@H]1CCc2ccccc2N(CC(=O)O)C1=O)C(=O)O | alpha | 1.2619 | 334.372 | TRUE | TRUE |
| CHEMBL441460 | Cn1cnc(-c2onc(O)c2CC(N)C(=O)O)n1 | alpha | -0.8699 | 253.218 | TRUE | FALSE |
| CHEMBL441601 | Cc1ccc(-c2ccc(C[C@H](NCP(=O)(O)O)C(=O)O)cc2)cc1 | alpha | 2.38252 | 349.323 | TRUE | TRUE |
| CHEMBL442076 | NC(C(=O)O)[C@H]1C[C@@H]1P(=O)(O)O | alpha | -1.0355 | 195.111 | FALSE | TRUE |
| CHEMBL442149 | O=C(O)C1CN(Cc2cccc(P(=O)(O)O)c2)CCN1 | alpha | -0.652 | 300.251 | TRUE | FALSE |
| CHEMBL442342 | O=C(O)C1NCCc2c1cccc2P(=O)(O)O | alpha | -0.239 | 257.182 | TRUE | FALSE |
| CHEMBL442347 | N[C@H](C(=O)O)c1cccc(O)c1 | alpha | 0.4766 | 167.164 | TRUE | TRUE |
| CHEMBL442543 | N[C@H](Cc1nc2cc(Cl)ccc2cc1CP(=O)(O)O)C(=O)O | alpha | 1.5203 | 344.691 | TRUE | TRUE |
| CHEMBL442607 | N#[N+]/C=C(\[O-])OC[C@H](N)C(=O)O | alpha | -1.57282 | 173.128 | FALSE | TRUE |
| CHEMBL442625 | CC(C)(CCSCC[C@@H](N)C(=O)O)CC(=O)O | alpha | 1.4126 | 263.359 | FALSE | TRUE |
| CHEMBL442638 | C[C@@H](NCCSCCC(N)C(=O)O)C(=O)O | alpha | -0.4157 | 250.32 | FALSE | TRUE |
| CHEMBL44270 | NC(C(=O)O)C1CC1CCCP(=O)(O)O | alpha | -0.0077 | 237.192 | FALSE | FALSE |
| CHEMBL442712 | N[C@@H](C[C@H](CCc1ccccc1)C(=O)O)C(=O)O | alpha | 1.122 | 251.282 | TRUE | TRUE |
| CHEMBL442816 | N[C@@H](CCC(=O)Nc1ccc(Oc2ccccc2)cc1)C(=O)O | alpha | 2.6095 | 314.341 | TRUE | TRUE |
| CHEMBL442857 | NC(CCOCCCCC(=O)O)C(=O)O | alpha | 0.0599 | 219.237 | FALSE | FALSE |
| CHEMBL442916 | N=C(NCCC[C@H](N)C(=O)O)N1CCOCC1 | alpha | -0.96493 | 244.295 | FALSE | TRUE |
| CHEMBL443372 | CC(C)(CCSCCC(N)C(=O)O)CC(=O)O | alpha | 1.4126 | 263.359 | FALSE | FALSE |
| CHEMBL44348 | Cc1cc2cc(CP(=O)(O)O)c(C[C@@H](N)C(=O)O)nc2cc1C | alpha | 1.48374 | 338.3 | TRUE | TRUE |
| CHEMBL443673 | N[C@H](C(=O)O)C(Cc1ccccc1)C(=O)O | alpha | 0.3418 | 223.228 | TRUE | TRUE |
| CHEMBL4437345 | CC(C)[C@H](NCc1nnnn1CCCc1ccccc1)C(=O)O | alpha | 1.5047 | 317.393 | TRUE | TRUE |
| CHEMBL443916 | NC(CCCCCCCC(=O)O)C(=O)O | alpha | 1.2136 | 217.265 | FALSE | FALSE |
| CHEMBL4439195 | C=C(C)[C@]1(O)CN[C@H](C(=O)O)[C@H]1CC(=O)O | alpha | -0.5591 | 229.232 | FALSE | TRUE |
| CHEMBL443927 | N[C@H](C(=O)O)C(Cc1c(Cl)cccc1Cl)C(=O)O | alpha | 1.6486 | 292.118 | TRUE | TRUE |
| CHEMBL444101 | NC(C(=O)O)c1cnn(O)c1Cc1ccccc1 | alpha | 0.7956 | 247.254 | TRUE | FALSE |
| CHEMBL444207 | C[C@H](NCCC1OCC(C)(C)CO1)C(=O)O | alpha | 0.8383 | 231.292 | FALSE | TRUE |
| CHEMBL4442120 | COCC(=N)NCCC[C@H](N)C(=O)O | alpha | -0.60823 | 203.242 | FALSE | TRUE |
| CHEMBL4442624 | N[C@@H](CSCc1ccc(F)cc1)C(=O)O | alpha | 1.4708 | 229.276 | TRUE | TRUE |
| CHEMBL444323 | NC(CC1CCCC(CP(=O)(O)O)C1)C(=O)O | alpha | 0.7725 | 265.246 | FALSE | FALSE |
| CHEMBL4444367 | Cn1nc(O)c(C(N)C(=O)O)n1 | alpha | -1.3949 | 172.144 | TRUE | FALSE |
| CHEMBL444589 | NC(C(=O)O)c1ccc(C(=O)O)c(O)c1 | alpha | 0.1748 | 211.173 | TRUE | FALSE |
| CHEMBL444812 | N[C@@H](Cc1ccccc1CN(CCCl)CCCl)C(=O)O | alpha | 1.9206 | 319.232 | TRUE | TRUE |
| CHEMBL4448290 | CCn1nc(O)c(CC(N)C(=O)O)n1 | alpha | -1.042 | 200.198 | TRUE | FALSE |
| CHEMBL4448346 | N[C@@H](Cn1cc(-c2ccccc2)nn1)C(=O)O | alpha | 0.357 | 232.243 | TRUE | TRUE |
| CHEMBL444863 | COc1cccc(C[C@@H](C[C@@H](N)C(=O)O)C(=O)O)c1 | alpha | 0.7405 | 267.281 | TRUE | TRUE |
| CHEMBL4449638 | COCCC(=N)NCCC[C@H](N)C(=O)O | alpha | -0.21813 | 217.269 | FALSE | TRUE |
| CHEMBL444997 | CC[C@H](C)[C@H](NCCC1OCC(C)(C)CO1)C(=O)O | alpha | 1.8645 | 273.373 | FALSE | TRUE |
| CHEMBL4451018 | CCCn1nc(O)c(CC(N)C(=O)O)n1 | alpha | -0.6519 | 214.225 | TRUE | FALSE |
| CHEMBL445363 | NC(CCCC(NO)C(=O)O)C(=O)O | alpha | -0.9994 | 206.198 | FALSE | FALSE |
| CHEMBL4456101 | CCCCCCCCCCC[C@@H]1CCN[C@H](C(=O)O)C1 | alpha | 4.3601 | 283.456 | FALSE | TRUE |
| CHEMBL4457506 | N[C@@H](Cn1cc(Cc2ccccc2)nn1)C(=O)O | alpha | 0.2808 | 246.27 | TRUE | TRUE |
| CHEMBL446023 | N[C@H](C(=O)O)C(Cc1ccc(F)cc1)C(=O)O | alpha | 0.4809 | 241.218 | TRUE | TRUE |
| CHEMBL4461206 | N=C(CCl)NCCC[C@H](N)C(=O)O | alpha | -0.01583 | 207.661 | FALSE | TRUE |
| CHEMBL446256 | N[C@@H](C[C@@H](COCc1ccccc1)C(=O)O)C(=O)O | alpha | 0.706 | 267.281 | TRUE | TRUE |
| CHEMBL4464816 | N[C@@H](Cn1ccnn1)C(=O)O | alpha | -1.31 | 156.145 | TRUE | TRUE |
| CHEMBL446749 | CCc1cnn(O)c1[C@@H](N)C(=O)O | alpha | -0.2328 | 185.183 | TRUE | TRUE |
| CHEMBL446907 | N[C@H](C(=O)O)C(Cc1cccc(Br)c1)C(=O)O | alpha | 1.1043 | 302.124 | TRUE | TRUE |
| CHEMBL4470206 | C=C(C)[C@@]1(O)CN[C@H](C(=O)O)[C@H]1CC(=O)O | alpha | -0.5591 | 229.232 | FALSE | TRUE |
| CHEMBL447260 | CN(O)CC[C@H](N)C(=O)O | alpha | -0.8906 | 148.162 | FALSE | TRUE |
| CHEMBL447489 | N[C@@H](CCCCCCc1cnc[nH]1)C(=O)O | alpha | 1.3146 | 225.292 | TRUE | TRUE |
| CHEMBL447691 | O=C(O)[C@@H]1CS[C@H]([C@H](C(=O)O)c2ccccc2)N1 | alpha | 0.9705 | 267.306 | TRUE | TRUE |
| CHEMBL448098 | O=C(O)[C@H](CC#Cc1ccccc1F)NCP(=O)(O)O | alpha | 0.7453 | 301.21 | TRUE | TRUE |
| CHEMBL448138 | NC(CCSCCNCCC(=O)O)C(=O)O | alpha | -0.4141 | 250.32 | FALSE | FALSE |
| CHEMBL448512 | CC1(C)COC(CCN[C@@H](CO)C(=O)O)OC1 | alpha | -0.1893 | 247.291 | FALSE | TRUE |
| CHEMBL448548 | NC(CC[Se]CCCCC(=O)O)C(=O)O | alpha | 0.5841 | 282.198 | FALSE | FALSE |
| CHEMBL448691 | NC(CCSCSCCC(=O)O)C(=O)O | alpha | 0.687 | 253.345 | FALSE | FALSE |
| CHEMBL448976 | N[C@H](C(=O)O)C(Cc1ccc([N+](=O)[O-])cc1)C(=O)O | alpha | 0.25 | 268.225 | TRUE | TRUE |
| CHEMBL449175 | CSc1cccc(N[C@@H](CO)C(=O)O)c1 | alpha | 1.2659 | 227.285 | TRUE | TRUE |
| CHEMBL449297 | Cc1ccccc1[C@H]1CC[C@](C(=O)O)([C@@H](O)[C@@H](N)C(=O)O)N1 | alpha | 0.01572 | 308.334 | TRUE | TRUE |
| CHEMBL449974 | N[C@@H](CCCc1cnc[nH]1)C(=O)O | alpha | 0.1443 | 183.211 | TRUE | TRUE |
| CHEMBL450160 | N[C@@H](CCCCCc1cncn1-c1ccccc1)C(=O)O | alpha | 2.3871 | 287.363 | TRUE | TRUE |
| CHEMBL450245 | CC(C)c1cnn(O)c1[C@H](N)C(=O)O | alpha | 0.3282 | 199.21 | TRUE | TRUE |
| CHEMBL450445 | O=C(O)/C1=N/CCCC[C@@H](C(=O)O)N1 | alpha | -0.3038 | 200.194 | FALSE | TRUE |
| CHEMBL450968 | Cc1cnn(O)c1[C@@H](N)C(=O)O | alpha | -0.48678 | 171.156 | TRUE | TRUE |
| CHEMBL451212 | C[N+](C)(CCCSCCC(N)C(=O)O)CC(=O)[O-] | alpha | -1.262 | 278.374 | FALSE | FALSE |
| CHEMBL45167 | NC(C(=O)O)C1CC1CCP(=O)(O)O | alpha | -0.3978 | 223.165 | FALSE | FALSE |
| CHEMBL4516756 | NC(Cc1ccccc1I)C(=O)O.O=C(O)C(F)(F)F | alpha | 1.8789 | 405.11 | TRUE | FALSE |
| CHEMBL45170 | NC(Cc1c[nH]c(C2CCC2)n1)C(=O)O | alpha | 0.6316 | 209.249 | TRUE | FALSE |
| CHEMBL45171 | CC(C)(C)c1nc(CC(N)C(=O)O)c[nH]1 | alpha | 0.6616 | 211.265 | TRUE | FALSE |
| CHEMBL451754 | CC1(C)COC(CCN[C@@H](CC(=O)O)C(=O)O)OC1 | alpha | 0.2931 | 275.301 | FALSE | TRUE |
| CHEMBL451755 | CC1(C)COC(CCN[C@@H](CCC(=O)O)C(=O)O)OC1 | alpha | 0.6832 | 289.328 | FALSE | TRUE |
| CHEMBL451995 | O=C(O)CN[C@H](Cc1c[nH]c2ccccc12)C(=O)O | alpha | 0.8378 | 262.265 | TRUE | TRUE |
| CHEMBL452017 | CSCC[C@H](NCCC1OCC(C)(C)CO1)C(=O)O | alpha | 1.5715 | 291.413 | FALSE | TRUE |
| CHEMBL45225 | CC(C)c1nc(CC(N)C(=O)O)c[nH]1 | alpha | 0.4875 | 197.238 | TRUE | FALSE |
| CHEMBL452262 | CC1(C)COC(CCN[C@@H](Cc2ccccc2)C(=O)O)OC1 | alpha | 2.0611 | 307.39 | TRUE | TRUE |
| CHEMBL452264 | CC1(C)COC(CCN[C@@H](Cc2ccc(O)cc2)C(=O)O)OC1 | alpha | 1.7667 | 323.389 | TRUE | TRUE |
| CHEMBL452265 | C[C@@H](O)[C@H](NCCC1OCC(C)(C)CO1)C(=O)O | alpha | 0.1992 | 261.318 | FALSE | TRUE |
| CHEMBL4525720 | COc1cc2nccc(SC[C@H](N)C(=O)O)c2cc1C(N)=O | alpha | 0.8463 | 321.358 | TRUE | TRUE |
| CHEMBL452711 | N[C@@H](CCNO)C(=O)O | alpha | -1.2328 | 134.135 | FALSE | TRUE |
| CHEMBL452971 | N[C@H](C(=O)O)c1c(Cl)cnn1O | alpha | -0.1418 | 191.574 | TRUE | TRUE |
| CHEMBL453189 | CC[C@H](C)[C@H](NS(=O)(=O)c1ccc(Cl)cc1)C(=O)O | alpha | 2.1176 | 305.783 | TRUE | TRUE |
| CHEMBL45324 | CC(C)n1cnc(CC(N)C(=O)O)c1 | alpha | 0.4184 | 197.238 | TRUE | FALSE |
| CHEMBL453265 | N=C(NCCC[C@H](N)C(=O)O)NCC(F)(F)F | alpha | -0.14523 | 256.228 | FALSE | TRUE |
| CHEMBL4537472 | COCCCC(=N)NCCC[C@H](N)C(=O)O | alpha | 0.17197 | 231.296 | FALSE | TRUE |
| CHEMBL4539393 | NC(Cc1ccccc1F)C(=O)O.O=C(O)C(F)(F)F | alpha | 1.4134 | 297.204 | TRUE | FALSE |
| CHEMBL45399 | N[C@H](C[C@@H]1CC1P(=O)(O)O)C(=O)O | alpha | -0.6454 | 209.138 | FALSE | TRUE |
| CHEMBL454079 | CC(C)[C@H](NCCC1OCC(C)(C)CO1)C(=O)O | alpha | 1.4744 | 259.346 | FALSE | TRUE |
| CHEMBL454080 | CC(C)C[C@H](NCCC1OCC(C)(C)CO1)C(=O)O | alpha | 1.8645 | 273.373 | FALSE | TRUE |
| CHEMBL4540850 | C=CCCSC[C@H](N)C(=O)O | alpha | 0.7076 | 175.253 | FALSE | TRUE |
| CHEMBL4540977 | O=C(O)CN[C@H](Cc1ccccc1)C(=O)O | alpha | 0.3565 | 223.228 | TRUE | TRUE |
| CHEMBL454191 | CCOP(=O)(OCC)c1cccc(N[C@H](CO)C(=O)O)c1 | alpha | 1.4354 | 317.278 | TRUE | TRUE |
| CHEMBL4543592 | COc1ccc(CN[C@H](C(=O)O)C(C)C)cc1-c1cccc(Cl)c1 | alpha | 4.2144 | 347.842 | TRUE | TRUE |
| CHEMBL45453 | CCc1nc(CC(N)C(=O)O)c[nH]1 | alpha | -0.0735 | 183.211 | TRUE | FALSE |
| CHEMBL4547433 | CC(C)[C@H](NCc1nnnn1Cc1ccccc1)C(=O)O | alpha | 0.9202 | 289.339 | TRUE | TRUE |
| CHEMBL4548192 | CC(C)=CCN[C@@H](CCC(=O)O)C(=O)O | alpha | 0.8602 | 215.249 | FALSE | TRUE |
| CHEMBL455117 | CC1(C)COC(CCN[C@@H](CC(N)=O)C(=O)O)OC1 | alpha | -0.3062 | 274.317 | FALSE | TRUE |
| CHEMBL455118 | CC1(C)COC(CCN[C@@H](CCC(N)=O)C(=O)O)OC1 | alpha | 0.0839 | 288.344 | FALSE | TRUE |
| CHEMBL455219 | N[C@H](C(=O)O)C(Cc1cccc(F)c1)C(=O)O | alpha | 0.4809 | 241.218 | TRUE | TRUE |
| CHEMBL45530 | CSC(C)C[C@@H](N)C(=O)O | alpha | 0.5399 | 163.242 | FALSE | TRUE |
| CHEMBL455377 | CC1(C)COC(CCN[C@@H](Cc2c[nH]cn2)C(=O)O)OC1 | alpha | 0.7842 | 297.355 | TRUE | TRUE |
| CHEMBL455378 | CC1(C)COC(CCN[C@@H](CCCCN)C(=O)O)OC1 | alpha | 0.9474 | 288.388 | FALSE | TRUE |
| CHEMBL4554017 | O=C(O)C1CCCCNN1 | alpha | -0.2823 | 144.174 | FALSE | FALSE |
| CHEMBL4556711 | CCCn1nnc(O)c1CC(N)C(=O)O | alpha | -0.6519 | 214.225 | TRUE | FALSE |
| CHEMBL455841 | NC(CCSCCNCC(=O)O)C(=O)O | alpha | -0.8042 | 236.293 | FALSE | FALSE |
| CHEMBL4559029 | N[C@@H](Cn1nnc2ccccc21)C(=O)O | alpha | -0.1568 | 206.205 | TRUE | TRUE |
| CHEMBL4561071 | Cc1ncc(Cn2cc(CC(N)C(=O)O)nn2)c(N)n1.Cl | alpha | -0.61678 | 313.749 | TRUE | FALSE |
| CHEMBL456540 | N=C(NCCC[C@H](N)C(=O)O)NCCC(N)=O | alpha | -1.83213 | 245.283 | FALSE | TRUE |
| CHEMBL456882 | C/C=C/CC(=N)NCCC[C@H](N)C(=O)O | alpha | 0.71157 | 213.281 | FALSE | TRUE |
| CHEMBL4569744 | N[C@@H](Cn1cc(CO)nn1)C(=O)O | alpha | -1.8177 | 186.171 | TRUE | TRUE |
| CHEMBL4570233 | N[C@@H](Cc1cn(Cc2ccccc2)nn1)C(=O)O | alpha | 0.2808 | 246.27 | TRUE | TRUE |
| CHEMBL4572199 | CCc1sc2ncnc(N[C@@H](C)C(=O)O)c2c1CC | alpha | 2.7011 | 279.365 | TRUE | TRUE |
| CHEMBL4572915 | COCCCC(=N)NCC[C@H](N)C(=O)O | alpha | -0.21813 | 217.269 | FALSE | TRUE |
| CHEMBL457411 | N[C@H](C(=O)O)[C@H](Cc1cccc(F)c1)C(=O)O | alpha | 0.4809 | 241.218 | TRUE | TRUE |
| CHEMBL457412 | N[C@H](C(=O)O)[C@@H](Cc1cccc(F)c1)C(=O)O | alpha | 0.4809 | 241.218 | TRUE | TRUE |
| CHEMBL457413 | N[C@H](C(=O)O)C(Cc1ccc2ccccc2c1)C(=O)O | alpha | 1.495 | 273.288 | TRUE | TRUE |
| CHEMBL457631 | Cc1cccc(CC(C(=O)O)[C@H](N)C(=O)O)c1 | alpha | 0.65022 | 237.255 | TRUE | TRUE |
| CHEMBL4578102 | CC(C)=CCN[C@H](C(=O)O)[C@H](O)CC(=O)O | alpha | -0.169 | 231.248 | FALSE | TRUE |
| CHEMBL4578941 | O=C(O)C1CSC(c2ccccc2)N1 | alpha | 1.4748 | 209.27 | TRUE | FALSE |
| CHEMBL458081 | C/C=C/CC[S+]([O-])C[C@@H](N)C(=O)O | alpha | 0.1132 | 205.279 | FALSE | TRUE |
| CHEMBL458117 | N[C@@H](C[C@H]1C[C@]1(C(=O)O)c1ccccc1)C(=O)O | alpha | 0.8309 | 249.266 | TRUE | TRUE |
| CHEMBL458273 | N[C@H](C(=O)O)[C@H](Cc1ccccc1)C(=O)O | alpha | 0.3418 | 223.228 | TRUE | TRUE |
| CHEMBL458352 | Cl.N[C@@H](CCCN/C=C1\C(=O)Nc2ccccc21)C(=O)O | alpha | 1.1831 | 311.769 | TRUE | TRUE |
| CHEMBL458440 | Cl.N[C@@H](CCCCN/C=C1\C(=O)Nc2ccccc21)C(=O)O | alpha | 1.5732 | 325.796 | TRUE | TRUE |
| CHEMBL4586342 | Cc1cn(C[C@H](N)C(=O)O)nn1 | alpha | -1.00158 | 170.172 | TRUE | TRUE |
| CHEMBL4587150 | C=CCCCSC[C@H](N)C(=O)O | alpha | 1.0977 | 189.28 | FALSE | TRUE |
| CHEMBL4587818 | Cn1cc(C[C@H](N)C(=O)O)nn1 | alpha | -1.2305 | 170.172 | TRUE | TRUE |
| CHEMBL4594943 | Cc1ncc(Cn2cc(CC(N)C(=O)O)nn2)c(N)n1 | alpha | -1.03858 | 277.288 | TRUE | FALSE |
| CHEMBL4595513 | NC(Cc1ccccc1I)C(=O)O | alpha | 1.2456 | 291.088 | TRUE | FALSE |
| CHEMBL4597435 | NC(Cc1ccccc1F)C(=O)O | alpha | 0.7801 | 183.182 | TRUE | FALSE |
| CHEMBL461166 | NC(Cc1cccc(N(CCCl)CCCl)c1)C(=O)O | alpha | 1.925 | 305.205 | TRUE | FALSE |
| CHEMBL46118 | CSC[C@@H](C)[C@@H](N)C(=O)O | alpha | 0.3974 | 163.242 | FALSE | TRUE |
| CHEMBL461845 | N[C@@H](CCCCP(=O)(O)O)C(=O)O | alpha | -0.2537 | 211.154 | FALSE | TRUE |
| CHEMBL461871 | NC(CCc1ccc(N(CCCl)CCCl)cc1)C(=O)O | alpha | 2.3151 | 319.232 | TRUE | FALSE |
| CHEMBL46228 | Cn1cc(CC(N)C(=O)O)nc1C1CCCCC1 | alpha | 1.4222 | 251.33 | TRUE | FALSE |
| CHEMBL462421 | NC(CCSSCCCC(=O)O)C(=O)O | alpha | 1.0346 | 253.345 | FALSE | FALSE |
| CHEMBL4633056 | N[C@@H](CF)C(=O)O | alpha | -0.6322 | 107.084 | FALSE | TRUE |
| CHEMBL4633276 | N[C@H](Cc1ccc(Cl)cc1Cl)C(=O)O | alpha | 1.9478 | 234.082 | TRUE | TRUE |
| CHEMBL4635699 | N[C@H](C(=O)O)C(F)F | alpha | -0.3366 | 125.074 | FALSE | TRUE |
| CHEMBL4639725 | CCCCCCCNC(CCC1CCCC1)C(=O)O | alpha | 3.97 | 269.429 | FALSE | FALSE |
| CHEMBL4640591 | N[C@@H](C(=O)O)C(F)F | alpha | -0.3366 | 125.074 | FALSE | TRUE |
| CHEMBL464167 | C[S+]([O-])C[C@H](N)C(=O)O | alpha | -1.2232 | 151.187 | FALSE | TRUE |
| CHEMBL4642872 | C/C(=C\C(=O)N(O)CCC[C@@H](N)C(=O)O)CCO | alpha | -0.275 | 260.29 | FALSE | TRUE |
| CHEMBL464373 | C/C=C/[S+]([O-])C[C@H](N)C(=O)O | alpha | -0.3194 | 177.225 | FALSE | TRUE |
| CHEMBL4647106 | COc1ccc([C@H](O)[C@H](N)C(=O)O)cc1OC | alpha | 0.1491 | 241.243 | TRUE | TRUE |
| CHEMBL4649276 | N[C@@H](Cc1c[nH]c2cc(C(F)F)ccc12)C(=O)O | alpha | 2.0599 | 254.236 | TRUE | TRUE |
| CHEMBL465458 | N=C(NCCC[C@H](N)C(=O)O)N1CC=CC1 | alpha | -0.42533 | 226.28 | FALSE | TRUE |
| CHEMBL466242 | C[N+](C)(CSCCC(N)C(=O)O)CC(=O)[O-] | alpha | -1.6946 | 250.32 | FALSE | FALSE |
| CHEMBL46713 | N[C@H](Cc1nc2ncccc2cc1CP(=O)(O)O)C(=O)O | alpha | 0.2619 | 311.234 | TRUE | TRUE |
| CHEMBL46859 | Cc1onc(OCC(=O)O)c1C(N)C(=O)O | alpha | -0.46918 | 230.176 | TRUE | FALSE |
| CHEMBL469269 | N[C@@H](CCCCCc1cnc[nH]1)C(=O)O | alpha | 0.9245 | 211.265 | TRUE | TRUE |
| CHEMBL469866 | N[C@@H](CCSN=O)C(=O)O | alpha | 0.203 | 164.186 | FALSE | TRUE |
| CHEMBL47063 | CS[C@@H](C)C[C@@H](N)C(=O)O | alpha | 0.5399 | 163.242 | FALSE | TRUE |
| CHEMBL472562 | N[C@H](C(=O)O)c1ccc(CO)c(O)c1 | alpha | -0.0311 | 197.19 | TRUE | TRUE |
| CHEMBL472693 | N[C@@H](Cc1cc(N(CCCl)CCCl)ccc1F)C(=O)O | alpha | 2.0641 | 323.195 | TRUE | TRUE |
| CHEMBL473232 | N[C@H](CSCC(=O)Nc1c[nH]c(=O)[nH]c1=O)C(=O)O | alpha | -1.8532 | 288.285 | TRUE | TRUE |
| CHEMBL473864 | Cc1cnn(O)c1[C@H](N)C(=O)O | alpha | -0.48678 | 171.156 | TRUE | TRUE |
| CHEMBL4744943 | COc1cc2c(cc1O)CCC(C)(CC(N)C(=O)O)O2 | alpha | 1.2865 | 281.308 | TRUE | FALSE |
| CHEMBL47458 | CC(C)c1nc(CC(N)C(=O)O)cn1Cc1ccccc1 | alpha | 2.0092 | 287.363 | TRUE | FALSE |
| CHEMBL4748950 | CC(C)(C)[C@H](N)C(=O)N[C@H]1CN[C@H](C(=O)O)[C@@H]1CCCB(O)O | alpha | -1.2298 | 329.206 | FALSE | TRUE |
| CHEMBL4751973 | N[C@H](C(=O)O)[C@@H]1C[C@]1(CO)C(=O)O | alpha | -1.5185 | 189.167 | FALSE | TRUE |
| CHEMBL4752242 | CCCC[C@@H](Nc1nc(N)nc2cccnc12)C(=O)O | alpha | 1.6623 | 275.312 | TRUE | TRUE |
| CHEMBL4754637 | N[C@H](C(=O)N[C@H]1CN[C@H](C(=O)O)[C@@H]1CCCB(O)O)C1CCCC1 | alpha | -1.0857 | 341.217 | FALSE | TRUE |
| CHEMBL4755350 | N[C@H](C(=O)O)[C@@H]1C[C@]1(CS)C(=O)O | alpha | -0.581 | 205.235 | FALSE | TRUE |
| CHEMBL4755761 | N[C@@H](CCC(=O)N[C@@H](Cc1c[nH]c2ccccc12)C(=O)O)C(=O)O | alpha | 0.4719 | 333.344 | TRUE | TRUE |
| CHEMBL4758283 | C=CCOC[C@]1(C(=O)O)C[C@H]1[C@H](N)C(=O)O | alpha | -0.3082 | 229.232 | FALSE | TRUE |
| CHEMBL4759097 | CC(C)n1c(CC(N)C(=O)O)cnc1Cc1ccccc1 | alpha | 2.0092 | 287.363 | TRUE | FALSE |
| CHEMBL4761641 | O=C(O)C[C@@H]1[C@@H](C(=O)O)NC[C@@H]1c1ccc(=O)[nH]c1 | alpha | -0.3942 | 266.253 | TRUE | TRUE |
| CHEMBL4762611 | NC[C@@]1(CCCB(O)O)CN[C@@H]1C(=O)O | alpha | -1.7591 | 216.046 | FALSE | TRUE |
| CHEMBL4763578 | N[C@H]1CN[C@H](C(=O)O)[C@@H]1CCCB(O)O | alpha | -1.7607 | 216.046 | FALSE | TRUE |
| CHEMBL476416 | N=C(N)NCC(F)(F)C[C@H](N)C(=O)O | alpha | -1.09323 | 210.184 | FALSE | TRUE |
| CHEMBL476417 | N/C(=N\O)NCC(F)(F)C[C@H](N)C(=O)O | alpha | -1.2828 | 226.183 | FALSE | TRUE |
| CHEMBL477278 | N[C@@H](Cc1ccc(OCC[18F])cc1)C(=O)O | alpha | 0.9893 | 226.237938 | TRUE | TRUE |
| CHEMBL47730 | CC(C)(C)c1nc(CC(N)C(=O)O)cn1Cc1ccccc1 | alpha | 2.1833 | 301.39 | TRUE | FALSE |
| CHEMBL4778978 | O=C(O)[C@H]1NC[C@H](O)[C@H]1CCCB(O)O | alpha | -1.7271 | 217.03 | FALSE | TRUE |
| CHEMBL4782098 | CC(C)(C)n1c(CC(N)C(=O)O)cnc1Cc1ccccc1 | alpha | 2.1833 | 301.39 | TRUE | FALSE |
| CHEMBL4782185 | CSCC[C@H](NCc1oc(CO)cc(=O)c1O)C(=O)O | alpha | 0.1336 | 303.336 | TRUE | TRUE |
| CHEMBL478993 | N[C@@H](CCC(=O)Nc1ccc(OCCc2ccccc2)cc1)C(=O)O | alpha | 2.4387 | 342.395 | TRUE | TRUE |
| CHEMBL4790569 | CC(C)[C@H](N[C@H](N)C(=O)Nc1csc(-c2ccccc2)n1)C(=O)O | alpha | 1.7322 | 348.428 | TRUE | TRUE |
| CHEMBL4791224 | CC(C)C[C@H](N)C(=O)N[C@H]1CN[C@H](C(=O)O)[C@@H]1CCCB(O)O | alpha | -1.2298 | 329.206 | FALSE | TRUE |
| CHEMBL4792263 | N[C@H](C(=O)O)[C@@H]1C[C@]1(COCc1ccccc1)C(=O)O | alpha | 0.706 | 279.292 | TRUE | TRUE |
| CHEMBL4792944 | CCCCC(NC/C(=C(/C)O)c1cc2c(c(=O)o1)CCCC2)C(=O)O | alpha | 3.0406 | 349.427 | TRUE | FALSE |
| CHEMBL4796146 | CC(C)n1cncc1CC(N)C(=O)O | alpha | 0.4184 | 197.238 | TRUE | FALSE |
| CHEMBL4796331 | [N-]=[N+]=NC[C@]1(C(=O)O)C[C@H]1[C@H](N)C(=O)O | alpha | -0.2005 | 214.181 | FALSE | TRUE |
| CHEMBL4797098 | Cc1ccc(S(=O)(=O)N[C@@H](CC(=O)NCC(C)C)C(=O)O)cc1 | alpha | 0.88882 | 342.417 | TRUE | TRUE |
| CHEMBL4797726 | N[C@H](CCC(=O)NCCS(=O)(=O)O)C(=O)O | alpha | -1.8175 | 254.264 | FALSE | TRUE |
| CHEMBL479804 | NC(=O)CC[C@H](C[C@H](N)C(=O)O)C(=O)O | alpha | -1.2453 | 218.209 | FALSE | TRUE |
| CHEMBL479805 | CCNC(=O)CC[C@H](C[C@H](N)C(=O)O)C(=O)O | alpha | -0.5945 | 246.263 | FALSE | TRUE |
| CHEMBL480206 | Cc1ccc([C@H]2CC[C@](C(=O)O)([C@@H](O)[C@@H](N)C(=O)O)N2)cc1 | alpha | 0.01572 | 308.334 | TRUE | TRUE |
| CHEMBL4802413 | CC(C)C[C@H](NP(O)(=S)c1ccccc1)C(=O)O | alpha | 1.7026 | 287.321 | TRUE | TRUE |
| CHEMBL4802719 | C[C@H](NP(O)(=S)c1ccccc1)C(=O)O | alpha | 0.6764 | 245.24 | TRUE | TRUE |
| CHEMBL4802780 | CC(C)[C@H](NP(O)(=S)c1ccccc1)C(=O)O | alpha | 1.3125 | 273.294 | TRUE | TRUE |
| CHEMBL4803043 | CC[C@H](C)[C@H](NP(O)(=S)c1ccccc1)C(=O)O | alpha | 1.7026 | 287.321 | TRUE | TRUE |
| CHEMBL4803162 | CSCC[C@H](NP(O)(=S)c1ccccc1)C(=O)O | alpha | 1.4096 | 305.361 | TRUE | TRUE |
| CHEMBL4803319 | O=C(O)[C@H](Cc1ccccc1)NP(O)(=S)c1ccccc1 | alpha | 1.8992 | 321.338 | TRUE | TRUE |
| CHEMBL48038 | NC(Cc1c(OCC(=O)O)noc1-c1ccccc1)C(=O)O | alpha | 0.7594 | 306.274 | TRUE | FALSE |
| CHEMBL48054 | CC(C)(C)Cc1onc(OCC(=O)O)c1CC(N)C(=O)O | alpha | 0.681 | 300.311 | TRUE | FALSE |
| CHEMBL480879 | CC(C)c1cnn(O)c1[C@@H](N)C(=O)O | alpha | 0.3282 | 199.21 | TRUE | TRUE |
| CHEMBL482076 | N[C@@H](C(=O)O)c1c(Cl)cnn1O | alpha | -0.1418 | 191.574 | TRUE | TRUE |
| CHEMBL483331 | Cc1c(O)noc1C[C@H](N)C(=O)O | alpha | -0.35698 | 186.167 | TRUE | TRUE |
| CHEMBL4845882 | CC(C)[C@@](C)(N)C(=O)N[C@H]1CN[C@H](C(=O)O)[C@@H]1CCCB(O)O | alpha | -1.2298 | 329.206 | FALSE | TRUE |
| CHEMBL4847500 | O=C(O)[C@H]1NC[C@@H]2NC[C@@]21CCCB(O)O | alpha | -1.746 | 228.057 | FALSE | TRUE |
| CHEMBL4848931 | CC(=O)N[C@H]1CN[C@H](C(=O)O)[C@@H]1CCCB(O)O | alpha | -1.5833 | 258.083 | FALSE | TRUE |
| CHEMBL484913 | N[C@@H](CCCCc1cnc[nH]1)C(=O)O | alpha | 0.5344 | 197.238 | TRUE | TRUE |
| CHEMBL484914 | N[C@@H](CCc1cnc[nH]1)C(=O)O | alpha | -0.2458 | 169.184 | TRUE | TRUE |
| CHEMBL4850213 | CC(C)[C@H](N)C(=O)N[C@H]1CN[C@H](C(=O)O)[C@@H]1CCCB(O)O | alpha | -1.6199 | 315.179 | FALSE | TRUE |
| CHEMBL4850423 | O=C(O)[C@H]1NC[C@@H]2SC[C@@]21CCCB(O)O | alpha | -0.6024 | 245.109 | FALSE | TRUE |
| CHEMBL4851360 | O=C(O)[C@H]1NC[C@@H]1CCCB(O)O | alpha | -1.088 | 187.004 | FALSE | TRUE |
| CHEMBL4852406 | NC[C@@]1(CCCB(O)O)CCN[C@@H]1C(=O)O | alpha | -1.369 | 230.073 | FALSE | TRUE |
| CHEMBL4853009 | O=C(O)C1CC(CCB(O)O)CCN1 | alpha | -0.6979 | 201.031 | FALSE | FALSE |
| CHEMBL4853323 | O=C(O)[C@H]1NCCO[C@H]1CCCB(O)O | alpha | -1.319 | 217.03 | FALSE | TRUE |
| CHEMBL485526 | N[C@@H](CCCc1cncn1-c1ccccc1)C(=O)O | alpha | 1.6069 | 259.309 | TRUE | TRUE |
| CHEMBL485527 | N[C@@H](CCCCc1cncn1-c1ccccc1)C(=O)O | alpha | 1.997 | 273.336 | TRUE | TRUE |
| CHEMBL4855923 | O=C(O)[C@H]1NCC[C@@]1(CO)CCCB(O)O | alpha | -1.3354 | 231.057 | FALSE | TRUE |
| CHEMBL4856276 | CC(C)[C@@H](N)C(=O)N[C@H]1CN[C@H](C(=O)O)[C@@H]1CCCB(O)O | alpha | -1.6199 | 315.179 | FALSE | TRUE |
| CHEMBL4856326 | O=C(O)[C@H]1NC[C@H]1CCCB(O)O | alpha | -1.088 | 187.004 | FALSE | TRUE |
| CHEMBL485864 | C[S+]([O-])c1cccc(N[C@H](CO)C(=O)O)c1 | alpha | 0.2814 | 243.284 | TRUE | TRUE |
| CHEMBL486279 | CC(CCSCCC(N)C(=O)O)CC(=O)O | alpha | 1.0225 | 249.332 | FALSE | FALSE |
| CHEMBL4862982 | CO[C@H]1CN[C@H](C(=O)O)[C@@H]1CCCB(O)O | alpha | -1.073 | 231.057 | FALSE | TRUE |
| CHEMBL4862990 | NC[C@@H]1C[C@@H](CCCB(O)O)[C@@H](C(=O)O)N1 | alpha | -1.3706 | 230.073 | FALSE | TRUE |
| CHEMBL4863141 | O=C(O)C1NCCCC1CCCB(O)O | alpha | -0.3078 | 215.058 | FALSE | FALSE |
| CHEMBL4863411 | C[C@]1(CCCB(O)O)[C@@H](C(=O)O)N[C@H]2C[C@H]21 | alpha | -0.3094 | 227.069 | FALSE | TRUE |
| CHEMBL4863489 | CN[C@H]1CN[C@H](C(=O)O)[C@@H]1CCCB(O)O | alpha | -1.5 | 230.073 | FALSE | TRUE |
| CHEMBL4864502 | CN[C@H](C(=O)N[C@H]1CN[C@H](C(=O)O)[C@@H]1CCCB(O)O)C(C)C | alpha | -1.3592 | 329.206 | FALSE | TRUE |
| CHEMBL4865209 | O=C(O)[C@H]1NC[C@@H]2COC[C@@]21CCCB(O)O | alpha | -1.0714 | 243.068 | FALSE | TRUE |
| CHEMBL4867493 | O=C(O)[C@H]1NC[C@H](NCC(F)(F)F)[C@H]1CCCB(O)O | alpha | -0.5675 | 298.07 | FALSE | TRUE |
| CHEMBL4868283 | C[C@@H]1C[C@@H](CCCB(O)O)[C@@H](C(=O)O)N1 | alpha | -0.3094 | 215.058 | FALSE | TRUE |
| CHEMBL4868849 | CN(C)[C@H]1CN[C@H](C(=O)O)[C@@H]1CCCB(O)O | alpha | -1.1578 | 244.1 | FALSE | TRUE |
| CHEMBL4870852 | NC(Cc1ccc(Oc2ccc(O)cc2)cc1)C(=O)O | alpha | 2.1389 | 273.288 | TRUE | FALSE |
| CHEMBL4870874 | O=C(O)[C@H]1NCCC[C@H]1CCB(O)O | alpha | -0.6979 | 201.031 | FALSE | TRUE |
| CHEMBL4871090 | C[C@]1(CCCB(O)O)[C@@H](C(=O)O)N[C@@H]2CC[C@@H]21 | alpha | 0.0807 | 241.096 | FALSE | TRUE |
| CHEMBL487146 | Cc1ccc(S(=O)(=O)N[C@H](CCC(=O)O)C(=O)O)cc1 | alpha | 0.59132 | 301.32 | TRUE | TRUE |
| CHEMBL4871970 | O=C(O)[C@H]1NC[C@@H]2NCC[C@@]21CCCB(O)O | alpha | -1.3559 | 242.084 | FALSE | TRUE |
| CHEMBL4872801 | O=C(O)[C@H]1N[C@H](CO)C[C@H]1CCCB(O)O | alpha | -1.337 | 231.057 | FALSE | TRUE |
| CHEMBL487301 | CN(CCSCCC(N)C(=O)O)CC(=O)O | alpha | -0.462 | 250.32 | FALSE | FALSE |
| CHEMBL487302 | C[C@H](NCCSCCC(N)C(=O)O)C(=O)O | alpha | -0.4157 | 250.32 | FALSE | TRUE |
| CHEMBL4875190 | O=C(O)C1CC(CCB(O)O)CN1 | alpha | -1.088 | 187.004 | FALSE | FALSE |
| CHEMBL4875641 | C[C@H](N)C(=O)N[C@H]1CN[C@H](C(=O)O)[C@@H]1CCCB(O)O | alpha | -2.256 | 287.125 | FALSE | TRUE |
| CHEMBL4876150 | C[C@@]1(CCCB(O)O)CCN[C@@H]1C(=O)O | alpha | -0.3078 | 215.058 | FALSE | TRUE |
| CHEMBL488121 | CC(C)(CCSCC[C@H](N)C(=O)O)CC(=O)O | alpha | 1.4126 | 263.359 | FALSE | TRUE |
| CHEMBL488378 | O=C(O)CC[C@@H](NS(=O)(=O)Cc1ccccc1)C(=O)O | alpha | 0.424 | 301.32 | TRUE | TRUE |
| CHEMBL488540 | NC(CCCSCCCC(=O)O)C(=O)O | alpha | 0.7765 | 235.305 | FALSE | FALSE |
| CHEMBL48861 | C/N=C(/N)Nc1cccc(CC(N)C(=O)O)c1 | alpha | -0.0026 | 236.275 | TRUE | FALSE |
| CHEMBL488739 | C[N+](C)(CCSCCC(N)C(=O)O)CC(=O)[O-] | alpha | -1.6521 | 264.347 | FALSE | FALSE |
| CHEMBL489059 | C[S+]([O-])c1cccc(N[C@@H](CO)C(=O)O)c1 | alpha | 0.2814 | 243.284 | TRUE | TRUE |
| CHEMBL48933 | CSC[C@@H](C)[C@H](N)C(=O)O | alpha | 0.3974 | 163.242 | FALSE | TRUE |
| CHEMBL4896574 | CCC(C)C(NCCC(=O)c1ccc(Cl)cc1)C(=O)O | alpha | 3.0017 | 297.782 | TRUE | FALSE |
| CHEMBL4897361 | Cc1ccccc1C(C)NC(C)C(=O)O | alpha | 2.11872 | 207.273 | TRUE | FALSE |
| CHEMBL4898493 | CC(C)CCN[C@@H](CC(=O)Nc1cccc(Cl)c1Cl)C(=O)O | alpha | 3.4109 | 347.242 | TRUE | TRUE |
| CHEMBL48995 | CCCCN[C@@H](CCSC)C(=O)O | alpha | 1.5824 | 205.323 | FALSE | TRUE |
| CHEMBL4900155 | CCN[C@@H](CC(=O)Nc1ccc(Cl)c(Cl)c1)C(=O)O | alpha | 2.3847 | 305.161 | TRUE | TRUE |
| CHEMBL49010 | CC(=N)Nc1cccc(CC(N)C(=O)O)c1 | alpha | 1.05007 | 221.26 | TRUE | FALSE |
| CHEMBL49034 | CNCCC(N)C(=O)O | alpha | -0.9922 | 132.163 | FALSE | FALSE |
| CHEMBL4907031 | C=CCC(NCC(C)CCc1ccccc1)C(=O)O | alpha | 2.8742 | 261.365 | TRUE | FALSE |
| CHEMBL4907863 | O=C(O)[C@H](Cc1ccccc1)Nc1ncccn1 | alpha | 1.5844 | 243.266 | TRUE | TRUE |
| CHEMBL4908862 | Cc1ccsc1S(=O)(=O)N[C@@H](CC(C)C)C(=O)O | alpha | 1.83412 | 291.394 | TRUE | TRUE |
| CHEMBL4911911 | Cc1cccc(S(=O)(=O)N[C@H](C(=O)O)C(C)C)c1Cl | alpha | 2.03592 | 305.783 | TRUE | TRUE |
| CHEMBL4918409 | CC(C)[C@H](NCCC(=O)c1ccc(Br)cc1)C(=O)O | alpha | 2.7207 | 328.206 | TRUE | TRUE |
| CHEMBL4922520 | Cc1cccc(S(=O)(=O)N[C@H](C(=O)O)C(C)C)c1F | alpha | 1.52162 | 289.328 | TRUE | TRUE |
| CHEMBL492629 | CC1(C)Cc2c(n(C[C@H](N)C(=O)O)c(=O)[nH]c2=O)C1 | alpha | -0.9267 | 267.285 | TRUE | TRUE |
| CHEMBL492630 | N[C@@H](Cn1c(=O)[nH]c(=O)c2sccc21)C(=O)O | alpha | -0.8368 | 255.255 | TRUE | TRUE |
| CHEMBL4928792 | CC(C)C[C@@H](NS(=O)(=O)c1cnc(-c2ccccc2)nc1)C(=O)O | alpha | 1.9212 | 349.412 | TRUE | TRUE |
| CHEMBL493208 | NS(=O)(=O)c1cccc(N[C@@H](CO)C(=O)O)c1 | alpha | -0.8086 | 260.271 | TRUE | TRUE |
| CHEMBL4932103 | CCCCC(CC)CNC(CC(=O)Nc1ccccc1C#N)C(=O)O | alpha | 3.14608 | 345.443 | TRUE | FALSE |
| CHEMBL493665 | N[C@@H](CCn1c2c(c(=O)[nH]c1=O)CCC2)C(=O)O | alpha | -1.1727 | 253.258 | TRUE | TRUE |
| CHEMBL494215 | CCOP(=O)(OCC)c1cccc(N[C@@H](CO)C(=O)O)c1 | alpha | 1.4354 | 317.278 | TRUE | TRUE |
| CHEMBL494216 | O=C(O)c1cccc(N[C@H](CO)C(=O)O)c1 | alpha | 0.2422 | 225.2 | TRUE | TRUE |
| CHEMBL494217 | CSc1cccc(N[C@H](CO)C(=O)O)c1 | alpha | 1.2659 | 227.285 | TRUE | TRUE |
| CHEMBL4944142 | CC[C@H](Nc1ccc(OC)cc1)C(=O)O | alpha | 1.9703 | 209.245 | TRUE | TRUE |
| CHEMBL4947989 | N[C@@H](Cc1ccsc1Cl)C(=O)O | alpha | 1.3559 | 205.666 | TRUE | TRUE |
| CHEMBL494878 | CCCNC(=O)CC[C@H](C[C@H](N)C(=O)O)C(=O)O | alpha | -0.2044 | 260.29 | FALSE | TRUE |
| CHEMBL4950671 | N[C@@H](C(=O)O)c1ccc(F)c(Cl)c1 | alpha | 1.5635 | 203.6 | TRUE | TRUE |
| CHEMBL495223 | O=C(O)c1cccc(N[C@@H](CO)C(=O)O)c1 | alpha | 0.2422 | 225.2 | TRUE | TRUE |
| CHEMBL4955056 | N[C@@H](Cc1c(F)cccc1Cl)C(=O)O | alpha | 1.4335 | 217.627 | TRUE | TRUE |
| CHEMBL4962483 | CCOc1ccc(CN[C@@H](C(=O)O)c2ccnn2C)cc1F | alpha | 1.8734 | 307.325 | TRUE | TRUE |
| CHEMBL4966741 | Cc1ccc(C(C)NC(C)C(=O)O)cc1F | alpha | 2.25782 | 225.263 | TRUE | FALSE |
| CHEMBL49687 | CSC[C@@H](C)C(N)C(=O)O | alpha | 0.3974 | 163.242 | FALSE | TRUE |
| CHEMBL4971532 | O=C(C[C@H](NCCO)C(=O)O)c1ccc2c(c1)CCCC2 | alpha | 1.1733 | 291.347 | TRUE | TRUE |
| CHEMBL4979387 | O=C(O)C1CCC(Cc2ccc(O)cc2)N1 | alpha | 1.1399 | 221.256 | TRUE | FALSE |
| CHEMBL497950 | N[C@@H](C(=O)O)[C@@H](O)c1ccc([N+](=O)[O-])cc1 | alpha | 0.0401 | 226.188 | TRUE | TRUE |
| CHEMBL4984704 | O=C(O)C1CC(Oc2cccc(Cl)c2)CN1 | alpha | 1.5339 | 241.674 | TRUE | FALSE |
| CHEMBL4985681 | O=C(CCN[C@@H](CO)C(=O)O)c1ccc(Br)cc1 | alpha | 1.057 | 316.151 | TRUE | TRUE |
| CHEMBL4987041 | O=C(O)[C@H](Cc1ccccc1)Nc1nc(C(F)(F)F)ns1 | alpha | 2.6647 | 317.292 | TRUE | TRUE |
| CHEMBL49881 | C/N=C(/N)Nc1ccccc1CC(N)C(=O)O | alpha | -0.0026 | 236.275 | TRUE | FALSE |
| CHEMBL4992563 | CCCc1ccc(CN[C@@H](C(=O)O)c2ccnn2C)s1 | alpha | 2.3496 | 293.392 | TRUE | TRUE |
| CHEMBL49934 | N=C(N)Nc1cccc(CC(N)C(=O)O)c1 | alpha | -0.05363 | 222.248 | TRUE | FALSE |
| CHEMBL4999446 | Cc1cc(O)ccc1NC(=O)C[C@H](NCC(C)C)C(=O)O | alpha | 1.72802 | 294.351 | TRUE | TRUE |
| CHEMBL50041 | CCC/C=C(/C[C@H](N)C(=O)O)C(=O)O | alpha | 0.5995 | 201.222 | FALSE | TRUE |
| CHEMBL500448 | N[C@@H](C[C@@H](CO)C(=O)O)C(=O)O | alpha | -1.5185 | 177.156 | FALSE | TRUE |
| CHEMBL500839 | Cc1cc(C)cc(CC(C(=O)O)[C@H](N)C(=O)O)c1 | alpha | 0.95864 | 251.282 | TRUE | TRUE |
| CHEMBL5009345 | CC(C)C(NCC1CCCC(C)(C)C1)C(=O)O | alpha | 2.9016 | 241.375 | FALSE | FALSE |
| CHEMBL5009733 | CN(C)CCN[C@@H](CC(=O)Nc1cccc(Cl)c1Cl)C(=O)O | alpha | 1.9264 | 348.23 | TRUE | TRUE |
| CHEMBL5011552 | Cn1nccc1[C@@H](NCc1cc(Cl)cs1)C(=O)O | alpha | 2.0505 | 285.756 | TRUE | TRUE |
| CHEMBL5013206 | Cc1cc(N[C@H](C(=O)O)C2CCCCC2)nc(C(C)C)n1 | alpha | 3.35382 | 291.395 | TRUE | TRUE |
| CHEMBL501418 | N[C@H](C(=O)O)c1ccnn1O | alpha | -0.7952 | 157.129 | TRUE | TRUE |
| CHEMBL5024749 | CCCOc1ccc(C(O)CCNC(C)C(=O)O)cc1 | alpha | 1.9616 | 281.352 | TRUE | FALSE |
| CHEMBL502477 | C[C@H](Nn1ccc(=O)c(O)c1)C(=O)O | alpha | -0.4295 | 198.178 | TRUE | TRUE |
| CHEMBL502740 | N[C@@H](CCCCCP(=O)(O)O)C(=O)O | alpha | 0.1364 | 225.181 | FALSE | TRUE |
| CHEMBL503142 | Cc1cnn(O)c1C(N)C(=O)O | alpha | -0.48678 | 171.156 | TRUE | FALSE |
| CHEMBL503440 | Cc1cccc([C@H]2CC[C@](C(=O)O)([C@@H](O)[C@@H](N)C(=O)O)N2)c1 | alpha | 0.01572 | 308.334 | TRUE | TRUE |
| CHEMBL504184 | O=C(O)[C@H]1C[C@@H](O)CCN1 | alpha | -0.8161 | 145.158 | FALSE | TRUE |
| CHEMBL504317 | N[C@@H](C(=O)O)c1ccnn1O | alpha | -0.7952 | 157.129 | TRUE | TRUE |
| CHEMBL504772 | NC(C(=O)O)c1ccnn1O | alpha | -0.7952 | 157.129 | TRUE | FALSE |
| CHEMBL505700 | CCc1cnn(O)c1[C@H](N)C(=O)O | alpha | -0.2328 | 185.183 | TRUE | TRUE |
| CHEMBL505731 | CN(CCCSCCC(N)C(=O)O)CC(=O)O | alpha | -0.0719 | 264.347 | FALSE | FALSE |
| CHEMBL506027 | CCc1cnn(O)c1C(N)C(=O)O | alpha | -0.2328 | 185.183 | TRUE | FALSE |
| CHEMBL506409 | NC(C(=O)O)c1c(Cl)cnn1O | alpha | -0.1418 | 191.574 | TRUE | FALSE |
| CHEMBL5072895 | N[C@H](CNC(=O)c1ccc(-c2ccccc2)o1)C(=O)O | alpha | 1.0883 | 274.276 | TRUE | TRUE |
| CHEMBL5075942 | COc1ccc(-c2ccc(C(=O)NC[C@@H](N)C(=O)O)o2)cc1 | alpha | 1.0969 | 304.302 | TRUE | TRUE |
| CHEMBL5076681 | N[C@H](CNC(=O)c1ccc(-c2cccc(Cl)c2)o1)C(=O)O | alpha | 1.7417 | 308.721 | TRUE | TRUE |
| CHEMBL507830 | O=C(O)[C@H]1C[C@H](O)CCN1 | alpha | -0.8161 | 145.158 | FALSE | TRUE |
| CHEMBL507969 | O=C(O)CC[C@@H](NS(=O)(=O)c1ccc2ccccc2c1)C(=O)O | alpha | 1.4361 | 337.353 | TRUE | TRUE |
| CHEMBL508094 | C[C@@H]([C@H](C)O)[C@H](N)C(=O)O | alpha | -0.5848 | 147.174 | FALSE | TRUE |
| CHEMBL5081008 | CCc1ccccc1-c1ccc(C(=O)NC[C@@H](N)C(=O)O)o1 | alpha | 1.6507 | 302.33 | TRUE | TRUE |
| CHEMBL5081203 | N[C@H](CNC(=O)c1ccc(-c2ccccc2Cl)o1)C(=O)O | alpha | 1.7417 | 308.721 | TRUE | TRUE |
| CHEMBL5083882 | N[C@H](CNC(=O)c1ccc(-c2ccc(Cl)c(Cl)c2)o1)C(=O)O | alpha | 2.3951 | 343.166 | TRUE | TRUE |
| CHEMBL5085583 | Cc1ccccc1-c1ccc(C(=O)NC[C@@H](N)C(=O)O)o1 | alpha | 1.39672 | 288.303 | TRUE | TRUE |
| CHEMBL5087170 | COCc1ccccc1-c1ccc(C(=O)NC[C@@H](N)C(=O)O)o1 | alpha | 1.2347 | 318.329 | TRUE | TRUE |
| CHEMBL5087374 | N[C@H](CNC(=O)c1ccc(-c2ccccc2C(F)(F)F)o1)C(=O)O | alpha | 2.1071 | 342.273 | TRUE | TRUE |
| CHEMBL5087550 | N[C@H](CNC(=O)c1ccc(-c2cccc(C(F)(F)F)c2)o1)C(=O)O | alpha | 2.1071 | 342.273 | TRUE | TRUE |
| CHEMBL508818 | N[C@@H](Cn1c(=O)[nH]c(=O)c2ccccc21)C(=O)O | alpha | -0.8983 | 249.226 | TRUE | TRUE |
| CHEMBL509090 | O=C(O)[C@@H]1C[C@@H](OS(=O)(=O)O)CCN1 | alpha | -0.989 | 225.222 | FALSE | TRUE |
| CHEMBL509344 | CC(NC(=S)c1ccccc1)C(=O)O | alpha | 1.4248 | 209.27 | TRUE | FALSE |
| CHEMBL5093773 | N[C@H](CNC(=O)c1ccc(-c2ccc(Cl)cc2)o1)C(=O)O | alpha | 1.7417 | 308.721 | TRUE | TRUE |
| CHEMBL509466 | CC1(C)COC(CCN[C@@H](CCCNC(=N)N)C(=O)O)OC1 | alpha | 0.08167 | 316.402 | FALSE | TRUE |
| CHEMBL509731 | N[C@H](C(=O)O)C(Cc1cccc([N+](=O)[O-])c1)C(=O)O | alpha | 0.25 | 268.225 | TRUE | TRUE |
| CHEMBL51008 | CCC(CC)=C(C[C@H](N)C(=O)O)C(=O)O | alpha | 0.9896 | 215.249 | FALSE | TRUE |
| CHEMBL510487 | NS(=O)(=O)c1cccc(N[C@H](CO)C(=O)O)c1 | alpha | -0.8086 | 260.271 | TRUE | TRUE |
| CHEMBL51054 | N[C@@H](CC(C(=O)O)=C1CCC1)C(=O)O | alpha | 0.3535 | 199.206 | FALSE | TRUE |
| CHEMBL51395 | CC1(C)CCC/C1=C(/C[C@H](N)C(=O)O)C(=O)O | alpha | 1.3797 | 241.287 | FALSE | TRUE |
| CHEMBL51660 | N[C@@H](CC(C(=O)O)=C1CCCC1)C(=O)O | alpha | 0.7436 | 213.233 | FALSE | TRUE |
| CHEMBL517209 | N[C@@H](CC(=O)Nc1ccc(OCc2ccccc2)cc1)C(=O)O | alpha | 2.0061 | 314.341 | TRUE | TRUE |
| CHEMBL517211 | N[C@@H](CCC(=O)Nc1ccc(Nc2ccccc2)cc1)C(=O)O | alpha | 2.5608 | 313.357 | TRUE | TRUE |
| CHEMBL5173310 | NC(CSCc1ccc(C(=O)c2ccccc2)cc1)C(=O)O | alpha | 2.5627 | 315.394 | TRUE | FALSE |
| CHEMBL5177039 | C[N+](C)(C)CCC[C@H](N)C(=O)O.Cl.[Cl-] | alpha | -2.6895 | 247.166 | FALSE | TRUE |
| CHEMBL518147 | CC(C)c1cnn(O)c1C(N)C(=O)O | alpha | 0.3282 | 199.21 | TRUE | FALSE |
| CHEMBL5182629 | N#Cc1ccc(/C=C/CNCC[C@H](N)C(=O)O)cc1 | alpha | 0.96308 | 259.309 | TRUE | TRUE |
| CHEMBL518313 | N[C@@H](C[C@@H](CCC(=O)NCc1ccccc1)C(=O)O)C(=O)O | alpha | 0.5858 | 308.334 | TRUE | TRUE |
| CHEMBL5183425 | CN(C/C=C/c1ccc(C#N)cc1)CC[C@H](N)C(=O)O | alpha | 1.30528 | 273.336 | TRUE | TRUE |
| CHEMBL5184064 | NC(CNC(=O)C(=O)O)C(=O)O | alpha | -2.4009 | 176.128 | FALSE | FALSE |
| CHEMBL5188085 | N[C@@H](Cc1ccc2[nH]c(=O)c(=O)[nH]c2c1)C(=O)O | alpha | -0.8292 | 249.226 | TRUE | TRUE |
| CHEMBL519109 | N[C@@H](C(=O)O)[C@H](O)[C@@]1(C(=O)O)CC[C@H](c2ccccc2)N1 | alpha | -0.2927 | 294.307 | TRUE | TRUE |
| CHEMBL5192372 | CC(C)[N+](C)(C)CCCC[C@H](N)C(=O)O.Cl.[Cl-] | alpha | -1.5208 | 289.247 | FALSE | TRUE |
| CHEMBL5195112 | CCC[N+](C)(C)CCCC[C@H](N)C(=O)O.Cl.[Cl-] | alpha | -1.5192 | 289.247 | FALSE | TRUE |
| CHEMBL5196653 | NC(Cn1c(=O)c(=O)[nH]c2ccccc21)C(=O)O | alpha | -0.8983 | 249.226 | TRUE | FALSE |
| CHEMBL5196808 | CC[N+](C)(C)CCCC[C@H](N)C(=O)O.Cl.[Cl-] | alpha | -1.9093 | 275.22 | FALSE | TRUE |
| CHEMBL5197010 | CC(C)C[C@H](Nc1ncnc2[nH]ccc12)C(=O)O | alpha | 1.8691 | 248.286 | TRUE | TRUE |
| CHEMBL5208790 | C[N+](C)(C)CCCCC[C@H](N)C(=O)O.Cl.[Cl-] | alpha | -1.9093 | 275.22 | FALSE | TRUE |
| CHEMBL5218520 | O=C(O)c1cccc(CN[C@H](CCB(O)O)C(=O)O)c1 | alpha | -0.2095 | 281.073 | TRUE | TRUE |
| CHEMBL5218792 | N[C@H](CN[C@H](CCB(O)O)C(=O)O)Cc1ccccc1 | alpha | -0.5379 | 280.133 | TRUE | TRUE |
| CHEMBL5218962 | N[C@@H](CN[C@H](CCB(O)O)C(=O)O)Cc1ccccc1 | alpha | -0.5379 | 280.133 | TRUE | TRUE |
| CHEMBL5219218 | C[C@@H](N)CN[C@H](CCB(O)O)C(=O)O | alpha | -1.7607 | 204.035 | FALSE | TRUE |
| CHEMBL5219724 | O=C(O)[C@@H](CCB(O)O)NCCCc1ccccc1 | alpha | 0.5249 | 265.118 | TRUE | TRUE |
| CHEMBL5219778 | O=C(O)[C@@H](CCB(O)O)NCc1ccccc1 | alpha | 0.0923 | 237.064 | TRUE | TRUE |
| CHEMBL5220006 | COc1ccc(CN[C@H](CCB(O)O)C(=O)O)cc1 | alpha | 0.1009 | 267.09 | TRUE | TRUE |
| CHEMBL5220724 | O=C(O)[C@@H](CCB(O)O)NCCc1ccccc1 | alpha | 0.1348 | 251.091 | TRUE | TRUE |
| CHEMBL5221744 | C[N+](C)(C)CCC[C@H](N)C(=O)O | alpha | -0.1153 | 175.252 | FALSE | TRUE |
| CHEMBL5222160 | CC(C)[N+](C)(C)CCCC[C@H](N)C(=O)O | alpha | 1.0534 | 217.333 | FALSE | TRUE |
| CHEMBL5222325 | CC[N+](C)(C)CCCC[C@H](N)C(=O)O | alpha | 0.6649 | 203.306 | FALSE | TRUE |
| CHEMBL5222465 | CCC[N+](C)(C)CCCC[C@H](N)C(=O)O | alpha | 1.055 | 217.333 | FALSE | TRUE |
| CHEMBL5222809 | C[N+](C)(C)CCCCC[C@H](N)C(=O)O | alpha | 0.6649 | 203.306 | FALSE | TRUE |
| CHEMBL52280 | CC(=N)Nc1ccccc1CC(N)C(=O)O | alpha | 1.05007 | 221.26 | TRUE | FALSE |
| CHEMBL52295 | CC/C=C(/C[C@H](N)C(=O)O)C(=O)O | alpha | 0.2094 | 187.195 | FALSE | TRUE |
| CHEMBL52514 | C/C=C(/C[C@H](N)C(=O)O)C(=O)O | alpha | -0.1807 | 173.168 | FALSE | TRUE |
| CHEMBL526030 | CC(C)[C@H](NCC(=O)c1ccccc1)C(=O)O.Cl | alpha | 1.9899 | 271.744 | TRUE | TRUE |
| CHEMBL5267843 | C=C(C[C@H](N)C(=O)O)C(=O)Nc1ccc(Cl)cc1 | alpha | 1.6367 | 268.7 | TRUE | TRUE |
| CHEMBL5268691 | O=C(O)C1CS[C@@H](c2cccc(F)c2F)N1 | alpha | 1.753 | 245.25 | TRUE | TRUE |
| CHEMBL5268925 | COc1ccccc1[C@H]1NC(C(=O)O)CS1 | alpha | 1.4834 | 239.296 | TRUE | TRUE |
| CHEMBL5272397 | NC(Cc1coc2ccccc12)C(=O)O | alpha | 1.3872 | 205.213 | TRUE | FALSE |
| CHEMBL52742 | CC(C)(C)/C=C(\C[C@H](N)C(=O)O)C(=O)O | alpha | 0.8455 | 215.249 | FALSE | TRUE |
| CHEMBL5274872 | C=C(C[C@H](N)C(=O)O)C(=O)Nc1ccccc1 | alpha | 0.9833 | 234.255 | TRUE | TRUE |
| CHEMBL5275042 | N[C@@H](Cc1cccc(NC(=O)c2ccccc2O)c1)C(=O)O | alpha | 1.5989 | 300.314 | TRUE | TRUE |
| CHEMBL5275111 | O=C(O)C1CS[C@@H](c2ccccc2)N1 | alpha | 1.4748 | 209.27 | TRUE | TRUE |
| CHEMBL5277774 | CC(=O)C1CSC(C(=O)O)N1 | alpha | -0.3089 | 175.209 | FALSE | FALSE |
| CHEMBL5279453 | Cc1ccc([C@H]2NC(C(=O)O)CS2)cc1 | alpha | 1.78322 | 223.297 | TRUE | TRUE |
| CHEMBL5280215 | C=C(C[C@H](N)C(=O)O)C(=O)N1CCCCC1 | alpha | 0.3571 | 226.276 | FALSE | TRUE |
| CHEMBL5280896 | O=C(O)C1CS[C@@H](c2cccc(Cl)c2)N1 | alpha | 2.1282 | 243.715 | TRUE | TRUE |
| CHEMBL5281484 | O=C(O)C1CS[C@@H](c2cccc(Cl)c2Cl)N1 | alpha | 2.7816 | 278.16 | TRUE | TRUE |
| CHEMBL5281769 | N[C@@H](CC/C=C/B(O)O)C(=O)O | alpha | -1.2533 | 172.977 | FALSE | TRUE |
| CHEMBL5282845 | C=C(C[C@H](N)C(=O)O)C(=O)Nc1ccc(F)cc1 | alpha | 1.1224 | 252.245 | TRUE | TRUE |
| CHEMBL5283079 | C=C(C[C@H](N)C(=O)O)C(N)=O | alpha | -1.1701 | 158.157 | FALSE | TRUE |
| CHEMBL5283716 | N[C@@H](C[Se][Se]C[C@H](N)C(=O)O)C(=O)O | alpha | -2.0298 | 334.092 | FALSE | TRUE |
| CHEMBL5285537 | C=C(C[C@H](N)C(=O)O)C(=O)NCc1ccccc1 | alpha | 0.661 | 248.282 | TRUE | TRUE |
| CHEMBL5285943 | C=C(C[C@H](N)C(=O)O)C(=O)NCc1ccc(F)cc1 | alpha | 0.8001 | 266.272 | TRUE | TRUE |
| CHEMBL5287570 | N[C@@H](CCCCS(N)(=O)=O)C(=O)O | alpha | -1.1429 | 210.255 | FALSE | TRUE |
| CHEMBL5289666 | C=C(C[C@H](N)C(=O)O)C(=O)NCc1ccc([N+](=O)[O-])cc1 | alpha | 0.5692 | 293.279 | TRUE | TRUE |
| CHEMBL5289865 | C=C(C[C@H](N)C(=O)O)C(=O)NC1CC1 | alpha | -0.3768 | 198.222 | FALSE | TRUE |
| CHEMBL5305110 | O=C(O)C(Cc1ccc(F)cc1)NS(=O)(=O)c1cccs1 | alpha | 1.8614 | 329.374 | TRUE | FALSE |
| CHEMBL5305225 | CC(C)C(NS(=O)(=O)c1cccc2nsnc12)C(=O)O | alpha | 1.0788 | 315.376 | TRUE | FALSE |
| CHEMBL5305233 | CCC(C)C(NS(=O)(=O)c1ccc2c(c1)OCCO2)C(=O)O | alpha | 1.2354 | 329.374 | TRUE | FALSE |
| CHEMBL5305281 | CCC(C)C(NS(=O)(=O)c1ccc(Cl)cc1)C(=O)O | alpha | 2.1176 | 305.783 | TRUE | FALSE |
| CHEMBL5305333 | CSCCC(Nc1nc(C)nc2sc(C)c(C)c12)C(=O)O | alpha | 3.23476 | 325.459 | TRUE | FALSE |
| CHEMBL5305366 | O=C(O)C(Cc1ccccc1)NS(=O)(=O)c1cccs1 | alpha | 1.7223 | 311.384 | TRUE | FALSE |
| CHEMBL5305378 | CCC(C)C(NS(=O)(=O)c1ccc(OC)cc1)C(=O)O | alpha | 1.4728 | 301.364 | TRUE | FALSE |
| CHEMBL5305388 | C[C@H](NS(=O)(=O)c1c(Cl)cccc1Cl)C(=O)O | alpha | 1.7448 | 298.147 | TRUE | TRUE |
| CHEMBL5305501 | CCCC(NS(=O)(=O)c1cccc2nsnc12)C(=O)O | alpha | 1.2229 | 315.376 | TRUE | FALSE |
| CHEMBL5305521 | Cc1ccc(S(=O)(=O)NC(CC(C)C)C(=O)O)cc1 | alpha | 1.77262 | 285.365 | TRUE | FALSE |
| CHEMBL5305669 | O=C(O)C(Cc1ccccc1)NS(=O)(=O)c1ccccc1F | alpha | 1.7999 | 323.345 | TRUE | FALSE |
| CHEMBL5305713 | CC(C)C(NS(=O)(=O)c1ccc(F)c(Cl)c1)C(=O)O | alpha | 1.8666 | 309.746 | TRUE | FALSE |
| CHEMBL5305767 | CCC(NS(=O)(=O)c1ccc2ccccc2c1)C(=O)O | alpha | 1.9813 | 293.344 | TRUE | FALSE |
| CHEMBL5305782 | Cc1ccc(C)c(S(=O)(=O)NC(C(=O)O)C(C)C)c1 | alpha | 1.69094 | 285.365 | TRUE | FALSE |
| CHEMBL53060 | CS/C(=N/N)NCCC[C@H](N)C(=O)O | alpha | -0.6392 | 220.298 | FALSE | TRUE |
| CHEMBL5306037 | COc1ccc(S(=O)(=O)N[C@@H](Cc2ccccc2)C(=O)O)cc1 | alpha | 1.6694 | 335.381 | TRUE | TRUE |
| CHEMBL5306281 | CC(C)CC(NS(=O)(=O)c1ccc(Br)cc1)C(=O)O | alpha | 2.2267 | 350.234 | TRUE | FALSE |
| CHEMBL5306324 | CC(C)[C@H](NS(=O)(=O)/C=C/c1ccccc1)C(=O)O | alpha | 1.6859 | 283.349 | TRUE | TRUE |
| CHEMBL5306447 | O=C(O)[C@H](Cc1ccccc1)NS(=O)(=O)c1ccc(F)cc1 | alpha | 1.7999 | 323.345 | TRUE | TRUE |
| CHEMBL5306482 | O=C(O)C(Cc1ccccc1)NS(=O)(=O)c1ccc(F)cc1 | alpha | 1.7999 | 323.345 | TRUE | FALSE |
| CHEMBL5306500 | CSC[C@H](NS(=O)(=O)c1ccc(Cl)cc1)C(=O)O | alpha | 1.4345 | 309.796 | TRUE | TRUE |
| CHEMBL5306581 | CC(C)[C@H](NS(=O)(=O)c1ccccc1F)C(=O)O | alpha | 1.2132 | 275.301 | TRUE | TRUE |
| CHEMBL5306749 | COc1ccc(S(=O)(=O)NC(CC(C)C)C(=O)O)cc1 | alpha | 1.4728 | 301.364 | TRUE | FALSE |
| CHEMBL5306795 | CC(C)CC(NS(=O)(=O)c1ccc(Cl)cc1)C(=O)O | alpha | 2.1176 | 305.783 | TRUE | FALSE |
| CHEMBL5306987 | CCc1cc2c(N[C@@H](CC(C)C)C(=O)O)ncnc2s1 | alpha | 3.1649 | 293.392 | TRUE | TRUE |
| CHEMBL5307034 | CC[C@H](C)[C@H](NS(=O)(=O)c1cccc(C(F)(F)F)c1)C(=O)O | alpha | 2.483 | 339.335 | TRUE | TRUE |
| CHEMBL5307216 | CCc1cc2c(NC(CCSC)C(=O)O)nc(C)nc2s1 | alpha | 3.18032 | 325.459 | TRUE | FALSE |
| CHEMBL5307281 | CCC(C)C(NS(=O)(=O)c1ccc(F)cc1)C(=O)O | alpha | 1.6033 | 289.328 | TRUE | FALSE |
| CHEMBL5307300 | O=C(O)C(Cc1ccc(Cl)cc1)NS(=O)(=O)c1ccccc1 | alpha | 2.3142 | 339.8 | TRUE | FALSE |
| CHEMBL5307360 | CCCCC(NS(=O)(=O)c1ccc(Br)cc1)C(=O)O | alpha | 2.3708 | 350.234 | TRUE | FALSE |
| CHEMBL5307509 | COc1cccc(S(=O)(=O)NC(Cc2ccccc2)C(=O)O)c1 | alpha | 1.6694 | 335.381 | TRUE | FALSE |
| CHEMBL5307591 | Cc1sc2ncnc(N[C@@H](CC(C)C)C(=O)O)c2c1C | alpha | 3.21934 | 293.392 | TRUE | TRUE |
| CHEMBL5307638 | CC(C)C(NS(=O)(=O)c1ccc(Cl)cc1)C(=O)O | alpha | 1.7275 | 291.756 | TRUE | FALSE |
| CHEMBL5307666 | CCCC(NS(=O)(=O)c1ccc(F)cc1)C(=O)O | alpha | 1.3573 | 275.301 | TRUE | FALSE |
| CHEMBL5307994 | CC(C)CC(NS(=O)(=O)c1cccc2c1N=S=N2)C(=O)O | alpha | 2.1906 | 329.403 | TRUE | FALSE |
| CHEMBL5308075 | CC(C)C(NS(=O)(=O)c1ccc2ccccc2c1)C(=O)O | alpha | 2.2273 | 307.371 | TRUE | FALSE |
| CHEMBL5308078 | CCc1cc2c(NC(CCSC)C(=O)O)ncnc2s1 | alpha | 2.8719 | 311.432 | TRUE | FALSE |
| CHEMBL5308291 | CCCCC(NS(=O)(=O)c1ccc2c(c1)CCC(=O)N2)C(=O)O | alpha | 1.493 | 340.401 | TRUE | FALSE |
| CHEMBL5308319 | CC(NS(=O)(=O)c1ccc(Cl)c(C(F)(F)F)c1)C(=O)O | alpha | 2.1102 | 331.699 | TRUE | FALSE |
| CHEMBL5308328 | CSCCC(NS(=O)(=O)c1cc(C)ccc1C)C(=O)O | alpha | 1.78804 | 317.432 | TRUE | FALSE |
| CHEMBL5308377 | NC(CC(F)C(=O)O)C(=O)O | alpha | -0.7889 | 165.12 | FALSE | FALSE |
| CHEMBL5313139 | CC[C@@H](Nc1cc(OC)nc(COC)n1)C(=O)O | alpha | 0.9067 | 255.274 | TRUE | TRUE |
| CHEMBL5314941 | N[C@@H](C[C@H](CCC[18F])C(=O)O)C(=O)O | alpha | 0.2389 | 206.203938 | FALSE | TRUE |
| CHEMBL5316159 | N[C@@H](C[C@H](CCCF)C(=O)O)C(=O)O | alpha | 0.2389 | 207.201 | FALSE | TRUE |
| CHEMBL53194 | N[C@@H](C/C(=C\C=C\c1ccccc1)C(=O)O)C(=O)O | alpha | 1.5128 | 261.277 | TRUE | TRUE |
| CHEMBL535134 | CC(=N)NCC(F)(F)CC[C@H](N)C(=O)O.Cl.Cl | alpha | 1.24417 | 296.145 | FALSE | TRUE |
| CHEMBL535223 | CC1SC(=S)NC1C(=O)O | alpha | 0.4494 | 177.25 | FALSE | FALSE |
| CHEMBL535377 | Cl.N[C@@H](CCP(=O)(O)CO)C(=O)O | alpha | -0.5696 | 233.588 | FALSE | TRUE |
| CHEMBL536253 | CC(=N)NCC(F)(F)CCC(N)C(=O)O.Cl.Cl | alpha | 1.24417 | 296.145 | FALSE | FALSE |
| CHEMBL53702 | N[C@@H](C/C(=C\C1CCCCC1)C(=O)O)C(=O)O | alpha | 1.3797 | 241.287 | FALSE | TRUE |
| CHEMBL53775 | N[C@@H](C/C(=C\CCc1ccccc1)C(=O)O)C(=O)O | alpha | 1.4322 | 263.293 | TRUE | TRUE |
| CHEMBL538419 | O=C(O)[C@H]1N[C@@H]1C(=O)O | alpha | -1.504 | 131.087 | FALSE | TRUE |
| CHEMBL538546 | Cl.NC(CSc1cccc(O)c1O)C(=O)O | alpha | 1.0236 | 265.718 | TRUE | FALSE |
| CHEMBL538848 | CNC(CN=C(N)N)C(=O)O.Cl.Cl | alpha | -1.224 | 233.099 | FALSE | FALSE |
| CHEMBL539019 | Br.C[C@H](NCc1cc([N+](=O)[O-])cc2nc(O)c(O)nc12)C(=O)O | alpha | 1.0898 | 389.162 | TRUE | TRUE |
| CHEMBL539335 | C[C@H](N)P(=O)(O)CC[C@H](N)C(=O)O.Cl | alpha | -0.2147 | 246.631 | FALSE | TRUE |
| CHEMBL539877 | C[S+](CCc1ccccc1)CCC(N)C(=O)O.[I-] | alpha | -1.7168 | 381.279 | TRUE | FALSE |
| CHEMBL5400039 | Cc1ccc(S(=O)(=O)N[C@@H](CCCNC(=N)N)C(=O)O)cc1 | alpha | -0.01021 | 328.394 | TRUE | TRUE |
| CHEMBL540067 | Cl.N[C@@H](Cn1c(=O)c(=O)[nH]c2ccccc21)C(=O)O | alpha | -0.4765 | 285.687 | TRUE | TRUE |
| CHEMBL540208 | CCCCCC/C=C/[C@H](N)C(=O)O | alpha | 1.9249 | 185.267 | FALSE | TRUE |
| CHEMBL540219 | CC(=O)NC[C@@H](NS(=O)(=O)Cc1ccccc1)C(=O)O | alpha | -0.3047 | 300.336 | TRUE | TRUE |
| CHEMBL5404697 | N=C(N)NCC(C[C@H](NCc1ccccc1)C(=O)O)[N+](=O)[O-] | alpha | -0.25213 | 309.326 | TRUE | TRUE |
| CHEMBL5405359 | N[C@H](C[C@H]1CC(C(=O)O)=NN1c1ccc(CCCF)cc1)C(=O)O | alpha | 1.41 | 337.351 | TRUE | TRUE |
| CHEMBL5406958 | N[C@@H](CCCB(O)O)C(=O)O | alpha | -1.3487 | 160.966 | FALSE | TRUE |
| CHEMBL541604 | Cl.NC(CCCc1nnn[nH]1)C(=O)O | alpha | -0.6439 | 221.648 | TRUE | FALSE |
| CHEMBL541763 | NC(CNC(=O)c1ccco1)C(=O)O | alpha | -0.5787 | 198.178 | TRUE | FALSE |
| CHEMBL541929 | C[S+](C)CC(=O)CC[C@H](N)C(=O)O.O=C([O-])C(F)(F)F | alpha | -1.0759 | 319.301 | FALSE | TRUE |
| CHEMBL541956 | CC(=O)NC[C@@H](NS(=O)(=O)c1ccccc1)C(=O)O | alpha | -0.4458 | 286.309 | TRUE | TRUE |
| CHEMBL5420581 | CCCCCC(=N)NCCC[C@H](N)C(=O)O | alpha | 1.32567 | 229.324 | FALSE | TRUE |
| CHEMBL542245 | Cl.Cl.N[C@H]1CCN[C@H]1C(=O)O | alpha | -0.3962 | 203.069 | FALSE | TRUE |
| CHEMBL5422471 | CCC(=O)Nc1cccc(CO[C@H](C(=O)O)[C@H](N)C(=O)O)c1 | alpha | 0.4168 | 310.306 | TRUE | TRUE |
| CHEMBL54238 | N[C@@H](CC(C(=O)O)=C1CCCCC1)C(=O)O | alpha | 1.1337 | 227.26 | FALSE | TRUE |
| CHEMBL542649 | Cl.O=C(O)C1NCC12CCCCC2 | alpha | 1.4151 | 205.685 | FALSE | FALSE |
| CHEMBL54291 | CC1(C)CCC/C1=C(\C[C@H](N)C(=O)O)C(=O)O | alpha | 1.3797 | 241.287 | FALSE | TRUE |
| CHEMBL5430848 | CC(C)[C@@H]1CN[C@H](C(=O)O)[C@H]1CC(=O)O | alpha | 0.4059 | 215.249 | FALSE | TRUE |
| CHEMBL5432409 | Cl.N[C@H](Cc1ccc(=O)n(O)c1)C(=O)O | alpha | -0.5382 | 234.639 | TRUE | TRUE |
| CHEMBL543323 | Cl.NC(CS/C(S)=N/CCCc1ccccc1)C(=O)O | alpha | 2.4719 | 334.894 | TRUE | FALSE |
| CHEMBL54336 | O=C(O)[C@H]1CCN[C@@H]1C(=O)O | alpha | -0.8663 | 159.141 | FALSE | TRUE |
| CHEMBL543367 | Br.C[C@@H](NCc1cc([N+](=O)[O-])cc2nc(O)c(O)nc12)C(=O)O | alpha | 1.0898 | 389.162 | TRUE | TRUE |
| CHEMBL5434506 | Cl.N[C@@H](Cc1ccc(=O)n(O)c1)C(=O)O | alpha | -0.5382 | 234.639 | TRUE | TRUE |
| CHEMBL5436239 | Cl.NC(Cc1ccc(=O)n(O)c1)C(=O)O | alpha | -0.5382 | 234.639 | TRUE | FALSE |
| CHEMBL5441462 | CC(C)C[C@@H](Nc1ncnc2[nH]ccc12)C(=O)O | alpha | 1.8691 | 248.286 | TRUE | TRUE |
| CHEMBL5441541 | CCC[C@@H](Nc1ncnc2[nH]ccc12)C(=O)O | alpha | 1.6231 | 234.259 | TRUE | TRUE |
| CHEMBL5441655 | O=C(O)[C@@H](CC(F)F)Nc1ncnc2[nH]ccc12 | alpha | 1.4782 | 256.212 | TRUE | TRUE |
| CHEMBL5442127 | CCC[C@H](Nc1ncnc2[nH]ccc12)C(=O)O | alpha | 1.6231 | 234.259 | TRUE | TRUE |
| CHEMBL544390 | Cl.NC(CCCCc1nnn[nH]1)C(=O)O | alpha | -0.2538 | 235.675 | TRUE | FALSE |
| CHEMBL544391 | Cl.NC(CCCCCCc1nnn[nH]1)C(=O)O | alpha | 0.5264 | 263.729 | TRUE | FALSE |
| CHEMBL544447 | CC(Nc1cccc(C(=O)c2ccccc2)c1)C(=O)O.Cl | alpha | 3.2244 | 305.761 | TRUE | FALSE |
| CHEMBL544844 | C=C1CN[C@H](C(=O)O)C1.Cl | alpha | 0.4109 | 163.604 | FALSE | TRUE |
| CHEMBL545083 | Cl.N[C@H](Cn1c(=O)c(=O)[nH]c2ccccc21)C(=O)O | alpha | -0.4765 | 285.687 | TRUE | TRUE |
| CHEMBL545139 | Cl.NC(CCCCCCn1ccnc1)C(=O)O | alpha | 1.6673 | 261.753 | TRUE | FALSE |
| CHEMBL545314 | Cc1cc2[nH]c(=O)c(=O)n(C[C@@H](N)C(=O)O)c2cc1C.Cl | alpha | 0.14034 | 313.741 | TRUE | TRUE |
| CHEMBL545548 | Cc1cc2[nH]c(=O)c(=O)n(C[C@H](N)C(=O)O)c2cc1C.Cl | alpha | 0.14034 | 313.741 | TRUE | TRUE |
| CHEMBL546046 | CC(C)[C@H](NCC(=O)c1ccccc1)C(=O)O | alpha | 1.5681 | 235.283 | TRUE | TRUE |
| CHEMBL54634 | O=C(O)[C@H]1CCN[C@H]1C(=O)O | alpha | -0.8663 | 159.141 | FALSE | TRUE |
| CHEMBL5483662 | Cc1cc(C(NC(C)(C)C)C(=O)O)cc(C)c1O | alpha | 2.52284 | 251.326 | TRUE | FALSE |
| CHEMBL5486008 | CC1(C)SC(c2ccc([N+](=O)[O-])o2)N[C@H]1C(=O)O | alpha | 1.7546 | 272.282 | TRUE | TRUE |
| CHEMBL549578 | N#Cc1cccc2sc(C(=O)NCC(N)C(=O)O)cc12 | alpha | 0.91468 | 289.316 | TRUE | FALSE |
| CHEMBL549782 | NC(CNC(=O)c1cc2ccc(Cl)cc2[nH]1)C(=O)O | alpha | 0.963 | 281.699 | TRUE | FALSE |
| CHEMBL549783 | NC(CNC(=O)c1cc2cccc(Cl)c2[nH]1)C(=O)O | alpha | 0.963 | 281.699 | TRUE | FALSE |
| CHEMBL5499862 | NC(Cc1ccc(=O)n(O)c1)C(=O)O | alpha | -0.96 | 198.178 | TRUE | FALSE |
| CHEMBL5499956 | N[C@H](Cc1ccc(=O)n(O)c1)C(=O)O | alpha | -0.96 | 198.178 | TRUE | TRUE |
| CHEMBL5500014 | N[C@@H](Cc1ccc(=O)n(O)c1)C(=O)O | alpha | -0.96 | 198.178 | TRUE | TRUE |
| CHEMBL551265 | NC(CNC(=O)c1c[nH]c2ccccc12)C(=O)O | alpha | 0.3096 | 247.254 | TRUE | FALSE |
| CHEMBL551464 | NC(CNC(=O)c1cc2c(Cl)cccc2s1)C(=O)O | alpha | 1.6964 | 298.751 | TRUE | FALSE |
| CHEMBL551465 | COc1cccc2[nH]c(C(=O)NCC(N)C(=O)O)cc12 | alpha | 0.3182 | 277.28 | TRUE | FALSE |
| CHEMBL5517920 | C[C@@]1(C(=O)O)C[C@@H]1C(N)C(=O)O.Cl | alpha | -0.0691 | 209.629 | FALSE | TRUE |
| CHEMBL552223 | CCC/N=C\C=C1/C=C(C(=O)O)N[C@H](C(=O)O)C1 | alpha | 0.8086 | 252.27 | FALSE | TRUE |
| CHEMBL5523505 | C[C@@]1(C[C@H](N)C(=O)O)C[C@H]1C(=O)O | alpha | -0.1008 | 187.195 | FALSE | TRUE |
| CHEMBL552476 | NC(CNC(=O)c1cc2cc(Cl)ccc2[nH]1)C(=O)O | alpha | 0.963 | 281.699 | TRUE | FALSE |
| CHEMBL553028 | CC1CCC=C(C[C@H](N)C(=O)O)c2c(O)noc21 | alpha | 1.4629 | 252.27 | TRUE | TRUE |
| CHEMBL553200 | COc1ccccc1CC[S+](C)CCC(N)C(=O)O.[I-] | alpha | -1.7082 | 411.305 | TRUE | FALSE |
| CHEMBL5532032 | C[C@@]1(C(=O)O)C[C@H]1[C@H](N)C(=O)O.Cl | alpha | -0.0691 | 209.629 | FALSE | TRUE |
| CHEMBL553428 | NC(CNC(=O)c1cc2c(Cl)ccc(Cl)c2[nH]1)C(=O)O | alpha | 1.6164 | 316.144 | TRUE | FALSE |
| CHEMBL553701 | N[C@@H](/C=C/CCO)C(=O)O | alpha | -0.6631 | 145.158 | FALSE | TRUE |
| CHEMBL5537101 | C[C@@]1(C(=O)O)C[C@@H]1C[C@H](N)C(=O)O.Cl | alpha | 0.321 | 223.656 | FALSE | TRUE |
| CHEMBL553895 | Br.C[C@H](NCCc1cc([N+](=O)[O-])cc2nc(O)c(O)nc12)C(=O)O | alpha | 1.1323 | 403.189 | TRUE | TRUE |
| CHEMBL553901 | Cc1cc(C)c(S(=O)(=O)N[C@H](CN)C(=O)O)c(C)c1 | alpha | 0.30206 | 286.353 | TRUE | TRUE |
| CHEMBL554000 | Cl.NC(CC(=O)c1ccc(Cl)c(Cl)c1)C(=O)O | alpha | 2.3999 | 298.553 | TRUE | FALSE |
| CHEMBL554043 | Cl.NC(CS/C(S)=N/Cc1ccccc1)C(=O)O | alpha | 2.0393 | 306.84 | TRUE | FALSE |
| CHEMBL554071 | O=C(O)C1=C/C(=C\C=N/CCc2ccccc2)C[C@@H](C(=O)O)N1 | alpha | 1.6413 | 314.341 | TRUE | TRUE |
| CHEMBL5542987 | CC1(C[C@H](N)C(=O)O)CC1C(=O)O | alpha | -0.1008 | 187.195 | FALSE | TRUE |
| CHEMBL554460 | Br.CC[C@H](NCc1cc([N+](=O)[O-])cc2nc(O)c(O)nc12)C(=O)O | alpha | 1.4799 | 403.189 | TRUE | TRUE |
| CHEMBL554613 | Br.C[C@H](NCc1cc(Br)cc2nc(O)c(O)nc12)C(=O)O | alpha | 1.9441 | 423.061 | TRUE | TRUE |
| CHEMBL5555290 | CC[C@@]1(C(=O)O)C[C@H]1[C@H](N)C(=O)O.Cl | alpha | 0.321 | 223.656 | FALSE | TRUE |
| CHEMBL5557914 | CC[C@]1(C(=O)O)C[C@@H]1[C@H](N)C(=O)O.Cl | alpha | 0.321 | 223.656 | FALSE | TRUE |
| CHEMBL5558260 | C[C@@]1(C[C@H](N)C(=O)O)C[C@@H]1C(=O)O | alpha | -0.1008 | 187.195 | FALSE | TRUE |
| CHEMBL5558871 | C[C@]1(C[C@H](N)C(=O)O)C[C@@H]1C(=O)O | alpha | -0.1008 | 187.195 | FALSE | TRUE |
| CHEMBL5559238 | C[C@]1(C(=O)O)C[C@H]1C(N)C(=O)O.Cl | alpha | -0.0691 | 209.629 | FALSE | TRUE |
| CHEMBL5559557 | C[C@H](NC1=CC(=O)c2ccccc2C1=O)C(=O)O | alpha | 1.0122 | 245.234 | TRUE | TRUE |
| CHEMBL5559857 | C[C@]1(C[C@H](N)C(=O)O)C[C@H]1C(=O)O | alpha | -0.1008 | 187.195 | FALSE | TRUE |
| CHEMBL5560070 | C[C@]1(C(=O)O)C[C@@H]1[C@H](N)C(=O)O.Cl | alpha | -0.0691 | 209.629 | FALSE | TRUE |
| CHEMBL556075 | NC(CNC(=O)c1cc2c(Br)cccc2[nH]1)C(=O)O | alpha | 1.0721 | 326.15 | TRUE | FALSE |
| CHEMBL5560959 | Cc1ccc(S(=O)(=O)N[C@H](CCC(=O)NC(C)C)C(=O)O)cc1 | alpha | 1.03132 | 342.417 | TRUE | TRUE |
| CHEMBL5560982 | CCCCCC[C@@H](O)CCCCCCCC[C@@H](O)[C@H](N)C(=O)O | alpha | 3.2113 | 331.497 | FALSE | TRUE |
| CHEMBL5561001 | CNC(=O)CC[C@@H](NS(=O)(=O)c1ccc(C)cc1)C(=O)O | alpha | 0.25272 | 314.363 | TRUE | TRUE |
| CHEMBL5561771 | Cc1ccc(S(=O)(=O)N[C@H](CCC(N)=O)C(=O)O)cc1 | alpha | -0.00798 | 300.336 | TRUE | TRUE |
| CHEMBL5563067 | CCNC(=O)CC[C@@H](NS(=O)(=O)c1ccc(C)cc1)C(=O)O | alpha | 0.64282 | 328.39 | TRUE | TRUE |
| CHEMBL5563086 | Cl.N[C@@H](C[C@H]1C[C@@H]1C(=O)O)C(=O)O | alpha | -0.0691 | 209.629 | FALSE | TRUE |
| CHEMBL556337 | Cn1c(C(=O)NCC(N)C(=O)O)cc2ccccc21 | alpha | 0.32 | 261.281 | TRUE | FALSE |
| CHEMBL556423 | NC(CNC(=O)C1=Cc2ccccc2C1)C(=O)O | alpha | 0.1542 | 246.266 | TRUE | FALSE |
| CHEMBL556424 | NC(CNC(=O)Cc1c[nH]c2ccccc12)C(=O)O | alpha | 0.2385 | 261.281 | TRUE | FALSE |
| CHEMBL556425 | NC(CNC(=O)c1ccc2ccccc2c1)C(=O)O | alpha | 0.9815 | 258.277 | TRUE | FALSE |
| CHEMBL5565734 | N[C@H](C(=O)O)[C@H]1[C@@H](CO)[C@@H]1C(=O)O | alpha | -1.6626 | 189.167 | FALSE | TRUE |
| CHEMBL556841 | Cn1c(C(=O)NCC(N)C(=O)O)cc2c(Br)cccc21 | alpha | 1.0825 | 340.177 | TRUE | FALSE |
| CHEMBL556845 | CC(=O)NC[C@@H](NS(=O)(=O)c1c(C)cc(C)cc1C)C(=O)O | alpha | 0.47946 | 328.39 | TRUE | TRUE |
| CHEMBL556931 | CC(=NCCCC[C@H](N)C(=O)O)NO | alpha | -0.0342 | 203.242 | FALSE | TRUE |
| CHEMBL5570623 | C[C@@]1(C(=O)O)C[C@H]1[C@H](N)C(=O)O | alpha | -0.4909 | 173.168 | FALSE | TRUE |
| CHEMBL557328 | N[C@@H](Cc1ccc(O)cc1CC[18F])C(=O)O | alpha | 0.8586 | 226.237938 | TRUE | TRUE |
| CHEMBL557574 | C[C@@H](N)P(=O)(O)CC[C@H](N)C(=O)O.Cl | alpha | -0.2147 | 246.631 | FALSE | TRUE |
| CHEMBL55859 | CNC(N)NCCCC(N)C(=O)O | alpha | -1.7702 | 190.247 | FALSE | FALSE |
| CHEMBL558908 | NC(CNC(=O)C1Cc2ccccc2C1)C(=O)O | alpha | -0.0705 | 248.282 | TRUE | FALSE |
| CHEMBL558909 | NC(CNC(=O)c1cc2c(Br)cccc2s1)C(=O)O | alpha | 1.8055 | 343.202 | TRUE | FALSE |
| CHEMBL5592694 | Cl.O=C(O)C[C@@H]1[C@@H](C(=O)O)NC[C@@H]1n1nncc1CO | alpha | -1.1194 | 306.706 | TRUE | TRUE |
| CHEMBL5592807 | Cl.NCc1cnnn1[C@H]1CN[C@H](C(=O)O)[C@H]1CC(=O)O | alpha | -1.153 | 305.722 | TRUE | TRUE |
| CHEMBL5594630 | Cc1nc([C@H]2CN[C@H](C(=O)O)[C@H]2CC(=O)O)cs1 | alpha | 0.68242 | 270.31 | TRUE | TRUE |
| CHEMBL5594816 | Nc1nc([C@H]2CN[C@H](C(=O)O)[C@H]2CC(=O)O)cs1 | alpha | -0.0438 | 271.298 | TRUE | TRUE |
| CHEMBL5595349 | Cl.O=C(O)C[C@@H]1[C@@H](C(=O)O)NC[C@@H]1n1ccnn1 | alpha | -0.6117 | 276.68 | TRUE | TRUE |
| CHEMBL559933 | NC(C(=O)O)C1CC1 | alpha | -0.1917 | 115.132 | FALSE | FALSE |
| CHEMBL560127 | NC(CNC(=O)c1ccc[nH]1)C(=O)O | alpha | -0.8436 | 197.194 | TRUE | FALSE |
| CHEMBL560128 | NC(CNC(=O)Cc1c[nH]c2cccc(Br)c12)C(=O)O | alpha | 1.001 | 340.177 | TRUE | FALSE |
| CHEMBL560130 | NC(CNC(=O)c1cc2c(Cl)cccc2[nH]1)C(=O)O | alpha | 0.963 | 281.699 | TRUE | FALSE |
| CHEMBL5607633 | N[C@@H](Cc1ccc2ccccc2n1)C(=O)O | alpha | 1.1892 | 216.24 | TRUE | TRUE |
| CHEMBL5612562 | CSCCC(NS(=O)(=O)c1cccc2cccnc12)C(=O)O | alpha | 1.7194 | 340.426 | TRUE | FALSE |
| CHEMBL561385 | NC(CNC(=O)c1cc2ccccc2o1)C(=O)O | alpha | 0.5745 | 248.238 | TRUE | FALSE |
| CHEMBL561386 | NC(CNC(=O)c1ccc2c(Br)cccc2c1)C(=O)O | alpha | 1.744 | 337.173 | TRUE | FALSE |
| CHEMBL561668 | NC(CNC(=O)c1ccccn1)C(=O)O | alpha | -0.7767 | 209.205 | TRUE | FALSE |
| CHEMBL561669 | NC(CNC(=O)c1cccc2ccccc12)C(=O)O | alpha | 0.9815 | 258.277 | TRUE | FALSE |
| CHEMBL561688 | O=C(O)C1=C/C(=C\C=N/CCc2ccc(O)cc2)C[C@@H](C(=O)O)N1 | alpha | 1.3469 | 330.34 | TRUE | TRUE |
| CHEMBL561869 | CCCc1c(C(=O)NCC(N)C(=O)O)[nH]c2ccccc12 | alpha | 1.2621 | 289.335 | TRUE | FALSE |
| CHEMBL561996 | CC(=O)NC[C@H](NS(=O)(=O)c1c(C)cc(C)cc1C)C(=O)O | alpha | 0.47946 | 328.39 | TRUE | TRUE |
| CHEMBL562068 | O=C(NC[C@@H](NS(=O)(=O)c1cccnc1)C(=O)O)c1ccoc1 | alpha | -0.1639 | 339.329 | TRUE | TRUE |
| CHEMBL562088 | O=C(O)C1=C/C(=C\C=N/CCc2ccc(O)c(O)c2)C[C@@H](C(=O)O)N1 | alpha | 1.0525 | 346.339 | TRUE | TRUE |
| CHEMBL562590 | Cc1c(C(=O)NCC(N)C(=O)O)[nH]c2ccccc12 | alpha | 0.61802 | 261.281 | TRUE | FALSE |
| CHEMBL562800 | NC(CNC(=O)c1cccs1)C(=O)O | alpha | -0.1102 | 214.246 | TRUE | FALSE |
| CHEMBL562848 | NC(CNC(=O)c1cc2ccccc2s1)C(=O)O | alpha | 1.043 | 264.306 | TRUE | FALSE |
| CHEMBL56299 | O=C(O)[C@@H]1CSC(C(O)CO)N1 | alpha | -1.5447 | 193.224 | FALSE | TRUE |
| CHEMBL563629 | Cc1cccc2[nH]cc(CC(N)C(=O)O)c12 | alpha | 1.43072 | 218.256 | TRUE | FALSE |
| CHEMBL563662 | NC(CNC(=O)c1ccc2ccccc2n1)C(=O)O | alpha | 0.3765 | 259.265 | TRUE | FALSE |
| CHEMBL563982 | N[C@H](CNC(=O)c1cc2ccccc2[nH]1)C(=O)O | alpha | 0.3096 | 247.254 | TRUE | TRUE |
| CHEMBL564352 | O=C(O)[C@@H]1CSC(c2cc(Cl)ccc2O)N1 | alpha | 1.8338 | 259.714 | TRUE | TRUE |
| CHEMBL564584 | N[C@@H](CNC(=O)c1cc2ccccc2[nH]1)C(=O)O | alpha | 0.3096 | 247.254 | TRUE | TRUE |
| CHEMBL564805 | O=C(O)C1=C/C(=C\C=N/CCc2cccc(O)c2)C[C@@H](C(=O)O)N1 | alpha | 1.3469 | 330.34 | TRUE | TRUE |
| CHEMBL56689 | NC(CCC(=O)CP(=O)(O)O)C(=O)O | alpha | -1.0747 | 225.137 | FALSE | FALSE |
| CHEMBL569487 | Nc1ncnc2c1ncn2CC(N)C(=O)O | alpha | -1.1796 | 222.208 | TRUE | FALSE |
| CHEMBL571004 | CCC/C=C/[C@H](N)C(=O)O | alpha | 0.7546 | 143.186 | FALSE | TRUE |
| CHEMBL571005 | N[C@H](CNC(=O)c1cc2c(Br)cccc2[nH]1)C(=O)O | alpha | 1.0721 | 326.15 | TRUE | TRUE |
| CHEMBL57111 | N/N=C(/S)NCCC[C@H](N)C(=O)O | alpha | -1.0724 | 206.271 | FALSE | TRUE |
| CHEMBL57164 | CS/C(=N/O)NCCC[C@H](N)C(=O)O | alpha | -0.1237 | 221.282 | FALSE | TRUE |
| CHEMBL5723327 | Cl.N[C@@H](CCCCB(O)O)C(=O)O | alpha | -0.5368 | 211.454 | FALSE | TRUE |
| CHEMBL573395 | O=C(O)[C@@H]1CSC(c2ccc(Br)cc2)N1 | alpha | 2.2373 | 288.166 | TRUE | TRUE |
| CHEMBL573396 | O=C(O)[C@@H]1CSC(c2cc(Br)ccc2O)N1 | alpha | 1.9429 | 304.165 | TRUE | TRUE |
| CHEMBL573641 | O=C(O)[C@@H]1CSC(c2cc(F)ccc2O)N1 | alpha | 1.3195 | 243.259 | TRUE | TRUE |
| CHEMBL573784 | CC(C)=CCc1cc(C[C@H](N)C(=O)O)ccc1O | alpha | 1.8553 | 249.31 | TRUE | TRUE |
| CHEMBL5746157 | Cc1cnc2cc(C(=O)NC[C@@H](N)C(=O)O)sc2c1C | alpha | 1.05484 | 293.348 | TRUE | TRUE |
| CHEMBL576775 | CC(C)(C)c1cc(C2N[C@H](C(=O)O)CS2)cc(C(C)(C)C)c1O | alpha | 3.7754 | 337.485 | TRUE | TRUE |
| CHEMBL5774300 | O=C(O)[C@H](NS(=O)(=O)c1ccc(O)c(O)c1)c1ccccc1 | alpha | 1.202 | 323.326 | TRUE | TRUE |
| CHEMBL577683 | COc1ccc(C2N[C@H](C(=O)O)CS2)cc1 | alpha | 1.4834 | 239.296 | TRUE | TRUE |
| CHEMBL5777291 | N[C@@H](Cc1c[nH]c2c(Cl)c(F)ccc12)C(=O)O | alpha | 1.9148 | 256.664 | TRUE | TRUE |
| CHEMBL5782535 | CCOc1ccnc2cc(C(=O)NC[C@@H](N)C(=O)O)sc12 | alpha | 0.8367 | 309.347 | TRUE | TRUE |
| CHEMBL5789690 | N[C@H](CNC(=O)c1cc2nccc(C(F)(F)F)c2s1)C(=O)O | alpha | 1.4568 | 333.291 | TRUE | TRUE |
| CHEMBL5794524 | CCc1ccnc2cc(C(=O)NC[C@@H](N)C(=O)O)sc12 | alpha | 1.0004 | 293.348 | TRUE | TRUE |
| CHEMBL57955 | O=C(O)C1CCc2c(Cl)cc(Cl)cc2N1 | alpha | 2.8047 | 246.093 | TRUE | FALSE |
| CHEMBL5801446 | O=C(O)[C@H](Cc1ccccc1)NS(=O)(=O)c1ccc(O)c(O)c1 | alpha | 1.072 | 337.353 | TRUE | TRUE |
| CHEMBL58027 | CCCCC[C@H](C[C@H](N)C(=O)O)C(=O)O | alpha | 1.0695 | 217.265 | FALSE | TRUE |
| CHEMBL58054 | N[C@@H](C[C@@H](C/C=C/c1cccc(Cl)c1)C(=O)O)C(=O)O | alpha | 2.2461 | 297.738 | TRUE | TRUE |
| CHEMBL5806540 | N[C@H](CNC(=O)c1cc2nccc(C(F)F)c2s1)C(=O)O | alpha | 1.3756 | 315.301 | TRUE | TRUE |
| CHEMBL58095 | N[C@@H](C[C@@H](C/C=C/c1ccccc1)C(=O)O)C(=O)O | alpha | 1.5927 | 263.293 | TRUE | TRUE |
| CHEMBL58129 | N[C@@H](Cc1cccc(-c2ccccc2)c1)C(=O)O | alpha | 2.308 | 241.29 | TRUE | TRUE |
| CHEMBL58163 | O=C1CC(C(=O)O)Nc2cc(Cl)ccc21 | alpha | 1.7915 | 225.631 | TRUE | FALSE |
| CHEMBL58164 | O=C1CC(C(=O)O)Nc2cccc(Cl)c21 | alpha | 1.7915 | 225.631 | TRUE | FALSE |
| CHEMBL58171 | O=C(O)C[C@H]1C[C@@H](C(=O)O)Nc2cc(Cl)cc(Cl)c21 | alpha | 2.8205 | 304.129 | TRUE | TRUE |
| CHEMBL58229 | O=C(O)[C@H](CO)NC/C=C/c1cccc(Oc2ccccc2)c1 | alpha | 2.5272 | 313.353 | TRUE | TRUE |
| CHEMBL582332 | NC(CC1CC(=O)N1CC(=O)O)C(=O)O | alpha | -1.5261 | 216.193 | FALSE | FALSE |
| CHEMBL58247 | C[C@H](Nc1ccc(C(=O)O)cc1)C(=O)O | alpha | 1.2698 | 209.201 | TRUE | TRUE |
| CHEMBL5831924 | N[C@H](CNC(=O)c1cc2nccc(CO)c2s1)C(=O)O | alpha | -0.0697 | 295.32 | TRUE | TRUE |
| CHEMBL5835774 | CC1CC(C(=O)O)NN1 | alpha | -0.674 | 130.147 | FALSE | FALSE |
| CHEMBL58365 | N[C@H](Cc1cc(CP(=O)(O)O)cc(-c2ccccc2)c1)C(=O)O | alpha | 1.9857 | 335.296 | TRUE | TRUE |
| CHEMBL58368 | Cc1cc(C)c2c(c1)N[C@@H](C(=O)O)[C@H](C)C2=O | alpha | 2.00094 | 233.267 | TRUE | TRUE |
| CHEMBL58404 | O=C1CC(C(=O)O)Nc2ccccc21 | alpha | 1.1381 | 191.186 | TRUE | FALSE |
| CHEMBL584591 | O=C(O)[C@@H]1CSC(c2ccc(O)cc2)N1 | alpha | 1.1804 | 225.269 | TRUE | TRUE |
| CHEMBL58541 | CCC#CC[C@H](C[C@H](N)C(=O)O)C(=O)O | alpha | 0.2927 | 213.233 | FALSE | TRUE |
| CHEMBL58586 | O=C1CC(C(=O)O)Nc2cc(Cl)cc(Cl)c21 | alpha | 2.4449 | 260.076 | TRUE | FALSE |
| CHEMBL5858619 | CC(C)Oc1ccnc2cc(C(=O)NC[C@@H](N)C(=O)O)sc12 | alpha | 1.2252 | 323.374 | TRUE | TRUE |
| CHEMBL58590 | N[C@@H](C[C@@H](C/C=C/c1ccc(Cl)cc1)C(=O)O)C(=O)O | alpha | 2.2461 | 297.738 | TRUE | TRUE |
| CHEMBL586418 | O=CNC(CSC(c1ccccc1)c1ccccc1)C(=O)O | alpha | 2.7084 | 315.394 | TRUE | FALSE |
| CHEMBL5869131 | N[C@H](CNC(=O)c1cc2ncccc2s1)C(=O)O | alpha | 0.438 | 265.294 | TRUE | TRUE |
| CHEMBL58718 | C#CC[C@@H](C[C@H](N)C(=O)O)C(=O)O | alpha | -0.4875 | 185.179 | FALSE | TRUE |
| CHEMBL5879133 | O=C(O)[C@@H](Cc1ccccc1)NS(=O)(=O)c1ccc(O)c(O)c1 | alpha | 1.072 | 337.353 | TRUE | TRUE |
| CHEMBL5879275 | COc1ccnc2cc(C(=O)NC[C@@H](N)C(=O)O)sc12 | alpha | 0.4466 | 295.32 | TRUE | TRUE |
| CHEMBL58808 | N[C@@H](C[C@@H](C/C=C\c1ccccc1)C(=O)O)C(=O)O | alpha | 1.5927 | 263.293 | TRUE | TRUE |
| CHEMBL589116 | COC(C)c1onc(C(=O)O)c1C[C@H](N)C(=O)O | alpha | 0.0346 | 258.23 | TRUE | TRUE |
| CHEMBL59011 | CC(=O)N[C@H]1C[C@H](C(=O)O)Nc2cc(Cl)cc(Cl)c21 | alpha | 2.4394 | 303.145 | TRUE | TRUE |
| CHEMBL5903643 | N[C@H](CNC(=O)c1cc2nccc(CF)c2s1)C(=O)O | alpha | 0.9075 | 297.311 | TRUE | TRUE |
| CHEMBL5913368 | Cc1cccc(Nc2nc(NCC(N)C(=O)O)ncc2C(N)=O)c1 | alpha | 0.45132 | 330.348 | TRUE | FALSE |
| CHEMBL591745 | N[C@@H](Cc1c(C(=O)O)noc1COCc1ccccc1)C(=O)O | alpha | 1.044 | 320.301 | TRUE | TRUE |
| CHEMBL591768 | COCc1onc(C(=O)O)c1C[C@H](N)C(=O)O | alpha | -0.5264 | 244.203 | TRUE | TRUE |
| CHEMBL591769 | N[C@@H](Cc1c(C(=O)O)noc1CCc1ccccc1)C(=O)O | alpha | 1.1124 | 304.302 | TRUE | TRUE |
| CHEMBL59284 | COc1ccccc1/C=C/C[C@H](C[C@H](N)C(=O)O)C(=O)O | alpha | 1.6013 | 293.319 | TRUE | TRUE |
| CHEMBL59286 | CC(C)=CC[C@H](C[C@H](N)C(=O)O)C(=O)O | alpha | 0.8455 | 215.249 | FALSE | TRUE |
| CHEMBL59297 | N[C@@H](C[C@@H](C/C=C/c1c(Cl)cccc1Cl)C(=O)O)C(=O)O | alpha | 2.8995 | 332.183 | TRUE | TRUE |
| CHEMBL59299 | COc1ccc(/C=C/C[C@H](C[C@H](N)C(=O)O)C(=O)O)cc1 | alpha | 1.6013 | 293.319 | TRUE | TRUE |
| CHEMBL593408 | NC(CCSC[C@@H]1C[C@@H](O)C(O)O1)C(=O)O | alpha | -1.0102 | 251.304 | FALSE | TRUE |
| CHEMBL5934769 | N[C@H](CNC(=O)c1cc2nccc(Br)c2s1)C(=O)O | alpha | 1.2005 | 344.19 | TRUE | TRUE |
| CHEMBL593753 | NC(Cc1cccc(-c2ccccc2C(=O)O)c1)C(=O)O | alpha | 2.0062 | 285.299 | TRUE | FALSE |
| CHEMBL594416 | NC(Cc1cccc(-c2ccc(CC(=O)O)cc2)c1)C(=O)O | alpha | 1.9351 | 299.326 | TRUE | FALSE |
| CHEMBL594636 | N[C@@H](Cc1cccc(-c2cccc(C(=O)O)c2)c1)C(=O)O | alpha | 2.0062 | 285.299 | TRUE | TRUE |
| CHEMBL594637 | N[C@H](Cc1cccc(-c2cccc(C(=O)O)c2)c1)C(=O)O | alpha | 2.0062 | 285.299 | TRUE | TRUE |
| CHEMBL594642 | NC(Cc1cc(Cl)c(Cl)c(-c2ccccc2O)c1)C(=O)O | alpha | 3.3204 | 326.179 | TRUE | FALSE |
| CHEMBL594842 | NC(Cc1cccc(Cc2ccccc2C(=O)O)c1)C(=O)O | alpha | 1.93 | 299.326 | TRUE | FALSE |
| CHEMBL59551 | C#CC[C@H](C[C@H](N)C(=O)O)C(=O)O | alpha | -0.4875 | 185.179 | FALSE | TRUE |
| CHEMBL595811 | NC(Cc1cc(Cl)c(Cl)c(-c2ccc(O)cc2)c1)C(=O)O | alpha | 3.3204 | 326.179 | TRUE | FALSE |
| CHEMBL596045 | NC(Cc1cccc(-c2ccc(C(=O)O)cc2)c1)C(=O)O | alpha | 2.0062 | 285.299 | TRUE | FALSE |
| CHEMBL596497 | NC[C@H](N)C(=O)NC[C@@H](N)C(=O)O | alpha | -3.1995 | 190.203 | FALSE | TRUE |
| CHEMBL59792 | N[C@@H](C[C@@H](CC#Cc1ccccc1)C(=O)O)C(=O)O | alpha | 0.931 | 261.277 | TRUE | TRUE |
| CHEMBL5982066 | Cc1c(F)cnc2cc(C(=O)NC[C@@H](N)C(=O)O)sc12 | alpha | 0.88552 | 297.311 | TRUE | TRUE |
| CHEMBL598756 | N[C@H](Cc1cccc(C2(C(F)(F)F)N=N2)c1)C(=O)O | alpha | 1.8219 | 273.214 | TRUE | TRUE |
| CHEMBL59903 | CCC[Se]C[C@H](N)C(=O)O | alpha | 0.3491 | 210.135 | FALSE | TRUE |
| CHEMBL59948 | COc1ccc(/C=C/C[C@H](C[C@H](N)C(=O)O)C(=O)O)cc1OC | alpha | 1.6099 | 323.345 | TRUE | TRUE |
| CHEMBL60062 | N[C@H](C(=O)O)[C@H]1[C@H](C(=O)O)C1(F)F | alpha | -0.6358 | 195.121 | FALSE | TRUE |
| CHEMBL601047 | N[C@H](Cc1ccc(C2(C(F)(F)F)N=N2)cc1)C(=O)O | alpha | 1.8219 | 273.214 | TRUE | TRUE |
| CHEMBL60114 | N[C@@H](C[C@@H](C/C=C/c1ccc(OC(F)(F)F)cc1)C(=O)O)C(=O)O | alpha | 2.4913 | 347.289 | TRUE | TRUE |
| CHEMBL60168 | N[C@@H](C[C@@H](C/C=C/c1ccc([N+](=O)[O-])cc1)C(=O)O)C(=O)O | alpha | 1.5009 | 308.29 | TRUE | TRUE |
| CHEMBL6023630 | O=C(O)[C@@H](NS(=O)(=O)c1ccc(O)c(O)c1)c1ccccc1 | alpha | 1.202 | 323.326 | TRUE | TRUE |
| CHEMBL602590 | NC(Cc1cccc(-c2cccc(CC(=O)O)c2)c1)C(=O)O | alpha | 1.9351 | 299.326 | TRUE | FALSE |
| CHEMBL602640 | CCc1onc(C(=O)O)c1C[C@H](N)C(=O)O | alpha | -0.1104 | 228.204 | TRUE | TRUE |
| CHEMBL6032507 | CC(C)c1ccnc2cc(C(=O)NC[C@@H](N)C(=O)O)sc12 | alpha | 1.5614 | 307.375 | TRUE | TRUE |
| CHEMBL60398 | O=C(O)[C@H]1CCc2c(Cl)cc(Cl)cc2N1 | alpha | 2.8047 | 246.093 | TRUE | TRUE |
| CHEMBL60406 | N[C@@H](C[C@@H](C/C=C/Cl)C(=O)O)C(=O)O | alpha | 0.6318 | 221.64 | FALSE | TRUE |
| CHEMBL60652 | O=C(O)[C@H]1C[C@H](C(=O)O)Nc2cc(Cl)cc(Cl)c21 | alpha | 2.4304 | 290.102 | TRUE | TRUE |
| CHEMBL6068327 | CC(=O)N[C@@H](CS)C(=O)O.NCCCC[C@H](N)C(=O)O | alpha | -0.9672 | 309.388 | FALSE | TRUE |
| CHEMBL6068712 | C[C@@]1(C(=O)O)C[C@@H]1C(N)C(=O)O | alpha | -0.4909 | 173.168 | FALSE | TRUE |
| CHEMBL6068787 | NCc1cnnn1[C@H]1CN[C@H](C(=O)O)[C@H]1CC(=O)O | alpha | -1.5748 | 269.261 | TRUE | TRUE |
| CHEMBL6068907 | O=C(O)C[C@@H]1[C@@H](C(=O)O)NC[C@@H]1n1nncc1CO | alpha | -1.5412 | 270.245 | TRUE | TRUE |
| CHEMBL6068990 | O=C(O)C[C@@H]1[C@@H](C(=O)O)NC[C@@H]1n1ccnn1 | alpha | -1.0335 | 240.219 | TRUE | TRUE |
| CHEMBL6069232 | C[C@@]1(C(=O)O)C[C@@H]1C[C@H](N)C(=O)O | alpha | -0.1008 | 187.195 | FALSE | TRUE |
| CHEMBL6069263 | C[C@]1(C(=O)O)C[C@H]1C(N)C(=O)O | alpha | -0.4909 | 173.168 | FALSE | TRUE |
| CHEMBL6069490 | CC[C@]1(C(=O)O)C[C@@H]1[C@H](N)C(=O)O | alpha | -0.1008 | 187.195 | FALSE | TRUE |
| CHEMBL6069604 | N[C@@H](C[C@H]1C[C@@H]1C(=O)O)C(=O)O | alpha | -0.4909 | 173.168 | FALSE | TRUE |
| CHEMBL6069725 | C[C@]1(C(=O)O)C[C@@H]1[C@H](N)C(=O)O | alpha | -0.4909 | 173.168 | FALSE | TRUE |
| CHEMBL6070548 | CC[C@@]1(C(=O)O)C[C@H]1[C@H](N)C(=O)O | alpha | -0.1008 | 187.195 | FALSE | TRUE |
| CHEMBL608107 | NC(Cc1cccc(Cc2ccc(C(=O)O)cc2)c1)C(=O)O | alpha | 1.93 | 299.326 | TRUE | FALSE |
| CHEMBL609458 | CSCCC(Nc1ccc([N+](=O)[O-])cc1[N+](=O)[O-])C(=O)O | alpha | 2.1212 | 315.307 | TRUE | FALSE |
| CHEMBL609711 | CC(C)CC(Nc1ccc([N+](=O)[O-])cc1[N+](=O)[O-])C(=O)O | alpha | 2.4142 | 297.267 | TRUE | FALSE |
| CHEMBL609716 | CC(C)C(Nc1ccc([N+](=O)[O-])cc1[N+](=O)[O-])C(=O)O | alpha | 2.0241 | 283.24 | TRUE | FALSE |
| CHEMBL609726 | O=C(O)C(Cc1ccc(O)cc1)NSc1ccccc1[N+](=O)[O-] | alpha | 2.593 | 334.353 | TRUE | FALSE |
| CHEMBL609876 | NC(Cc1cccc(-c2cccc(C(=O)O)c2)c1)C(=O)O | alpha | 2.0062 | 285.299 | TRUE | FALSE |
| CHEMBL609969 | CC(Nc1ccc([N+](=O)[O-])cc1[N+](=O)[O-])C(=O)O | alpha | 1.388 | 255.186 | TRUE | FALSE |
| CHEMBL609970 | O=C(O)C(Cc1ccccc1)Nc1ccc([N+](=O)[O-])cc1[N+](=O)[O-] | alpha | 2.6108 | 331.284 | TRUE | FALSE |
| CHEMBL610746 | NC(Cc1cccc(Cc2cccc(C(=O)O)c2)c1)C(=O)O | alpha | 1.93 | 299.326 | TRUE | FALSE |
| CHEMBL611036 | NC(Cc1cc(Cl)c(Cl)c(-c2cccc(O)c2)c1)C(=O)O | alpha | 3.3204 | 326.179 | TRUE | FALSE |
| CHEMBL61217 | COc1ccc(C[Se]C[C@H](N)C(=O)O)cc1 | alpha | 0.7296 | 288.205 | TRUE | TRUE |
| CHEMBL61307 | C/C=C\C[C@H](C[C@H](N)C(=O)O)C(=O)O | alpha | 0.4554 | 201.222 | FALSE | TRUE |
| CHEMBL61502 | O=C1CC(C(=O)O)Nc2cc(Br)cc(Br)c21 | alpha | 2.6631 | 348.978 | TRUE | FALSE |
| CHEMBL61605 | N[C@H](C(=O)O)[C@@H]1[C@@H](C(=O)O)C1(F)F | alpha | -0.6358 | 195.121 | FALSE | TRUE |
| CHEMBL61697 | C/C(=C\c1ccccc1)C[C@H](C[C@H](N)C(=O)O)C(=O)O | alpha | 1.9828 | 277.32 | TRUE | TRUE |
| CHEMBL61751 | N[C@@H](C[C@@H](C/C=C/c1cccc2ccccc12)C(=O)O)C(=O)O | alpha | 2.7459 | 313.353 | TRUE | TRUE |
| CHEMBL61997 | CCC[C@H](C[C@H](N)C(=O)O)C(=O)O | alpha | 0.2893 | 189.211 | FALSE | TRUE |
| CHEMBL62053 | CCCCCC[C@H](C[C@H](N)C(=O)O)C(=O)O | alpha | 1.4596 | 231.292 | FALSE | TRUE |
| CHEMBL62055 | C=CC[C@@H](C[C@H](N)C(=O)O)C(=O)O | alpha | 0.0653 | 187.195 | FALSE | TRUE |
| CHEMBL62057 | C=CC[C@H](C[C@H](N)C(=O)O)C(=O)O | alpha | 0.0653 | 187.195 | FALSE | TRUE |
| CHEMBL62086 | CC#CC[C@H](C[C@H](N)C(=O)O)C(=O)O | alpha | -0.0974 | 199.206 | FALSE | TRUE |
| CHEMBL62117 | COc1ccc([Se]C[C@H](N)C(=O)O)cc1 | alpha | -0.1451 | 274.178 | TRUE | TRUE |
| CHEMBL62214 | N[C@H](C(=O)O)[C@H]1[C@@H](C(=O)O)C1(F)F | alpha | -0.6358 | 195.121 | FALSE | TRUE |
| CHEMBL62250 | N[C@H]1C[C@H](C(=O)O)Nc2cc(Cl)cc(Cl)c21 | alpha | 2.262 | 261.108 | TRUE | TRUE |
| CHEMBL62382 | C[Se]C[C@H](N)C(=O)O | alpha | -0.4311 | 182.081 | FALSE | TRUE |
| CHEMBL6251 | N[C@@H](CCC(=O)Nc1ccc([N+](=O)[O-])cc1)C(=O)O | alpha | 0.7254 | 267.241 | TRUE | TRUE |
| CHEMBL62587 | N[C@@H](C[Se]c1ccccc1)C(=O)O | alpha | -0.1537 | 244.152 | TRUE | TRUE |
| CHEMBL62628 | N=C(N)c1ccc(C2=NOC(CC(=O)NC[C@H](N)C(=O)O)C2)cc1.O=C(O)C(F)(F)F | alpha | 0.01517 | 447.37 | TRUE | TRUE |
| CHEMBL62755 | N[C@@H](C[Se]Cc1ccccc1)C(=O)O | alpha | 0.721 | 258.179 | TRUE | TRUE |
| CHEMBL62915 | CCCC[Se]C[C@H](N)C(=O)O | alpha | 0.7392 | 224.162 | FALSE | TRUE |
| CHEMBL63023 | Cc1ccc(C[Se]C[C@H](N)C(=O)O)cc1 | alpha | 1.02942 | 272.206 | TRUE | TRUE |
| CHEMBL63037 | N[C@@H](C[Se]c1ccc(Cl)cc1)C(=O)O | alpha | 0.4997 | 278.597 | TRUE | TRUE |
| CHEMBL63062 | N[C@@H](CSc1ccccc1)C(=O)O | alpha | 1.1906 | 197.259 | TRUE | TRUE |
| CHEMBL63228 | O=C(O)[C@H](Cc1ccccc1)NCc1ccccc1 | alpha | 2.4721 | 255.317 | TRUE | TRUE |
| CHEMBL64242 | Cc1ccc(CSC[C@H](N)C(=O)O)cc1 | alpha | 1.64012 | 225.313 | TRUE | TRUE |
| CHEMBL64353 | Cc1ccc([Se]C[C@H](N)C(=O)O)cc1 | alpha | 0.15472 | 258.179 | TRUE | TRUE |
| CHEMBL65631 | O=C(O)C1CC2CC(CP(=O)(O)O)CCC2CN1 | alpha | 0.6431 | 277.257 | FALSE | FALSE |
| CHEMBL67160 | C[C@@H](O)[C@H]1C(=O)N2C(C(=O)O)=C(CSC[C@@H](N)C(=O)O)S[C@H]12 | alpha | -0.6602 | 348.402 | FALSE | TRUE |
| CHEMBL67244 | O=C(O)C1CC(CP(=O)(O)O)N=CN1 | alpha | -0.9925 | 222.137 | FALSE | FALSE |
| CHEMBL67332 | NC(CCCC1(C(=O)O)CN1)C(=O)O | alpha | -1.0048 | 202.21 | FALSE | FALSE |
| CHEMBL67689 | O=C(CP(=O)(O)O)C1=CCCN[C@H]1C(=O)O | alpha | -0.8939 | 249.159 | FALSE | TRUE |
| CHEMBL67775 | NC(CCCP(=O)(O)O)CC(N)C(=O)O | alpha | -0.9264 | 240.196 | FALSE | FALSE |
| CHEMBL68347 | O=C(O)C1CC(CCCP(=O)(O)O)N=CN1 | alpha | -0.2123 | 250.191 | FALSE | FALSE |
| CHEMBL68517 | O=C(O)[C@H]1C[C@@H](CCCP(=O)(O)O)CCN1 | alpha | 0.3971 | 251.219 | FALSE | TRUE |
| CHEMBL68623 | C[C@@H](O)[C@H]1C(=O)N2C(C(=O)O)=C(CC[C@H](N)C(=O)O)S[C@H]12 | alpha | -0.6132 | 316.335 | FALSE | TRUE |
| CHEMBL68966 | O=C(O)[C@H]1C[C@H](CCCP(=O)(O)O)CCN1 | alpha | 0.3971 | 251.219 | FALSE | TRUE |
| CHEMBL69740 | C[C@@H](O)[C@H]1C(=O)N2C(C(=O)O)=C(SC[C@H](N)C(=O)O)S[C@H]12 | alpha | -0.7027 | 334.375 | FALSE | TRUE |
| CHEMBL70725 | CC(C)C[C@H](NCC(S)[C@@H](N)Cc1ccccc1)C(=O)O | alpha | 1.9437 | 310.463 | TRUE | TRUE |
| CHEMBL72157 | NC(C(=O)O)[C@@H]1[C@H](C(=O)O)[C@H]1c1ccccc1 | alpha | 0.5127 | 235.239 | TRUE | TRUE |
| CHEMBL72366 | NC(Cc1onc(O)c1Cl)C(=O)O | alpha | -0.012 | 206.585 | TRUE | FALSE |
| CHEMBL72573 | O=C(O)CC[C@H]1CC[C@H]2CN[C@H](C(=O)O)C[C@H]2C1 | alpha | 1.3302 | 255.314 | FALSE | TRUE |
| CHEMBL72592 | O=C(O)[C@@H]1C[C@H]2C[C@H](CCP(=O)(O)O)CC[C@H]2CN1 | alpha | 1.0332 | 291.284 | FALSE | TRUE |
| CHEMBL73837 | O=C(O)[C@@H]1C[C@H]2C[C@@H](CCS(=O)(=O)O)CC[C@H]2CN1 | alpha | 0.7433 | 291.369 | FALSE | TRUE |
| CHEMBL7471 | CCC(NC(=O)CCC(N)C(=O)O)C(=O)O | alpha | -0.8421 | 232.236 | FALSE | FALSE |
| CHEMBL74994 | N[C@@H](CC[C@H](F)[C@@H](N)C(=O)O)C(=O)O | alpha | -1.0715 | 208.189 | FALSE | TRUE |
| CHEMBL7503 | NC(CCC(=O)N1CCCC1C(=O)O)C(=O)O | alpha | -0.7459 | 244.247 | FALSE | FALSE |
| CHEMBL75191 | O=C(O)[C@@H]1CCC[C@@H](C(=O)O)N1 | alpha | -0.3337 | 173.168 | FALSE | TRUE |
| CHEMBL7521 | NC(CCC(=O)Nc1ccccc1O)C(=O)O | alpha | 0.5228 | 238.243 | TRUE | FALSE |
| CHEMBL75577 | N[C@@H](CC[C@@H](F)[C@@H](N)C(=O)O)C(=O)O | alpha | -1.0715 | 208.189 | FALSE | TRUE |
| CHEMBL75640 | C=C[C@H]1[C@H](C(=O)O)[C@@H]1[C@H](N)C(=O)O | alpha | -0.4689 | 185.179 | FALSE | TRUE |
| CHEMBL75846 | N[C@H](C(=O)O)[C@H](F)CC[C@H](N)C(=O)O | alpha | -1.0715 | 208.189 | FALSE | TRUE |
| CHEMBL7596 | NC(CCC(=O)N1CSCC1C(=O)O)C(=O)O | alpha | -0.8354 | 262.287 | FALSE | FALSE |
| CHEMBL75991 | N[C@H](C(=O)O)[C@@H](F)CC[C@H](N)C(=O)O | alpha | -1.0715 | 208.189 | FALSE | TRUE |
| CHEMBL76117 | CC(C)/N=C(\NO)NCCC[C@H](N)C(=O)O | alpha | -0.4887 | 232.284 | FALSE | TRUE |
| CHEMBL76397 | C/C=C/CCC(N)C(=O)O | alpha | 0.7546 | 143.186 | FALSE | FALSE |
| CHEMBL7730 | NC(CCC(=O)Nc1cccc(O)c1)C(=O)O | alpha | 0.5228 | 238.243 | TRUE | FALSE |
| CHEMBL78340 | CC(C)CCC(N[C@@H](C)C(=O)N1CCC[C@H]1C(=O)O)C(=O)O | alpha | 0.9295 | 314.382 | FALSE | TRUE |
| CHEMBL78920 | O=C(O)[C@@H]1C=CCN1 | alpha | -0.401 | 113.116 | FALSE | TRUE |
| CHEMBL79212 | C[C@H](NC(CCN)C(=O)O)C(=O)N1CCC[C@H]1C(=O)O | alpha | -1.1579 | 287.316 | FALSE | TRUE |
| CHEMBL80376 | C=C1C[C@@H]([C@H](N)C(=O)O)[C@@H](C(=O)O)C1 | alpha | 0.0653 | 199.206 | FALSE | TRUE |
| CHEMBL80628 | COC[C@H]1[C@H](C(=O)O)[C@@H]1[C@H](N)C(=O)O | alpha | -1.0085 | 203.194 | FALSE | TRUE |
| CHEMBL8137 | NC(/C=C(\Cl)CC(N)C(=O)O)C(=O)O | alpha | -0.677 | 222.628 | FALSE | FALSE |
| CHEMBL8185 | C=C(CC(N)C(=O)O)CC(N)C(=O)O | alpha | -0.8534 | 202.21 | FALSE | FALSE |
| CHEMBL82172 | N[C@@H](CCc1nc(C(=O)O)co1)C(=O)O | alpha | -0.2827 | 214.177 | TRUE | TRUE |
| CHEMBL82406 | N[C@@H](Cc1c(O)noc1-c1ccccn1)C(=O)O | alpha | 0.3966 | 249.226 | TRUE | TRUE |
| CHEMBL82935 | N[C@@H](CCC1=N[C@H](C(=O)O)CO1)C(=O)O | alpha | -0.9396 | 216.193 | FALSE | TRUE |
| CHEMBL83122 | NC(CC1CCC(CP(=O)(O)O)CC1)C(=O)O | alpha | 0.7725 | 265.246 | FALSE | FALSE |
| CHEMBL8326 | C/C(=C/C(N)C(=O)O)CC(N)C(=O)O | alpha | -0.8534 | 202.21 | FALSE | FALSE |
| CHEMBL83720 | N[C@@H](Cc1c(O)noc1-c1ccccc1)C(=O)O | alpha | 1.0016 | 248.238 | TRUE | TRUE |
| CHEMBL8396 | CC(N)(/C=C/CC(N)C(=O)O)C(=O)O | alpha | -0.8534 | 202.21 | FALSE | FALSE |
| CHEMBL84114 | N[C@H](Cc1c(O)noc1-c1ccco1)C(=O)O | alpha | 0.5946 | 238.199 | TRUE | TRUE |
| CHEMBL84132 | NC(Cc1c(O)noc1-c1ccccn1)C(=O)O | alpha | 0.3966 | 249.226 | TRUE | FALSE |
| CHEMBL84432 | C[C@@H](NCc1cc(Br)cc2nc(O)c(O)nc12)C(=O)O | alpha | 1.3662 | 342.149 | TRUE | TRUE |
| CHEMBL84589 | NCCCCCCOc1ccc(C[C@H](N)C(=O)O)cc1 | alpha | 1.5389 | 280.368 | TRUE | TRUE |
| CHEMBL84746 | NC(CCC(N)(F)F)C(=O)O | alpha | -0.27 | 168.143 | FALSE | FALSE |
| CHEMBL84788 | NC(CC1CCC(CS(=O)(=O)[O-])CC1)C(=O)O.[Na+] | alpha | -2.856 | 287.313 | FALSE | FALSE |
| CHEMBL85169 | NC(CC1CCCCC1CP(=O)(O)O)C(=O)O | alpha | 0.7725 | 265.246 | FALSE | FALSE |
| CHEMBL85175 | O=C(O)CN1CCCCC(NC(CCc2ccccc2)C(=O)O)C1=O | alpha | 1.1277 | 348.399 | TRUE | FALSE |
| CHEMBL85257 | NC(Cc1ccccc1CS(=O)(=O)[O-])C(=O)O.[Na+] | alpha | -3.3098 | 281.265 | TRUE | FALSE |
| CHEMBL85258 | NC(Cc1cccc(CS(=O)(=O)[O-])c1)C(=O)O.[Na+] | alpha | -3.3098 | 281.265 | TRUE | FALSE |
| CHEMBL85331 | NC(Cc1c(O)noc1-c1ccncc1)C(=O)O | alpha | 0.3966 | 249.226 | TRUE | FALSE |
| CHEMBL85688 | NC(Cc1c(O)noc1-c1ccco1)C(=O)O | alpha | 0.5946 | 238.199 | TRUE | FALSE |
| CHEMBL86216 | N[C@@H](Cc1c(O)noc1-c1ccco1)C(=O)O | alpha | 0.5946 | 238.199 | TRUE | TRUE |
| CHEMBL86371 | N[C@H](C/C(CP(=O)(O)O)=N/O)C(=O)O | alpha | -1.2037 | 226.125 | FALSE | TRUE |
| CHEMBL86395 | CC(C(=O)C(C)P(=O)(O)O)C(N)C(=O)O | alpha | -0.8303 | 239.164 | FALSE | FALSE |
| CHEMBL86396 | CC(C(=O)C[C@@H](N)C(=O)O)P(=O)(O)O | alpha | -1.0763 | 225.137 | FALSE | TRUE |
| CHEMBL86862 | COP(=O)(O)CC(=O)CC(N)C(=O)O | alpha | -0.8107 | 225.137 | FALSE | FALSE |
| CHEMBL86891 | O=C(O)CN1CCC(NC(CCc2ccccc2)C(=O)O)C1=O | alpha | 0.3475 | 320.345 | TRUE | FALSE |
| CHEMBL87815 | NC(Cc1cccc(CC(=O)O)c1)C(=O)O | alpha | 0.2681 | 223.228 | TRUE | FALSE |
| CHEMBL88124 | NC(C(=O)O)c1cccc(O)c1 | alpha | 0.4766 | 167.164 | TRUE | FALSE |
| CHEMBL88165 | Cc1ccc([N+](=O)[O-])c(NCCC[C@@H](N)C(=O)O)c1 | alpha | 1.50722 | 267.285 | TRUE | TRUE |
| CHEMBL88166 | Cc1ccc([N+](=O)[O-])c(NCCC[C@H](N)C(=O)O)c1 | alpha | 1.50722 | 267.285 | TRUE | TRUE |
| CHEMBL88177 | Cc1ccc([N+](=O)[O-])c(SCCC[C@H](N)C(=O)O)c1 | alpha | 2.18742 | 284.337 | TRUE | TRUE |
| CHEMBL88178 | N[C@@H](CCCNc1ccc(C(=O)O)cc1[N+](=O)[O-])C(=O)O | alpha | 0.897 | 297.267 | TRUE | TRUE |
| CHEMBL88184 | N[C@@H](Cc1cc(O)no1)C(=O)O | alpha | -0.6654 | 172.14 | TRUE | TRUE |
| CHEMBL88309 | Cc1cccc(NCCC[C@H](N)C(=O)O)c1 | alpha | 1.59902 | 222.288 | TRUE | TRUE |
| CHEMBL88626 | Nc1ccccc1S(=O)(=O)C[C@@H](N)C(=O)O | alpha | -0.5456 | 244.272 | TRUE | TRUE |
| CHEMBL88665 | Cc1ccc([N+](=O)[O-])c(OCCC[C@H](N)C(=O)O)c1 | alpha | 1.47412 | 268.269 | TRUE | TRUE |
| CHEMBL89370 | COc1ccc([N+](=O)[O-])c(NCCC[C@H](N)C(=O)O)c1 | alpha | 1.2074 | 283.284 | TRUE | TRUE |
| CHEMBL89491 | N[C@@H](CCCNc1ccc([N+](=O)[O-])cc1[N+](=O)[O-])C(=O)O | alpha | 1.107 | 298.255 | TRUE | TRUE |
| CHEMBL89696 | N[C@@H](CCCNc1ccc(C(F)(F)F)cc1[N+](=O)[O-])C(=O)O | alpha | 2.2176 | 321.255 | TRUE | TRUE |
| CHEMBL89746 | CO/N=C(/C[C@@H](N)C(=O)O)CP(=O)(O)O | alpha | -1.0315 | 240.152 | FALSE | TRUE |
| CHEMBL9038 | NC(Cc1ccccc1OCC(=O)O)C(=O)O | alpha | 0.1044 | 239.227 | TRUE | FALSE |
| CHEMBL90564 | O=C(O)[C@H]1CN(C/C=C/P(=O)(O)O)CCN1 | alpha | -0.9638 | 250.191 | FALSE | TRUE |
| CHEMBL90963 | Cc1cccc(SCCC[C@H](N)C(=O)O)c1 | alpha | 2.27922 | 239.34 | TRUE | TRUE |
| CHEMBL91734 | N[C@@H](CCCNc1cc(O)ccc1[N+](=O)[O-])C(=O)O | alpha | 0.9044 | 269.257 | TRUE | TRUE |
| CHEMBL91860 | NC(C(=O)O)C(O)C(F)F | alpha | -0.9757 | 155.1 | FALSE | FALSE |
| CHEMBL92293 | NC(/C=C\CP(=O)(O)O)C(=O)O | alpha | -0.8678 | 195.111 | FALSE | FALSE |
| CHEMBL92448 | CCOP(=S)(NCCC[C@H](N)C(=O)O)OCC | alpha | 1.0656 | 284.318 | FALSE | TRUE |
| CHEMBL94205 | N[C@@H](CCC(=O)CCl)C(=O)O | alpha | -0.0136 | 179.603 | FALSE | TRUE |
| CHEMBL94305 | N[C@@H](CCC(=O)CBr)C(=O)O | alpha | 0.1425 | 224.054 | FALSE | TRUE |
| CHEMBL94631 | Cc1cc(C(=O)O)ccc1[C@H](N)C(=O)O | alpha | 0.77762 | 209.201 | TRUE | TRUE |
| CHEMBL94816 | NC(CC#CP(=O)(O)O)C(=O)O | alpha | -1.073 | 193.095 | FALSE | FALSE |
| CHEMBL94860 | NC(Cc1c(O)noc1-c1nn[nH]n1)C(=O)O | alpha | -1.4853 | 240.179 | TRUE | FALSE |
| CHEMBL94909 | NC(C/C=C\P(=O)(O)O)C(=O)O | alpha | -0.5202 | 195.111 | FALSE | FALSE |
| CHEMBL94990 | N[C@H](C(=O)O)c1ccc(C(=O)O)cc1 | alpha | 0.4692 | 195.174 | TRUE | TRUE |
| CHEMBL95033 | NC(/C=C/CP(=O)(O)O)C(=O)O | alpha | -0.8678 | 195.111 | FALSE | FALSE |
| CHEMBL95094 | O=C[C@@H](NS(=O)(=O)c1ccccc1-c1ccccc1)C(=O)O | alpha | 1.284 | 319.338 | TRUE | TRUE |
| CHEMBL9515 | NC(Cc1onc(O)c1-c1ccccc1)C(=O)O | alpha | 1.0016 | 248.238 | TRUE | FALSE |
| CHEMBL95682 | O=C(O)C1CCC(P(=O)(O)O)N1 | alpha | -0.6732 | 195.111 | FALSE | FALSE |
| CHEMBL96101 | NC(COCP(=O)(O)O)C(=O)O | alpha | -1.4499 | 199.099 | FALSE | FALSE |
| CHEMBL96181 | Cc1cccc(Oc2ccccc2S(=O)(=O)N[C@H](C=O)C(=O)O)c1 | alpha | 1.71772 | 349.364 | TRUE | TRUE |
| CHEMBL96624 | N/C(=N\CCCC(N)C(=O)O)[N+](=O)[O-] | alpha | -1.2301 | 204.186 | FALSE | FALSE |
| CHEMBL96698 | NC(CNCP(=O)(O)O)C(=O)O | alpha | -1.8769 | 198.115 | FALSE | FALSE |
| CHEMBL9698 | N[C@@H](Cc1onc(O)c1-c1ccccc1)C(=O)O | alpha | 1.0016 | 248.238 | TRUE | TRUE |
| CHEMBL97485 | [N-]=[N+]=CC(=O)CC[C@H](N)C(=O)O | alpha | -0.9518 | 171.156 | FALSE | TRUE |
| CHEMBL9752 | CCCCCCCc1c(O)noc1CC(N)C(=O)O | alpha | 1.8475 | 270.329 | TRUE | FALSE |
| CHEMBL9758 | CCCCCCc1c(O)noc1CC(N)C(=O)O | alpha | 1.4574 | 256.302 | TRUE | FALSE |
| CHEMBL97742 | NC(Cc1c(O)noc1-c1ncc[nH]1)C(=O)O | alpha | -0.2753 | 238.203 | TRUE | FALSE |
| CHEMBL9777 | N[C@H](Cc1onc(O)c1-c1ccccc1)C(=O)O | alpha | 1.0016 | 248.238 | TRUE | TRUE |
| CHEMBL9822 | CCCCCCCCc1c(O)noc1CC(N)C(=O)O | alpha | 2.2376 | 284.356 | TRUE | FALSE |
| CHEMBL98445 | O=C(O)C1CN(Cc2cccc(CP(=O)(O)O)c2)CCN1 | alpha | 0.2227 | 314.278 | TRUE | FALSE |
| CHEMBL98723 | Cn1ccnc1-c1onc(O)c1CC(N)C(=O)O | alpha | -0.2649 | 252.23 | TRUE | FALSE |
| CHEMBL98731 | CC(NCP(=O)(O)O)C(=O)O | alpha | -0.8157 | 183.1 | FALSE | FALSE |
| CHEMBL98814 | O=C(O)CN1CCNC(C(=O)O)C1 | alpha | -1.5706 | 188.183 | FALSE | FALSE |
| CHEMBL98870 | CP(=O)(O)CCCN1CCNC(C(=O)O)C1 | alpha | -0.3649 | 250.235 | FALSE | FALSE |
| CHEMBL9907 | NC(Cc1cc(O)no1)C(=O)O | alpha | -0.6654 | 172.14 | TRUE | FALSE |
| CHEMBL99380 | O=C(O)c1ccccc1C(=O)N1CCNC(C(=O)O)C1 | alpha | -0.1166 | 278.264 | TRUE | FALSE |
| CHEMBL99636 | CN(CCC[C@H](N)C(=O)O)C(=N)N | alpha | -0.99623 | 188.231 | FALSE | TRUE |
| CHEMBL105300 | NCC(NC(=O)[C@@H](N)CN)C(=O)O | beta | -3.1995 | 190.203 | FALSE | TRUE |
| CHEMBL1087229 | CN(C)CCNC(=O)[C@@H]1CNC[C@@H]1C(=O)O | beta | -1.4156 | 229.28 | FALSE | TRUE |
| CHEMBL1087357 | CCCCNC(=O)[C@@H]1CNC[C@@H]1C(=O)O | beta | -0.1771 | 214.265 | FALSE | TRUE |
| CHEMBL1095791 | O=C(O)[C@H]1CNC[C@H]1C(=O)O | beta | -1.0088 | 159.141 | FALSE | TRUE |
| CHEMBL1189195 | O=C(O)[C@@H]1CNCC12CCCCC2 | beta | 1.2409 | 183.251 | FALSE | TRUE |
| CHEMBL1194738 | O=C(O)[C@H]1CNCC12CCCCC2 | beta | 1.2409 | 183.251 | FALSE | TRUE |
| CHEMBL1195389 | CC(CNc1ccccc1C(=O)c1ccccc1)C(=O)O | beta | 3.0502 | 283.327 | TRUE | FALSE |
| CHEMBL1203754 | Cl.NCC(C(=O)O)c1c[nH]c2ccc(Br)cc12 | beta | 2.4791 | 319.586 | TRUE | FALSE |
| CHEMBL1213526 | O=C(O)[C@@H]1CNC(Cc2ccc(Cl)cc2)=N1 | beta | 1.3374 | 238.674 | TRUE | TRUE |
| CHEMBL1347633 | CC(CNc1ccccc1O)C(=O)O | beta | 1.5248 | 195.218 | TRUE | FALSE |
| CHEMBL137399 | CC(C)(CN)C(CN)C(=O)O | beta | -0.3692 | 160.217 | FALSE | FALSE |
| CHEMBL139503 | NCCC(CN)C(=O)O | beta | -1.0053 | 132.163 | FALSE | FALSE |
| CHEMBL146705 | NS(=O)(=O)NCC(Cc1ccccc1)C(=O)O | beta | -0.277 | 258.299 | TRUE | FALSE |
| CHEMBL146790 | NS(=O)(=O)NC[C@@H](Cc1ccccc1)C(=O)O | beta | -0.277 | 258.299 | TRUE | TRUE |
| CHEMBL15851 | O=C(O)[C@H]1CNC[C@@H](O)C1 | beta | -0.9586 | 145.158 | FALSE | TRUE |
| CHEMBL161941 | COc1ccc2[nH]cc(C(CN)C(=O)O)c2c1 | beta | 1.3034 | 234.255 | TRUE | FALSE |
| CHEMBL16226 | O=C(O)[C@H]1CNCC[C@@H]1O | beta | -0.9586 | 145.158 | FALSE | TRUE |
| CHEMBL162823 | NCC(C(=O)O)c1c[nH]c2ccccc12 | beta | 1.2948 | 204.229 | TRUE | FALSE |
| CHEMBL164197 | NCC(C(=O)O)c1c[nH]c2ccc(Br)cc12 | beta | 2.0573 | 283.125 | TRUE | FALSE |
| CHEMBL1977251 | COc1ccc(C)c(NCC(C)C(=O)O)c1 | beta | 2.13622 | 223.272 | TRUE | FALSE |
| CHEMBL1980212 | CC(CNc1ccc(Oc2ccccc2)cc1)C(=O)O | beta | 3.6115 | 271.316 | TRUE | FALSE |
| CHEMBL1983911 | COc1cc(NCC(C)C(=O)O)cc(OC)c1OC | beta | 1.845 | 269.297 | TRUE | FALSE |
| CHEMBL1992481 | CC(CNc1ccccc1Cl)C(=O)O | beta | 2.4726 | 213.664 | TRUE | FALSE |
| CHEMBL2000484 | CC(CNC(C)c1ccccc1)C(=O)O | beta | 2.0579 | 207.273 | TRUE | FALSE |
| CHEMBL2002363 | Cc1ccc(NCC(C)C(=O)O)cc1C | beta | 2.43604 | 207.273 | TRUE | FALSE |
| CHEMBL201333 | O=C(O)[C@H]1CN[C@H](NC(=O)C(F)(F)F)[C@@H](O)[C@@H]1O | beta | -1.9832 | 272.179 | FALSE | TRUE |
| CHEMBL202303 | CC(=O)N[C@H]1NC[C@@H](C(=O)O)[C@H](N)[C@@H]1O | beta | -2.5592 | 217.225 | FALSE | TRUE |
| CHEMBL20723 | N=C(N)NCC(Cc1ccccc1)C(=O)O | beta | 0.41297 | 221.26 | TRUE | FALSE |
| CHEMBL20887 | N=C(N)NCC(S)C(=O)O | beta | -1.14753 | 163.202 | FALSE | FALSE |
| CHEMBL20949 | N=C(N)NCC(O)C(=O)O | beta | -2.08503 | 147.134 | FALSE | FALSE |
| CHEMBL2115223 | CC(=O)N[C@H]1NC[C@@H](C(=O)O)[C@H](N)[C@H]1O | beta | -2.5592 | 217.225 | FALSE | TRUE |
| CHEMBL2347885 | O=C(O)C[C@H]1CNC[C@@H]1C(=O)O | beta | -0.6187 | 173.168 | FALSE | TRUE |
| CHEMBL2349245 | O=C(O)C1CNC(NC(=O)C(F)(F)F)C(O)C1O | beta | -1.9832 | 272.179 | FALSE | FALSE |
| CHEMBL277372 | O=C(O)[C@H]1CNCC[C@H]1O | beta | -0.9586 | 145.158 | FALSE | TRUE |
| CHEMBL280511 | CC(=O)N[C@@H]1CCNC[C@@H]1C(=O)O | beta | -0.8148 | 186.211 | FALSE | TRUE |
| CHEMBL282617 | CNCC(C)C(=O)O | beta | -0.0735 | 117.148 | FALSE | FALSE |
| CHEMBL310435 | O=C(O)C1CNC1 | beta | -0.7096 | 101.105 | FALSE | FALSE |
| CHEMBL312866 | NC1C(C(=O)O)CNC(NC(=O)C(F)(F)F)C1O | beta | -2.0168 | 271.195 | FALSE | FALSE |
| CHEMBL3251903 | NCC(C(=O)O)c1ccc(N2CCCCC2)cc1 | beta | 1.8038 | 248.326 | TRUE | FALSE |
| CHEMBL327967 | NCC[C@H](N)C(=O)NC(CN)C(=O)O | beta | -2.8094 | 204.23 | FALSE | TRUE |
| CHEMBL3322884 | O=C(O)C1CN=CNC1 | beta | -0.6813 | 128.131 | FALSE | FALSE |
| CHEMBL3322886 | NC1=NCC(C(=O)O)CN1 | beta | -1.3949 | 143.146 | FALSE | FALSE |
| CHEMBL3403592 | CCN1CCNCC1C(=O)O | beta | -0.6353 | 158.201 | FALSE | FALSE |
| CHEMBL344906 | CC(CN)C(CN)C(=O)O | beta | -0.7593 | 146.19 | FALSE | FALSE |
| CHEMBL348084 | O=C(O)C1CNCCC1O | beta | -0.9586 | 145.158 | FALSE | FALSE |
| CHEMBL348748 | NCC(C(=O)O)c1c[nH]c2ccc(F)cc12 | beta | 1.4339 | 222.219 | TRUE | FALSE |
| CHEMBL3544571 | NCC(F)C(=O)O | beta | -0.6322 | 107.084 | FALSE | FALSE |
| CHEMBL3665067 | COc1cc(Br)cc(C2NCC(C(=O)O)S2)c1O | beta | 1.9515 | 334.191 | TRUE | FALSE |
| CHEMBL370619 | CC(=O)N[C@H]1NC[C@@H](C(=O)O)[C@H](N=C(N)N)[C@@H]1O | beta | -3.2445 | 259.266 | FALSE | TRUE |
| CHEMBL370990 | O=C(O)[C@@H]1CN[C@H](NC(=O)C(F)(F)F)[C@@H](O)[C@@H]1O | beta | -1.9832 | 272.179 | FALSE | TRUE |
| CHEMBL371321 | O=C(O)[C@H]1CN[C@H](NC(=O)C(F)(F)F)[C@@H](O)[C@H]1O | beta | -1.9832 | 272.179 | FALSE | TRUE |
| CHEMBL371971 | O=C(O)[C@@H]1CN[C@@H](NC(=O)C(F)(F)F)[C@@H](O)[C@@H]1O | beta | -1.9832 | 272.179 | FALSE | TRUE |
| CHEMBL3774496 | C#CCCC[C@H]1CCNC[C@@H]1C(=O)O | beta | 1.1002 | 195.262 | FALSE | TRUE |
| CHEMBL3774788 | CCCC[C@H]1CCNC[C@@H]1C(=O)O | beta | 1.4869 | 185.267 | FALSE | TRUE |
| CHEMBL3774880 | C#CCC[C@H]1CCNC[C@@H]1C(=O)O | beta | 0.7101 | 181.235 | FALSE | TRUE |
| CHEMBL3775161 | C=CCC[C@H]1CCNC[C@@H]1C(=O)O | beta | 1.2629 | 183.251 | FALSE | TRUE |
| CHEMBL3775515 | C=CCCCC[C@H]1CCNC[C@@H]1C(=O)O | beta | 2.0431 | 211.305 | FALSE | TRUE |
| CHEMBL429974 | CC(=O)N[C@@H]1CNC[C@H](C(=O)O)C1 | beta | -0.8148 | 186.211 | FALSE | TRUE |
| CHEMBL441297 | NCC(C(=O)O)c1c[nH]c2cc(Cl)ccc12 | beta | 1.9482 | 238.674 | TRUE | FALSE |
| CHEMBL4436035 | Cl.O=C(O)[C@H]1CNC[C@@H](O)[C@H](O)[C@H]1O | beta | -2.2051 | 227.644 | FALSE | TRUE |
| CHEMBL4447893 | O=C(O)[C@H]1CNC[C@@H](CN/N=C/c2cccs2)C1 | beta | 0.9819 | 267.354 | TRUE | TRUE |
| CHEMBL4457266 | NN[C@@H]1CNC[C@H](C(=O)O)C1 | beta | -1.4876 | 159.189 | FALSE | TRUE |
| CHEMBL4465595 | NNC[C@H]1CNC[C@H](C(=O)O)C1 | beta | -1.24 | 173.216 | FALSE | TRUE |
| CHEMBL4471080 | COc1ccc(/C=N/NC[C@@H]2CNC[C@H](C(=O)O)C2)cc1 | beta | 0.929 | 291.351 | TRUE | TRUE |
| CHEMBL4471899 | NNCC[C@H]1CNC[C@H](C(=O)O)C1 | beta | -0.8499 | 187.243 | FALSE | TRUE |
| CHEMBL4474296 | NNCC[C@@H]1CNC[C@H](C(=O)O)C1 | beta | -0.8499 | 187.243 | FALSE | TRUE |
| CHEMBL4544286 | NNC[C@@H]1CNC[C@H](C(=O)O)C1 | beta | -1.24 | 173.216 | FALSE | TRUE |
| CHEMBL4545282 | O=C(O)[C@H]1CNC[C@@H](CN/N=C/c2cc(Br)co2)C1 | beta | 1.2759 | 330.182 | TRUE | TRUE |
| CHEMBL4554622 | O=C(O)[C@H]1CNC[C@@H](CN/N=C/c2cncnc2)C1 | beta | -0.2896 | 263.301 | TRUE | TRUE |
| CHEMBL4577771 | O=C(O)[C@H]1CNC[C@@H](CN/N=C/c2ccc[nH]2)C1 | beta | 0.2485 | 250.302 | TRUE | TRUE |
| CHEMBL4581945 | O=C(O)[C@H]1CNC[C@@H](CN/N=C/c2ccc([N+](=O)[O-])cc2)C1 | beta | 0.8286 | 306.322 | TRUE | TRUE |
| CHEMBL4585359 | O=C(O)[C@H]1CNC[C@@H](CCN/N=C/c2ccco2)C1 | beta | 0.9035 | 265.313 | TRUE | TRUE |
| CHEMBL4586784 | O=C(O)C1CCNC1 | beta | -0.3195 | 115.132 | FALSE | FALSE |
| CHEMBL4593108 | O=C(O)[C@H]1CNC[C@@H](CN/N=C/c2ccc(Br)o2)C1 | beta | 1.2759 | 330.182 | TRUE | TRUE |
| CHEMBL4593330 | COc1ccccc1/C=N/NC[C@@H]1CNC[C@H](C(=O)O)C1 | beta | 0.929 | 291.351 | TRUE | TRUE |
| CHEMBL4595354 | O=C(O)[C@H]1CNC[C@@H](O)[C@H](O)[C@H]1O | beta | -2.6269 | 191.183 | FALSE | TRUE |
| CHEMBL4869708 | O=C(O)C1CNc2ccccc2O1 | beta | 0.9441 | 179.175 | TRUE | FALSE |
| CHEMBL4908604 | CC[C@@H](CNc1cc(NC2CC2)ncn1)C(=O)O | beta | 1.5736 | 250.302 | TRUE | TRUE |
| CHEMBL4926868 | CC(C)[C@H](CNS(=O)(=O)c1ccc2c(c1)CC(C)(C)O2)C(=O)O | beta | 2.0352 | 341.429 | TRUE | TRUE |
| CHEMBL4970112 | CCc1ccccc1S(=O)(=O)NC[C@H](C(=O)O)C(C)C | beta | 1.8841 | 299.392 | TRUE | TRUE |
| CHEMBL4976510 | CC(C)c1cc(NC[C@@H](C(=O)O)c2ccccc2)ncn1 | beta | 2.8803 | 285.347 | TRUE | TRUE |
| CHEMBL4984703 | Cc1ccc(C)c(S(=O)(=O)NC[C@H](Cc2ccccn2)C(=O)O)c1 | beta | 1.92024 | 348.424 | TRUE | TRUE |
| CHEMBL538255 | Cl.O=C(O)[C@@H]1CNCC12CCCCC2 | beta | 1.6627 | 219.712 | FALSE | TRUE |
| CHEMBL553219 | Cl.O=C(O)[C@H]1CNCC12CCCCC2 | beta | 1.6627 | 219.712 | FALSE | TRUE |
| CHEMBL554438 | CC(CNc1ccccc1C(=O)c1ccccc1)C(=O)O.Cl | beta | 3.472 | 319.788 | TRUE | FALSE |
| CHEMBL89787 | CC(=O)NC1NCC(C(=O)O)C(N=C(N)N)C1O | beta | -3.2445 | 259.266 | FALSE | FALSE |
| CHEMBL103698 | NCCC(NC(=O)[C@@H](N)CCN)C(=O)O | gamma | -2.4193 | 218.257 | FALSE | TRUE |
| CHEMBL118068 | CCCCCCCCCc1ccc(CNCCC(O)C(=O)O)cc1 | gamma | 3.9049 | 335.488 | TRUE | FALSE |
| CHEMBL1191543 | O=C(O)C1CCNCC12CCCCC2 | gamma | 1.631 | 197.278 | FALSE | FALSE |
| CHEMBL16132 | O=C(O)[C@@H]1CCNC[C@@H]1O | gamma | -0.9586 | 145.158 | FALSE | TRUE |
| CHEMBL16357 | O=C(O)[C@@H]1CCNC[C@H]1O | gamma | -0.9586 | 145.158 | FALSE | TRUE |
| CHEMBL256608 | C[C@@H](CCN)C(=O)O | gamma | 0.0559 | 117.148 | FALSE | TRUE |
| CHEMBL257443 | C[C@H](CCN)C(=O)O | gamma | 0.0559 | 117.148 | FALSE | TRUE |
| CHEMBL363611 | CC(C)C[C@@H](CCN)C(=O)O | gamma | 1.0821 | 159.229 | FALSE | TRUE |
| CHEMBL419546 | NCCC(NC(=O)[C@@H](N)CN)C(=O)O | gamma | -2.8094 | 204.23 | FALSE | TRUE |
| CHEMBL542655 | Cl.O=C(O)C1CCNCC12CCCCC2 | gamma | 2.0528 | 233.739 | FALSE | FALSE |
| CHEMBL5606914 | Cc1cc(CO)c2cccc(CC(=O)N[C@@H](CCN)C(=O)O)c2n1 | gamma | 0.49622 | 331.372 | TRUE | TRUE |


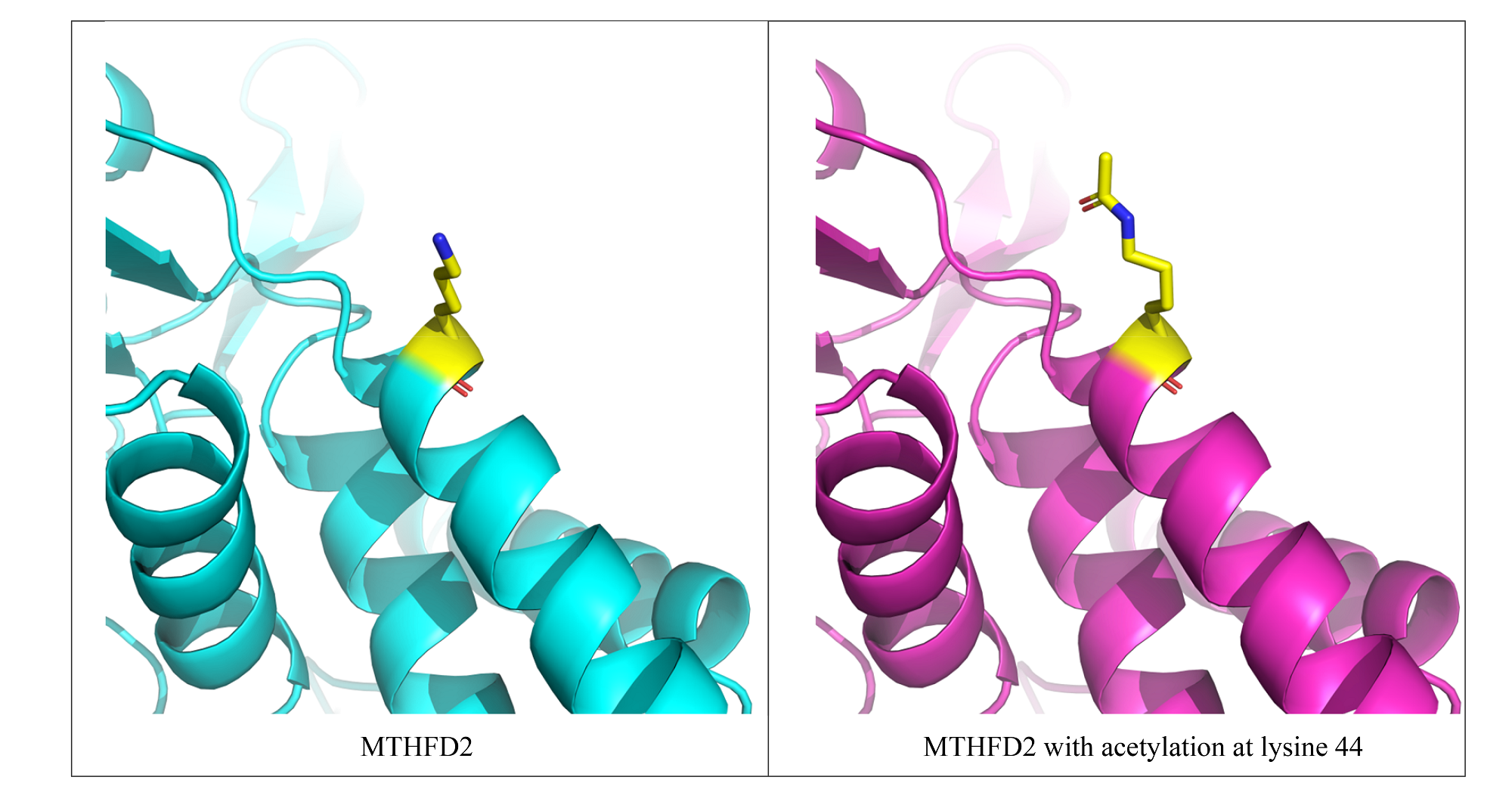


Figure S1. Predicted structures of MTHFD2 and its acetylated form generated by AlphaFold3.


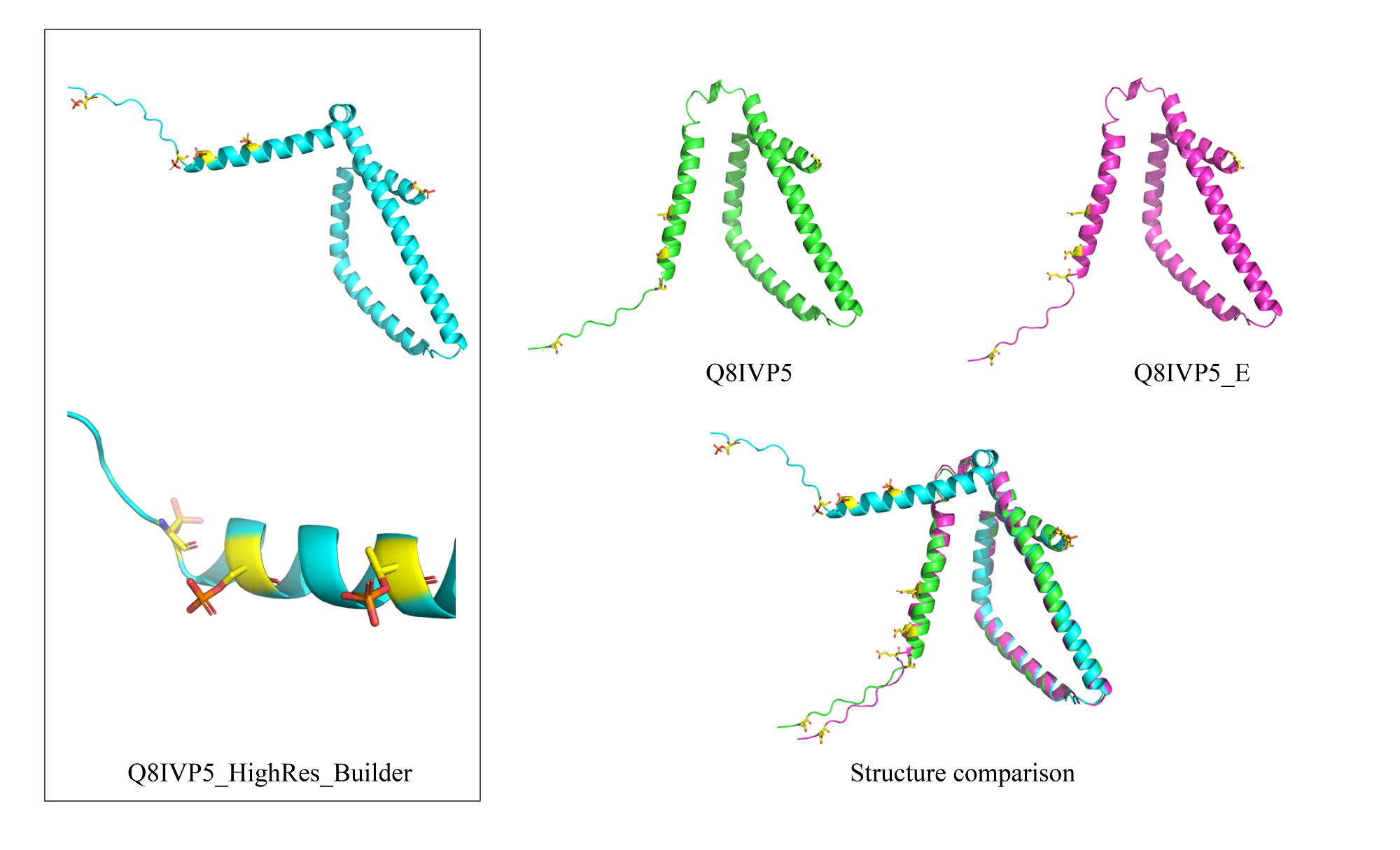


Figure S2. Predicted structures of Q8IVP5, including its wild-type, phosphomimetic mutant (S/T to E), and direct phosphomimetic residue forms, generated by AlphaFold3.
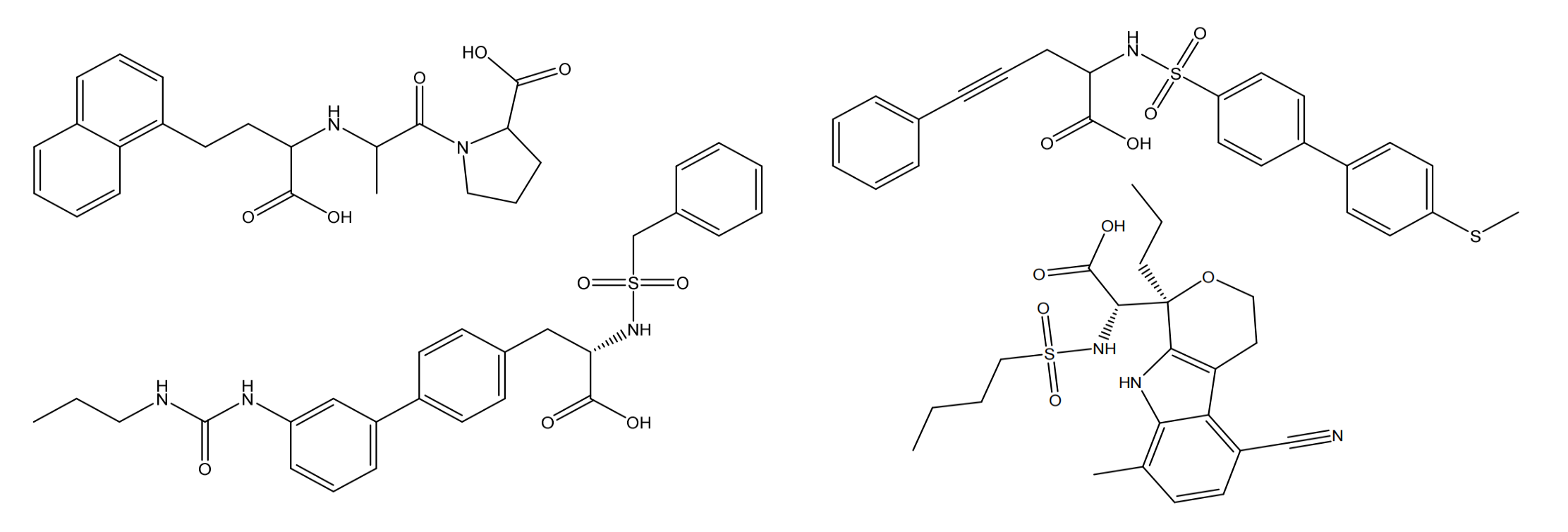


Figure S3. Representative examples of structures excluded from our dataset.


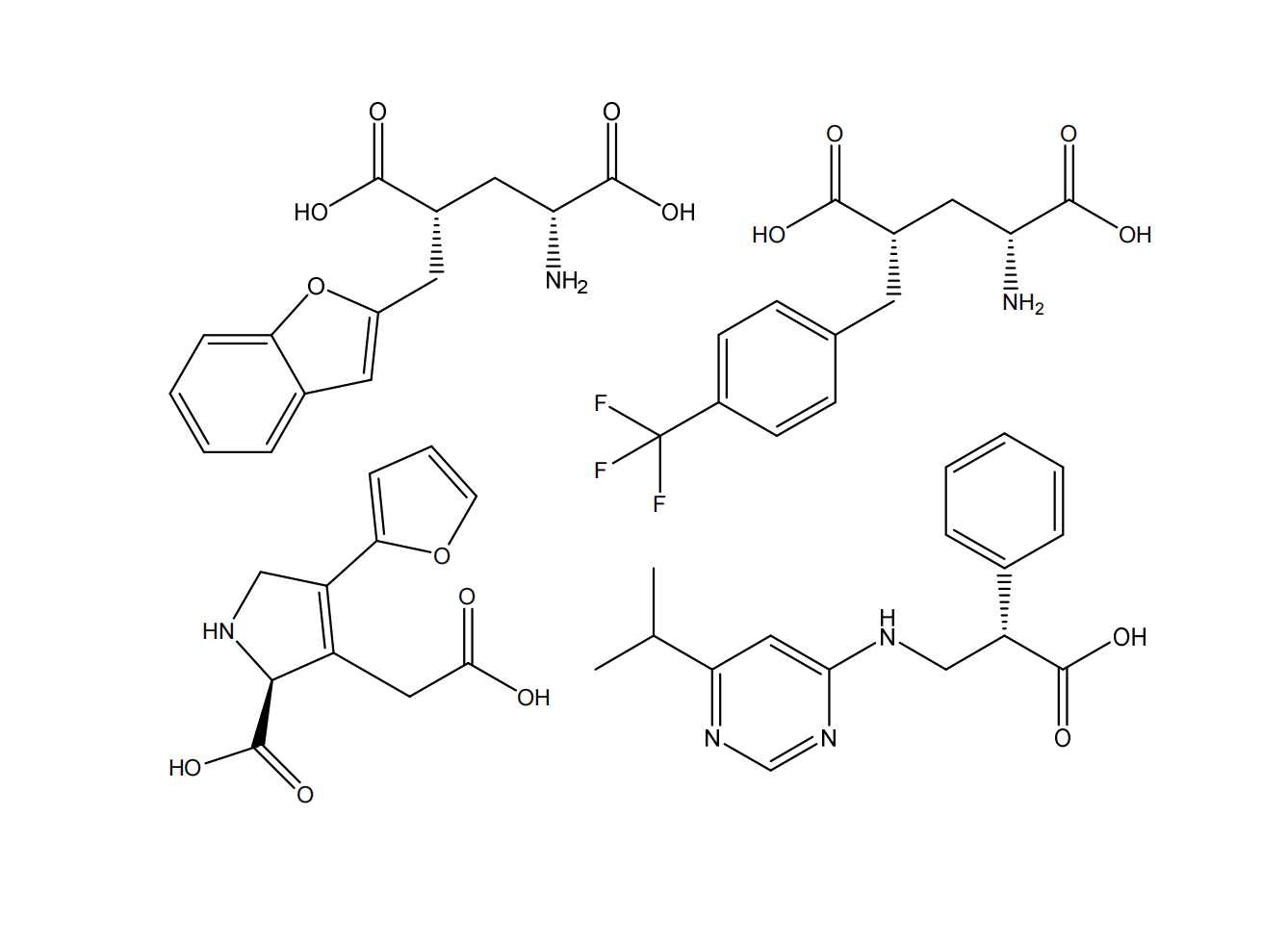


Figure S4. Representative examples of structures included from our dataset.
